# Effect of dietary Astragalus Polysaccharide supplements on testicular miRNA expression profiles and enzymatic changes of breeder cocks

**DOI:** 10.1038/srep38864

**Published:** 2017-01-05

**Authors:** Shengru Wu, Xiaochun Ren, Yulong Li, Wei Guo, Xinyu Lei, Junhu Yao, Xiaojun Yang

**Affiliations:** 1 College of Animal Science and Technology, Northwest A&F University, Yangling 712100, China

## Abstract

Astragalus Polysaccharide (APS) is an important feed additive due to its immunomodulatory functions. Previous studies have proven that miRNAs play important roles in posttranscriptional gene regulation. Our goals were to identify differentially expressed miRNAs in testes in responses to APS dietary supplements and to find the effects of APS on breeder cock testes. We measured several enzymatic activities in testes and sperm samples and further generated miRNA expression profiles of testes from breeder cocks fed with control diets and extra APS. As a result, we found APS could increase testicular functional activities of marker enzymes. Meanwhile, there were 16 up-regulated and 17 down-regulated miRNAs in APS group, compared with the control group meeting the criteria of P-values < 0.05. Meanwhile, twelve differentially expressed miRNAs were validated by Mir-XTM miRNA RT-qPCR. Further GO and KEGG analyses of target genes for differentially expressed miRNAs revealed that some miRNAs may be involved in testicular nutrient metabolisms and NK cell mediated cytotoxicity pathway. Moreover, the effect of dietary APS supplements on NK cell mediated cytotoxicity pathway was also validated by RT-qPCR. Our results provided a novel insight into the effect of dietary APS supplements on testicular miRNA expression profiles and enzymatic changes of breeder cocks.

Astragalus membranaceus has been used for thousands of years as a traditional Chinese medicine to enhance innate immune functions. Astragalus polysaccharide (APS) is the main active ingredient in Astragalus membranaceus and has been widely used as a safe antibiotic alternative in feed additive due to its extensive biological activities, such as anti-inflammatory^1^, anti-carcinogenic^2^, anti-virus^3^, and immunomodulatory^4^. In addition, during the last few years, researches have shown that APS was responsible for changing animal performances and nutritional metabolisms^5^,^6^. Therefore, APS could play a regulatory role in changing gene expressions of the immune and metabolic systems.

MiRNAs, consistent with 18 to 26 nucleotides (nt), are a major class of noncoding RNA, which play a crucial role in post-transcriptional gene regulation^7^. To date, thousands of miRNAs have been identified by many methods, such as RT-PCR^8^, northern blotting^9^, microarrays^10^, the RNA-primed, array-based Klenow enzyme (RAKE) assay^11^, and the next generation sequencing^12^. It is well known that expressions of approximately thirty percent of protein-coding genes are regulated by miRNAs^13^, and the expressions of genes associated with testicular development and functions are regulated by many miRNAs, which could regulate gene expressions at either the transcriptional or post-transcriptional levels by RNA-RNA interactions^14^. Yan *et al*.^15^ compared the differentially expressed miRNAs in testes between juvenile and adult mice, and identified 14 up-expressed miRNAs (including let-7e) and 5 down-expressed miRNAs (miR-34a, miR-34b, miR-34c, miR-29b, and miR-449). When piglets and adult pigs were tested, 122 differentially expressed miRNAs were reported in testes, including miR-30a and the let-7 family members^16^. These differentially expressed miRNAs in testicular specific development phases could play an important role in regulation of testicular functions. However, the main miRNAs in chicken testes and differentially expressed miRNAs, which could have been induced by dietary APS supplements, remain unknown.

Testes providing the environment for spermatogenesis and better testicular condition are essential for better reproductive ability. Additionally, the metabolic and immune conditions in testes, as well as testicular development play important roles in the stabilization of testes and testicular functions^17^,^18^. As an important feed additive, containing immunomodulatory and metabolic regulation activities, adequate knowledge of the effects of dietary APS supplements on the testes and the regulatory mechanisms of these effects are essential to use the APS better in breeder cock feeding. Additionally, more and more articles have verified that different nutritional supplements, or dietary intake, could change the expression of miRNAs and further regulate metabolic and immune process^19^,^20^. As APS’s potential roles emerge in the metabolic and immune changes of breeder cocks’ testes, identifying differentially expressed miRNAs induced by dietary APS is an important step in investigating the effect of APS on testicular metabolic and immune changes, as well as clarifying the potential regulatory roles of miRNAs in these testicular changes.

Hence, we hypothesized that APS supplements could change the miRNA expression profiles of breeder cock, which is the potential mechanism to further influence the testes condition. Herein, we focused on enzymatic activities changes related to testicular functions and identified miRNAs related to dietary APS supplements by using 6 testes miRNA transcriptome libraries from Cobb500 breeder cocks. Taken together, our results suggest the roles of microRNAs in the interactions between breeder cock testes and dietary APS supplements.

## Results

### Enzymatic activities in testes and sperm samples

Effects of dietary APS supplements on enzymatic activities of breeder cock testes and sperm are showed in Tables 1 and 2. In this study, dietary APS supplements significantly increased a portion of the testicular metabolic enzymatic activities, including acid phosphatase (ACP, *P* = 0.001), succinodehydrogenase (SDH, *P* = 0.007), and lactate dehydrogenase (LDH, *P* = 0.020). Simultaneously, glutathione peroxidase (GSH-px) and total superoxide dismutase (T-SOD) significantly increased (*P* = 0.006, *P* = 0.002), showing no significant changes in malonaldehyde (MDA); however, there were no relevant changes to alkaline phosphatase (AKP) and testosterone concentrations in testes (Table 1). Furthermore, LDH activity in sperm also significantly increased (*P* = 0.002); whereas, ACP, AKP, and SDH activities showed no significant changes (Table 2).

### Overview of miRNA expression profiles in testes tissues of breeder cocks

**Figure 1:**
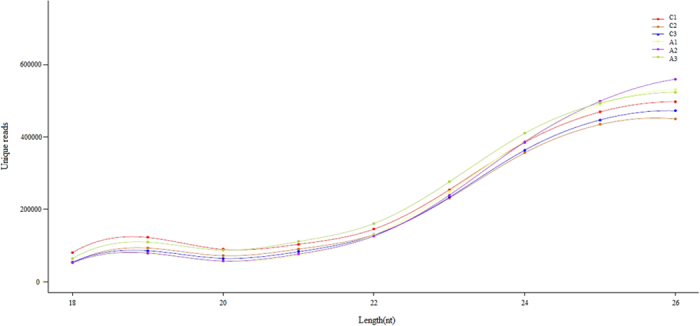
Size distribution of small RNAs in C and A libraries.

To systematically identify small RNAs and differentially expressed miRNAs between the testes from breeder cocks with dietary APS supplements (A group) and without dietary APS supplements (C group), we generated six miRNA expression profiles of testes tissues from control individuals and breeder cocks with dietary APS supplements. The numbers of raw reads of these six transcriptomes ranged from 10 to 15 million (Table 3). After filtering for reads with 3′Adaptor and length filters, junk reads, Rfam, mRNA, repeats, rRNA, tRNA, snRNA and snoRNA sequences, as well as other Rfam RNA sequences (see Methods), we obtained 2,147,970, 1,909,629, and 1,932,749 unique sequences from three duplicate samples in C library and 2,047,767, 2,076,708, and 2,237,798 unique sequences from A library (Table 3), and the discovery of mRNA and tRNA indicated that the prepared RNA for sequencing was of good quality. More than ninety percent of the total clean reads had lengths of 20 to 26 nt (Fig. 1), which was consistent with the features of Dicer processing and characteristics of miRNAs^21^.

### Identification of known and potential novel miRNAs

To identify known and potential novel miRNAs in testes of breeder cocks, the dataset was compared with known miRNAs of *Gallus gallus* (chicken) and other organisms (miRNA precursors and mature miRNAs) in miRBase 21.0. Sequences with a perfect match or one mismatch were retained in the known alignment. Sequencing reads that did not match any of the known miRNA were further analyzed to discover potential novel miRNAs. To determine whether these un-annotated small RNA reads were genuine miRNA, their hairpin structures, dicer cleavage sites, and minimal free energies were explored using RNAfold software (http://rna.tbi.univie.ac.at/cgi-bin/RNAfold.cgi/). According to these bioinformatic analyses, we identified 1,305 kinds of miRNAs (Table S1). In order to distinguish these expressed miRNAs into known and potentially novel miRNAs, we carried out advanced bioinformatic analyses and divided the clean reads into five groups, based on these 1,305 miRNAs (Table 4). All miRNAs in group 4 were new novel miRNAs. In total, we identified 1,156 miRNAs; respectively, 839 known miRNAs and 317 new miRNAs were expressed in all testes samples (Table S1).

**Figure 2:**
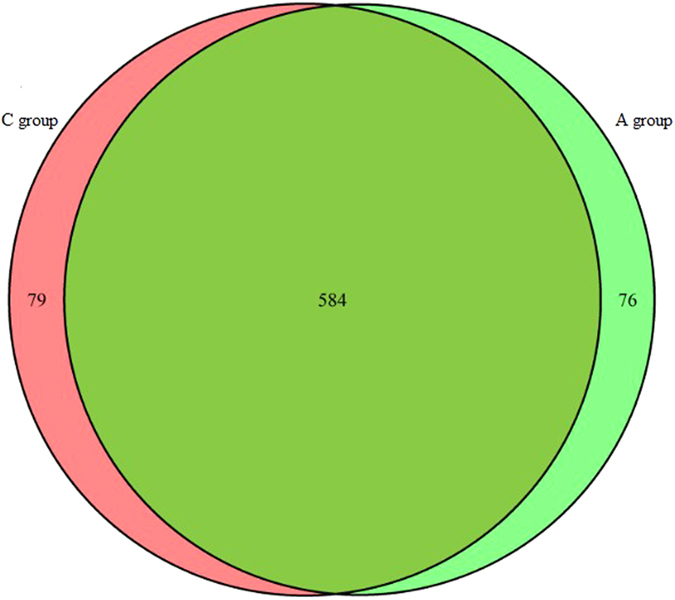
Venn diagrams of miRNAs detected from two different groups (group C VS group A).

**Figure 3:**
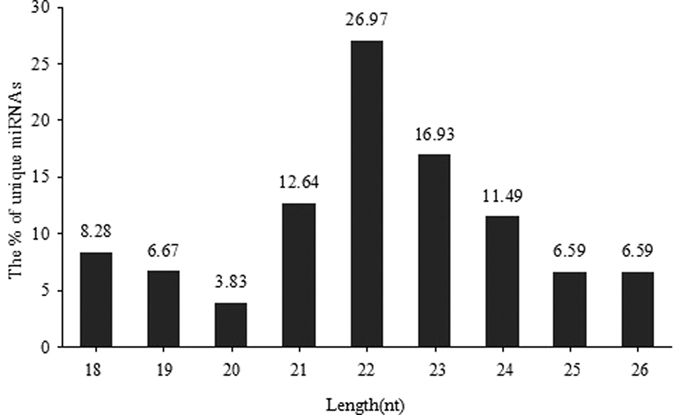
The size distribution of identified miRNAs.

Through co-expression analysis between two groups of libraries, we identified 739 miRNAs and 584 of these were co-expressed in both libraries; 74 miRNAs were detected in the C libraries and 79 were detected in the A libraries (Fig. 2). We then counted the lengths of detected unique miRNAs, approximately 70% of miRNAs were 20 to 24 nt in length and the most abundant class size in the small RNA sequence distribution was 22 nt (Fig. 3). Our results coincided with the definition of miRNAs. Considering the conservation of identified miRNAs among various species, the sequences of existing miRNAs in chickens were aligned and further analyzed to investigate their evolutionary relationships (Figure S1).

### Differential expression of miRNAs induced by dietary APS

**Figure 4:**
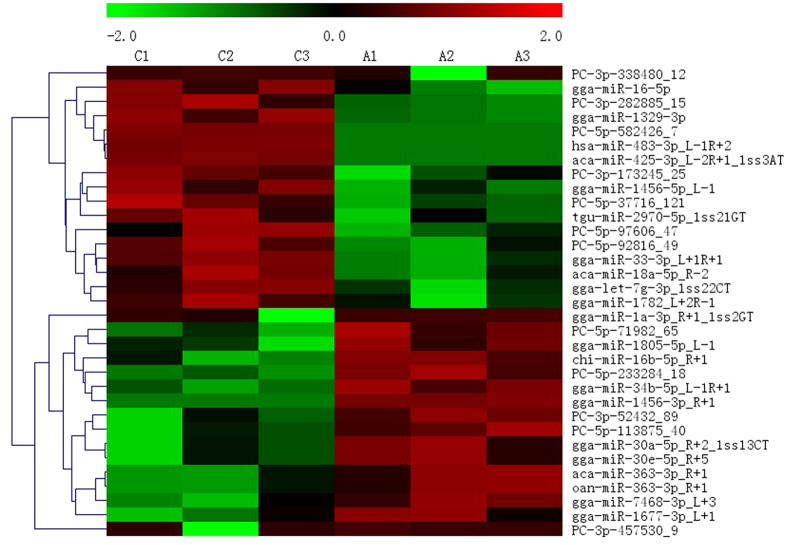
The heat map of differentially expressed miRNAs between C and A libraries. Notes: The unregulated miRNAs are depicted in red color whereas the down-regulated miRNAs are depicted in green color.

Based on the results of Student t test, we gathered 33 differentially expressed miRNAs of testes between control group fed with basal diet (C libraries) and APS group with extra APS supplements (A libraries); of these, 16 miRNAs were up-regulated and 17 miRNAs were down-regulated in the APS group relative to the control group (Fig. 4). These 12 potentially novel miRNAs and 21 known miRNAs could play important roles in the effects of APS on testes. Moreover, to identify conserved miRNAs in breeder cock testicle, the miRNAs in breeder cock testes were compared with testicular miRNAs in cow, goat, and mouse^22^,^23^,^24^. The conserved differentially expressed miRNAs in testes were list in Table S2.

### Validation of differentially expressed miRNAs

**Figure 5:**
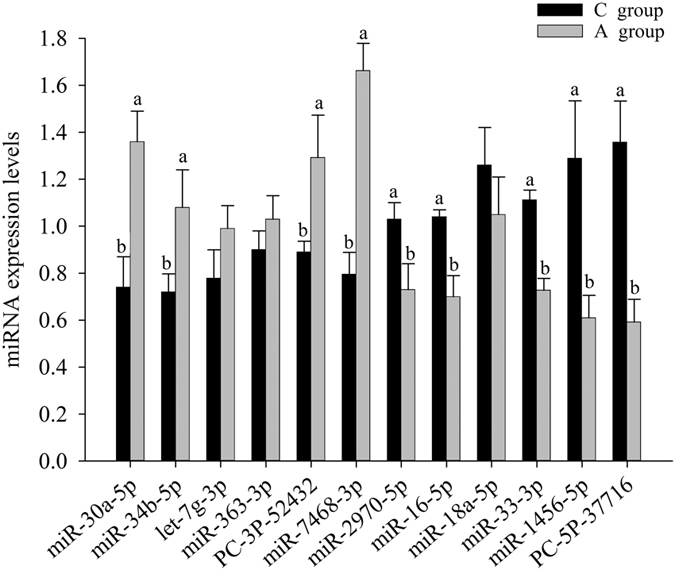
Twelve testes tissue differentially expressed miRNAs, which was validated by reverse-transcription quantitative polymerase chain reaction. Notes: U6 was used as an internal control gene for normalization in our experiments. The data were presented as means ± SE (n = 4). Upper letters (a,b) on bars denote significantly different expression levels in the same miRNAs (*P* < 0.05).

In this experiment, the expression levels of 12 differentially expressed miRNAs representing high, medium, and low expression levels were measured by using the Mir-X miRNA First-Strand Synthesis Kit (Takara Bio Inc., Ohtsu, Japan) according to the manufacturer’s protocol. The results showed that 4 miRNAs had significantly higher expression levels in A group than in C group and 5 miRNAs in A group were significantly lower than C group (Fig. 5), which was consistent with the miRNA-seq data. For another three miRNAs, the different expression trends of aca-miR-363 and aca-miR-18a observed in the miRNA-seq data and in the qRT-PCR were uniform in control and APS groups (Fig. 5), and only the different expression trends of gga-let-7 g observed in the miRNA-seq data and in the qRT-PCR were inconsistent.

### GO enrichment and KEGG pathway analysis of target genes of differentially expressed miRNAs

MiRanda and TargetScan softwares were used to predict target genes of these miRNAs; a total of 3,060 target genes which could be influenced by APS were predicted from the 33 differentially expressed miRNAs (Table S3). Then the predicted target genes were classified according to Gene Ontology (GO) analysis and Kyoto Encyclopedia of Genes and Genomes (KEGG) progress was used to further identify pathways that were actively regulated by miRNAs.

According to the results of GO analysis, among the 3,060 target genes, there were 1,004 differentially expressed genes. By using the biological process for the GO enrichment analysis, 77 differentially expressed target genes were assigned to metabolic process and enzymatic activities (Table 5). Results of the biological process analysis revealed that some target genes of differentially expressed miRNAs could regulate the metabolism process, including nucleotide metabolic process (GO: 0009117), phospholipid biosynthetic process (GO: 0008654), and regulation of proteolysis (GO: 0030162). Meanwhile, we discovered that many biological processes are involved in protein kinase, including transmembrane receptor protein tyrosine kinase signaling pathway (GO: 0007169), positive regulation of protein kinase activity (GO: 0045860), and intracellular protein kinase cascade (GO: 0007243). Additionally, an epigenetic process relating to histone deacetylation was discovered (GO: 0016575).

Moreover, after analysis of cellular component and molecular functions was performed, it was revealed that 770 and 536 differentially expressed target genes were involved in cellular components and molecular functions in the database (Table 5). Analysis based on cellular components and molecular functions showed that differentially expressed miRNAs were involved in sperm motility function, including microtubule motor activity (GO: 0003777), ATP binding (GO: 0005524), oxidoreductase activity (GO: 0016491), and cytosol (GO: 0005829); we also found that molecular functions and biological processes in the epigenetic process included ubiquitin-protein ligase activity (GO: 0004842) and double-stranded RNA binding (GO: 0003725).

**Figure 6:**
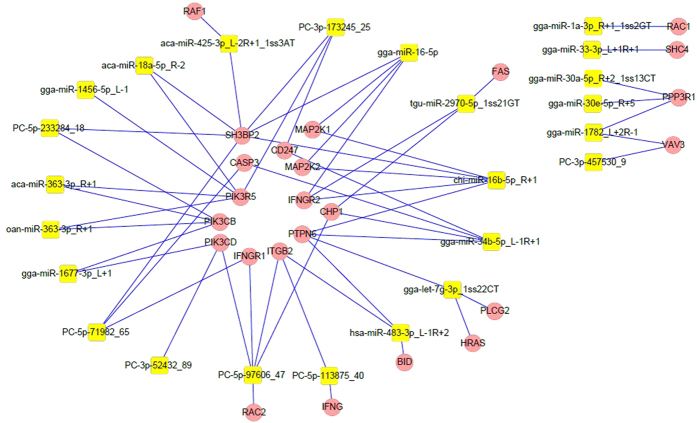
MiRNA-gene network of natural killer cell mediated cytotoxicity pathway (ko04650). Note: pink circular nodes represent genes, and yellow rectangular nodes represent miRNAs. The edges between two nodes represent interactions between genes and miRNAs.

KEGG pathway annotation showed 927 target genes that were annotated for 16 KEGG pathways with P values less than 0.05 (Table 6). KEGG pathway analysis based on predicted targets revealed that differentially expressed miRNAs were involved in several pathways affecting the metabolism of testes, including ribosome biogenesis in eukaryotes (ko03008) and protein processing in endoplasmic reticulum (ko04141) which apply to protein synthesis, amino sugar and nucleotide sugar metabolisms and propanoate metabolism which are involved in propanoate metabolism (ko00640), fatty acid metabolism(ko00071), and one carbon unit metabolisms, including nicotinate and nicotinamide metabolism (ko00760), Cysteine and methionine metabolism (ko00270), were also found in the results of KEGG analysis. Furthermore, the influence of APS on the immune system was also discovered using KEGG analysis; 24 putative target genes of differentially expressed miRNAs enriched natural killer cell mediated cytotoxicity (ko04650) (Fig. 6).

### Expression of NK cell mediated cytotoxicity-associated genes

**Figure 7:**
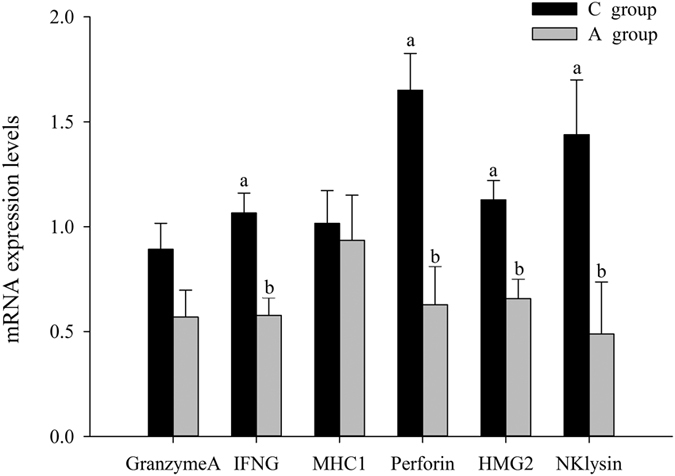
Six NK cell mediated cytotoxicity-associated genes relative expression level. Note: β-actin was used as internal control gene for normalization in our experiments. The data were presented as means ± SE (n = 4). Upper letters (a,b) on bars denote significantly different expression levels in the same miRNA (*P* < 0.05).

In order to validate the effect of dietary APS supplements on NK cell mediated cytotoxicity in breeder cock testes, the expression level of NK cell mediated cytotoxicity-associated genes were detected. In this experiment, compared with control group, the gene expression levels of IFN-γ, perforin, the DNA-binding protein high mobility group-2 (HMG-2), and NK lysin in APS group showed significantly decreased (*P* < 0.05, Fig. 7); whereas, the gene expression levels of Granzyme A and histocompatibility complex class I (MHC I) showed no significant changes.

### gga-miR-16–5p targets the 3′UTRs of MAP2K1, MAP2K2, SH3BP2, and IFNGR2

**Figure 8:**
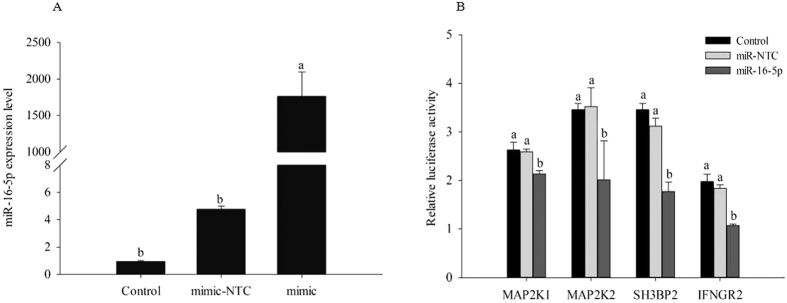
MiR-16–5p mimic or negative control (miR-NTC) and luciferase reporter genes with MAP2K1, MAP2K2, SH3BP2, or IFNGR2 3′UTRs in psiCHECKTM-2 vectors were co-transfected into HEK293T cells. Note: (**A**) The overexpression of miR-16–5p was detected at 48 h in miR-16-5p group after transfection. (**B**) Renilla luciferase activity was normalized to firefly luciferase. All measurements shown are the means ± SE of four independent experiments.

In order to further validate the interaction of gga-miR-16–5p with MAP2K1, MAP2K2, SH3BP2, and IFNGR2 *in vitro*, we constructed luciferase reporter genes with MAP2K1, MAP2K2, SH3BP2, and IFNGR2 3′UTRs in psiCHECK^TM^−2 vectors respectively and then co-transfected the vectors and gga-miR-16–5p mimic or miR-NTC into HEK293T cells respectively. The significant changes of gga-miR-16-5p expression levels in the gga-miR-16-5p groups showed that our co-transfections were successful (Fig. 8A). Furthermore, compared with the control and miR-NTC groups, luciferase reporter assays manifested a significant decrease in luciferase activity (*P* < 0.05) when the MAP2K1, MAP2K2, SH3BP2, and IFNGR2 3′UTR were co-transfected with gga-miR-16–5p mimic respectively (Fig. 8B). These results demonstrated that MAP2K1, MAP2K2, SH3BP2, and IFNGR2 were targeted by gga-miR-16–5p.

### Target gene expression of gga-miR-16-5p related to natural killer cell mediated cytotoxicity pathway

**Figure 9:**
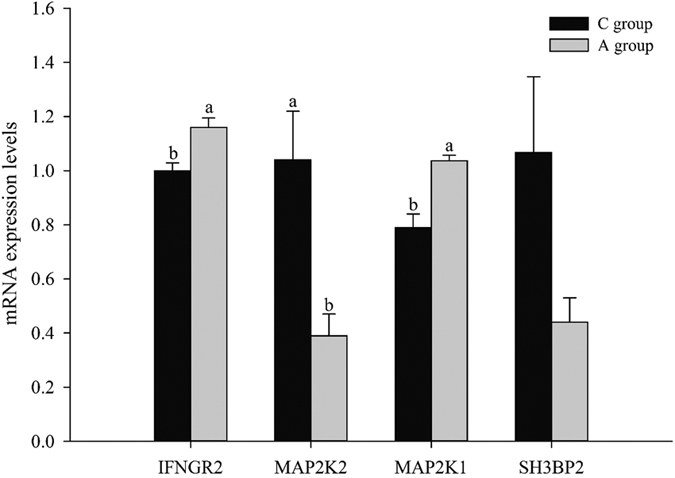
Four target gene expressions of gga-miR-16-5p related to natural killer cell mediated cytotoxicity pathway, validated by reverse-transcription quantitative polymerase chain reaction. Note: β-actin was used as internal control gene for normalization in our experiments. The data were presented as means ± SE (n = 4). Upper letters (a,b) on bars denote significantly different expression levels in the same mRNA (*P* < 0.05).

**Figure 10:**
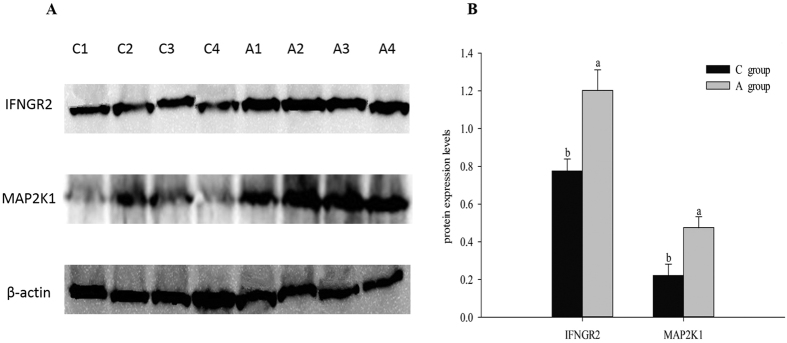
Protein IFNGR2 and MAP2K1 expressions related to dietary APS supplement, validated by western blot. Note: β-actin was used as internal control protein for normalization in our experiments. The data were presented as means ± SE (n = 4). Upper letters (a,b) on bars denote significantly different expression levels in the same protein (*P* < 0.05).

In order to analyze the target gene expression of gga-miR-16-5p in testes and further illuminate the effect of APS in the NK cell mediated cytotoxicity process, we used qRT-PCR and western blot as the standard methods to detect predicted target gene expression of gga-miR-16-5p. In this experiment, when compared with control group, the mRNA expression levels of three of the four target genes in APS group showed significant changes (*P* < 0.05, Fig. 9), including *mitogen-activated protein kinase kinase 2 (MAP2K2*), *interferon gamma receptor 2 (IFNGR2*), and *mitogen-activated protein kinase kinase 1 (MAP2K1*). Moreover, the protein expression levels of these four target genes were measured by western blot; however, only MAP2K1 and IFNGR2 could be detected, and the significant changes of these two protein levels were similar to the mRNA expression levels (*P* < 0.05, Fig. 10). Lacking suitable antibodies for chicken research, although we performed western blot by using the antibodies for humans and mice, we couldn’t detect MAP2K2 and SH3BP2 in western blot.

## Discussion

To date, although previous researches have proved that APS exhibits a distinct suite of immunomodulatory^4^ and metabolic regulation activities^5^, they have ceased to prove whether APS could influence testicular condition of breeder cock. As is known scientifically, the activities of testicular enzymes are important indexes to reflect testicular condition, especially for ACP, AKP, SDH, and LDH^25^. The ACP, which is distributed in the lysosome of testes sertoli cells, could participate in protein synthesis and nutrient intake of testicular cells, as well as provide energy for different periods of sperm cells, including primary spermatocyte and secondary spermatocyte^26^. Besides, the activities of ACP are negatively correlated with the percentage of abnormal sperm while positively correlated with normal testicular function^27^.Simultaneously, SDH and LDH, related to spermatogenesis and sperm maturation, could take part both in aerobic oxidation and anaerobic oxidation of glucose. To be more specific, testicular SDH activity was positively correlated with testicular development and function^28^,^29^. Testicular LDH is mainly secreted by spermatogenic cells and its increased activity could be of paramount importance to successful spermatogenesis^29^,^30^. In the present study, the testicular enzymatic activities of ACP, SDH, and LDH in APS group were significantly increased compared with the control group, which represented an improvement in testicular functions. Meanwhile, when APS supplements were added to breeder cocks’ diet in APS group, LDH increased, energized^31^, and finally elevated sperm enzymatic activities, which improved the testicular functions. Furthermore, GSH-px and T-SOD were significantly increased (P = 0.006, P = 0.002) while MDA were not notably changed, which again represented the improvement of both testicular antioxidant abilities and functions^32^.

In recent years, several miRNAs have been identified in different parts of chickens, for example, in breast muscles, ovaries, abdominal adipose, and skeletal muscles^33^,^34^,^35^,^36^. However, neither the miRNAs expression profiles of breeder cocks testes nor their expression responses to dietary APS intake have yet been reported. Thanks to the next generation sequencing which provided a new insight and evidence for the complexity of the chicken’s transcriptome and miRNAomics^37^, we could not only obtain more information about the potential effect of APS on testes but also it provided a novel insight into the roles of miRNAs on the interactions between dietary APS supplementations and breeder cock testes at the same time. Based on the effect of APS on testicular and spermatozoal enzymatic changes, differentially expressed miRNAs that induced by dietary APS were further identified by miRNA sequencing to further investigate the potential course of testicular condition and enzymatic changes.

According to an overview of miRNA expression profiles in testes tissues of breeder cocks, we found that more than 90% of the small RNA sequences were primarily distributed in the 20–26 nt range in both libraries (Fig. 1). This was not fully consistent with the typical size of mature mammalian miRNA, the lengths of which were mainly distributed in 20–24 nt^38^. Our results showed that the lengths of chicken miRNA in testes were mainly distributed in 22–26 nt, and all of the six testes samples had good consistency. Therefore, it indicated that small RNA length distributions of testes in breeder cocks may follow the distribution pattern discovered in our research. In the RNA that we found in testes samples, it could be possible that piRNA had lengths of 25–33nt^39^,^40^, which allowed us to determine the special length distribution in testes. Furthermore, the proportion of total rRNA was used for a quality check of the samples. The proportion of total rRNA in our samples was from 1.40% to 2.74% in all libraries compared to a normal range of less than 40% in high quality animal samples^21^ (Table 3), which indicated that the collected endometrium samples were of high quality. We aligned the clean reads to the miRNAs of all known animals in the miRBase 21.0 database. A total of 739 miRNAs were identified, 584 of which were co-expressed in both libraries; 79 miRNAs were detected in the R library and 76 in the P library. It was determined that these 584 co-expressed miRNAs played an important role in testes functions. In addition, according to identification of known and potential novel miRNAs, we found that the size distribution of identified miRNAs was consistent with the typical size of mature miRNAs^38^, indicating that follow-up studies should be based on mature miRNAs.

In the present study, the miRNA profiles in the testes tissues with or without dietary were obtained using next-generation sequencing and we identified 33 differentially expressed miRNAs between the APS and control libraries. Meanwhile, qRT-PCR were used to detect and validate the expression levels of miRNAs; in all twelve miRNAs except gga-let-7 g, the different expression trends observed in the miRNA-seq data and in the qRT-PCR were uniform in the control group and APS group, suggesting that the miRNA expression profiles could represent actual miRNA expression levels. As for gga-let-7 g, the differential expression trends observed in qRT-PCR and miRNA sequencing were not uniform. This inconsistency might have resulted from differences in the two methods^12^. The expression levels of miRNAs measured by Solexa deep sequencing was accurate^37^, however, in the process of qRT-PCR we used, RNA molecules were polyadenylated and reverse transcribed using polyA polymerase. The gga-let-7 g consisted of a very low quantity (26 in control groups and 19 in APS groups), and the miRNAs in let-7 family were highly similar to each other despite that sometimes there is only one nucleotide differences. Therefore, the qRT-PCR we used may not have been sensitive enough to detect the differences of let-7 g between Control and APS groups.

Among these 33 differentially expressed miRNAs, 13 were known miRNAs of *Gallus gallus* in miRBase 21.0, and 3 of these miRNAs were of high expression and among which the gga-miR-30a was the highest differentially expressed one. Several studies have demonstrated that miR-30a could play an important role in testicular development of pigs^16^ and the expression of miR-30a in mature testes was nearly doubled the amount in immature testes. When patients from control group were compared with patients with non-obstructive azoospermia, clinical research results showed that miR-30a were down-regulated in patients with non-obstructive azoospermia^41^. All these researches have proven that an increase of miR-30a expression is beneficial for testes development, and our research have proven that APS supplements could increase the miR-30a expression in testes. The gga-miR-16 was found to be the second highest differentially expressed miRNA in testes of breeder cocks and had also been detected in testes of many other species, including mice, humans, and monkeys^39^,^42^,^43^. The gga-miR-34b was found to be the third most highly expressed miRNA, and researches on mice and pigs have also revealed the influence of miR-34b on testicular development^15^,^16^. Additionally, aca-miR-363 and aca-miR-18a were also found to be high differentially expresser miRNAs in testes of breeder cocks, as well as in other species^44^,^45^. In addition, compared with the miRNA expression profiles from testes of goat, cow, and mouse^22^,^23^,^24^, we found 13 miRNAs were conserved in testes, which means that these 13 differentially expressed miRNAs could play crucial roles in testicular developments or their functions and the APS could regulate testicular condition by inducing the differential expression of miRNAs. Although we analyzed the function of differentially expressed miRNAs between APS and control libraries in our research and inferred that APS could influence testicular development or functions by regulating miRNAs, further researches need to be conducted to ascertain our findings.

In order to illuminate the effect of differentially expressed miRNAs on testes, we performed the GO and KEGG analyses and found that differentially expressed miRNAs regulated many metabolism processes in testes, for example, the nucleotide metabolic process (GO: 0009117), regulation of proteolysis (GO: 0030162), propanoate metabolism (ko00640), fatty acid metabolism (ko00071) and phospholipid biosynthetic process (GO: 0008654), one carbon unit metabolisms including nicotinate and nicotinamide metabolism (ko00760), cysteine and methionine metabolism (ko00270), and other metabolic pathways (ko01100). Combined with the results of GO and KEGG analysis, we found that dietary APS supplements could influence glucose, lipid, protein, as well as nucleotide metabolism. These metabolic processes might be correlated with the function changes of testes we discovered in Table 1. the increased activities of LDH and SDH may be influenced by some miRNAs (Table S4 and Table S5) involved in some metabolic pathways that related to energy supply, such as propanoate metabolism (ko00640), fatty acid metabolism(ko00071), phospholipid biosynthetic process (GO: 0008654), nicotinate and nicotinamide metabolism (ko00760), and Cysteine and methionine metabolism (ko00270). Moreover, the ACP, which may participate in protein synthesis and nutrient intake of testicular cell^26^, could be influenced by some miRNAs (Table S4 and Table S5) involved in endoplasmic reticulum (GO: 0005783), endoplasmic reticulum-Golgi intermediate compartment (GO: 0005793), protein processing in endoplasmic reticulum (ko04141), and ribosome biogenesis in eukaryotes (ko03008). Additionally, previous studies about APS functions have verified our findings. With the injection of lipopolysaccharide, Liu Lei *et al*.^46^ believed that APS was able to suppress the expression of pro-inflammatory cytokines and improve energy and protein metabolism. Recent studies have proved that APS could increase liver glycogen synthesis and skeletal muscle glucose translocation^47^, enabling insulin-sensitizing and hypoglycemic activity at least in part by enhancing the adaptive capacity of hepatic endoplasmic reticulum^48^, which can further promote insulin signal transduction and alter myocardial glucose and lipid metabolism patterns^5^. Considering the amino acid metabolism, F. G. Yin *et al*.^49^ discovered that APS could ameliorate amino acid metabolism to beneficially increase the entry of dietary amino acids into the systemic circulation. Therefore, APS could easily regulate nutritional metabolism and further improve testicular functions. Additionally, the increase of GSH-px and T-SOD activities also could be regulated by miRNAs (Table S4) involved in oxidoreductase activity (GO: 0016491), which related to dietary APS supplements. In conclusion, APS supplements could regulate testicular metabolism and further improve testicular condition by changing the different miRNA expressions. Meanwhile, we found that many biological processes in GO analysis were involved in protein kinase, which include transmembrane receptor protein tyrosine kinase signaling pathway (GO: 0007169), positive regulation of protein kinase activity (GO: 0045860), and intracellular protein kinase cascade (GO: 0007243). All of these biological processes were typically characteristic of one famous cell signaling pathway called RTK-Ras-MAPK signaling pathway^50^. Previous researches about the effect of APS have proven that MAPK signaling pathway played an important role in regulating glycometabolism^47^,^51^. According to these analyses between testicular enzymatic activities and the function of differentially expressed miRNA, APS could play an important role in regulating testicular metabolism and further testicular development and condition by investigating the miRNA differential expressions.

Furthermore, more researches are focusing on APS immunoregulation. In our studies, we discovered a pathway called natural killer cell mediated cytotoxicity, which is associated with testes innate immunity and induced by dietary APS supplements. As testes are typical tissues with immune exemptions, systemic immune responses are inhibited; however, some special cells in testes contribute to the foundation of innate immunity^52^,^53^. NK cells are innate lymphoid cells (ILCs) widely renowned for their role in eliminating transformed and virus-infected cells and the NK cell mediated cytotoxicity is independent of antibodies^54^. Therefore, NK cell mediated cytotoxicity may contribute to regulating APS regarding innate immunity in testes. Moreover, we also tested some gene expression levels associated with the NK cell mediated cytotoxicity pathway, including the Granzyme A, MHC I, IFN-γ, perforin, HMG-2, and NK lysine^55^. Compared with control group, the gene expression levels of IFN-γ, perforin, HMG-2, and NK lysin in APS group showed a significant decrease (*P* < 0.05, Fig. 7). This represented that APS supplements could decrease NK cell mediated cytotoxicity in testes and improve testicular health condition, which are the foundation of good testicular function.

In order to illuminate the function of miRNAs in regulating the process of NK cell mediated cytotoxicity, we made an analysis of miRNA-gene networks (Fig. 6), and found that 24 differentially expressed miRNAs were involved in NK cell mediated cytotoxicity. According to the results of our previous analysis, we found that gga-miR-16–5p plays an important role in all pathways except selenocompound metabolism (ko00450) of KEGG analysis. Besides, the gga-miR-16-5p was the only high differentially expressed miRNA that could regulate several target genes involved in the NK cell mediated cytotoxicity pathway. Subsequently, we chose gga-miR-16-5p as our further research project.

According to the validation of target gene expression of gga-miR-16-5p, we found that APS could regulate four target genes expressions which were involved in NK cell mediated cytotoxicity. Additionally, dual luciferase reporter system was used to illuminate the interaction between gga-miR-16-5p and the 3′UTR of IFNGR2, SH3BP2, MAP2K2, and MAP2K1. The luciferase assay results confirmed that gga-miR-16-5p repressed potential target genes (IFNGR2, SH3BP2, MAP2K2, and MAP2K1) expression by interacting with their 3′UTR. There was compelling evidence both that miRNAs repress target translation and that they trigger target degradation^56^,^57^, and all these regulatory mechanisms could result in the negative correlation between the expression of miRNAs and target genes, which was consistent with our dual luciferase assay results.

Further validation of these four target gene expressions by q-PCR and western blot proved that the expressions of IFNGR2 and MAP2K1 decreased (Fig. 9). As scientifically known, miRNAs are hairpin-derived RNAs with imperfect complementarity to target genes, causing decreased target gene expression^58^. Thus IFNGR2 and MAP2K1 are potential target genes of gga-miR-16-5p (Fig. 9). In previous studies, MAP2Ks and IFNGR2 have been proven to participate in regulating NK cell cytotoxic functions^59^,^60^; therefore, gga-miR-16-5p may have been involved in regulating NK cell mediated cytotoxicity by taking IFNGR2 and MAP2K1 as target genes. As for SH3BP2 and MAP2K2, their mRNA expression levels were increased, which were not consistent with the change of gga-miR-16-5p. However, the mature miRNA is incorporated into an RNA-induced silencing complex that binds to a target mRNA and further influences the translation of mRNA^61^,^62^; therefore, gga-miR-16 might influence the protein expression levels of SH3BP2 and MAP2K2. Since we didn’t find suitable antibodies for these two protein, it’s hard for us to certify whether these two protein expression could be regulated by gga-miR-16 on translation process or not. Besides, a miRNA could regulate many gene expressions, and a gene expression could also be regulated by many miRNAs^63^,^64^, which are important components of “competing endogenous RNA (ceRNA)” mechanism^65^. In our experiment, SH3BP2 and MAP2K2 might not be regulated by gga-miR-16-5p, but might be regulated by chi-miR-16b-5p, whose expression change was consistent with these genes. Meanwhile, the actual activity and effect of gga-miR-16-5p on these two genes might be influenced by lncRNAs, other mRNAs and miRNAs (such as chi-miR-16b-5p) that contain the same miRNA response elements (MREs)^66^. According to the validation of differentially expressed miRNAs and target gene expressions of gga-miR-16-5p both *in vivo* and *in vitro*, we believe that APS could change gga-miR-16-5p expression and its target gene (IFNGR2 and MAP2K1) expressions and further regulate the natural killer cell mediated cytotoxicity pathway in testes of breeder cocks.

In conclusion, we found that dietary APS supplements could regulate enzymatic activities related to testicular functions and we successfully obtained high-quality miRNA expression profiles from testes tissues of breeder cocks fed with or without APS and predicted 699 known and 252 novel miRNAs in testes of breeders for the first time. Our researches also suggested that target gene predictions for the 33 differentially expressed miRNAs, functional annotations, and pathway analyses in GO and KEGG databases could contribute to a better understanding of the miRNA mediated regulations of target genes in response to dietary APS supplements. According to our results, APS could regulate the miRNAs expression related to metabolism of testes, including glucose, lipid, protein, and nucleotide metabolism process. Furthermore, we found that APS could regulate the innate immunity of testes by natural killer cell mediated cytotoxicity pathway. Meanwhile, the changed expression of gga-miR-16-5p and its target genes such as IFNGR2 and MAP2K1 could play an important role in the regulation of natural killer cell mediated cytotoxicity pathway.

## Materials and Methods

### Ethics statement

This study was conducted in strict accordance with the Regulations for the Administration of Affairs Concerning Experimental Animals (Ministry of Science and Technology, China, revised 2004). All experimental protocols in the study were approved by the Institutional Animal Care and Use Committee (IACUC) of the College of Animal Science and Technology of the Northwest A&F University (Yangling, Shaanxi, China), and the birds were managed in accordance with recommendations established by the Ministry of Agriculture, P. R. China. In addition, all surgeries was performed under sodium pentobarbital anesthesia and all efforts were made to minimize suffering.

### Animal feedings and sample collections

Based on a single factor experimental design, a total of 64 one-day-old Cobb500 breeder cocks were randomly assigned to two groups with four replicates per treatment and 8 birds per replicate, including two treatments: control and dietary APS supplements (10 g/kg) groups. The chickens in control group (C group) were fed with corn soybean diet, and 10 g APS per kilogram fodder were added to the feed of APS group (A group). All chickens were kept in an environmentally controlled henhouse with double-floor metabolism cages and exposed to a 16 h photoperiod. Water was available ad libitum and food was available according to the feeding standard of Cobb500 breeder cocks.

At forty weeks of age, one bird was selected randomly from each replicate and the semen sample from these 8 breeder cocks were collected and frozen immediately in liquid nitrogen. Then these 8 birds were killed and rapidly dissected. The left sides of the testes without blood contamination were collected into 2 ml Eppendorf tubes and frozen immediately in liquid nitrogen. All samples were stored at −80 °C until analyzed. Six testes samples from three replicates were selected randomly for next generation sequencing analysis of miRNAs.

### Biochemical assay

The enzymatic activities of ACP, AKP, SDH, and LDH were determined with a spectrophotometric method according to the manufacturer’s procedure (Jiancheng Biological Engineering Research Institute, Nanjing, China). Respectively, the rate of pyruvate generated from lactate, and the reduction rate from FAD to FADH could represent the LDH and SDH activities^67^,^68^. Moreover, the Kings methods by detecting the decomposition rate of disodium phenyl phosphate were used to measure the ACP and AKP activities^67^,^68^.

The activities of GSH-Px and SOD, as well as the MDA concentration of testes samples, were measured by a spectrophotometric method according to the methods previously described in detail^69^. Moreover, the testosterone content of testes sample were measured by ELISA kits (Jiancheng Biological Engineering Research Institute, Nanjing, China) for chicken following the methods previously described^70^.

### Small RNA library construction and sequencing

Total RNA from testes for miRNAs sequencing was extracted using Trizol reagent (Invitrogen, CA, USA) according to the manufacturer’s procedure. The quantity and purity of total RNA were analyzed by Bioanalyzer 2100 and RNA 6000 Nano LabChip Kit (Agilent, CA, USA) with RIN number >7.0. From each sample we gathered, approximately 1 μg of total RNA was used to prepare a small RNA library according to protocol of TruSeq Small RNA Sample Prep Kits (Illumina, San Diego, USA).

Following the recommended protocol for each treatment, 3 biological replicates were prepared; then, we performed the single-end sequencing (1*50bp) on an Illumina Hiseq2500 at the LC-BIO (Hangzhou, China).

We processed our data according to procedures described in a previous study^71^ by LC Sciences Service. The raw reads were subjected to the Illumina pipeline filter (Solexa 0.3); then, the dataset was further processed with an in-house program named ACGT101-miR (LC Sciences, Houston, Texas, USA) to remove adapter dimers, junk, low complexity, common RNA families (rRNA, tRNA, snRNA and snoRNA) and repeats. The sequence data from this study have been deposited at Gene Expression Omnibus (GEO) and can be accessed with the number GSE79479.

### Identification of known miRNAs and potential novel miRNAs

Unique sequences with lengths from 18 to 26 nt were mapped to specific species precursors in miRBase21.0 (ftp://mirbase.org/pub/mirbase/CURRENT/) by BLAST search to identify known miRNAs and novel 3p- and 5p- derived miRNAs. Length variations at both 3 ft. and 5 ft. ends and one mismatch inside the sequence were allowed in the alignment. The unique sequences mapping to specific species mature miRNAs in hairpin arms were identified as known miRNAs. The unique sequences mapping to the other arm of known specific species precursor hairpin opposite the annotated mature miRNA-containing arm were considered to be novel 5p- or 3p derived miRNA candidates. The remaining sequences were mapped to other selected species precursors (with the exclusion of specific species) in miRBase 21.0 by BLAST search and mapped pre-miRNAs were further BLASTed against the specific species genomes to determine their genomic locations. We defined the above two as known miRNAs.

The unmapped sequences were BLASTed against the specific genomes and the hairpin RNA structures containing sequences were predicated from the flank at 80 nt sequences using RNAfold software (http://rna.tbi.univie.ac.at/cgi-bin/RNAfold.cgi/). The criteria for secondary structure prediction followed the procedures described in a previous study^72^,^35^: (1) number of nucleotides in one bulge in stem (< = 12); (2) number of base pairs in the stem region of the predicted hairpin (> = 16); (3) cutoff free energy (kCal/mol < = 15); (4) length of hairpin (up and down stems +terminal loop > = 50); (5) length of hairpin loop (< = 20); (6) number of nucleotides in one bulge in mature region (<8); (7) number of biased errors in one bulge in mature region (< = 4); (8) number of biased bulges in mature region (< = 2); (9) number of errors in mature region (< = 7); (10) number of base pairs in the mature region of the predicted hairpin (> = 12); (11) percent of mature in stem (> = 80).

### Analysis of differentially expressed miRNAs

To compare differentially expressed miRNAs of testes between control group fed with basal diet (C libraries) and APS group with extra APS supplements (A libraries), we normalized the expression abundances of miRNAs in the six samples from two treatments; the formula was: Normalized Expression (NE) = Actual miRNA count/Total count of clean reads. When the NE of a certain miRNA was 0 in one library, we revised the 0 to 0.001 for the comparative and the miRNAs whose NE was less than 1 in both libraries were discarded, as previously described in detail^73^.

The differential expression of miRNAs based on normalized counts was analyzed using Student t test according to the experimental design and the significance threshold was set as 0.05 for each test^74^,^75^,^76^,^77^. The fold-change and P-value for each miRNA was calculated, based on the normalized expression using the formula as previously described^12^,^73^.

### GO enrichment and KEGG pathway analysis of target genes

To predict the genes targeted by differentially expressed miRNAs, computational target prediction algorithms (TargetScan 50 and miRanda 3.3a) were used to identify miRNA binding sites. Finally, the data predicted by both algorithms were combined and the overlaps were calculated. Functional annotation of predicted miRNA targets were performed based on GO database and enriched pathways were analyzed using Kyoto Encyclopedia of Genes and Genomes database KEGG^12^. The significantly enriched functional categories were determined by the multiple test adjustment proposed by Benjamini & Hochberg. KEGG pathway enrichment analysis and visualization were performed using R packages: GOstats and pathview. The KEGG pathways with a *P* < 0.05 were defined as significantly enriched pathways. Additionally, the miRNA-pathway interaction network was constructed using Cytoscape v2.8.3 software (http://www.cytoscape.org/).

### Real-time quantitative PCR

To validate the differentially expressed miRNAs in our present manuscript, 12 differentially expressed miRNAs representing high, medium, and low expression levels according to the sequencing results were selected randomly for further qRT-PCR validation. Total RNA was extracted using Trizol reagent (TaKaRa, Dalian, China) following the manufacturer’s instructions; the total RNA was quantified using a NanoDrop^®^ ND-1000 spectrophotometer (Thermo Scientific) with the OD value set at 260 nm; the purity was assessed by determining the OD260/OD280 ratio using formaldehyde-agarose gel electrophoresis. About 1 μg of total RNA was reverse transcribed using the Mir-X miRNA First-Strand Synthesis Kit (Takara Bio Inc., Ohtsu, Japan) according to the manufacturer’s protocol. By using this method, RNA molecules were polyadenylated and reverse transcribed using polyA polymerase. The reverse transcriptase in the Synthesis Kit and all miRNAs were reverse transcribed at one time. The miRNA quantification was performed with iCycler IQTM5 (Bio-Rad Laboratories, Hercules, CA, USA) using SYBR Advantage qPCR Premix (Takara Bio Inc.). The reaction mixtures were a combination of the following reagents: cDNA, 2 μL; 2 × SYBR Advantage Premix, 12.5 μL; mRQ 3′ Primer (supplied with the Mir-X miRNA First-Strand Synthesis Kit), 0.5 μL; miRNA-specific primer, 0.5 μL; ddH20, 9 μL. The reaction mixtures were denaturation at 95 °C for 10 s, followed by 40 cycles of 95 °C for 5 s and 60 °C for 20 s. The U6 was used as an internal normalization control. The specific primers are shown in Table S6.

Furthermore, we detected the relative transcript level of some NK cell cytotoxicity-associated genes (IFN-γ, GranzymeA, MHC I, perforin, HMG2, and NK-lysin) and the target genes (MAP2K2, IFNGR2, SH3BP2 and MAP2K1) of gga-miR-16-5p in testes of breeder cocks by using real-time qPCR. The cDNA was synthesized with a Prime Script^®^ RT reagent Kit (Takara) according to the manufacturer’s protocols. The expression levels of four target genes of gga-miR-16-5p were determined using SYBR^®^ Premix Ex Taq^TM^ II (Takara). A 25-μL PCR mixture was quickly prepared from 12.5 μL of SYBR^®^ Premix ExTaq II (2 × ), 1 μL of forward primer (10 μM/L), 1 μL of reverse primer (10 μM/L), 1 μL of cDNA, and 9.5 μL of double-distilled water. Primers for real-time PCR were synthesized by Sangon Biotech and are also listed in Table S6. The PCR with amplifications was conducted in an iCycler iQ5 multicolor real-time PCR detection system (Bio-Rad Laboratories) and programmed as follows: 95 °C for 10 min; 40 cycles of 95 °C for 10 s; 60 °C for 30 s; 72 °C for 30 s; and 72 °C for 5 min. All samples were examined in triplicate. All data were analyzed using the 2−ΔΔCt method^78^.

### 3′UTR luciferase reporter assays

The 3′UTR of MAP2K1, MAP2K2, SH3BP2 or IFNGR2 mRNA, containing the binding site of gga-miR-16–5p, were amplified from total RNA extracted from breeder cocks’ testes by RT-PCR and further PCR with primers shown in Table S6. All constructs were further confirmed by sequencing. The fragments were cloned into psiCHECKTM-2 Vectors (Promega, Madison, WI, USA) at the 3′-end of the Renilla gene using XhoI and NotI restriction sites.

Luciferase reporter experiments were performed in human embryonic kidney (HEK) 293 T cells. More than 2.0 × 10^4^ HEK293T cells were seeded in 24-well plates and cultured in high glucose Dulbecco’s modified Eagle’s medium (DMEM) with 10% fetal bovine serum. 24 hours later, 500 ng of vector constructs were co-transfected with either 50 nM of gga-miR-16-5p mimic or miR-NTC using 2.0 μL X-tremeGENE siRNA Transfection Reagent (Roche, USA), and the cells in control group were without any treatment. The expression level of gga-miR-16-5p from cells in three groups were measured by qRT-PCR; the methods and data process were described as before. Luciferase activity was measured at 48 h after transfection by the DualGlo Luciferase Assay System (Promega). Renill luciferase activity was measured and normalized to corresponding firefly luciferase activity.

### Western blot experiment

Testes samples were homogenized in ice-cold protein lysis buffer (RIPA, Beyotime, Shanghai, China) containing protease and phosphorylase inhibitor. The lysates were centrifuged to remove tissue and cell debris. Proteins were boiled in 4 × protein loading sample buffer (Solarbio, Beijing, China) for 10 min. A total of 20 mg of protein were electrophoresed on a 12% SDS-polyacrylamide gel and transferred to 0.45 μm PVDF (polyvinyldene difluoride) membranes (Merck Millipore, USA). The membrane was blocked in 5% skim milk, and then incubated at 4 °C overnight with primary antibodies. Antibodies against MAP2K2, IFNGR2, SH3BP2, MAP2K1, and β-actin (Abcam, Cambridge, UK) were used. The results were visualized with horseradish peroxidase-conjugated secondary antibodies (CWBIO, Beijing, China) and enhanced chemiluminescence. The protein bands were quantified using the Image J program (National Institutes of Health, Bethesda, MD).

### Statistical analysis

The statistical evaluation of experimental results, including results of biochemical assay, real-time quantitative PCR, 3′UTR luciferase reporter assays, and western blot experiment, were analyzed by student’s T test using SPSS 20.0 statistical software. All data were expressed as means with standard error (SE). Differences were considered to be statistically significant at *P* < 0.05.

## Additional Information

**Accession codes:** The sequence data from this study have been deposited at Gene Expression Omnibus (GEO) and can be accessed with the number GSE79479.

**How to cite this article**: Wu, S. *et al*. Effect of dietary Astragalus Polysaccharide supplements on testicular miRNA expression profiles and enzymatic changes of breeder cocks. *Sci. Rep.* **7**, 38864; doi: 10.1038/srep38864 (2017).

**Publisher's note:** Springer Nature remains neutral with regard to jurisdictional claims in published maps and institutional affiliations.

## Supplementary Material

Supplementary Information

## Figures and Tables

**Table 1:** Effect of dietary APS supplements on the enzymatic activities of breeder cocks testes.

Item	Control	APS	*P*value
ACP (King unit/mgprot)	2629.36 ± 35.08	2897.93 ± 18.49	0.001
AKP (King unit/mgprot)	494.02 ± 69.77	520.25 ± 74.27	0.805
SDH (U/mgprot)	77.14 ± 3.79	92.95 ± 1.05	0.007
LDH (U/mgprot)	1555.02 ± 61.77	1824.75 ± 12.44	0.020
Testosterone (nmol/mgprot)	4.87 ± 0.20	5.29 ± 0.28	0.266
MDA (nmol/mgprot)	51.56 ± 4.85	45.74 ± 3.28	0.358
T-SOD (U/mgprot)	116.06 ± 2.62	134.48 ± 3.59	0.006
GSH-px (U/mgprot)	256.38 ± 11.62	386.68 ± 22.17	0.002

Note: The data were presented as means ± SE (n = 4). Differences were considered to be statistically significant at *P* < 0.05.

**Table 2:** Effect of dietary APS supplements on the enzymatic activities of breeder cocks sperm.

Item	Control	APS	P value
ACP (King unit/mgprot)	7084.32 ± 137.45	7115.55 ± 361.65	0.938
AKP (King unit/mgprot)	393.98 ± 25.60	437.46 ± 25.46	0.274
SDH (U/mgprot)	119.72 ± 13.57	92.33 ± 9.60	0.151
LDH (U/mgprot)	534.44 ± 54.06	817.83 ± 11.24	0.002

Note: The data were presented as means ± SE (n = 4). Differences were considered to be statistically significant at *P* < 0.05.

**Table 3:** Summary of raw data reads to cleaned sequences for small RNAs in testes from breeder cocks.

lib	Group C						Group A					
	C1		C2		C3		A1		A2		A3	
	Total	uniq	Total	uniq	Total	uniq	Total	uniq	Total	uniq	Total	uniq
Raw reads	13,973,000	5,244,082	10,731,609	4,546,673	11,606,715	4,641,106	13,429,114	5,296,244	12,441,763	5,175,695	13,968,612	5,639,991
3ADT&length filter	6,634,798	2,583,368	4,098,829	2,186,594	4,807,423	2,262,696	5,541,024	2,702,673	5,048,328	2,598,685	5,565,900	2,836,613
Junk reads	17,148	11,761	13,239	9,476	13,445	9,511	16,865	11,630	15,506	11,030	17,900	12,719
Rfam	383,026	37,110	236,408	28,731	248,915	28,577	517,420	41,547	337,071	33,583	480,930	41,338
mRNA	1,176,749	468,100	1,105,020	415,320	1,162,131	410,714	1,279,612	497,207	1,198,488	459,526	1,381,133	516,122
Repeats	44,827	5,172	24,423	3,707	24,764	3,709	46,595	4,424	33,441	4,074	44,222	4,942
valid reads	5,775,619	2,147,970	5,288,916	1,909,629	5,384,254	1,932,749	6,095,003	2,047,767	5,856,291	2,076,708	6,541,779	2,237,798
rRNA	245,739	15,816	150,401	13,200	165,510	13,192	368,061	19,076	233,048	15,353	331,706	18,142
tRNA	52,108	7,375	32,054	5,030	28,080	4,791	56,103	7,891	40,753	6,126	58,604	8,005
snoRNA	15,393	3,475	13,425	2,653	11,817	2,622	15,779	3,918	12,344	3,034	17,174	3,794
snRNA	10,771	3,677	6,935	2,792	7,097	2,793	18,207	4,391	10,420	3,426	15,947	4,488
other Rfam RNA	59,015	6,767	33,593	5,056	36,411	5,179	59,270	6,271	40,506	5,644	57,499	6,909

Notes: (1) ADT & length filter: reads without 3′ Adaptor and with length of <18 nt or >26 nt were removed (for animals). (2) Junk reads: > = 2 N number of Ns (discontinued) in a read that will be filtered out (> = 2). > = 7 A number of consecutive A (adenine) in a read that will be filtered out (> = 7). > = 8 C number of consecutive C (cytosine) in a read that will be filtered out (> = 8). > = 6 G number of consecutive G (guanylic acid) in a read that will be filtered out (> = 6). > = 7 T number of consecutive T (thymine) in a read that will be filtered out (> = 7). > = 10Dimer number of dimer repeats in a read that will be filtered out (> = 10). > = 6Trimer number of trimer repeats in a read that will be filtered out (> = 6). > = 5Tetramer number of tetramer repeats in a read that will be filtered out (> = 5).

(3) Rfam: Collection of many common non-coding RNA families except micro RNA (http://rfam.janelia.org), including rRNA, snoRNA, snRNA, tRNA.

(4) Repeats: Prototypic sequences representing repetitive DNA from different eukaryotic species (http://www.girinst.org/repbase). There are redundant sequences between mRNA, Rfam and Repeats.

**Table 4:** Summary of known and predicted miRNA.

			C library						A library					
Total			C1		C2		C3		A1		A2		A3	
Criteria	Pre-miRNA	Unique miRNA	Pre-miRNA	Unique miRNA	Pre-miRNA	Unique miRNA	Pre-miRNA	Unique miRNA	Pre-miRNA	Unique miRNA	Pre-miRNA	Unique miRNA	Pre-miRNA	Unique miRNA
gp1a	326	434	228	294	231	300	208	271	229	300	217	288	233	308
gp1b	2	3	1	1	1	2	2	2	1	1	2	2	1	1
gp2	38	38	31	31	24	25	26	26	23	25	27	28	25	26
gp3	371	364	169	161	274	257	161	149	152	143	154	146	215	200
gp4	252	317	225	285	213	270	216	272	207	261	215	274	211	265

(1) gp1a: Reads map to *Gallus gallus* pre-miRNAs in miRbase and the pre-miRNAs further map to the genome & EST. (2) gp1b: Reads map to other vertebrata pre-miRNAs in miRbase we selected and the pre-miRNAs further map to the genome & EST. (3) gp2: Reads map to all vertebrata pre-miRNAs we selected in miRbase. The mapped pre-miRNAs do not map to the genome; however, the reads (and of course the miRNAs) map to genome. The extended genome sequences from the genome loci may form hairpins. (4) gp3: Reads map to all vertebrata pre-miRNAs in miRbase we selected. Both the mapped pre-miRNAs and the reads do not map to the genome. (5) gp4: Reads do not map to all of the vertebrata pre-miRNAs in miRbase we selected. However, the reads map to genome & the extended genome sequences from genome may form hairpins.

**Table 5:** The GO enrichment analysis of predicted targets of differentially expressed miRNAs with *P* < 0.05.

GO Id	Function	GO term	S gene number	TS gene number	B gene number	TB gene number
GO:0007032	Biological process	endosome organization	7	1,004	7	2,717
GO:0009117		nucleotide metabolic process	6	1,004	6	2,717
GO:0010951		negative regulation of endopeptidase activity	5	1,004	5	2,717
GO:0030030		cell projection organization	5	1,004	5	2,717
GO:0008654		phospholipid biosynthetic process	6	1,004	7	2,717
GO:0000910		cytokinesis	6	1,004	7	2,717
GO:0045860		positive regulation of protein kinase activity	6	1,004	7	2,717
GO:0008016		regulation of heart contraction	4	1,004	4	2,717
GO:0008150		biological process	4	1,004	4	2,717
GO:0016575		histone deacetylation	5	1,004	6	2,717
GO:0007169		transmembrane receptor protein tyrosine kinase signaling pathway	5	1,004	6	2,717
GO:0030162		regulation of proteolysis	5	1,004	6	2,717
GO:0071230		cellular response to amino acid stimulus	6	1,004	8	2,717
GO:0007243		intracellular protein kinase cascade	7	1,004	10	2,717
GO:0005739	Cellular component	mitochondrion	95	1,004	190	2,717
GO:0005829		cytosol	51	1,004	103	2,717
GO:0035098		ESC/E (Z) complex	5	1,004	5	2,717
GO:0016020		membrane	189	1,004	448	2,717
GO:0031965		nuclear membrane	12	1,004	18	2,717
GO:0005793		endoplasmic reticulum-Golgi intermediate compartment	6	1,004	7	2,717
GO:0005737		cytoplasm	314	1,004	780	2,717
GO:0005783		endoplasmic reticulum	53	1,004	112	2,717
GO:0005681		spliceosomal complex	6	1,004	8	2,717
GO:0005764		lysosome	10	1,004	16	2,717
GO:0016605		PML body	7	1,004	10	2,717
GO:0005819		spindle	8	1,004	12	2,717
GO:0009897		external side of plasma membrane	14	1,004	25	2,717
GO:0016787	Molecular function	hydrolase activity	68	1,004	139	2,717
GO:0019899		enzyme binding	13	1,004	18	2,717
GO:0003777		microtubule motor activity	6	1,004	6	2,717
GO:0042802		identical protein binding	30	1,004	55	2,717
GO:0016791		phosphatase activity	5	1,004	5	2,717
GO:0016301		kinase activity	14	1,004	23	2,717
GO:0008022		protein C-terminus binding	13	1,004	21	2,717
GO:0000166		nucleotide binding	155	1,004	372	2,717
GO:0003725		double-stranded RNA binding	5	1,004	6	2,717
GO:0016874		ligase activity	22	1,004	42	2,717
GO:0005524		ATP binding	115	1,004	271	2,717
GO:0015293		symporter activity	6	1,004	8	2,717
GO:0008026		ATP-dependent helicase activity	7	1,004	10	2,717
GO:0019003		GDP binding	7	1,004	10	2,717
GO:0016491		oxidoreductase activity	28	1,004	57	2,717
GO:0004842		ubiquitin-protein ligase activity	14	1,004	25	2,717
GO:0004386		helicase activity	12	1,004	21	2,717
GO:0000287		magnesium ion binding	16	1,004	30	2,717

**Table 6:** The KEGG pathway analysis of predicted targets of differentially expressed miRNAs with *P* < 0.05.

Pathway Id	Pathway description	S gene number	TS gene number	B gene number	TB gene number
ko01100	Metabolic pathways	244	927	880	3,670
ko04510	Focal adhesion	53	927	169	3,670
ko04141	Protein processing in endoplasmic reticulum	50	927	132	3,670
ko03013	RNA transport	39	927	114	3,670
ko04110	Cell cycle	37	927	103	3,670
ko03008	Ribosome biogenesis in eukaryotes	24	927	63	3,670
ko04650	Natural killer cell mediated cytotoxicity	24	927	66	3,670
ko03018	RNA degradation	22	927	56	3,670
ko00071	Fatty acid metabolism	16	927	30	3,670
ko00270	Cysteine and methionine metabolism	16	927	31	3,670
ko00520	Amino sugar and nucleotide sugar metabolism	16	927	39	3,670
ko03022	Basal transcription factors	14	927	32	3,670
ko00640	Propanoate metabolism	12	927	25	3,670
ko00760	Nicotinate and nicotinamide metabolism	9	927	19	3,670
ko00450	Selenocompound metabolism	7	927	14	3,670
ko04122	Sulfur relay system	6	927	10	3,670
