# Supplementary material for: Effect of dietary Astragalus Polysaccharide supplements on testicular miRNA expression profiles and enzymatic changes of breeder cocks
Source: Sci Rep. 2017 Jan 5;7:38864. doi: 10.1038/srep38864 (PMC5214674; doi:10.1038/srep38864)
Supplement: Supplementary Information [file srep38864-s1.doc]

**Effect of dietary Astragalus Polysaccharide supplements on testicular miRNA expression profiles and enzymatic changes of breeder cocks** Shengru Wu ^¶^, Xiaochun Ren ^¶^, Yulong Li ^¶^, Wei Guo, Xinyu Lei, Junhu Yao^*^and Xiaojun Yang^*^

**Supplementary Information**

**S1 Figure. Conservation of the identified miRNA with other species.**

**S1 Table. Summary of all expression miRNAs in this study.**

**S2 Table.** **Conservative testicular differential expressed miRNAs induced by APS supplements in breeder cock compared with goat, cow and mouse testicular miRNAs.**

**S3 Table. Target genes of the 33 differential expression miRNAs induced by APS.**

**S4 Table. The interaction between miRNAs and their target genes which involved in different GO term.**

**S5 Table. The interaction between miRNAs and their target genes which involved in different KEGG term.**

**S6 Table. Primer sequences for PCR reactions in this study.**

**
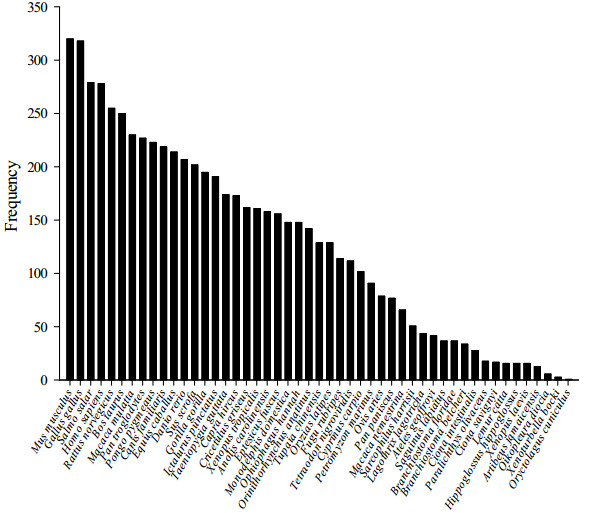
**

**S1 Figure. Conservation of the identified miRNA with other species.**

S1 Table. Summary of all expression miRNAs in this study.

| miRNA name | miRNA sequence | length | group |
| --- | --- | --- | --- |
| gga-miR-1a-3p_R+1_1ss2GT | TTGAATGTAAAGAAGTATGTAT | 22 | gp1a |
| gga-miR-1a-3p_R+1_2ss1TC20TG | CGGAATGTAAAGAAGTATGGAT | 22 | gp1a |
| gga-miR-1a-1-5p | ACATACTTCTTTATATGCCCATA | 23 | gp1a |
| gga-miR-1a-3p_R+1_1ss2GT | TTGAATGTAAAGAAGTATGTAT | 22 | gp1a |
| gga-miR-1a-3p_R+1_2ss1TC20TG | CGGAATGTAAAGAAGTATGGAT | 22 | gp1a |
| gga-miR-7_R+4 | TGGAAGACTAGTGATTTTGTTGTTAT | 26 | gp1a |
| gga-let-7g-5p_R+2_1ss9TA | TGAGGTAGAAGTTTGTACAGTTA | 23 | gp1a |
| gga-miR-7_R+2 | TGGAAGACTAGTGATTTTGTTGTT | 24 | gp1a |
| tgu-miR-7-1-3p_R+1 | CAACAAATCACAGTCTGCCATA | 22 | gp1a |
| gga-let-7a-5p | TGAGGTAGTAGGTTGTATAGTT | 22 | gp1a |
| gga-let-7k-3p | CTATACAATCTACTGTCTTTCC | 22 | gp1a |
| gga-let-7b_R+2_1ss1TN | NGAGGTAGTAGGTTGTGTGGTTTT | 24 | gp1a |
| gga-let-7i_R+1 | TGAGGTAGTAGTTTGTGCTGTT | 22 | gp1a |
| gga-let-7a-5p_2ss12GT18TC | TGAGGTAGTAGTTTGTACAGTT | 22 | gp1a |
| gga-let-7g-3p_1ss22CT | CTGTACAGGCCACTGCCTTGCT | 22 | gp1a |
| gga-let-7f-5p | TGAGGTAGTAGATTGTATAGTT | 22 | gp1a |
| gga-let-7f-3p_1ss22CT | CTATACAATCTATTGCCTTCCT | 22 | gp1a |
| gga-miR-7b_R-1_1ss17TG | TGGAAGACTAGTGATTGTTGT | 21 | gp1a |
| gga-let-7a-5p_R+2_1ss3AC | TGCGGTAGTAGGTTGTATAGTTAT | 24 | gp1a |
| gga-let-7f-5p | TGAGGTAGTAGATTGTATAGTT | 22 | gp1a |
| gga-let-7j-3p | CTATACAGTCTATTGCCTTCCT | 22 | gp1a |
| gga-let-7j-p3_1ss14AG | AACTATACAGTCTGTTGC | 18 | gp1a |
| gga-let-7c-5p | TGAGGTAGTAGGTTGTATGGTT | 22 | gp1a |
| gga-let-7c-3p_1ss12TC | CTGTACAACCTCCTAGCTTTCC | 22 | gp1a |
| gga-let-7a-5p_R+2_1ss3AC | TGCGGTAGTAGGTTGTATAGTTAT | 24 | gp1a |
| gga-let-7a-5p_1ss19AG | TGAGGTAGTAGGTTGTATGGTT | 22 | gp1a |
| gga-let-7k-3p_1ss22CT | CTATACAATCTACTGTCTTTCT | 22 | gp1a |
| gga-miR-7_R+4 | TGGAAGACTAGTGATTTTGTTGTTAT | 26 | gp1a |
| gga-miR-9-5p_L+1 | CTCTTTGGTTATCTAGCTGTATGA | 24 | gp1a |
| gga-miR-9-3p | TAAAGCTAGAGAACCGAATGT | 21 | gp1a |
| gga-miR-9-5p_L+1 | CTCTTTGGTTATCTAGCTGTATGA | 24 | gp1a |
| gga-miR-9-3p_L+1R+2_2ss12GT20TA | ATAAAGCTAGATAACCGAAAGTAG | 24 | gp1a |
| gga-miR-9-5p | TCTTTGGTTATCTAGCTGTATGA | 23 | gp1a |
| tgu-miR-9-3p | ATAAAGCTAGATAACCGAAAGT | 22 | gp1a |
| gga-miR-10a-5p | TACCCTGTAGATCCGAATTTGT | 22 | gp1a |
| gga-miR-10a-3p_L+1R-1 | CAAATTCGTATCTAGGGGAAT | 21 | gp1a |
| gga-miR-10a-5p_R+2_1ss1TN | NACCCTGTAGATCCGAATTTGTGA | 24 | gp1a |
| gga-miR-10b-5p_1ss12AT | TACCCTGTAGATCCGAATTTGT | 22 | gp1a |
| gga-miR-10b-3p_L+2R-1 | ACAGATTCGATTCTAGGGGAAT | 22 | gp1a |
| gga-miR-15b-5p_R-1 | TAGCAGCACATCATGGTTTGC | 21 | gp1a |
| gga-miR-15c-3p_R+1 | CAGACCATTCTGGGCTGCCTCAT | 23 | gp1a |
| gga-miR-15b-5p_R-1_1ss9CT | TAGCAGCATATCATGGTTTGC | 21 | gp1a |
| gga-miR-15b-3p_L+1_1ss22AT | CGAATCATTATTTGCTGCTTTT | 22 | gp1a |
| gga-miR-16-5p | TAGCAGCACGTAAATATTGGTG | 22 | gp1a |
| gga-miR-16-1-3p_R-1 | CCAGTATTAACTGTGCTGCTGA | 22 | gp1a |
| gga-miR-16c-5p_R+1_1ss1TN | NAGCAGCACGTAAATACTGGAGA | 23 | gp1a |
| gga-miR-16-5p | TAGCAGCACGTAAATATTGGTG | 22 | gp1a |
| gga-miR-19a-3p_R+1_1ss6AG | TGTGCGAATCTATGCAAAACTGAA | 24 | gp1a |
| gga-miR-19b-5p | AGTTTTGCAGGTTTGCATCCAGC | 23 | gp1a |
| gga-miR-20b-5p_R+1_1ss21TA | CAAAGTGCTCATAGTGCAGGAAGA | 24 | gp1a |
| gga-miR-20b-5p | CAAAGTGCTCATAGTGCAGGTAG | 23 | gp1a |
| gga-mir-20b-p3 | ACTGTAATGTGGGCACTTAC | 20 | gp1a |
| gga-miR-20a-3p_L-5 | ACTGCATTATAAGCACTTAAAGT | 23 | gp1a |
| gga-miR-21-5p | TAGCTTATCAGACTGATGTTGA | 22 | gp1a |
| gga-miR-21-3p_R+1 | CAACAACAGTCGGTAGGCTGTCT | 23 | gp1a |
| gga-miR-21-5p_R+1_1ss1TN | NAGCTTATCAGACTGATGTTGAA | 23 | gp1a |
| gga-miR-22-5p_R-1 | AGTTCTTCAGTGGCAAGCTTT | 21 | gp1a |
| gga-miR-22-3p | AAGCTGCCAGTTGAAGAACTGT | 22 | gp1a |
| gga-miR-23b-5p_L+1 | TGGGTTCCTGGCATGATGATTT | 22 | gp1a |
| gga-miR-23b-3p | ATCACATTGCCAGGGATTACC | 21 | gp1a |
| gga-miR-24-5p | GTGCCTACTGAGCTGATATCAGT | 23 | gp1a |
| gga-miR-24-3p_R-2 | TGGCTCAGTTCAGCAGGAAC | 20 | gp1a |
| gga-miR-26a-5p_R+1 | TTCAAGTAATCCAGGATAGGCT | 22 | gp1a |
| gga-miR-26a-3p_R-1 | CCTATTCTTGGTTACTTGCACT | 22 | gp1a |
| gga-miR-26a-5p_R+1 | TTCAAGTAATCCAGGATAGGCT | 22 | gp1a |
| gga-miR-27b-5p_R-1 | AGAGCTTAGCTGATTGGTGAAC | 22 | gp1a |
| gga-miR-27b-3p | TTCACAGTGGCTAAGTTCTGC | 21 | gp1a |
| gga-miR-29b-3p_R-1_2ss21GC22TC | TAGCACCATTTGAAATCAGTCC | 22 | gp1a |
| gga-miR-29b-2-5p_L-2 | CTGGTTTCACATGGTGGCTTAGA | 23 | gp1a |
| gga-miR-29c-3p_L+1R+1_1ss19GA | CTAGCACCATTTGAAATCAGTT | 22 | gp1a |
| gga-miR-29b-2-5p_L-2_2ss12CT20CT | CTGGTTTCATATGGTGGTTTAGA | 23 | gp1a |
| gga-miR-29b-3p | TAGCACCATTTGAAATCAGTGTT | 23 | gp1a |
| gga-miR-30c-5p_R+1_1ss1TN | NGTAAACATCCTACACTCTCAGCTT | 25 | gp1a |
| gga-miR-30c-1-3p_R+1_1ss5AC | TGGGCGAGGATTGTTTACGCCA | 22 | gp1a |
| gga-miR-30c-5p_R+1_1ss1TN | NGTAAACATCCTACACTCTCAGCTT | 25 | gp1a |
| gga-miR-30a-5p_R+2_1ss13CT | TGTAAACATCCTTGACTGGAAGCT | 24 | gp1a |
| gga-miR-30a-3p_1ss18GA | CTTTCAGTCGGATGTTTACAGC | 22 | gp1a |
| gga-miR-30a-5p_R+4_1ss15AC | TGTAAACATCCTCGCCTGGAAGCTTT | 26 | gp1a |
| gga-miR-30e-5p_R+5 | TGTAAACATCCTTGACTGGAAGCT | 24 | gp1a |
| gga-miR-30e-3p_L+1 | CTTTCAGTCGGATGTTTACAGC | 22 | gp1a |
| gga-miR-30c-5p | TGTAAACATCCTACACTCTCAGCT | 24 | gp1a |
| gga-miR-30c-2-3p_L+1 | CTGGGAGAAGGCTGTTTACTCT | 22 | gp1a |
| gga-miR-30b-5p | TGTAAACATCCTACACTCAGCT | 22 | gp1a |
| gga-miR-31-5p_R+1 | AGGCAAGATGTTGGCATAGCTGT | 23 | gp1a |
| gga-miR-31-3p | TGCTATGCCAACATATTGTCATC | 23 | gp1a |
| gga-miR-32-5p | TATTGCACATTACTAAGTTGC | 21 | gp1a |
| gga-miR-33-5p | GTGCATTGTAGTTGCATTGC | 20 | gp1a |
| gga-miR-33-3p_L+1R+1 | CAATGTTCCTGCAGTGCAGTAT | 22 | gp1a |
| gga-miR-33-5p | GTGCATTGTAGTTGCATTGC | 20 | gp1a |
| gga-miR-34a-5p_R-1 | TGGCAGTGTCTTAGCTGGTTGT | 22 | gp1a |
| gga-miR-34a-3p_1ss23AT | CAATCAGCAAGTATACTGCCCTT | 23 | gp1a |
| gga-miR-34b-5p_L-1R+1 | AGGCAGTGTAGTTAGCTGATTGT | 23 | gp1a |
| gga-miR-34c-3p_L-1R+1 | ATCACTAACCACACAGCCAGGT | 22 | gp1a |
| gga-miR-34a-5p_2ss7TG23TA | TGGCAGGGTCTTAGCTGGTTGTA | 23 | gp1a |
| gga-miR-92-5p | AGGTTGGGATCAGTTGCAATGCT | 23 | gp1a |
| gga-miR-92-3p_R+1 | TATTGCACTTGTCCCGGCCTGT | 22 | gp1a |
| gga-miR-92-3p_R+1 | TATTGCACTTGTCCCGGCCTGT | 22 | gp1a |
| gga-miR-99a-5p_R-1 | AACCCGTAGATCCGATCTTGT | 21 | gp1a |
| gga-miR-99a-3p_R+2 | CAAGCTCGCTTCTATGGGTCTGT | 23 | gp1a |
| gga-miR-100-5p_R+1_1ss12CN | AACCCGTAGATNCGAACTTGTGA | 23 | gp1a |
| gga-miR-100-5p_R+1 | AACCCGTAGATCCGAACTTGTGA | 23 | gp1a |
| gga-miR-100-3p_L+1R-1 | CAAGCTTGTATCTATAGGTAT | 21 | gp1a |
| gga-miR-101-3p_R-1_1ss11GA | GTACAGTACTATGATAACTGA | 21 | gp1a |
| gga-miR-101-1-5p_R-1 | TCGGTTATCATGGTACCGGTGC | 22 | gp1a |
| gga-miR-101-3p_R-1 | GTACAGTACTGTGATAACTGA | 21 | gp1a |
| gga-miR-101-2-5p_R-1 | TCAGTTATCACAGTGCTGATGC | 22 | gp1a |
| gga-miR-101-3p_L-1R+2 | TACAGTACTGTGATAACTGAAGG | 23 | gp1a |
| gga-miR-103-2-5p_R+1_1ss1AG | GGCTTCTTTACAGTGCTGCCTTGT | 24 | gp1a |
| gga-miR-103-3p | AGCAGCATTGTACAGGGCTATGA | 23 | gp1a |
| gga-miR-103-2-5p_R+1_1ss1AG | GGCTTCTTTACAGTGCTGCCTTGT | 24 | gp1a |
| gga-miR-103-3p | AGCAGCATTGTACAGGGCTATGA | 23 | gp1a |
| gga-miR-106-5p_R+1_1ss4AN | AAANGTGCTTACAGTGCAGGTAT | 23 | gp1a |
| gga-miR-106-3p_R+1_1ss4GN | ACTNCAGTATAAGCACTTCTGGA | 23 | gp1a |
| gga-miR-107-5p_R+1_1ss16TC | AGCTTCTTTACAGTGCTGCCTTGT | 24 | gp1a |
| gga-miR-107-3p_R-1 | AGCAGCATTGTACAGGGCTATC | 22 | gp1a |
| gga-miR-122-5p | TGGAGTGTGACAATGGTGTTTGT | 23 | gp1a |
| gga-miR-122-3p_R-1 | AACGCCATTATCACACTAAAT | 21 | gp1a |
| gga-miR-122-5p | TGGAGTGTGACAATGGTGTTTGT | 23 | gp1a |
| gga-miR-122-3p_R-1 | AACGCCATTATCACACTAAAT | 21 | gp1a |
| gga-miR-124b_L-1R-1_1ss12AG | TAAGGCACGCGGTGAATGCC | 20 | gp1a |
| gga-miR-124c-5p | CATTCACCGCGTGCCTTAATT | 21 | gp1a |
| gga-miR-124c-3p | TCAAGGTCCGCTGTGAACACGG | 22 | gp1a |
| gga-miR-124a-3p_L-1R-1 | TAAGGCACGCGGTGAATGCC | 20 | gp1a |
| gga-miR-125b-5p | TCCCTGAGACCCTAACTTGTGA | 22 | gp1a |
| gga-miR-125b-5p | TCCCTGAGACCCTAACTTGTGA | 22 | gp1a |
| gga-miR-125b-3p_R-1_1ss21CT | ACAAGTCAGGCTCTTGGGACT | 21 | gp1a |
| gga-miR-125b-3p_R-1_2ss1AN21CT | NCAAGTCAGGCTCTTGGGACT | 21 | gp1a |
| gga-miR-126-5p | CATTATTACTTTTGGTACGCG | 21 | gp1a |
| gga-miR-126-3p_R-2 | TCGTACCGTGAGTAATAATGC | 21 | gp1a |
| gga-miR-130b-5p_R-3_1ss5TC | CCTCCTTCCCTGTTGCACT | 19 | gp1a |
| gga-miR-130c-3p | CAGTGCAATGTTAAAAGGGCAT | 22 | gp1a |
| gga-miR-130a-5p | GCCCTTTTTCTGTTGTACTACT | 22 | gp1a |
| gga-miR-130a-3p_1ss10AG | CAGTGCAATGTTAAAAGGGCAT | 22 | gp1a |
| gga-miR-130c-5p_1ss10AC | GCCCTTTTTCTGTTGTACTACT | 22 | gp1a |
| gga-miR-133a-5p_R-1 | AGCTGGTAAAATGGAACCAAAT | 22 | gp1a |
| gga-miR-133a-3p | TTGGTCCCCTTCAACCAGCTGT | 22 | gp1a |
| gga-miR-133c-5p_L+1R-1_2ss12AT20GA | AGCTGGTAAAATGGAACCAAAT | 22 | gp1a |
| gga-miR-133c-3p_1ss22CT | TTGGTCCCCTTCAACCAGCTGT | 22 | gp1a |
| gga-miR-133b_L+1_1ss22AT | TTTGGTCCCCTTCAACCAGCTT | 22 | gp1a |
| gga-miR-135a-5p | TATGGCTTTTTATTCCTATGTGA | 23 | gp1a |
| gga-miR-135a-2-3p_L-1R+1 | TGTAGGGATGGAAGCCATGAAA | 22 | gp1a |
| gga-miR-135a-5p | TATGGCTTTTTATTCCTATGTGA | 23 | gp1a |
| gga-miR-135a-3-3p_R+1 | ATGTAGGGCGAAAAGCCATGGGA | 23 | gp1a |
| gga-miR-135a-5p_1ss23AT | TATGGCTTTTTATTCCTATGTGT | 23 | gp1a |
| gga-miR-135a-1-3p | ATATAGGGATTGAAGCCGTGCA | 22 | gp1a |
| gga-miR-140-5p_L+1 | CAGTGGTTTTACCCTATGGTAG | 22 | gp1a |
| gga-miR-140-3p_L+1 | ACCACAGGGTAGAACCACGGAC | 22 | gp1a |
| gga-miR-142-5p_L-2R+1 | CATAAAGTAGAAAGCACTACT | 21 | gp1a |
| gga-miR-142-3p | TGTAGTGTTTCCTACTTTATGG | 22 | gp1a |
| gga-miR-144-5p_R+1 | GGATATCATCATATACTGTAAGT | 23 | gp1a |
| gga-miR-144-5p_R+1 | GGATATCATCATATACTGTAAGT | 23 | gp1a |
| gga-miR-144-3p_L-1R+1 | TACAGTATAGATGATGTACTCT | 22 | gp1a |
| gga-miR-144-3p_L-1R+1 | TACAGTATAGATGATGTACTCT | 22 | gp1a |
| gga-miR-146c-5p_4ss18GA20AG22TG23GT | TGAGAACTGAATTCCATAGGCGT | 23 | gp1a |
| gga-miR-146b-3p_L+2 | TGCCCTATGGATTCAGTTCTGC | 22 | gp1a |
| gga-miR-146c-5p_L-1R-1 | GAGAACTGAATTCCATGGACT | 21 | gp1a |
| gga-miR-146c-3p_L-1R-1 | GTCCATGGTATTCAGTTCTC | 20 | gp1a |
| gga-miR-146a-5p_R+2 | TGAGAACTGAATTCCATGGGTTGT | 24 | gp1a |
| gga-miR-146a-3p_R-1 | ACCCATGGGGCTCAGTTCTTCA | 22 | gp1a |
| gga-miR-146c-5p_R-1 | TGAGAACTGAATTCCATGGACT | 22 | gp1a |
| gga-miR-146c-3p_L-1 | GTCCATGGTATTCAGTTCTCT | 21 | gp1a |
| gga-miR-148a-5p | AAAGTTCTGTGACACTCAGACT | 22 | gp1a |
| gga-miR-148a-3p | TCAGTGCACTACAGAACTTTGT | 22 | gp1a |
| gga-miR-148a-3p_R+1_1ss1TN | NCAGTGCACTACAGAACTTTGTA | 23 | gp1a |
| gga-miR-153-5p_L+1R+1_1ss22CA | GTCATTTTTGTGATGTTGCAGATT | 24 | gp1a |
| gga-miR-153-5p_L+1 | GTCATTTTTGTGATGTTGCAGCT | 23 | gp1a |
| gga-miR-153-3p_R+2 | TTGCATAGTCACAAAAGTGATC | 22 | gp1a |
| gga-miR-155_R+1 | TTAATGCTAATCGTGATAGGGGT | 23 | gp1a |
| gga-miR-181b-5p_R+2 | AACATTCATTGCTGTCGGTGGGGA | 24 | gp1a |
| gga-miR-181b-1-3p_L+6R-1_1ss26AT | ACAAGCTCACTGAACAATGAATGCAT | 26 | gp1a |
| gga-miR-181a-5p | AACATTCAACGCTGTCGGTGAGT | 23 | gp1a |
| gga-miR-181a-3p_1ss16TC | ACCATCGACCGTTGACTGTACC | 22 | gp1a |
| gga-miR-181b-1-3p_L+6R-1_1ss26AT | ACAAGCTCACTGAACAATGAATGCAT | 26 | gp1a |
| gga-miR-181a-5p | AACATTCAACGCTGTCGGTGAGT | 23 | gp1a |
| gga-miR-181a-3p_1ss16TC | ACCATCGACCGTTGACTGTACC | 22 | gp1a |
| gga-miR-181b-5p_R+1 | AACATTCATTGCTGTCGGTGGGT | 23 | gp1a |
| gga-miR-181b-2-3p_L+2R-3 | ACTCACTGATCAATGAATGC | 20 | gp1a |
| gga-miR-187-5p_2ss2GC22AT | GCCTACAACACAGGACATGGGT | 22 | gp1a |
| gga-miR-187-5p_L-1 | GCTACAACACAGGACATGGGA | 21 | gp1a |
| gga-miR-187-3p_R+3_1 | TCGTGTCTTGTGTTGCAGCCAGT | 23 | gp1a |
| gga-miR-187-3p_R+3_2 | TCGTGTCTTGTGTTGCAGCCATT | 23 | gp1a |
| gga-miR-190a-5p_R+1 | TGATATGTTTGATATATTAGGTT | 23 | gp1a |
| gga-miR-190a-3p_L+1 | ACTATATATCAAACATATTCCT | 22 | gp1a |
| gga-miR-193b-3p_2ss1AN24TA | NACTGGCCCACAAAGTCCCGCTTA | 24 | gp1a |
| gga-miR-193b-3p_R-2 | AACTGGCCCACAAAGTCCCGCT | 22 | gp1a |
| gga-miR-194_R+1 | TGTAACAGCAACTCCATGTGGAC | 23 | gp1a |
| gga-miR-196-5p_R+2 | TAGGTAGTTTCATGTTGTTGGGC | 23 | gp1a |
| gga-miR-196-1-3p_L+1R-1 | ACAAGAACATCAAACTACCTGA | 22 | gp1a |
| gga-miR-196-5p_R+2 | TAGGTAGTTTCATGTTGTTGGGC | 23 | gp1a |
| gga-miR-196-5p_R+1 | TAGGTAGTTTCATGTTGTTGGG | 22 | gp1a |
| gga-miR-199-5p | CCCAGTGTTCAGACTACCTGTTC | 23 | gp1a |
| gga-miR-199-3p_L-1R+2 | ACAGTAGTCTGCACATTGGTT | 21 | gp1a |
| gga-miR-199-3p_L-1R+4 | ACAGTAGTCTGCACATTGGTTTT | 23 | gp1a |
| gga-miR-199-5p_R-1_1ss7GT | CCCAGTTTTCAGACTACCTGTT | 22 | gp1a |
| gga-miR-199-3p_L-1R+5 | ACAGTAGTCTGCACATTGGTTAAG | 24 | gp1a |
| gga-mir-200a-p5 | CTGTGGGCATCTTACTAGACAGTGC | 25 | gp1a |
| gga-miR-200a-3p_R+1 | TAACACTGTCTGGTAACGATGTT | 23 | gp1a |
| gga-miR-200b-5p_L+2 | CATCTTACTGGGCAGCATTGGA | 22 | gp1a |
| gga-miR-200b-3p | TAATACTGCCTGGTAATGATGAT | 23 | gp1a |
| gga-miR-202-5p_L-1 | TTCCTATGCATATACTTCTTT | 21 | gp1a |
| gga-miR-202-3p_R-1 | AGAGGCATAGAGCATGGGAAA | 21 | gp1a |
| gga-miR-203a_R+1 | GTGAAATGTTTAGGACCACTTGT | 23 | gp1a |
| gga-miR-204_R+1 | TTCCCTTTGTCATCCTATGCCTA | 23 | gp1a |
| gga-miR-204 | TTCCCTTTGTCATCCTATGCCT | 22 | gp1a |
| hsa-miR-204-3p_R+2 | GCTGGGAAGGCAAAGGGACGTTT | 23 | gp1a |
| gga-miR-204_R+2 | TTCCCTTTGTCATCCTATGCCTGT | 24 | gp1a |
| gga-miR-205b_R+1 | CCCTTCATTCCACCGGAATCTGT | 23 | gp1a |
| gga-mir-205b-p3_1ss24CT | GATTTCAGTGAAATGAAGCCCATT | 24 | gp1a |
| gga-miR-205a_R+1 | TCCTTCATTCCACCGGAGTCTGT | 23 | gp1a |
| gga-miR-214_R+2 | ACAGCAGGCACAGACAGGCAGAA | 23 | gp1a |
| gga-miR-215-5p_R+1 | ATGACCTATGAATTGACAGACT | 22 | gp1a |
| gga-miR-215-3p_R-1 | CCTGTCATTTCTATAGGCCAAT | 22 | gp1a |
| gga-miR-216c_R-2 | ATCTCTACAGGTAATTGTGA | 20 | gp1a |
| gga-mir-216c-p3 | TCACATTTGCCTGCAGAGATTT | 22 | gp1a |
| gga-miR-216b | AAATCTCTGCAGGCAAATGTGA | 22 | gp1a |
| gga-miR-218-5p | TTGTGCTTGATCTAACCATGT | 21 | gp1a |
| gga-miR-218-3p_R-1 | CATGGTTCTGTCAAGCACCAT | 21 | gp1a |
| gga-miR-219b_R-1 | CACAAGAATTGCGTTTGGACA | 21 | gp1a |
| gga-miR-221-5p_R-5_2ss21TA22TA | AACCTGGCATACAATGTAGAAA | 22 | gp1a |
| gga-miR-221-5p_L-1R-4 | ACCTGGCATACAATGTAGATTT | 22 | gp1a |
| gga-miR-221-3p | AGCTACATTGTCTGCTGGGTTTC | 23 | gp1a |
| gga-miR-222a_R-1 | AGCTACATCTGGCTACTGGGTCT | 23 | gp1a |
| gga-miR-301b-5p_1ss8CG | GCTCTGAGTTTATTGCACTACT | 22 | gp1a |
| gga-miR-302a_L+1R-1 | AAAGTGCTTCCATGTTTTAGTG | 22 | gp1a |
| gga-miR-375_R-1 | TTTGTTCGTTCGGCTCGCGTT | 21 | gp1a |
| gga-miR-383-5p | AGATCAGAAGGTGATTGTGGCT | 22 | gp1a |
| gga-miR-383-3p | CCACAGCACTGCCTGGTCAGA | 21 | gp1a |
| gga-miR-429-3p | TAATACTGTCTGGTAATGCCGT | 22 | gp1a |
| gga-miR-449b-5p_R-1 | AGGCAGTGTGCTGTTAGCGGCT | 22 | gp1a |
| gga-miR-449b-3p_R+1 | CAGTCACTACCACACTGCCACT | 22 | gp1a |
| gga-miR-449a | TGGCAGTGTATGTTAGCTGGT | 21 | gp1a |
| gga-mir-449a-p3_1ss11CT | TCAGCTAACATGCAGTTGCTAAC | 23 | gp1a |
| gga-miR-449c-5p_R+1 | TGGCAGTGCGTGTTAGCTGGCTGTT | 25 | gp1a |
| gga-miR-449c-3p_L+1 | ACAGCTGCTCACGGCACTCCAGA | 23 | gp1a |
| gga-miR-455-5p | TATGTGCCCTTGGACTACATCG | 22 | gp1a |
| gga-miR-455-3p_L-1R+1 | GCAGTCCATGGGCATATACACC | 22 | gp1a |
| gga-miR-456-5p_L+1R-5 | GCAGGCATCTTCTCAGCCTAC | 21 | gp1a |
| gga-miR-456-3p_1ss22AT | CAGGCTGGTTAGATGGTTGTCT | 22 | gp1a |
| gga-miR-458a-3p | ATAGCTCTTTGAATGGTACTGC | 22 | gp1a |
| gga-miR-460b-5p | TCCTCATTGTACATGCTGTGTG | 22 | gp1a |
| gga-miR-460b-3p_R-1_1ss22AT | CACAGCGCATGCAATGTGGACT | 22 | gp1a |
| gga-miR-489-5p | TGGTCGTATGTATGACGTCATT | 22 | gp1a |
| gga-miR-489-3p_L-2R+1 | TGACATCATATGTACGGCTGCT | 22 | gp1a |
| gga-miR-490-3p_R+1 | CAACCTGGAGGACTCCATGCTGT | 23 | gp1a |
| gga-miR-499-5p_R-2 | TTAAGACTTGTAGTGATGTTT | 21 | gp1a |
| gga-miR-499-3p | AACATCACTTTAAGTCTGTGCT | 22 | gp1a |
| gga-miR-1329-5p_L-1R+2 | ACAGTGATCACGTTACGATGGAT | 23 | gp1a |
| gga-miR-1329-3p | CCTCGTAGCTTGATCACGATAT | 22 | gp1a |
| gga-miR-1416-5p_R+1 | TCCTTAACTCATGCCGCTGTGC | 22 | gp1a |
| gga-miR-1416-3p_R-1 | ACAATTGTATGAGTTGAGTAC | 21 | gp1a |
| gga-miR-1451-5p | TCGCACAGGAGCAAGTTACCGC | 22 | gp1a |
| gga-miR-1451-3p_R-1 | CGTAACTCGCTGCTGTGAGAGG | 22 | gp1a |
| gga-miR-1451-5p_R+1_1ss16TC | TCGCACAGGAGCAAGCTACCGCT | 23 | gp1a |
| gga-miR-1451-3p_R+1_2ss22GT23CT | CGTAACTCGCTGCTGTGAGAGTTA | 24 | gp1a |
| gga-miR-1452 | TTGAGATAAGACAGAGGATATT | 22 | gp1a |
| gga-miR-1456-5p_L-1 | AAAGGACGGAGGCGGCCCGCGC | 22 | gp1a |
| gga-miR-1456-3p_R+1 | CGGGGTCGTCGCCGTCCCTTCC | 22 | gp1a |
| gga-miR-1467-5p | TCTCAGCTACATCGGTGTAAATC | 23 | gp1a |
| gga-miR-1467-5p | TCTCAGCTACATCGGTGTAAATC | 23 | gp1a |
| gga-miR-1467-3p_L+1 | TTCACACCAGAGTAACTGGGATC | 23 | gp1a |
| gga-miR-1467-3p_L+1 | TTCACACCAGAGTAACTGGGATC | 23 | gp1a |
| gga-miR-1550-3p_L-1 | TTCACTTACACTGCTCACTGGT | 22 | gp1a |
| gga-miR-1552-5p_R+1 | TTAGTGCGCGGTAAGCTAGGGTGT | 24 | gp1a |
| gga-miR-1552-3p_R+1 | CTAGCTGCTCTGCACTGACTGT | 22 | gp1a |
| gga-miR-1553-5p_R+2 | AGTCCTGTCCTGTCTACCTACTGTTT | 26 | gp1a |
| gga-miR-1559-5p | TTCGATGCTTGTATGCTACTCC | 22 | gp1a |
| gga-miR-1559-3p_L-2R+2 | TTACATGTATGCATCGAGCAGA | 22 | gp1a |
| gga-miR-1561_L-2R-1 | CGGGTTTGCGGCCGCTTT | 18 | gp1a |
| gga-miR-1573_R+4 | TGTTCTGCAGGTGCCAGTCTGAGA | 24 | gp1a |
| gga-miR-1574-3p_L-4_1ss17AG | GAGGATGTCAGGGAGCTT | 18 | gp1a |
| gga-miR-1590_R+1 | AGGGACAAATTGCTCTCTGGAAGT | 24 | gp1a |
| gga-mir-1594-p5_1ss12GA | TGGGCATGGTGAGGATGGGTT | 21 | gp1a |
| gga-mir-1594-p3_1ss14CG | TGGGGTTGGGCTTGGGGAT | 19 | gp1a |
| gga-miR-1602 | TGGGCTCTGCATCACCCCATGG | 22 | gp1a |
| gga-miR-1604 | TGGGCCCAGGGCTGTGCTGGAGA | 23 | gp1a |
| gga-miR-1611_L-3R-1_1ss16GA | GGGCTTGCAGGCAGTGTG | 18 | gp1a |
| gga-miR-1618-3p_L+1 | AGAGCCCGGAGCCAGGCTGCTGT | 23 | gp1a |
| gga-miR-1631_L+1R+3 | GAAACTGGCGACGTGGGACGGTCACC | 26 | gp1a |
| gga-miR-1641 | TGAGGATTAATGACTGTCTGGG | 22 | gp1a |
| gga-miR-1643-5p_R-2 | TCTTATCACGAGGAGCACTT | 20 | gp1a |
| gga-mir-1648-p5_1ss11TG | GCGGCTCGGCGCGGCTCCG | 19 | gp1a |
| gga-miR-1649-5p_R+3 | TCCTGCAGAAGGTGCGGCTGTGT | 23 | gp1a |
| gga-miR-1650 | TCCTCTGAGCTCAGCTTCGCCTCT | 24 | gp1a |
| gga-mir-1651-p5 | AGGGGGTGGAAGCTAAGAC | 19 | gp1a |
| gga-miR-1651-3p_R+1 | TTGCTTTTGTTGGCCTCTGCTGT | 23 | gp1a |
| gga-miR-1651-3p_R+1_1ss1TN | NTGCTTTTGTTGGCCTCTGCTGT | 23 | gp1a |
| gga-miR-1655-5p | TCAGCTGGTCTTGGGTAAATGC | 22 | gp1a |
| gga-miR-1656 | TCACCAGCGGGCATTGGCATCA | 22 | gp1a |
| gga-miR-1662_R-1_2ss2TA21AT | TAGACATCATCATACTTGGGT | 21 | gp1a |
| gga-miR-1664-3p_L+1 | TTCTGTGACCTCATTTACCTCC | 22 | gp1a |
| gga-miR-1665_L+1R+1 | GTAAGGCCCAGGAGCGCTGCAGCT | 24 | gp1a |
| gga-mir-1665-p3 | TGGAGTGTCTCAGGGCCTTGC | 21 | gp1a |
| gga-miR-1668-3p_R-1 | TGCTGTGTCCTCACTCACCT | 20 | gp1a |
| gga-miR-1676-5p_L-2R+1 | TGACTCATCCCCTTTGCTCCT | 21 | gp1a |
| gga-miR-1677-5p | TCCTGCACCGCTGAAGTCAAT | 21 | gp1a |
| gga-miR-1677-3p_L+1 | TTGACTTCAGTAGGAGCAGGATT | 23 | gp1a |
| gga-miR-1684a-5p | AGCTCTGCTTCCTCATACATAC | 22 | gp1a |
| gga-miR-1684b-3p | AAGTATGAGGAAATGGAGATCT | 22 | gp1a |
| gga-miR-1684a-3p_L+1 | TAAGTATGAGGAAATGGAGCTCT | 23 | gp1a |
| gga-miR-1684a-3p_L+1 | TAAGTATGAGGAAATGGAGCTCT | 23 | gp1a |
| gga-miR-1703-5p | AGAGGCTGTAGGTCCCGTGCTTT | 23 | gp1a |
| gga-miR-1705 | AATCTGGAAGTCAGCACATGCT | 22 | gp1a |
| gga-miR-1707_1ss23GA | TTTGAGCGGGATCTGTTATCGTA | 23 | gp1a |
| gga-miR-1720-5p_R-2 | TGATCACCTCGGACGTTGCT | 20 | gp1a |
| gga-miR-1720-3p | AAGCAACGAGAGGTCGGTCTGA | 22 | gp1a |
| gga-mir-1723-p5_1ss21AT | AGCTGCACATTTCGTGTCCCT | 21 | gp1a |
| gga-miR-1723_L-3R+2 | GAGCGGAATGTGCAGCCTCACT | 22 | gp1a |
| gga-miR-1726 | AAGCTTGTTGGGTTTGGTTTGT | 22 | gp1a |
| gga-miR-1729-5p_R-1_2ss12AC19TG | ATCCCTTACTCCCATGAGGAGT | 22 | gp1a |
| gga-miR-1729-5p_R-1 | ATCCCTTACTCACATGAGTAGT | 22 | gp1a |
| gga-miR-1729-3p | CTACTCGGTGAGTAAGGATAGC | 22 | gp1a |
| gga-miR-1734_L-1R+2 | TATGCCCCAATGTATAAGCTAT | 22 | gp1a |
| gga-miR-1736-5p_R+2 | AGGGCAATTCCTGATAGGTGCTGT | 24 | gp1a |
| gga-miR-1736-3p_R-1 | AGCACCTTCGGAACTGTCCTC | 21 | gp1a |
| gga-miR-1744-3p | ACTTCAACAGGAGCAAGACTGA | 22 | gp1a |
| gga-miR-1746_R+1 | CTCAGAGCTGTGGTCCCATGGTT | 23 | gp1a |
| gga-miR-1747-5p_R+1 | TGCACTTGAATGGAGTTCTGGGA | 23 | gp1a |
| gga-miR-1747-3p_L+1R-2 | TCCTGAATCCCTTCAAGTGGA | 21 | gp1a |
| gga-miR-1751-5p_R-1 | TGAGCTGCTCTGTTCCTGCATC | 22 | gp1a |
| gga-mir-1751-p3 | TGAAGGACGAGGGCAGACCGCGT | 23 | gp1a |
| gga-miR-1752_L-2 | CTGTTGGAGAGAAGAGACA | 19 | gp1a |
| gga-miR-1756a | TCCAGTGATTCACACCAGCTGT | 22 | gp1a |
| gga-mir-1756b-p5_1ss13CG | GCTGGTGTAAATGAGTGT | 18 | gp1a |
| gga-miR-1756b_R-1 | TTCTCTGATTTACACTAGCTGA | 22 | gp1a |
| gga-mir-1757-p5 | TGGATGAACAAAACTCTTCTGGTCCG | 26 | gp1a |
| gga-mir-1757-p3_1ss24GA | TTAGTTCAATTAGCGCTTCAGAGAC | 25 | gp1a |
| gga-miR-1760_R-4 | TACCTCCTGTCTGACTGAT | 19 | gp1a |
| gga-mir-1768-p5 | CTCATCAGCATGACAATCCTCT | 22 | gp1a |
| gga-mir-1768-p5 | CTCATCAGCATGACAATCCTCT | 22 | gp1a |
| gga-mir-1768-p3 | AGGATTGTCGTGCTGATGAGTT | 22 | gp1a |
| gga-miR-1779_R-1 | AGACGTGGACTGGAACACCTGA | 22 | gp1a |
| gga-miR-1781-5p_R-1 | AACAGCTGAGTGATTTAAAGC | 21 | gp1a |
| gga-miR-1781-3p | TTTAAATCATGCAGCTGTTGA | 21 | gp1a |
| gga-miR-1782_L+2R-1 | AAACATTCATTGGAGCAGGGAC | 22 | gp1a |
| gga-miR-1784-5p | TTCTGCTCTTATTGAAATCAGT | 22 | gp1a |
| gga-miR-1788-5p_R+1 | GGCTTGTTTTCCGTTGCCTGCGT | 23 | gp1a |
| gga-miR-1788-5p_R+1 | GGCTTGTTTTCCGTTGCCTGCGT | 23 | gp1a |
| gga-mir-1789-p5 | TGCACCCACAAGCACAACCAAGC | 23 | gp1a |
| gga-miR-1789_R-1 | TGGGTGTACTTGCGGATGAAT | 21 | gp1a |
| gga-miR-1790_L-1R-1_1ss5TG | GGGGGAGGGCGGTGGGGG | 18 | gp1a |
| gga-miR-1792_L+1R-3 | TGCGATAGCTCAGGAACACTG | 21 | gp1a |
| gga-miR-1795_L+4 | AGAGGCAAGATGAAGAACAGGCCTGT | 26 | gp1a |
| gga-miR-1795_L-1R-3_1ss11GC | CAAGATGAACAACAGGCC | 18 | gp1a |
| gga-mir-1797-p3_1ss17CG | ACAGCTTGGAACTGAGGA | 18 | gp1a |
| gga-miR-1798-3p_L-3R+2 | TTTTCAGAAGTGTAGCGTTTA | 21 | gp1a |
| gga-miR-1800 | ACTACGTGATGCGATCTGATG | 21 | gp1a |
| gga-miR-1803_1ss21GT | ATTGACTTCAGCGGGGTTTGT | 21 | gp1a |
| gga-miR-1805-5p_L-1 | AGTTGTAGTCTTTCAAACAGA | 21 | gp1a |
| gga-miR-1805-3p_R+1 | TGTATTGGAACACTACAGCTCC | 22 | gp1a |
| gga-mir-1806-p5 | TATTCCTTGCAGGTTTTGCCTGT | 23 | gp1a |
| gga-mir-1807-p5 | AGCATTAGCCTAATTTTGCTCC | 22 | gp1a |
| gga-miR-1807_L+2R-3 | AGGCAGAATGAGGCTAGAGCT | 21 | gp1a |
| gga-mir-2130-p5 | AAACGGGGGCTGAGCATCGGC | 21 | gp1a |
| gga-miR-2130_R-1 | TCCCAGTGGAGCTCTGCAAGGA | 22 | gp1a |
| gga-miR-2131-5p | ATGCAGAAGTGCACGGAAACAGCT | 24 | gp1a |
| gga-miR-2131-3p_R+1 | CTGTTACTGTTCTTCTGATGGT | 22 | gp1a |
| gga-miR-2188-5p | AAGGTCCAACCTCACATGTCCT | 22 | gp1a |
| gga-miR-2188-3p_L-1R+1 | ATATATGTGGTCAGACCTATCT | 22 | gp1a |
| tgu-miR-2954-5p_L+1 | TGCTGAGAGGGCTTGGGGAGAGGA | 24 | gp1a |
| gga-miR-2954_R+2 | CATCCCCATTCCACTCCTAGCAGT | 24 | gp1a |
| gga-mir-3523-p5_1ss24GT | GAGTGCGAGGCGCTGCGGGGGCTT | 24 | gp1a |
| gga-miR-3523 | CCGCGCAGTGCCTCGTCCTCGA | 22 | gp1a |
| gga-miR-3524a_1ss22GT | CAGAATCGCAGAATGGCTGAGT | 22 | gp1a |
| gga-mir-3524b-p3_1ss18TG | TCACAGAATCACAGAATGGT | 20 | gp1a |
| gga-mir-3524a-p5_1ss12GA | ATCACAGAATCACAGAATGGC | 21 | gp1a |
| gga-miR-3525 | CAGCCATTCTGCGATTCTGTGA | 22 | gp1a |
| gga-miR-3529 | AGGCAGACTGTGACTTGTTGT | 21 | gp1a |
| gga-miR-3530-3p_L+1R+1 | ACAATGGTGTGAGCTGGGATGGA | 23 | gp1a |
| gga-miR-3530-5p_L+1_1ss22GT | CGCTCTGCTCGCACCATTGTGT | 22 | gp1a |
| gga-miR-3531-5p_R+2 | TCCTTGTTTGGGTTGTAATGAAA | 23 | gp1a |
| gga-miR-3531-3p_1ss23AT | CCTTGCAACACAAACAGGAGACT | 23 | gp1a |
| gga-miR-3532-3p | TTGGAGGCTGCAGTGTCATGGT | 22 | gp1a |
| gga-miR-3534_L+1R-5 | GTGTGGGTTGACAGTGCT | 18 | gp1a |
| gga-miR-3540 | ATGGTGGAAGAACAAGGCCTGC | 22 | gp1a |
| gga-miR-3594-3p | TCTGCATCGCTGGGCTGTGTCC | 22 | gp1a |
| gga-miR-3607-5p | TACATATGATGAGCTTTGCAGT | 22 | gp1a |
| gga-miR-3607-3p_R-4 | CTGTAAACGCTTTCTGATG | 19 | gp1a |
| gga-miR-6543-3p_L+4R+2_1ss5GA | CACAACCTCCTTTCAGGTCACTGT | 24 | gp1a |
| gga-miR-6544-5p_L-1R+1 | TCAGAAAAGGATATGAATTGTC | 22 | gp1a |
| gga-miR-6544-3p_L+1R-4 | CAAGTTGTATTTCTTTTCTGA | 21 | gp1a |
| gga-miR-6546-5p | AGGAGCGCCTCTGAGCTCCCCT | 22 | gp1a |
| gga-miR-6549-5p | AGCCTTCTGTTGTGCATCTGAGA | 23 | gp1a |
| gga-mir-6549-p3_1ss8AG | TTGTCAGGTCACAGCTGTAAGCAGA | 25 | gp1a |
| gga-miR-6552-3p_L+2R+1 | TTCAACAAGAAGTGGACAGACTGT | 24 | gp1a |
| gga-miR-6555-5p_L+3 | TCTGATCTGCAGAGCCCACAACTAG | 25 | gp1a |
| gga-miR-6556-5p_R-1 | AAGGCTGCTCGGCCCGCGCTG | 21 | gp1a |
| gga-miR-6557-3p_R-1 | CGCGCCGATTGTCTCCTCCGGGCA | 24 | gp1a |
| gga-miR-6560-5p_R+3 | TGTGTTGCTCCATACTGTGGTCTGTC | 26 | gp1a |
| gga-mir-6562-p5_1ss13TC | GCTCCGGAGTTCCTGTAGGGGAAA | 24 | gp1a |
| gga-mir-6562-p3 | TTCAGACGCCTCAGTCATTTCTTGCT | 26 | gp1a |
| gga-miR-6563-5p_R+2 | GTTGGTCGGTTGTGCTTAGAGATT | 24 | gp1a |
| gga-miR-6565-3p_R+1 | TCTGTGCTTGTGTACTCATAGT | 22 | gp1a |
| gga-mir-6566-p5 | GTCATTCCTGGATTTTCTCTGTCATC | 26 | gp1a |
| gga-miR-6568-3p | ACAACCAAGATGGCGCCCGGGC | 22 | gp1a |
| gga-miR-6570-5p_R-2 | AGGACCCACTGGATCTGCATCT | 22 | gp1a |
| gga-miR-6575-5p | TTGTCAGCTTGGGGAAGCTCTT | 22 | gp1a |
| gga-miR-6575-3p_1ss22AT | GAGTTTTCCCAGGCTGATGTCT | 22 | gp1a |
| gga-mir-6578-p3 | ACTATGGGGCACTCTGCTGACCGCT | 25 | gp1a |
| gga-miR-6582-5p_R+1 | TTAGAGAGACACTCATAGAGA | 21 | gp1a |
| gga-miR-6582-3p_L+1R-2 | TCACTCTGGGTGTTTCTCTTGC | 22 | gp1a |
| gga-miR-6582-3p_L-5_1ss22AT | TGGGTGTTTCTCTTGCTG | 18 | gp1a |
| gga-miR-6586-5p_R-1 | TGCTGCCAGATAGAAGTTCACC | 22 | gp1a |
| gga-miR-6590-3p_R+2 | TTACTTCTGTTCCTGATCATCAGA | 24 | gp1a |
| gga-miR-6599-3p | TGACGGATCCTGGCTCCCTCCG | 22 | gp1a |
| gga-miR-6608-3p_L-3R-1_1ss14GA | ACTGCAAGAGAGAGAGAGA | 19 | gp1a |
| gga-miR-6611-5p_R-1_1ss20TC | TGCTGGATCCAGTGACACTCC | 21 | gp1a |
| gga-mir-6613-p3_1ss11AT | GAGCTGGAAGTATCATGT | 18 | gp1a |
| gga-mir-6619-p3 | CAGCACAAATGACGTGTGGCGTG | 23 | gp1a |
| gga-miR-6620-5p_L-2R-3_1ss20AT | AGATGGAAGCGCTGCGCT | 18 | gp1a |
| gga-miR-6625-5p_L+1R-2 | TTGGGGGAACTGGAAGCAAGA | 21 | gp1a |
| gga-miR-6643-5p_L-1 | CAGGGCTGGCAGGGGAGGGT | 20 | gp1a |
| gga-mir-6643-p3_1ss22CT | CCACCCCTGCCAGCCTTGGGAT | 22 | gp1a |
| gga-miR-6645-5p | TGGGAGGTAGCAGTGGTGGTGA | 22 | gp1a |
| gga-mir-6645-p3_1ss22GT | ACCCCCACTGCTCTCTCCCAGT | 22 | gp1a |
| gga-miR-6647-5p_1ss2GA | CACTCTGGCCATGTGACGCAGCT | 23 | gp1a |
| gga-miR-6648-5p_L+1R-1_1ss22TA | AAGGAGCGTTCAGAATGCCGGA | 22 | gp1a |
| gga-miR-6648-5p_L+1R-1_1ss22TA | AAGGAGCGTTCAGAATGCCGGA | 22 | gp1a |
| gga-miR-6648-3p_L+1_1ss2TA | CACCGGCATTCTGAACGCTCCT | 22 | gp1a |
| gga-miR-6648-3p_L+1_1ss2TA | CACCGGCATTCTGAACGCTCCT | 22 | gp1a |
| gga-miR-6648-3p | TCCGGCATTCTGAACGCTCCT | 21 | gp1a |
| gga-miR-6648-3p | TCCGGCATTCTGAACGCTCCT | 21 | gp1a |
| gga-miR-6649-5p_R-2 | TTTATCACATCAATGCCAAGC | 21 | gp1a |
| gga-mir-6649-p3_1ss2AC | TCCTTGCTGGCAGTGCTG | 18 | gp1a |
| gga-miR-6651-5p_L+3R+1 | CACACCAGGTTGCCTAAGGAGGGGT | 25 | gp1a |
| gga-miR-6655-5p | TCTGCTAGGAGGCTGTGTATCCT | 23 | gp1a |
| gga-mir-6662-p5_1ss2CG | CGAATGGTTGACCAATGGT | 19 | gp1a |
| gga-miR-6663-5p_R+3 | ATTGCTGTGTGAGGAGTGCTGCCT | 24 | gp1a |
| gga-miR-6664-3p_L-3R-1_1ss4GC | CAATGGTGCTGGAGGCTG | 18 | gp1a |
| gga-miR-6665-5p | TGCCTGAAGGATTTTGATGCCC | 22 | gp1a |
| gga-mir-6669-p5 | GCAAGGCTGCATACTTGAAC | 20 | gp1a |
| gga-miR-6669-3p_L-1R+1 | TGCAGCCTGGCCGTATCTCAGT | 22 | gp1a |
| gga-miR-6678-3p_R+4 | TACCTTGGACTTCTGAAGGGCAGACT | 26 | gp1a |
| gga-miR-6680-3p_L-1R-3_1ss17CT | TTGCTGATGAAGATATTG | 18 | gp1a |
| gga-miR-6682-3p_R-4_1ss14GC | TGGAACAAAGTAGCTGGC | 18 | gp1a |
| gga-miR-6685-5p_L+1R+1 | CCAGCAATGCACGTGTACTCAGA | 23 | gp1a |
| gga-miR-6695-5p_L-1R-1 | TGGGAAGGAACAAAGCATGACT | 22 | gp1a |
| gga-miR-6699-5p | TGAAGACCTCGTGCATTGTAGC | 22 | gp1a |
| gga-mir-6700-p5_1ss2GA | AAGGAGCTGGGATTGTTCA | 19 | gp1a |
| gga-mir-6700-p3_1ss2GT | ATCTGAGGGAGCTGGGATT | 19 | gp1a |
| gga-mir-6701-p5 | GTGAAGTGATCTGCCTGCAAAGTACC | 26 | gp1a |
| gga-miR-6701-3p_R+1 | ATTATTTTACAGACAGATCGCT | 22 | gp1a |
| gga-miR-6702-5p_L+1R-2_1ss21TG | TCACCAGGGCAGAGTAGAGGGG | 22 | gp1a |
| gga-miR-6707-5p_L+1R-1 | CACTGGAACAAGCTGCCTGGAGAG | 24 | gp1a |
| gga-miR-7438-5p | TTTTTCAAGGGGACAACTCAGA | 22 | gp1a |
| gga-mir-7439-p5 | AAGAGGCAAAAGGCAAGGGAAAGTT | 25 | gp1a |
| gga-mir-7439-p3 | TTTGCTTCTCCTTCTCCAGTAGTTCC | 26 | gp1a |
| gga-mir-7440-p5 | TTTGAGTGCACCTGGGTGCTACAGG | 25 | gp1a |
| gga-miR-7440-3p_L+1 | TTTTGGTGTAGGCCAGGACAGC | 22 | gp1a |
| gga-miR-7445-5p | AAAGTTAAACAGACTCATCCAC | 22 | gp1a |
| gga-miR-7445-3p_L+1R-2 | TGGATGAGTCTGTTTAACTTT | 21 | gp1a |
| gga-miR-7445-5p | AAAGTTAAACAGACTCATCCAC | 22 | gp1a |
| gga-miR-7445-3p_L+1R-2 | TGGATGAGTCTGTTTAACTTT | 21 | gp1a |
| gga-miR-7449-3p_L-4_1ss18TC | TTGGACGAGATGGCCTTT | 18 | gp1a |
| gga-miR-7452-5p_R+1 | ACAGGGCTGGCTGATCCGTGCA | 22 | gp1a |
| gga-miR-7455-3p_R-2 | CTTCCCTCCGTCGGCGCGCAC | 21 | gp1a |
| gga-miR-7456-5p_L-1R+1_1ss21AG | CTGCAGCTGCATTTTCCCTGTGT | 23 | gp1a |
| gga-miR-7456-3p_R-1 | CAGGGAGATGTACCTGTAGGA | 21 | gp1a |
| gga-miR-7457-5p_R+1 | GGCATTCATAGGGCTGTGATCGGT | 24 | gp1a |
| gga-miR-7459-3p_1ss22GA | TGGGATAGTACTGTATGAGGCAT | 23 | gp1a |
| gga-miR-7462-5p_R+1 | CAACCTCATTTCTAGCTTCTGGT | 23 | gp1a |
| gga-miR-7464-3p_1ss23CT | TGGCACTGCACTGAGTCATTGGT | 23 | gp1a |
| gga-miR-7465-3p | AGAGTGTCTGTTACCTGACC | 20 | gp1a |
| gga-miR-7467-5p_L-1R-2_1ss21GT | CCAGCGCGGTGGTGATGGTT | 20 | gp1a |
| gga-miR-7468-5p_R-4_1ss4CT | TCTTACAGCTCCTTGCAT | 18 | gp1a |
| gga-miR-7468-3p_L+3 | GCTTGATGGAAGATGTTGAGCAGGCC | 26 | gp1a |
| gga-miR-7469-5p | TTTGCAATGCAATTTTACAGTAG | 23 | gp1a |
| gga-miR-7470-5p_R+2 | TGTGATCTTGAAGAACGTGGGCCT | 24 | gp1a |
| gga-miR-7475-5p_L-2_1ss3GA | ACCGCCGCCGCGCCCTCC | 18 | gp1a |
| gga-miR-7477-3p_L+1R-2 | TTACCGCCCCGTTCTGCGCTCG | 22 | gp1a |
| gga-mir-7480-1-p5_1ss13GT | ACGAGACTGAAGTAAGCAGCAATTGC | 26 | gp1a |
| gga-miR-7480-3p_L+2 | TCTTCTCTAAAGCGTGGACAGACA | 24 | gp1a |
| gga-mir-7481-p5 | TGTAGGAGCCAGGAAGAAAGACATT | 25 | gp1a |
| gga-mir-7481-p3 | GGAAGAAAGACATTCAGCTCTGGTCT | 26 | gp1a |
| gga-mir-7482-2-p5 | TTCACTCACCAGAGAGGGCAACTTTC | 26 | gp1a |
| gga-miR-7482-3p_L+1 | TTCTGCAGACACACAAGCCAGAGA | 24 | gp1a |
| gga-miR-7483-5p_R+1 | GCATGGTGTAGTGACGCAGGCAGA | 24 | gp1a |
| rno-miR-9b-5p_R+2 | TTCGGTTATCTAGCTTTATGA | 21 | gp1b |
| rno-miR-9b-3p_R+4 | ATACAGCTAGATAACCAAAGAT | 22 | gp1b |
| hsa-miR-3591-5p_L-1_1ss23AT | TTAGTGTGATAATGGCGTTTGT | 22 | gp1b |
| tgu-miR-7-4-3p | CAACAAATCATAGCCTGCCATA | 22 | gp2 |
| tgu-miR-16b-3p | CCCAATATTATTGTGCTGCTT | 21 | gp2 |
| hsa-miR-31-5p_R+2_1ss11CT | AGGCAAGATGTTGGCATAGCTGA | 23 | gp2 |
| hsa-miR-31-3p_R+1 | TGCTATGCCAACATATTGCCATC | 23 | gp2 |
| hsa-miR-32-3p_R-1_2ss15TC20TC | CAATTTAGTGTGTGCGATACT | 21 | gp2 |
| tgu-miR-92-2-5p_L+2R-1 | GGGTGGGGATTTGTTGCATTACT | 23 | gp2 |
| hsa-miR-96-5p | TTTGGCACTAGCACATTTTTGCT | 23 | gp2 |
| tgu-miR-125-2-3p_R+1 | ACGGGTTAGGCTCTTGGGAGCT | 22 | gp2 |
| hsa-miR-143-5p_R-1 | GGTGCAGTGCTGCATCTCTGG | 21 | gp2 |
| hsa-miR-143-3p_R+1 | TGAGATGAAGCACTGTAGCTCT | 22 | gp2 |
| tgu-miR-155-3p_1ss9CT | CTCCTACATGTTAGCATTAACA | 22 | gp2 |
| rno-miR-190b-5p_R+1 | TGATATGTTTGATATTAGGTTG | 22 | gp2 |
| oha-miR-190b-3p_R+2 | ACTAAATATCAAACATATTCTT | 22 | gp2 |
| rno-miR-190b-5p_R-1 | TGATATGTTTGATATTAGGT | 20 | gp2 |
| tgu-miR-194-3p | CCAGTGGAGATGCTGTTACTTT | 22 | gp2 |
| rno-miR-196c-5p_R-1 | TAGGTAGTTTCGTGTTGTTGG | 21 | gp2 |
| rno-miR-196c-5p_R-1 | TAGGTAGTTTCGTGTTGTTGG | 21 | gp2 |
| tgu-miR-203-5p | AGTGGTTCTTAACAGTTCAAC | 21 | gp2 |
| oan-miR-204-3p_L+1R+2_1ss21AC | GCAGGGACAGCAAAGGGATGCTC | 23 | gp2 |
| pma-mir-204-p5 | TTCCCTTTGTCATCCTATGCCTGT | 24 | gp2 |
| hsa-miR-219b-5p_L-1_2ss11CA21CT | GATGTCCAGACACAATTCTTG | 21 | gp2 |
| tgu-miR-222-5p | CGCTCAGTAGTCAGTGTAGATT | 22 | gp2 |
| aca-miR-375-5p_2ss16CT17AG | GCGTCGAGCCCCACGTGCAAGA | 22 | gp2 |
| mdo-miR-449c-5p_R+2_1ss1GA | AGGCAGTGTATTGTTAGTTAGCT | 23 | gp2 |
| efu-mir-574-1-p3_1ss1GA | ACCCTGTGGGGGTGTGGG | 18 | gp2 |
| efu-mir-574-1-p3_1ss1GA | ACCCTGTGGGGGTGTGGG | 18 | gp2 |
| tgu-miR-1784-3p_R-1_1ss11GA | TGACTTAAATAGGAGCAGAAT | 21 | gp2 |
| tgu-miR-1803-5p_R+1 | AGAGCCCATTGTAGTCAATGGA | 22 | gp2 |
| tgu-mir-2974-p3 | CGGCTCGGCTCGGCTCGGC | 19 | gp2 |
| tgu-miR-2984-5p_R+1 | AGCCTGCTGAGAGTGAAATTGA | 22 | gp2 |
| hsa-miR-3120-5p_L-1R+3 | CTGTCTGTGCCTGCTGTACAGGT | 23 | gp2 |
| hsa-miR-3120-3p | CACAGCAAGTGTAGACAGGCA | 21 | gp2 |
| hsa-miR-3529-3p_L-3R-1 | AACAAAATCACTAGTCTTCC | 20 | gp2 |
| hsa-miR-3529-3p_L-3R-1 | AACAAAATCACTAGTCTTCC | 20 | gp2 |
| hsa-miR-3620-5p_R-2_1ss11CG | GTGGGCTGGGGTGGGCTGGG | 20 | gp2 |
| hsa-miR-3620-5p_R-2_1ss11CG | GTGGGCTGGGGTGGGCTGGG | 20 | gp2 |
| hsa-mir-3665-p3_1ss17AC | GGGCGGCGGCGGCGGCCGC | 19 | gp2 |
| hsa-mir-3665-p5_1ss17AC | GGGCGGCGGCGGCGGCCG | 18 | gp2 |
| mmu-mir-6240-p3 | TTTCTGCCCAGTGCTCTG | 18 | gp2 |
| hsa-mir-7975-p5_1ss10GA | GAGCAGGAGAACAGGGGAT | 19 | gp2 |
| efu-mir-9203b-p5_1ss7AT | CATGACTCCCACCAGCCAGTC | 21 | gp2 |
| tgu-let-7i-3p | CTGCGCAAGCTACTGCCTTGCT | 22 | gp2 |
| odi-miR-7_R+2 | TGGAAGACTAGTGATTTTGTTGAA | 24 | gp3 |
| bbe-miR-9-5p_R+2 | TCTTTGGTTATCTAGCTGTATGAAA | 25 | gp3 |
| oha-miR-9-3p_L-2R+1 | ATACAGCTAGATAACCAAAGAC | 22 | gp3 |
| aca-miR-10c-5p_R-1_1ss12AC | TACCCTGTAGACTCGAATTTGT | 22 | gp3 |
| aca-miR-10c-3p_R+2_1ss1AG | GCAAATTCGTCTCTAGGGGAAT | 22 | gp3 |
| bbe-miR-10b-5p_L-1R-2_1ss9GA | ACCCTGTAGATCCGATCTTGT | 21 | gp3 |
| pma-miR-10a_1ss9GA | TACCCTGTAGACTCGAATTTGT | 22 | gp3 |
| xtr-miR-10c_L-1_1ss12AC | ACCCTGTAGACTCGAATTTGT | 21 | gp3 |
| oan-miR-15a-5p_R+2 | TAGCAGCACATAATGGTTTGTGT | 23 | gp3 |
| oan-miR-15a-3p_1ss22AT | CAGGCCATATTGTGCTGCCTCT | 22 | gp3 |
| chi-miR-16b-5p_R+1 | TAGCAGCACGTAAATATTGGGGA | 23 | gp3 |
| chi-miR-16b-3p_R+1_1ss1AC | CCCAATATTATTGTGCTGCTTTT | 23 | gp3 |
| ccr-miR-17-5p_R+2 | CAAAGTGCTTACAGTGCAGGTAGAA | 25 | gp3 |
| dre-miR-17a-5p_R+3 | CAAAGTGCTTACAGTGCAGGTAGAT | 25 | gp3 |
| aca-miR-18a-5p_R-2 | TAAGGTGCATCTAGTGCAGAT | 21 | gp3 |
| aca-miR-18a-3p_R-1 | ACTGCCCTAAGTGCTCCTTCT | 21 | gp3 |
| oan-miR-19b-1-5p_R+4 | AGTTTTGCAGGTTTGCATCCCAGC | 24 | gp3 |
| oan-miR-19b-3p | TGTGCAAATCCATGCAAAACTGA | 23 | gp3 |
| aca-miR-19b_R+2 | TGTGCAAATCCATGCAAAACTGA | 23 | gp3 |
| ssa-miR-19a-3p_R+1_1ss11TC | TGTGCAAATCCATGCAAAACTGT | 23 | gp3 |
| aca-miR-20a-5p_R+1 | TAAAGTGCTTATAGTGCAGGTAG | 23 | gp3 |
| ssa-miR-20a-5p | TAAAGTGCTTATAGTGCAGGTAG | 23 | gp3 |
| mdo-miR-22-3p_1ss21GC | AAGCTGCCAGTTGAAGAACTCC | 22 | gp3 |
| aca-miR-23a-5p_2ss12TC17TC | GGGGTTCCTGGCGATGCGATTT | 22 | gp3 |
| aca-miR-23a-3p_R+2 | ATCACATTGCCAGGGATTTCCAT | 23 | gp3 |
| dre-miR-24_R+2 | TGGCTCAGTTCAGCAGGAACAGTT | 24 | gp3 |
| bta-miR-25 | CATTGCACTTGTCTCGGTCTGA | 22 | gp3 |
| ola-miR-26_R+4 | TTCAAGTAATCCAGGATAGGCTTTT | 25 | gp3 |
| aca-miR-27a-3p | TTCACAGTGGCTAAGTTCCGC | 21 | gp3 |
| aca-miR-27a-3p | TTCACAGTGGCTAAGTTCCGC | 21 | gp3 |
| dre-miR-27a-3p_R-1 | TTCACAGTGGCTAAGTTCCGC | 21 | gp3 |
| bta-miR-28 | AAGGAGCTCACAGTCTATTGAG | 22 | gp3 |
| hsa-miR-28-3p_R+1_1ss11TA | CACTAGATTGAGAGCTCCTGGAG | 23 | gp3 |
| cgr-miR-28-5p_R+1 | AAGGAGCTCACAGTCTATTGAG | 22 | gp3 |
| cgr-miR-28-3p_L-1R+1 | ACTAGATTGTGAGCTCCTGGAG | 22 | gp3 |
| xtr-miR-29a_R+4 | TAGCACCATTTGAAATCGGTTAAAA | 25 | gp3 |
| oha-miR-30b-5p_R+3 | TGTAAACATCCTACACTCAGCTTTT | 25 | gp3 |
| ipu-miR-30d_1ss9TC | CTTTCAGTCGGATGTTTGCTGT | 22 | gp3 |
| aca-miR-31-5p_R+3 | AGGCAAGATGTTGGCATAGCTGAA | 24 | gp3 |
| aca-miR-31-5p_R+3 | AGGCAAGATGTTGGCATAGCTGAA | 24 | gp3 |
| eca-miR-32_R+2 | TATTGCACATTACTAAGTTGCACC | 24 | gp3 |
| ssa-miR-33a-5p_R+2 | GTGCATTGTAGTTGCATTGCAAT | 23 | gp3 |
| mdo-miR-34b-5p_R+2 | AGGCAGTGTAGTTAGCTGATTGTTT | 25 | gp3 |
| mdo-miR-34b-3p | AATCACTAACTCCACTGCCATC | 22 | gp3 |
| mdo-miR-34b-5p_R+2 | AGGCAGTGTAGTTAGCTGATTGTTT | 25 | gp3 |
| mdo-miR-34b-3p | AATCACTAACTCCACTGCCATC | 22 | gp3 |
| oan-miR-34a-5p_R+1_1ss23CA | AGGCAGTGTAGTTAGCTGATTGAT | 24 | gp3 |
| bbe-miR-71-5p_R+1 | TGAAAGACATGGGTAGTGAGATT | 23 | gp3 |
| dre-miR-92a-3p_R+2 | TATTGCACTTGTCCCGGCCTGTAT | 24 | gp3 |
| cin-miR-92c-3p_1ss18TC | TATTGCACTCGTCCCGGCCTAT | 22 | gp3 |
| age-miR-93_L+1 | CAAAGTGCTGTTCGTGCAGGTAG | 23 | gp3 |
| hsa-miR-93-3p_R+1 | ACTGCTGAGCTAGCACTTCCCGA | 23 | gp3 |
| ccr-miR-99_R+2_1ss1AG | GACCCGTAGATCCGATCTTGTGA | 23 | gp3 |
| aca-miR-99b-5p_1ss23GT | AACCCGTAGATCCGAACTTGCGT | 23 | gp3 |
| cfa-miR-99b | CACCCGTAGAACCGACCTTGCG | 22 | gp3 |
| ssa-miR-99-5p_R+2 | AACCCGTAGATCCGATCTTGTGAA | 24 | gp3 |
| cin-miR-101_L+1R-2_1ss18AC | GTACAGTACTGTGATAACTAT | 21 | gp3 |
| dre-miR-103_R+2 | AGCAGCATTGTACAGGGCTATGAGT | 25 | gp3 |
| bta-miR-106b_R+1 | TAAAGTGCTGACAGTGCAGATA | 22 | gp3 |
| hsa-miR-106b-3p_L+2R-2 | TACCGCACTGTGGGTACTTGCT | 22 | gp3 |
| cgr-miR-106b-5p | TAAAGTGCTGACAGTGCAGATA | 22 | gp3 |
| cgr-miR-106b-3p_L+2R-2 | TACCGCACTGTGGGTACTTGCT | 22 | gp3 |
| cin-miR-124-3p_R+2 | TAAGGCACGCGGTGAATGCCAATT | 24 | gp3 |
| oha-miR-124-4-3p_L+1R-1_1ss22AT | TTAAGGCACGCGGTGAATGCTT | 22 | gp3 |
| sha-miR-125a_R+2 | TCCCTGAGACCCTAACTTGTGAAA | 24 | gp3 |
| ssa-miR-125a-5p_R+2 | TCCCTGAGACCCTAACTTGTGAAT | 24 | gp3 |
| cgr-miR-125a-5p_R-2 | TCCCTGAGACCCTTTAACCTGT | 22 | gp3 |
| cin-miR-125-5p_R-3_1ss14AT | TCCCTGAGACCCTTAAAC | 18 | gp3 |
| ola-miR-126-5p_L+3 | ATCCATTATTACTTTTGGTACGCG | 24 | gp3 |
| ola-miR-126-3p_R+3 | TCGTACCGTGAGTAATAATGCAA | 23 | gp3 |
| ola-miR-126-5p_L+3 | ATCCATTATTACTTTTGGTACGCG | 24 | gp3 |
| ola-miR-126-3p_R+3 | TCGTACCGTGAGTAATAATGCAA | 23 | gp3 |
| hsa-miR-127-5p_R+1 | CTGAAGCTCAGAGGGCTCTGATT | 23 | gp3 |
| bta-miR-127 | TCGGATCCGTCTGAGCTTGGCT | 22 | gp3 |
| xtr-miR-128_R+2 | TCACAGTGAACCGGTCTCTTTTAT | 24 | gp3 |
| aca-miR-129b-5p_R+1_1ss20TG | CTTTTTGCGGTCTGGGCTTGC | 21 | gp3 |
| aca-miR-129b-3p_R+1 | AAGCCCTTACCCCAAAAAGCAT | 22 | gp3 |
| aca-miR-129b-5p_R+1_1ss20TG | CTTTTTGCGGTCTGGGCTTGC | 21 | gp3 |
| aca-miR-129b-3p_R+1 | AAGCCCTTACCCCAAAAAGCAT | 22 | gp3 |
| cgr-miR-130a-5p | GCTCTTTTCACATTGTGCTACT | 22 | gp3 |
| cgr-miR-130a-3p_R+2 | CAGTGCAATGTTAAAAGGGCATCA | 24 | gp3 |
| cgr-miR-130a-5p | GCTCTTTTCACATTGTGCTACT | 22 | gp3 |
| cgr-miR-130a-3p_R+2 | CAGTGCAATGTTAAAAGGGCATCA | 24 | gp3 |
| cgr-miR-130b-3p | CAGTGCAATGATGAAAGGGCAT | 22 | gp3 |
| aca-miR-132-5p_R+2 | ACCGTGGCTTTAGATTGTTACT | 22 | gp3 |
| aca-miR-132-3p_1ss21TC | TAACAGTCTACAGCCATGGTCG | 22 | gp3 |
| xtr-miR-133b_L+1R+2_1ss22AT | TTTGGTCCCCTTCAACCAGCTTTT | 24 | gp3 |
| cfa-miR-134 | TGTGACTGGTTGACCAGAGGGG | 22 | gp3 |
| bta-miR-136_R-1 | ACTCCATTTGTTTTGATGATGG | 22 | gp3 |
| hsa-miR-136-3p_L-1 | ATCATCGTCTCAAATGAGTCT | 21 | gp3 |
| aca-miR-137b-3p_R+1_1ss9TG | TTATTGCTGGAGAATACGCGTAGT | 24 | gp3 |
| aca-miR-138-5p_R+4_2 | AGCTGGTGTTGTGAATCAGGCCGTTT | 26 | gp3 |
| aca-miR-138-2-3p_L-2R+3 | GCTACTTCACAACACCAGGGTTT | 23 | gp3 |
| aca-miR-138-5p_R+4_1 | AGCTGGTGTTGTGAATCAGGCCGATA | 26 | gp3 |
| aca-miR-138-1-3p_R+2 | GCTATTTCACTACACCAGGGTCT | 23 | gp3 |
| bta-miR-139 | TCTACAGTGCACGTGTCTCCAGT | 23 | gp3 |
| tgu-miR-139-3p_R-2_2ss4AG7TC | TGGGGACGCGGCCCTGTTGGA | 21 | gp3 |
| eca-miR-139-5p_R+1 | TCTACAGTGCACGTGTCTCCAGT | 23 | gp3 |
| eca-miR-139-3p_L+1R-2_1ss4AG | TGGGGACGCGGCCCTGTTGGA | 21 | gp3 |
| ggo-miR-140_R+1_1ss22CA | ACCACAGGGTAGAACCACGGAAA | 23 | gp3 |
| hsa-miR-140-3p_L-1R+3 | ACCACAGGGTAGAACCACGGAAA | 23 | gp3 |
| oan-miR-142-5p_R+1_1ss1CN | NATAAAGTAGAAAGCACTACTT | 22 | gp3 |
| oan-miR-142-5p_R+1_1ss1CN | NATAAAGTAGAAAGCACTACTT | 22 | gp3 |
| hsa-miR-143-5p_R-1_1ss8TA | GGTGCAGAGCTGCATCTCTGG | 21 | gp3 |
| cgr-miR-143_R+2 | TGAGATGAAGCACTGTAGCTCAT | 23 | gp3 |
| pma-miR-145-5p_L+3 | ATCGTCCAGTTTTCCCAGGAATCCCT | 26 | gp3 |
| xtr-miR-146b_R+3 | TGAGAACTGAATTCCATGGACTGTA | 25 | gp3 |
| aca-miR-146a-5p_R+3_1ss18AG | TGAGAACTGAATTCCATGGGCTTT | 24 | gp3 |
| cgr-miR-146b-5p_R+1 | TGAGAACTGAATTCCATAGGCTGT | 24 | gp3 |
| aca-miR-148b-3p_1ss20TG | TCAGTGCATCACAGAACTTGGT | 22 | gp3 |
| ggo-miR-148b_R+1_1ss20TG | TCAGTGCATCACAGAACTTGGT | 22 | gp3 |
| ggo-miR-149_R-2 | TCTGGCTCCGTGTCTTCACTC | 21 | gp3 |
| aca-miR-150-5p_R-1_1ss12TC | TCTCCCAACCCCTGTACCAGT | 21 | gp3 |
| cgr-miR-151-5p | TCGAGGAGCTCACAGTCTAGT | 21 | gp3 |
| cgr-miR-151-3p_R+1 | CTAGACTGAGGCTCCTTGAGGA | 22 | gp3 |
| cgr-miR-151-5p | TCGAGGAGCTCACAGTCTAGT | 21 | gp3 |
| cgr-miR-151-3p_R+1 | CTAGACTGAGGCTCCTTGAGGA | 22 | gp3 |
| aca-miR-153-5p_L+1 | GTCATTTTTGTGATTTGCAGCT | 22 | gp3 |
| aca-miR-153-3p_R+5 | TTGCATAGTCACAAAAGTGATCGTTT | 26 | gp3 |
| cgr-miR-154-3p | AATCATACACGGTTGACCTATT | 22 | gp3 |
| bta-miR-154c_1ss5AC | AGATCTTGCACGGTTGATCTCT | 22 | gp3 |
| aca-miR-181a_R+3 | AACATTCAACGCTGTCGGTGAGTTAT | 26 | gp3 |
| aca-miR-181b_R+1_1ss12CT | AACATTCATTGTTGTCGGTGGGA | 23 | gp3 |
| pma-miR-181a-5p_R+3 | AACATTCAACGCTGTCGGTGAGTTAT | 26 | gp3 |
| cgr-miR-182_R+2 | TTTGGCAATGGTAGAACTCACACCGT | 26 | gp3 |
| bta-miR-183_R-1 | TATGGCACTGGTAGAATTCACT | 22 | gp3 |
| aca-miR-184-3p_R+3 | TGGACGGAGAACTGATAAGGGTTTT | 25 | gp3 |
| bta-miR-185_R+1 | TGGAGAGAAAGGCAGTTCCTGAA | 23 | gp3 |
| cgr-miR-185-5p_R+1 | TGGAGAGAAAGGCAGTTCCTGAA | 23 | gp3 |
| bta-miR-186_R+1 | CAAAGAATTCTCCTTTTGGGCTT | 23 | gp3 |
| ccr-miR-187_1ss22GT | TCGTGTCTTGTGTTGCAGCCATT | 23 | gp3 |
| ptr-miR-188_R-2 | CATCCCTTGCATGGTGGAGG | 20 | gp3 |
| tni-miR-190_L+3 | ATCTGATATGTTTGATATATTAGGT | 25 | gp3 |
| aca-miR-191-5p_R-1 | CAACGGAATCCCAAAAGCAGCT | 22 | gp3 |
| aca-miR-191-5p_R-1 | CAACGGAATCCCAAAAGCAGCT | 22 | gp3 |
| cfa-miR-192 | CTGACCTATGAATTGACAGCC | 21 | gp3 |
| aca-miR-193-5p_R+1 | TGGGTCTTTGCGGGCGAGATGA | 22 | gp3 |
| aca-miR-193-3p_R+2 | AACTGGCCTACAAAGTCCCAGT | 22 | gp3 |
| aca-miR-193-5p_R+1 | TGGGTCTTTGCGGGCGAGATGA | 22 | gp3 |
| aca-miR-193-3p_R+2 | AACTGGCCTACAAAGTCCCAGT | 22 | gp3 |
| aca-miR-194-5p_R+2 | TGTAACAGCAACTCCATGTGGAAA | 24 | gp3 |
| aca-miR-194-5p_R+2 | TGTAACAGCAACTCCATGTGGAAA | 24 | gp3 |
| tni-miR-199_R+2 | CCCAGTGTTCAGACTACCTGTTCTT | 25 | gp3 |
| bta-miR-199c_R+3 | TACAGTAGTCTGCACATTGGCTT | 23 | gp3 |
| bta-miR-200c | TAATACTGCCGGGTAATGATGGA | 23 | gp3 |
| hsa-miR-200b-3p_R+2 | TAATACTGCCTGGTAATGATGACA | 24 | gp3 |
| mmu-miR-202-5p_R+2 | TTCCTATGCATATACTTCTTTAT | 23 | gp3 |
| rno-miR-202-5p_R+4 | TTCCTATGCATATACTTCTTTAT | 23 | gp3 |
| aca-miR-203-3p_L-1R+2 | TGAAATGTTTAGGACCACTTGTT | 23 | gp3 |
| aca-miR-203-3p_L-1R+2 | TGAAATGTTTAGGACCACTTGTT | 23 | gp3 |
| pma-miR-204-5p_R+2 | TTCCCTTTGTCATCCTATGCCTTT | 24 | gp3 |
| dre-miR-205-5p_R+3 | TCCTTCATTCCACCGGAGTCTGAGT | 25 | gp3 |
| bbe-miR-210-5p_1ss22AT | CTGTGCGTGTGACAGCGGCTGT | 22 | gp3 |
| cgr-miR-210-3p_1ss22AT | CTGTGCGTGTGACAGCGGCTGT | 22 | gp3 |
| aca-miR-212-5p_R+1 | ACCTTGGCTCTAGACTGCTTACT | 23 | gp3 |
| aca-miR-212-3p_R-2_1ss9CT | TAACAGTCTACAGTCATGGCT | 21 | gp3 |
| aca-miR-212-5p_R+1 | ACCTTGGCTCTAGACTGCTTACT | 23 | gp3 |
| aca-miR-212-3p_R-2_1ss9CT | TAACAGTCTACAGTCATGGCT | 21 | gp3 |
| hsa-miR-212-5p | ACCTTGGCTCTAGACTGCTTACT | 23 | gp3 |
| bta-miR-216a_R+2_1ss22AC | TAATCTCAGCTGGCAACTGTGCGA | 24 | gp3 |
| hsa-miR-216b-3p_L-2_2ss12CT24AT | ACACTTACCTGTAGAGATTCTT | 22 | gp3 |
| fru-miR-218a_L+3R-1 | ATCTTGTGCTTGATCTAACCATGT | 24 | gp3 |
| ssa-miR-219c-5p_R+3 | TGATTGTCCAAACGCAATTCTTGAGT | 26 | gp3 |
| hsa-miR-219a-5p_R+5 | TGATTGTCCAAACGCAATTCTTGAGT | 26 | gp3 |
| hsa-miR-219b-3p_R+1 | AGAATTGCGTTTGGACAATCAGTT | 24 | gp3 |
| aca-miR-221-5p_R-1_1ss22TA | AACCTGGCATACAATGTAGAAA | 22 | gp3 |
| aca-miR-221-3p_R+2 | AGCTACATTGTCTGCTGGGTTTTA | 24 | gp3 |
| xtr-miR-221_R+2 | AGCTACATTGTCTGCTGGGTTTCTT | 25 | gp3 |
| age-miR-222_R+2 | AGCTACATCTGGCTACTGGGTCTCCT | 26 | gp3 |
| dre-miR-223_L-1R+4 | GTCAGTTTGTCAAATACCCCAATA | 24 | gp3 |
| bta-miR-224_R+1 | CAAGTCACTAGTGGTTCCGTTTAG | 24 | gp3 |
| bbe-miR-242-5p_L+1R-2_1ss21CG | TTGCGTAGGCGTTGTGCACAGT | 22 | gp3 |
| bbe-miR-252a-5p_R-2 | CTAAGTACTAGTGCCGCAGGT | 21 | gp3 |
| cgr-miR-296_R+2 | GAGGGTTGGGTGGAGGCTCTCC | 22 | gp3 |
| cgr-miR-298-5p | GGCAGAGGAGGGCTGTTCTTC | 21 | gp3 |
| bta-miR-299_L+1R-1 | ATGGTTTACCGTCCCACATACA | 22 | gp3 |
| hsa-miR-299-5p_L+1R-1 | ATGGTTTACCGTCCCACATACA | 22 | gp3 |
| mmu-miR-300-3p | TATGCAAGGGCAAGCTCTCTTC | 22 | gp3 |
| cgr-miR-300 | TATGCAAGGGCAAGCTCTCTTC | 22 | gp3 |
| aca-miR-301a-5p_1ss11CT | GCTCTGACTTTATTGCACTACT | 22 | gp3 |
| aca-miR-301a-3p | CAGTGCAATAGTATTGTCAAAGC | 23 | gp3 |
| pma-miR-315_R-1_1ss1AT | TTTTGATTGTTGCTCAGAAAGCC | 23 | gp3 |
| bta-miR-320a | AAAAGCTGGGTTGAGAGGGCGA | 22 | gp3 |
| mmu-miR-322-5p_R-1_1ss21GA | CAGCAGCAATTCATGTTTTGA | 21 | gp3 |
| mmu-miR-322-3p_R-1 | AAACATGAAGCGCTGCAACA | 20 | gp3 |
| cfa-miR-323 | CACATTACACGGTCGACCTCT | 21 | gp3 |
| hsa-miR-324-3p_R+1 | ACTGCCCCAGGTGCTGCTGGT | 21 | gp3 |
| cgr-miR-324-3p_L-2R+1 | ACTGCCCCAGGTGCTGCTGGT | 21 | gp3 |
| mmu-miR-325-3p_R-1 | TTTATTGAGCACCTCCTATCA | 21 | gp3 |
| rno-miR-326-3p | CCTCTGGGCCCTTCCTCCAGT | 21 | gp3 |
| mmu-miR-329-5p_R+1 | AGAGGTTTTCTGGGTCTCTGTTT | 23 | gp3 |
| mmu-miR-329-3p_R-1 | AACACACCCAGCTAACCTTTT | 21 | gp3 |
| aja-miR-331_L-1R+1 | CCCCTGGGCCTATCCTAGAAT | 21 | gp3 |
| bta-miR-335 | TCAAGAGCAATAACGAAAAATGT | 23 | gp3 |
| hsa-miR-335-3p | TTTTTCATTATTGCTCCTGACC | 22 | gp3 |
| mmu-miR-337-5p | CGGCGTCATGCAGGAGTTGATT | 22 | gp3 |
| mmu-miR-337-3p_L+1R-1 | TTCAGCTCCTATATGATGCCTT | 22 | gp3 |
| aca-miR-338-5p_R+4 | AACAATATCCTGGTGCTGAGTGAGAG | 26 | gp3 |
| aca-miR-338-3p_R+4 | TCCAGCATCAGTGATTTTGTTGAAT | 25 | gp3 |
| tgu-miR-338-3p_R+1_1ss22GT | TCCAGCATCAGTGATTTTGTTTT | 23 | gp3 |
| mmu-miR-339-5p_R+1 | TCCCTGTCCTCCAGGAGCTCACGA | 24 | gp3 |
| mmu-miR-339-3p_R-2 | TGAGCGCCTCGGCGACAGAGC | 21 | gp3 |
| mmu-miR-339-5p_R+1 | TCCCTGTCCTCCAGGAGCTCACGA | 24 | gp3 |
| mmu-miR-339-3p_R-2 | TGAGCGCCTCGGCGACAGAGC | 21 | gp3 |
| cfa-miR-340 | TTATAAAGCAATGAGACTGATT | 22 | gp3 |
| hsa-miR-340-3p | TCCGTCTCAGTTACTTTATAGC | 22 | gp3 |
| hsa-miR-340-5p | TTATAAAGCAATGAGACTGATT | 22 | gp3 |
| bta-miR-340_R-1 | TCCGTCTCAGTTACTTTATAGC | 22 | gp3 |
| bta-miR-342_R-2 | TCTCACACAGAAATCGCACCCAT | 23 | gp3 |
| mmu-miR-345-5p_L+1 | TGCTGACCCCTAGTCCAGTGCTT | 23 | gp3 |
| mmu-miR-351-5p | TCCCTGAGGAGCCCTTTGAGCCTG | 24 | gp3 |
| efu-miR-361 | TTATCAGAATCTCCAGGGGTAC | 22 | gp3 |
| hsa-miR-361-3p_R-1 | TCCCCCAGGTGTGATTCTGATT | 22 | gp3 |
| chi-miR-361-5p | TTATCAGAATCTCCAGGGGTAC | 22 | gp3 |
| chi-miR-361-3p | TCCCCCAGGTGTGATTCTGATT | 22 | gp3 |
| cgr-miR-362_R-3 | AATCCTTGGAACCTAGGTGTGAG | 23 | gp3 |
| hsa-miR-362-3p_R-1_1ss10AG | AACACACCTGTTCAAGGATTC | 21 | gp3 |
| mmu-miR-362-5p_R-1_1ss23AG | AATCCTTGGAACCTAGGTGTGAG | 23 | gp3 |
| mmu-miR-362-3p_R-1 | AACACACCTGTTCAAGGATTC | 21 | gp3 |
| aca-miR-363-5p | GTGGATCACGATGCAATTTTGA | 22 | gp3 |
| aca-miR-363-3p_R+1 | AATTGCACGGTATCCATCTGTA | 22 | gp3 |
| oan-miR-363-3p_R+1 | AATTGCACGGTATCCATCTGTA | 22 | gp3 |
| ssc-miR-369 | AATAATACATGGTTGATCTTT | 21 | gp3 |
| efu-miR-370_L-1R+2 | CCTGCTGGGGTGGAACCTGGTAT | 23 | gp3 |
| bta-miR-374a_R+1 | TTATAATACAACCTGATAAGTGT | 23 | gp3 |
| hsa-miR-374a-5p_R+1 | TTATAATACAACCTGATAAGTGT | 23 | gp3 |
| bbe-miR-375-3p_R+1 | TTTGTTCGTTCGGCTCGCGTTATA | 24 | gp3 |
| mmu-miR-376b-3p | ATCATAGAGGAACATCCACTT | 21 | gp3 |
| mmu-miR-376b-3p | ATCATAGAGGAACATCCACTT | 21 | gp3 |
| mmu-miR-376c-3p | AACATAGAGGAAATTTCACGT | 21 | gp3 |
| chi-miR-376a_L+1R+1 | GGTAGATTCTCCTTCTATGAGTA | 23 | gp3 |
| hsa-miR-378a-5p_L-1 | TCCTGACTCCAGGTCCTGTGT | 21 | gp3 |
| ssc-miR-378 | ACTGGACTTGGAGTCAGAAGGC | 22 | gp3 |
| bta-miR-379 | TGGTAGACTATGGAACGTAGG | 21 | gp3 |
| mmu-miR-380-3p_R-1 | TATGTAGTATGGTCCACATCT | 21 | gp3 |
| hsa-miR-381-5p_R+1 | AGCGAGGTTGCCCTTTGTATATT | 23 | gp3 |
| bta-miR-381 | TATACAAGGGCAAGCTCTCTGT | 22 | gp3 |
| hsa-miR-381-5p_R+1 | AGCGAGGTTGCCCTTTGTATATT | 23 | gp3 |
| hsa-miR-381-3p | TATACAAGGGCAAGCTCTCTGT | 22 | gp3 |
| hsa-miR-382-3p_R+1 | AATCATTCACGGACAACACTTT | 22 | gp3 |
| cfa-miR-382 | AATCATTCACGGACAACACTTT | 22 | gp3 |
| xtr-miR-383_R+3 | AGATCAGAAGGTGATTGTGGCTATT | 25 | gp3 |
| cgr-miR-409-5p | AGGTTACCCGAGCAACTTTGCATC | 24 | gp3 |
| cgr-miR-409-3p | GAATGTTGCTCGGTGAACCCCT | 22 | gp3 |
| bta-miR-409a_R+1 | AGGTTACCCGAGCAACTTTGCATC | 24 | gp3 |
| hsa-miR-409-3p | GAATGTTGCTCGGTGAACCCCT | 22 | gp3 |
| bta-miR-410 | AATATAACACAGATGGCCTGT | 21 | gp3 |
| chi-miR-411a-5p_L-1R+1 | TAGTAGACCGTATAGCGTACG | 21 | gp3 |
| chi-miR-411a-3p | TATGTAACACGGTCCACTAAC | 21 | gp3 |
| cfa-miR-411_L-1 | TAGTAGACCGTATAGCGTACG | 21 | gp3 |
| hsa-miR-411-3p_R-1 | TATGTAACACGGTCCACTAAC | 21 | gp3 |
| bta-miR-411c-5p | GGTTGATCAGAGAACATACATT | 22 | gp3 |
| bta-miR-423-5p_R+2 | TGAGGGGCAGAGAGCGAGACTTTTT | 25 | gp3 |
| bta-miR-423-3p_L-1 | AGCTCGGTCTGAGGCCCCTCAGT | 23 | gp3 |
| bta-miR-423-5p_R+2 | TGAGGGGCAGAGAGCGAGACTTTTT | 25 | gp3 |
| bta-miR-423-3p_L-1 | AGCTCGGTCTGAGGCCCCTCAGT | 23 | gp3 |
| aca-miR-425-5p_R+4 | AATGACACGATCACTCCCGCTGAGC | 25 | gp3 |
| aca-miR-425-3p_L-2R+1_1ss3AT | TCGGGGATGTCGTGTCTGTCC | 21 | gp3 |
| bta-miR-425-5p_L+1R+2_1ss20TC | AATGACACGATCACTCCCGCTGAGT | 25 | gp3 |
| cfa-miR-429_R+2 | TAATACTGTCTGGTAATGCCGTTT | 24 | gp3 |
| bta-miR-431_R-3 | TGTCTTGCAGGCCGTCATGC | 20 | gp3 |
| hsa-miR-431-3p_L+3R-1 | TTGCAGGTCGTCTTGCAGGGCTTC | 24 | gp3 |
| hsa-miR-431-5p_R-1 | TGTCTTGCAGGCCGTCATGC | 20 | gp3 |
| hsa-miR-431-3p_L+3R-1 | TTGCAGGTCGTCTTGCAGGGCTTC | 24 | gp3 |
| bta-miR-433 | ATCATGATGGGCTCCTCGGTGT | 22 | gp3 |
| mmu-miR-434-5p | GCTCGACTCATGGTTTGAACCA | 22 | gp3 |
| mmu-miR-434-3p | TTTGAACCATCACTCGACTCCT | 22 | gp3 |
| mdo-miR-449b-3p_L-4_1ss6AG | CGGGACTGCCCTGCCACT | 18 | gp3 |
| cfa-miR-450a_L-1R+1 | TTTTGCGATGTGTTCCTAATAT | 22 | gp3 |
| chi-miR-450-5p_R+2 | TTTTGCGATGTGTTCCTAATAT | 22 | gp3 |
| ssc-miR-451_L+3R-1 | ATCAAACCGTTACCATTACTGAGT | 24 | gp3 |
| sha-miR-454_R+3 | TAGTGCAATATTGCTTATAGGGTTCT | 26 | gp3 |
| aca-miR-456_R+3 | CAGGCTGGTTAGATGGTTGTCTTA | 24 | gp3 |
| ccr-miR-457b_L+1R+2_1ss10AG | TAGCAGCACGTAAATACTGGAGGT | 24 | gp3 |
| mmu-miR-466o-5p_1ss13AG | TGATGTGTGTGTGCATGTACAT | 22 | gp3 |
| mmu-miR-466b-3p_R-1 | ATACATACACGCACACATAAG | 21 | gp3 |
| mmu-miR-466o-5p_1ss13AG | TGATGTGTGTGTGCATGTACAT | 22 | gp3 |
| mmu-miR-466b-3p_R-1 | ATACATACACGCACACATAAG | 21 | gp3 |
| mmu-miR-466i-5p_L+1R+5_1ss15GC | GTGTGTGTGTGTGTCTGTGTGTGTGT | 26 | gp3 |
| rno-miR-466b-4-3p_L-1_1ss6CT | TATATATACACACATACACA | 20 | gp3 |
| mmu-miR-467b-5p_L-1 | TAAGTGCCTGCATGTATATG | 20 | gp3 |
| mmu-miR-467a-3p_L-2R+2 | TATACATACACACACCTACACA | 22 | gp3 |
| mmu-miR-467e-5p | ATAAGTGTGAGCATGTATATGT | 22 | gp3 |
| hsa-miR-483-5p | AAGACGGGAGGAAAGAAGGGAG | 22 | gp3 |
| hsa-miR-483-3p_L-1R+2 | CACTCCTCTCCTCCCGTCTTCT | 22 | gp3 |
| ggo-miR-483 | AAGACGGGAGGAAAGAAGGGAG | 22 | gp3 |
| hsa-miR-483-3p_L-1R+2 | CACTCCTCTCCTCCCGTCTTCT | 22 | gp3 |
| bta-miR-484 | TCAGGCTCAGTCCCCTCCCGAT | 22 | gp3 |
| hsa-miR-485-3p_L+1 | AGTCATACACGGCTCTCCTCTCT | 23 | gp3 |
| chi-miR-485-3p | AGTCATACACGGCTCTCCTCTCT | 23 | gp3 |
| cgr-miR-486-5p | TCCTGTACTGAGCTGCCCCGAG | 22 | gp3 |
| cgr-miR-486-3p_L-1R-2_1ss8GC | GGGGCACCTCAGTACAAGA | 19 | gp3 |
| cgr-miR-486-5p | TCCTGTACTGAGCTGCCCCGAG | 22 | gp3 |
| cgr-miR-486-3p_L-1R-2_1ss8GC | GGGGCACCTCAGTACAAGA | 19 | gp3 |
| bta-miR-487b_L-1R+1 | ATCGTACAGGGTCATCCACTTT | 22 | gp3 |
| hsa-miR-487b-3p_L-1R+1 | ATCGTACAGGGTCATCCACTTT | 22 | gp3 |
| hsa-miR-493-5p | TTGTACATGGTAGGCTTTCATT | 22 | gp3 |
| bta-miR-493 | TGAAGGTCTACTGTGTGCCAGG | 22 | gp3 |
| chi-miR-493-5p | TTGTACATGGTAGGCTTTCATT | 22 | gp3 |
| chi-miR-493-3p | TGAAGGTCTACTGTGTGCCAGG | 22 | gp3 |
| bta-miR-494_R+1 | TGAAACATACACGGGAAACCTCT | 23 | gp3 |
| hsa-miR-494-3p_R+1 | TGAAACATACACGGGAAACCTCT | 23 | gp3 |
| bta-miR-495 | AAACAAACATGGTGCACTTCTT | 22 | gp3 |
| bta-miR-496_L-2R+1 | AGTATTACATGGCCAATCTCC | 21 | gp3 |
| bta-miR-497_R-1 | CAGCAGCACACTGTGGTTTGT | 21 | gp3 |
| oha-miR-499-3p_R+2 | AACATCACTTTAAGTCTGTGCTTT | 24 | gp3 |
| oha-miR-499-3p_R+2 | AACATCACTTTAAGTCTGTGCTTT | 24 | gp3 |
| oan-miR-499-5p_R+4 | TTAAGACTTGTAGTGATGTTTAGCC | 25 | gp3 |
| bta-miR-500 | TAATCCTTGCTACCTGGGTGAGA | 23 | gp3 |
| hsa-miR-500a-3p_R-1 | ATGCACCTGGGCAAGGATTCT | 21 | gp3 |
| hsa-miR-500b-5p_L+1R+4 | TAATCCTTGCTACCTGGGTGAGA | 23 | gp3 |
| mmu-miR-501-5p_R+2 | AATCCTTTGTCCCTGGGTGAAAAT | 24 | gp3 |
| mmu-miR-501-3p_R+1 | AATGCACCCGGGCAAGGATTTGG | 23 | gp3 |
| bta-miR-503-5p_R+3 | TAGCAGCGGGAACAGTACTGCAG | 23 | gp3 |
| bta-miR-503-5p_R+3 | TAGCAGCGGGAACAGTACTGCAG | 23 | gp3 |
| bta-miR-505_L-1R+2 | GTCAACACTTGCTGGTTTCCTCT | 23 | gp3 |
| hsa-miR-505-3p_L-1R+2 | GTCAACACTTGCTGGTTTCCTCT | 23 | gp3 |
| ggo-miR-515-5p_R-2 | TTCTCCAAAAGAAAGCACTTTC | 22 | gp3 |
| ggo-miR-519a | AAAGTGCATCCTTTTAGAGTGT | 22 | gp3 |
| ggo-miR-519a | AAAGTGCATCCTTTTAGAGTGT | 22 | gp3 |
| bta-miR-532 | CATGCCTTGAGTGTAGGACCGT | 22 | gp3 |
| cgr-miR-532-5p | CATGCCTTGAGTGTAGGACCGT | 22 | gp3 |
| mmu-miR-540-3p_R+2 | AGGTCAGAGGTCGATCCTGGGC | 22 | gp3 |
| mmu-miR-541-5p | AAGGGATTCTGATGTTGGTCACACT | 25 | gp3 |
| cgr-miR-542-3p_R+1 | TGTGACAGATTGATAACTGAAAGT | 24 | gp3 |
| hsa-miR-542-3p_R+2 | TGTGACAGATTGATAACTGAAAGT | 24 | gp3 |
| bta-miR-543 | AAACATTCGCGGTGCACTTCTT | 22 | gp3 |
| hsa-miR-548g-3p_L-3R-1_1ss13AG | ACTGTAATTGCTTTTGTA | 18 | gp3 |
| aca-miR-551_R+2 | GCGACCCATACTTGGTTTCAGTA | 23 | gp3 |
| ggo-miR-574 | CACGCTCATGCACACACCCACA | 22 | gp3 |
| cgr-miR-582_R-2 | TAACCTGTTGAACAACTGAAC | 21 | gp3 |
| hsa-miR-636 | TGTGCTTGCTCGTCCCGCCCGCA | 23 | gp3 |
| ppy-miR-651_L-2R-2_1ss3TC | CAGGATAAGTTTGACTTT | 18 | gp3 |
| hsa-miR-652-5p_R-2 | CAACCCTAGGAGAGGGTGCCATT | 23 | gp3 |
| bta-miR-652_R+1 | AATGGCGCCACTAGGGTTGTGT | 22 | gp3 |
| cgr-miR-652-5p_R-2 | CAACCCTAGGAGAGGGTGCCATT | 23 | gp3 |
| cgr-miR-652-3p_R+1 | AATGGCGCCACTAGGGTTGTGT | 22 | gp3 |
| bta-miR-654_R-2 | TATGTCTGCTGACCATCACC | 20 | gp3 |
| hsa-miR-654-3p_R-2 | TATGTCTGCTGACCATCACC | 20 | gp3 |
| bta-miR-660 | TACCCATTGCATATCGGAGCTG | 22 | gp3 |
| mmu-miR-665-3p_R+3 | ACCAGGAGGCTGAGGTCCCTTAC | 23 | gp3 |
| mmu-miR-669e-5p_R+1 | TGTCTTGTGTGTGCATGTTCATT | 23 | gp3 |
| mmu-miR-673-5p_R+1 | CTCACAGCTCTGGTCCTTGGAGC | 23 | gp3 |
| cgr-miR-674 | CACAGCTCCCATCTCAGAAC | 20 | gp3 |
| mmu-miR-676-3p_R-1 | CCGTCCTGAGGTTGTTGAGC | 20 | gp3 |
| mmu-miR-681_L-2R-1_1ss6TG | GCCGCGCTGGCAGGCAGC | 18 | gp3 |
| bta-miR-708 | AAGGAGCTTACAATCTAGCTGGG | 23 | gp3 |
| hsa-miR-708-3p_R+1_1ss3AT | CATCTAGACTGTGAGCTTCTAGA | 23 | gp3 |
| bta-miR-744 | TGCGGGGCTAGGGCTAACAGCA | 22 | gp3 |
| ggo-miR-766_R-1 | ACTCCAGCCCCACAGCCTCAG | 21 | gp3 |
| cgr-miR-872-5p | AAGGTTACTTGTTAGTTCAGG | 21 | gp3 |
| rno-miR-875 | TATACCTCAGTTTTATCAGGTG | 22 | gp3 |
| hsa-miR-921_L-4R-3_1ss14GA | TGAGGGACAAAACCAGGA | 18 | gp3 |
| mmu-miR-1193-3p | TAGGTCACCCGTTTTACTATC | 21 | gp3 |
| cgr-miR-1260_L+1 | AATCCCACCGCTGCCACCA | 19 | gp3 |
| oan-miR-1357_L+3R+1 | AAGATTATGAGATCTGAGGGCCA | 23 | gp3 |
| oan-miR-1386_L+2 | GACTCCTGGCTGGCTCGCCA | 20 | gp3 |
| ola-miR-1388-3p_R+6 | ATCTCAGGTTCGTCAGCCCATGTA | 24 | gp3 |
| oan-miR-1419d-5p_L-2R-1_1ss20CA | AGTGCTGGAGATGTCATA | 18 | gp3 |
| hsa-miR-1469_L-4_1ss10GC | GCGCGCGGCGCGGGCTCC | 18 | gp3 |
| pma-miR-1788b-3p_R-5_1ss10TC | CAGGCAAGGCAAAACAAG | 18 | gp3 |
| aca-miR-1805-3p_R+1_1ss22CA | TGTATTGGAACACTACAGCTCAA | 23 | gp3 |
| bta-miR-1839_R-1_1ss23TA | AAGGTAGATAGAACAGGTCTTGA | 23 | gp3 |
| mmu-miR-1843b-5p_R+1 | ATGGAGGTCTCTGTCTGACTTA | 22 | gp3 |
| mmu-miR-1843b-5p_R+1 | ATGGAGGTCTCTGTCTGACTTA | 22 | gp3 |
| mmu-miR-1957a_R-1_1ss15AT | CAGTGGTAGAGCATTTGA | 18 | gp3 |
| tgu-miR-2131_R-1 | ATGCAGAAGTGCACAGAAACAGC | 23 | gp3 |
| mmu-miR-2137_L-1R-1_1ss16AG | CCGGCGGGAGCCCCGGGGA | 19 | gp3 |
| dre-miR-2184_R-1_1ss14TG | AACAGTAAGAGTTGATGTGC | 20 | gp3 |
| bta-miR-2284x_R+2 | TGAAAAGTTCGTTCGGGTTTTTA | 23 | gp3 |
| bta-miR-2324_L-5_1ss15TA | TTGGGGCAGAGTGGCTGT | 18 | gp3 |
| bta-miR-2407_L-2R-2_1ss9AC | GGGCGGCTGGGAAGGGCT | 18 | gp3 |
| bta-miR-2423_R-3_1ss3GC | TTCTGTGGTTGTTGTTTT | 18 | gp3 |
| bta-miR-2424_L-2R-3_1ss10GT | ATCTTTGTTAATCTGATG | 18 | gp3 |
| bta-miR-2478_1ss2TA | GAATCCCACTTCTGACACCA | 20 | gp3 |
| tgu-miR-2970-5p_1ss21GT | GACAGTCAGCAGTTGGTCTGT | 21 | gp3 |
| tgu-miR-2970-3p_L+1_1ss23TA | CAGATCACCTCTTGGCTGTGGGA | 23 | gp3 |
| tgu-miR-2976_R+3_1ss20GA | GCGGAGCGGAGCGGAGCGGAGCGG | 24 | gp3 |
| tgu-miR-2994_L-3R-1_1ss11AG | CGGGCTGGTCCCCGCTGC | 18 | gp3 |
| tgu-miR-2995_L-1_1ss2GT | TGCACTGTTCGTAACCTGTT | 20 | gp3 |
| mmu-miR-3082-3p_L-1R+1 | ACATGGCACTCAACTCTGCAGA | 22 | gp3 |
| hsa-miR-3156-3p_L-3_1ss5CA | CAACTTCCAGATCTTTCT | 18 | gp3 |
| hsa-miR-3156-3p_L-3_1ss5CA | CAACTTCCAGATCTTTCT | 18 | gp3 |
| hsa-miR-3187-5p_L-2_1ss3TC | CGGGCAGCGTGTGGCTGAAGG | 21 | gp3 |
| eca-miR-3200_R-3 | CACCTTGCGCTACTCAGGTCT | 21 | gp3 |
| bta-miR-3431 | CCTCAGTCAGCCTTGTGGATGT | 22 | gp3 |
| mmu-miR-3569-5p_L-3_1ss14GA | GAGGAGAGCAAACCGGTG | 18 | gp3 |
| hsa-miR-3671_R-4_1ss8AT | ATCAAATTAGGACTAGTC | 18 | gp3 |
| oar-miR-3957-3p_R-1 | ACGCACAGCACCTCACTGAGC | 21 | gp3 |
| mmu-miR-3964_R+1_1ss5GA | ATAAAGTAGAAAGCACTAAAT | 21 | gp3 |
| mmu-miR-3968_1ss14AT | CGAATCCCACTCCTGACACCA | 21 | gp3 |
| mmu-miR-3971_L+1R-1_1ss7CA | CCTCCCAACCCCTGTACCAGTG | 22 | gp3 |
| cin-miR-4016_1ss15AC | TGTTCTGTGATCTTCAAGG | 19 | gp3 |
| cin-miR-4037-5p_R-4_1ss14TA | ACAATTGCACTCGACTGT | 18 | gp3 |
| cin-miR-4171-5p_R-2_1ss15AT | TGACTCTCTTAAGGTAGC | 18 | gp3 |
| hsa-miR-4286_R+1 | ACCCCACTCCTGGTACCA | 18 | gp3 |
| hsa-miR-4454_L+1_1ss3GA | CGAATCCGAGTCACGGCACCA | 21 | gp3 |
| hsa-miR-4459_L-3_1ss9CA | GGAGGAGGAGGAGGTGGAG | 19 | gp3 |
| hsa-miR-4497_L+1 | GCTCCGGGACGGCTGGGC | 18 | gp3 |
| hsa-miR-4508_L+2 | AAGCGGGGCTGGGCGCGCG | 19 | gp3 |
| hsa-miR-4634_R-1_1ss8AC | CGGCGCGCCCGGCCCGGG | 18 | gp3 |
| ggo-miR-4660_R-4_1ss16AG | TGCAGCTCTGGTGGAGAA | 18 | gp3 |
| hsa-miR-4686_L-2R-3_1ss5TC | TCCGCTGGGCTTTCTGGT | 18 | gp3 |
| hsa-miR-4792_1ss9GT | CGGTGAGCTCTCGCTGGC | 18 | gp3 |
| bbe-miR-4860-3p_L-4R-2_1ss17CT | GAGATTGTGTGATGGGTA | 18 | gp3 |
| bfl-miR-4906_L-4R-2_1ss16GA | CCAAATGTGAAAAATGTT | 18 | gp3 |
| mmu-miR-5100_1ss21TC | TCGAATCCCAGCGGTGCCTCC | 21 | gp3 |
| mmu-miR-5106_R-4_1ss1AG | GGGTCTGTAGCTCAGTTGG | 19 | gp3 |
| mml-miR-6134_R+4_1ss18GT | TGAGGTAGTAGGATGTATAGTTA | 23 | gp3 |
| mmu-miR-6238_L-1R-3_1ss12TC | TATTAGTCAGCGGAGGAA | 18 | gp3 |
| mmu-miR-6240_L-1R-1_1ss21AG | CAAAGCATCGCGAAGGCCCGCGGC | 24 | gp3 |
| rno-miR-6332_L-5_1ss22CA | GGACTGCAAGGAGCCGAA | 18 | gp3 |
| mmu-miR-6384_L-2R+1 | TTTCCTACTGTTTCCCTGT | 19 | gp3 |
| mmu-miR-6412_R-2_1ss15AT | TCGAAACCATCCTCTGCTAC | 20 | gp3 |
| mmu-miR-6540-5p_R-4_1ss12AC | CTAAGGCAGGCCGACTTC | 18 | gp3 |
| hsa-miR-6758-5p_L-2R-3_1ss7GT | GAGATGGGAAGGATGTGA | 18 | gp3 |
| mmu-miR-6900-3p_L-3_1ss10GA | TGATGGACTCTCTTGTAG | 18 | gp3 |
| mmu-miR-6937-5p_L-4R-2_1ss6GC | TCTAAGGGCTGGGTCTGT | 18 | gp3 |
| mmu-miR-6946-3p_L-1R-1_1ss12CT | TTCTTCTCTTTCCTTTCA | 18 | gp3 |
| mmu-miR-6996-5p_L-3_1ss7GA | ACAAGACAGAGCACAGTC | 18 | gp3 |
| mmu-miR-6997-5p_L-2R-2_1ss19CG | ACAGGCTGGAGAGGTGGA | 18 | gp3 |
| mmu-miR-7027-5p_R-3_1ss7GT | TGGAAATGAAGAAACAGCAG | 20 | gp3 |
| mmu-miR-7054-5p_L-3R-4_1ss10GC | GAAGGTCGTTGGGCTGAG | 18 | gp3 |
| mmu-miR-7080-5p_L-1R-4_1ss15TA | TAGGAGCTGGAGGAGGGT | 18 | gp3 |
| hsa-miR-7160-5p_L-3R+1 | TGAGGTCCGGGCTGTGCCT | 19 | gp3 |
| mmu-miR-7222-3p_L-2R-3_1ss12GT | CAGGACAGTTGGCAGGAG | 18 | gp3 |
| mdo-miR-7319-3p_L-4_1ss16AG | TTGGACTAGAGGTGGTGT | 18 | gp3 |
| mdo-miR-7398b-5p_R-5_1ss12GA | TGTGTAGAGATAGGAAAT | 18 | gp3 |
| oan-miR-7428-5p_L-4_1ss17CA | TCAGTCATCAGTAAGAGA | 18 | gp3 |
| ipu-miR-7550_1ss1AT | TTCCGGCTCGAAGGACCA | 18 | gp3 |
| hsa-miR-7704_R-1_1ss8GC | CGGGGTCCGCGGCGACGT | 18 | gp3 |
| hsa-miR-7977_1ss6AG | TTCCCGGCCAACGCACCA | 18 | gp3 |
| mmu-miR-8117_L-1R-1_1ss15GA | CTCGTGTGGAACAAAAGGG | 19 | gp3 |
| ssa-miR-8157-3p_L-4_1ss11CG | TGTGCTGTGCTGTGCTGT | 18 | gp3 |
| eca-miR-8971_L-3R-4_1ss19CA | TGAGTGTGTACTGTGAGG | 18 | gp3 |
| eca-miR-9115_L-2R-4_1ss16TA | GTGAGAACTGAAGAGCAG | 18 | gp3 |
| eca-miR-9136_L-4R-2_1ss13CT | GAAGGACATACTTGGGGCT | 19 | gp3 |
| efu-miR-9226_1ss22GA | TCAAGTCCCTGTTCGGGCGCCA | 22 | gp3 |
| efu-miR-9270_L-3R-4_1ss16AG | GAAGGGACGGGCGATGGT | 18 | gp3 |
| efu-miR-9277_L-4R+1 | CGAATCCTGCCGACTACGCC | 20 | gp3 |
| aca-let-7e-5p_R+1 | TGAGGTAGTAGATTGAATAGTTT | 23 | gp3 |
| aca-let-7e-3p_L-3_2ss11AG23TC | CTATACAGTCTACTGTCTTCC | 21 | gp3 |
| aca-let-7d-5p_R+2 | AGAGGTAGTAGGTTGCATAGTTA | 23 | gp3 |
| aca-let-7d-3p_R+2_2ss1CN21CA | NTATACAACCTGCTGCCTTTATT | 23 | gp3 |
| oha-let-7i-5p_R+2 | TGAGGTAGTAGTTTGTGCTGTTCT | 24 | gp3 |
| oha-let-7i-3p_L-1R-1_2ss20GA21CA | TGCGCAAGCTACTGCCTTAAT | 21 | gp3 |
| csa-let-7d_1ss10TC | TGAGGTAGTCGGTTGTATTGTT | 22 | gp3 |
| oan-let-7f-5p_R+1_1ss16TA | TGAGGTAGTAGATTGAATAGTTT | 23 | gp3 |
| mml-let-7a-5p_L+1R+1_1ss13GA | CTGAGGTAGTAGATTGTATAGTTT | 24 | gp3 |
| mmu-let-7j_1ss8TG | TGAGGTAGTAGTTTGTGCTGTTAT | 24 | gp3 |
| hsa-let-7d-5p_R+1 | AGAGGTAGTAGGTTGCATAGTTA | 23 | gp3 |
| cin-let-7b-5p_R-3_1ss18TG | TGAGGTAGTAGGTTATGGT | 19 | gp3 |
| PC-5p-3655_746 | TTCCTCTTTGGGGCCTGGATGCT | 23 | gp4 |
| PC-3p-5076_599 | TTCCTCTTTGGGGCCTGGATGCTGC | 25 | gp4 |
| PC-5p-3655_746 | TTCCTCTTTGGGGCCTGGATGCT | 23 | gp4 |
| PC-3p-5076_599 | TTCCTCTTTGGGGCCTGGATGCTGC | 25 | gp4 |
| PC-5p-1122164_4 | GCAGCGGTGCGGCTGTGCTTGTGAC | 25 | gp4 |
| PC-3p-29351_153 | TCAGAGTAGAGGCAGCGCTGCGGC | 24 | gp4 |
| PC-5p-1122164_4 | GCAGCGGTGCGGCTGTGCTTGTGAC | 25 | gp4 |
| PC-3p-19523_219 | TGTCAGAGTAGAGGCAGCGCTGCGGC | 26 | gp4 |
| PC-5p-1065108_4 | TCCCGAGGGACCTGGGCATT | 20 | gp4 |
| PC-3p-111583_41 | TCCCGAGGGACCTGGGCACTTGCTCT | 26 | gp4 |
| PC-5p-187229_23 | TCCCGAGGGACCTGGGCACTTGA | 23 | gp4 |
| PC-3p-111583_41 | TCCCGAGGGACCTGGGCACTTGCTCT | 26 | gp4 |
| PC-5p-20326_212 | TTTTGAGGAAGATACGCGTGATA | 23 | gp4 |
| PC-3p-35214_130 | TCCATGTGTTCATCAGGGAGAGGA | 24 | gp4 |
| PC-5p-20326_212 | TTTTGAGGAAGATACGCGTGATA | 23 | gp4 |
| PC-3p-35214_130 | TCCATGTGTTCATCAGGGAGAGGA | 24 | gp4 |
| PC-5p-389334_11 | GCGGACAAAGGACGATGGCAC | 21 | gp4 |
| PC-3p-1350108_3 | TGCCATCGTCCTTTGTCCGCTGTT | 24 | gp4 |
| PC-5p-389334_11 | GCGGACAAAGGACGATGGCAC | 21 | gp4 |
| PC-3p-1350108_3 | TGCCATCGTCCTTTGTCCGCTGTT | 24 | gp4 |
| PC-5p-113875_40 | AGAGCTGAGAGTTCCCCTTTTGGGC | 25 | gp4 |
| PC-3p-625211_6 | TGTCAAGCGTTCTGCGTTTGGGGC | 24 | gp4 |
| PC-5p-113875_40 | AGAGCTGAGAGTTCCCCTTTTGGGC | 25 | gp4 |
| PC-5p-28067_159 | TCCTATGGCATCACTGGGGCCGGC | 24 | gp4 |
| PC-3p-50259_92 | TGATGTTCTGGGCCCGACGTGGTACC | 26 | gp4 |
| PC-5p-133891_33 | TCCTACGTACATCTTTGATGCTGTC | 25 | gp4 |
| PC-3p-510730_8 | ACACAATAAAGCTGGGTGACCTCACA | 26 | gp4 |
| PC-5p-10507_358 | TGTTCTTCTGCCAGCGCTGGGGC | 23 | gp4 |
| PC-3p-52432_89 | TGTCCCAGGACGCAGTGGCTCATC | 24 | gp4 |
| PC-5p-181492_24 | GCGGTGCGGCTGTGCTTGTGACAGC | 25 | gp4 |
| PC-3p-19523_219 | TGTCAGAGTAGAGGCAGCGCTGCGGC | 26 | gp4 |
| PC-5p-63297_73 | CACTGTGAAGCGGCTCTGG | 19 | gp4 |
| PC-3p-492301_8 | TTCAGACACATGAGAATTGAT | 21 | gp4 |
| PC-5p-606429_7 | GAAGGGCCCAGGGCACATGGCATC | 24 | gp4 |
| PC-3p-2204174_2 | GATCTGAGCTTTGACTACAGCTGCC | 25 | gp4 |
| PC-5p-181428_24 | GTGAGTAGAGCGGGTGTGAGCC | 22 | gp4 |
| PC-3p-505185_8 | ACTACCAGAACCCCACGGGCTGG | 23 | gp4 |
| PC-5p-71982_65 | TTCTGCTCCTATTTAAGTCAAT | 22 | gp4 |
| PC-3p-77518_60 | TGATTTCAATAAGAGCAGAAT | 21 | gp4 |
| PC-5p-167801_26 | TAATTCGGATCCAAACCATTGATT | 24 | gp4 |
| PC-3p-1101815_4 | AAATGACTTTGATGGCAATTCTCACA | 26 | gp4 |
| PC-5p-118093_38 | TTTGTGCCACAGCTGGAGAGCAGAGC | 26 | gp4 |
| PC-3p-2483451_2 | TGAGCGTGCTGCACGCAGAGACTGAT | 26 | gp4 |
| PC-5p-243166_18 | TTCTGGCAGCCAATCCCACCAGGGTA | 26 | gp4 |
| PC-3p-355163_12 | GAGGAGCTCTGGGACACGCTGCCT | 24 | gp4 |
| PC-5p-980793_4 | GAACTTTTATTGCGTGCGATGAACC | 25 | gp4 |
| PC-3p-1493521_3 | TTAAACGTGACCCTCTTGTCAGT | 23 | gp4 |
| PC-5p-511027_8 | TCATTCAGAAGGGTCTGGGGAT | 22 | gp4 |
| PC-3p-69790_67 | TCATCCAGAAGGGTCTGGGGATGTC | 25 | gp4 |
| PC-5p-660271_6 | TCTGGAAAAGAGCTGTGGTTTCT | 23 | gp4 |
| PC-3p-1217079_3 | TTTGCCAACATCTCTTGGCTAGAGAA | 26 | gp4 |
| PC-5p-112606_40 | AGAGCTGAGAGTTCCCCTTTTGG | 23 | gp4 |
| PC-3p-1479789_3 | GTCAAGCGTTCTGCGTTTGGGGCAGC | 26 | gp4 |
| PC-5p-35653_128 | AGCCCATGGACGTGGATCTGCCTCC | 25 | gp4 |
| PC-3p-56447_82 | AACCAATGGAGGTGGATCCATCTTC | 25 | gp4 |
| PC-5p-97606_47 | GCAGAAGGTGTTGAGCAAG | 19 | gp4 |
| PC-3p-204019_21 | TCAGCTCCTCCTGAGTGTGGTGGAGA | 26 | gp4 |
| PC-5p-1583206_2 | ACGGCTGGGCTTGGTGGAGCACA | 23 | gp4 |
| PC-3p-1338762_3 | GACGGCTGGGCTTGGTGGAGCACATT | 26 | gp4 |
| PC-5p-1423460_3 | TCTGGAGCCAAGAATGTCT | 19 | gp4 |
| PC-3p-2522867_2 | TAATGTCGGTTTCCAAGAGCTACC | 24 | gp4 |
| PC-5p-548407_7 | TGGTCATAGCTGCCACTGGACACGCA | 26 | gp4 |
| PC-3p-1641043_2 | GATGGTGGGTGAGAATTCAGGTGT | 24 | gp4 |
| PC-5p-175704_25 | TATGAACTGTCAGCTTATGAACTTGT | 26 | gp4 |
| PC-3p-539082_8 | TAGGTGCTGCTGTCCCCACTGTCT | 24 | gp4 |
| PC-5p-594946_7 | TCCTGAGAAATTAGCATTGCACTGAT | 26 | gp4 |
| PC-3p-1174314_3 | TATATGCTGATCCTAGGATCTCACCT | 26 | gp4 |
| PC-5p-26228_169 | CGAGGTAGAAGGCGCTCCCTGTGACC | 26 | gp4 |
| PC-3p-11513_334 | GAGGTAGAAGGCGCTCCCTGTGACC | 25 | gp4 |
| PC-5p-967306_4 | CTGCAAAGCACGTGCAAAGAGCT | 23 | gp4 |
| PC-3p-136421_33 | TGGCCACGAGCGTGCTAAC | 19 | gp4 |
| PC-5p-419180_10 | TCACAGAATCTGATACTTGGGAGGG | 25 | gp4 |
| PC-3p-2274895_2 | ACAGATTACGATGTAGGCCTTTCATC | 26 | gp4 |
| PC-5p-23016_190 | TGACACGGTAGGAGCCCTTGGTGCT | 25 | gp4 |
| PC-3p-21704_199 | TGACACGGTAGGAGCCCTTGGTGCTT | 26 | gp4 |
| PC-5p-312668_13 | GCCCCTGACCCTCACCTTGCC | 21 | gp4 |
| PC-3p-746015_5 | CAAGATGGGGGTCAAGGGAGCT | 22 | gp4 |
| PC-5p-981258_4 | TTCCTCTGTGTCTGAAGACTTGGCA | 25 | gp4 |
| PC-3p-1289800_3 | CAACCACAGAAAGCCAGTTCATGGCC | 26 | gp4 |
| PC-3p-1672184_2 | AGAGGTCCTGCAGGCCTTGTGTGAC | 25 | gp4 |
| PC-3p-1672184_2 | AGAGGTCCTGCAGGCCTTGTGTGAC | 25 | gp4 |
| PC-5p-1543364_3 | TTGATCTACTGTTCAGTCATT | 21 | gp4 |
| PC-3p-287586_15 | TGACTGAAGCACTAGATCAAGA | 22 | gp4 |
| PC-5p-165142_27 | ACAAAGTCCAGCAGCTTAGCT | 21 | gp4 |
| PC-3p-360459_12 | CAGCTAAGCTATTGGACTTTGC | 22 | gp4 |
| PC-5p-130566_34 | CGGCCAAGAAGCTGCTCTGGGCACC | 25 | gp4 |
| PC-3p-307312_14 | TGTGCTGCTGTGGTGCTCAGGGA | 23 | gp4 |
| PC-5p-114826_39 | TCCTTGGGTCTGTGCTGCTCCATG | 24 | gp4 |
| PC-3p-371451_11 | CAGTGACAGAGATGCGGAGGAAG | 23 | gp4 |
| PC-5p-398_3403 | GAGCGGTCGTCAGCCTCCGGCCC | 23 | gp4 |
| PC-3p-1073_1694 | GAGCGGTCGTCAGCCTCCGGCCCT | 24 | gp4 |
| PC-5p-255726_17 | TGTCAGTGGCATGGCACCGAGGGC | 24 | gp4 |
| PC-3p-374855_11 | ATGGATCTGAGGGAGATGGGGAAT | 24 | gp4 |
| PC-5p-609416_7 | TGTGAGAGCCCGACGGAAGCACC | 23 | gp4 |
| PC-3p-790532_5 | TCCTGTCCCAAAGGAGATTCCGGGAT | 26 | gp4 |
| PC-5p-256973_17 | ACCGATAGCACCAGCTGCTGCA | 22 | gp4 |
| PC-3p-625307_6 | TGCAGTTCAGGCAGGAGCACCGTG | 24 | gp4 |
| PC-5p-1973315_2 | AAGTGTGGAACCCACAGCAACTGATG | 26 | gp4 |
| PC-3p-2403332_2 | ACCTTGGGTTCCAGACCCTACACTC | 25 | gp4 |
| PC-5p-1505861_3 | TTGAGGCTATTAAGGAGTTAGGGGC | 25 | gp4 |
| PC-3p-1589882_2 | TCAAGTGCTAGCAGCTCT | 18 | gp4 |
| PC-5p-69873_67 | TCATCTGGTTGGGGTCGCCGTGCC | 24 | gp4 |
| PC-3p-283570_15 | GCTGCGCTGCGCGCGCCGTGGTGGG | 25 | gp4 |
| PC-5p-145763_31 | TTTGCACGCCTTTGCCTTGTGCCC | 24 | gp4 |
| PC-3p-363398_11 | TCCCGTCTGGACGGTGTAAGGCAGCC | 26 | gp4 |
| PC-5p-37270_123 | GCTGGAACGAGCTCTGGGTGCTGCT | 25 | gp4 |
| PC-3p-183227_24 | TCAGAGCCACTTGTTCTTTACCCCG | 25 | gp4 |
| PC-5p-196382_22 | TGAGAGGCTGTTCCTGTCT | 19 | gp4 |
| PC-3p-143287_31 | TGAGAGGCTGTTCCTGTCTTGTAT | 24 | gp4 |
| PC-5p-76057_61 | CGAGATTATCCGGCCTCTGAGGCA | 24 | gp4 |
| PC-3p-915763_4 | TCTGGGAGGAGGATGTTCTCACG | 23 | gp4 |
| PC-5p-840173_5 | TGTGTGTGGCTGTGAGGCAGAGGA | 24 | gp4 |
| PC-3p-1879599_2 | CACTCAGGTACTCGGTGGTGGTCT | 24 | gp4 |
| PC-5p-201155_22 | AGGCAGTGTATTGTTAGTTAGCTGC | 25 | gp4 |
| PC-3p-532391_8 | AGCAACTAACTACACTGCCACT | 22 | gp4 |
| PC-5p-116071_39 | TTTCACAGGACACCCCCCAGCCCACC | 26 | gp4 |
| PC-3p-1471643_3 | TGTGTCACTGAGTGACAGCATCAGGG | 26 | gp4 |
| PC-5p-111125_41 | AGACGTCAGAGATAGGAAAGCGATC | 25 | gp4 |
| PC-3p-1467850_3 | GAGAAAACCTTGGCTGGGGAGC | 22 | gp4 |
| PC-5p-84739_54 | TGATCTCTTACAGTCCTCCTGAGC | 24 | gp4 |
| PC-3p-1033045_4 | GCAAGGGAGAGGCTGAGAAATCACT | 25 | gp4 |
| PC-5p-975917_4 | TTTGGTCTGAAATAACAGAAGGCT | 24 | gp4 |
| PC-3p-1169177_3 | TATCAGCAAAATTGCATCTTGGACCT | 26 | gp4 |
| PC-5p-603699_7 | AAGGTACAGAGCTCAGCCCGGAATT | 25 | gp4 |
| PC-3p-655436_6 | TACAGAAAGAATGTCTTCCAAGGCCC | 26 | gp4 |
| PC-5p-33394_136 | ATGGCTCTGAGACGGCCACTGGG | 23 | gp4 |
| PC-3p-97072_47 | ACGTTCCAGCACCAAAGCTCGC | 22 | gp4 |
| PC-5p-37716_121 | TGGGTCTGGCTCTCCTCTGCTGCT | 24 | gp4 |
| PC-3p-29538_152 | TGGGTCTGGCTCTCCTCTGCTGCTT | 25 | gp4 |
| PC-5p-47265_98 | GATGCTGCTCTGGACCAACAGCCTGT | 26 | gp4 |
| PC-3p-481919_8 | TTGCTCATTACATACTTGTCATTAAG | 26 | gp4 |
| PC-5p-47227_98 | CAGGCCTGGCTGTTCCTGGGCACC | 24 | gp4 |
| PC-3p-250740_17 | TTCTTCAGGGGATTGCAATGGCAGG | 25 | gp4 |
| PC-5p-208515_21 | TAAGTGCTGGCCGTTGTCCTCT | 22 | gp4 |
| PC-3p-234558_18 | TTAGAGGACAATGGCCAATACT | 22 | gp4 |
| PC-5p-782228_5 | TATCTGCCCCTGACCCTCATCTTGC | 25 | gp4 |
| PC-3p-921757_4 | GCAAGATGGGGGTCAAGGGAC | 21 | gp4 |
| PC-5p-227215_19 | TGCAGTAAGATTATTCCTTGAAGAGT | 26 | gp4 |
| PC-3p-705640_6 | TGTGATTTTTATTGCCAGCTCTGGCT | 26 | gp4 |
| PC-5p-54732_85 | TCCTTGGGAAACTGGATGGTCAGA | 24 | gp4 |
| PC-3p-86168_53 | TTTGAAGTGATTTGCTGTTTCTTTCC | 26 | gp4 |
| PC-5p-419242_10 | TTGTGTTCTTTGTTGTGTTGACCTCT | 26 | gp4 |
| PC-3p-509479_8 | TCCGAGCGGTAGCGCAGAAGGAACGG | 26 | gp4 |
| PC-5p-11309_338 | TCCCGAGGGACCTGGGCACTTGCT | 24 | gp4 |
| PC-3p-247985_17 | TCCTAGCGTGACCCCTGCTGC | 21 | gp4 |
| PC-5p-200761_22 | TGTGACATCACAGAGCGGGGCATG | 24 | gp4 |
| PC-3p-598228_7 | ACAGTCTTTATCAAGTCACGTTGGCA | 26 | gp4 |
| PC-5p-265482_16 | TACACGTCGCAGGGAGAGAGGC | 22 | gp4 |
| PC-5p-265482_16 | TACACGTCGCAGGGAGAGAGGC | 22 | gp4 |
| PC-5p-142320_31 | TAGCACAAGAGAAGAGCAGCTCATC | 25 | gp4 |
| PC-3p-242195_18 | TTTTCTTCTCTCTGCCTGCTGCCT | 24 | gp4 |
| PC-5p-21310_203 | CTCCTTACCCGCTCTGCAGCCTCT | 24 | gp4 |
| PC-3p-43895_105 | GTGCAGGATAGCCTCCTTTCCCATC | 25 | gp4 |
| PC-3p-511018_8 | TCTGTACATCCTCTTCTGGGAGCT | 24 | gp4 |
| PC-3p-511018_8 | TCTGTACATCCTCTTCTGGGAGCT | 24 | gp4 |
| PC-5p-92816_49 | TGATGAAATCCCCCTATGGCCTGT | 24 | gp4 |
| PC-3p-438048_9 | GGATAGGTGGGTTTCATTGAAT | 22 | gp4 |
| PC-5p-14258_282 | TCAGTTGTTGAGAGGATG | 18 | gp4 |
| PC-3p-106665_43 | TACTGTGAGTTTTCTGATTATGTCAT | 26 | gp4 |
| PC-5p-392403_11 | CAGGACAATAGCAACAGGAGGAGC | 24 | gp4 |
| PC-5p-392403_11 | CAGGACAATAGCAACAGGAGGAGC | 24 | gp4 |
| PC-5p-208997_21 | TAAGAAGGTGACTCGAAGCTGCCT | 24 | gp4 |
| PC-3p-489864_8 | TGTCTGTGGATCCCAGTTCTGGCT | 24 | gp4 |
| PC-5p-112025_40 | TGTTCTTCTGCCAGCGCTGGGG | 22 | gp4 |
| PC-3p-10507_358 | TGTTCTTCTGCCAGCGCTGGGGC | 23 | gp4 |
| PC-5p-1599963_2 | TCCTCCCATGCGTCCACCCAGGCCT | 25 | gp4 |
| PC-3p-3184576_1 | ACTCACCTGACTCCCGTGATGCTGCT | 26 | gp4 |
| PC-5p-79288_58 | GCTGGAACGCGCTCTGGGTGCT | 22 | gp4 |
| PC-3p-218825_20 | TCAGAGCCACTTGTGCTGTACCCCG | 25 | gp4 |
| PC-3p-57704_81 | TCAGCGGGCGGCGCGGCGGC | 20 | gp4 |
| PC-5p-1235069_3 | TGGGCTTGTTCAGCTTGGAGAAGGC | 25 | gp4 |
| PC-5p-1878828_2 | AAGGCAAGGCAAGGCAAGGCAAAGA | 25 | gp4 |
| PC-5p-234704_18 | TCCAGTCTGATGGCACCTCCATGAC | 25 | gp4 |
| PC-5p-592370_7 | TATGTAGGAAAGGAGCGTAGGGATC | 25 | gp4 |
| PC-3p-321557_13 | TCAAGACAAAAGATTTCTC | 19 | gp4 |
| PC-3p-118072_38 | AGATGAGGAAGAATTTTTT | 19 | gp4 |
| PC-3p-88137_52 | TCCTGGTCGTCTCTGGGGT | 19 | gp4 |
| PC-5p-571585_7 | GAGGACAATAGCAACAGGAGGAGCA | 25 | gp4 |
| PC-5p-105021_43 | ACCAGATTCCACTCAGAACCACCTG | 25 | gp4 |
| PC-5p-62239_75 | CAGGTGCAGATCTTGGTGG | 19 | gp4 |
| PC-3p-908563_4 | GGGGAACGGAGCTGTGAGG | 19 | gp4 |
| PC-3p-247913_17 | TCTGCAGAAACCTGTGTGT | 19 | gp4 |
| PC-5p-16235_254 | TGTCTAATGCTGCCTCCATGCTTCCA | 26 | gp4 |
| PC-3p-776078_5 | GCTGTCTGGTGAAGTCCCC | 19 | gp4 |
| PC-3p-423361_10 | GGGCGGGCGGTCGGTGCGG | 19 | gp4 |
| PC-5p-366177_11 | TGCGTTTTGATCTTTTATT | 19 | gp4 |
| PC-3p-39506_116 | TCACAAGGACTCTCTGTGACGCTGT | 25 | gp4 |
| PC-3p-3330144_1 | GCACTTTTGAAGATAGAGGGCGTGAT | 26 | gp4 |
| PC-3p-382148_11 | GTCTGGAGGATTCAGGCATGGT | 22 | gp4 |
| PC-3p-283434_15 | TTTGGGTGCCAGCCCCGTTGCGT | 23 | gp4 |
| PC-3p-1339990_3 | GCAAGACTCAGAGATATAAT | 20 | gp4 |
| PC-5p-14476_279 | CCGTCGGATCCCCGCCTC | 18 | gp4 |
| PC-3p-192338_23 | TCGGAGGACTGTGGAGGG | 18 | gp4 |
| PC-3p-472283_9 | TGCAATCCGCCCTGGGCCAGA | 21 | gp4 |
| PC-3p-9441055_1 | TGGTGCGAAATTGGGGACTGTATGT | 25 | gp4 |
| PC-5p-363578_11 | CCGGAAGGCCCCGGGCGC | 18 | gp4 |
| PC-5p-689286_6 | CTGTATCTCTGTGCTTTC | 18 | gp4 |
| PC-5p-9286769_1 | AATGAAGGGTGAGAAGTGTCTGTAGA | 26 | gp4 |
| PC-5p-314769_13 | TTCTCTTGGATGTGGAAA | 18 | gp4 |
| PC-5p-442527_9 | TTTGTTGTGTTTGGCACTG | 19 | gp4 |
| PC-3p-2449910_2 | TGTATTGATGAGTGGCAGT | 19 | gp4 |
| PC-3p-881288_5 | GTGGAGCAGAAGCTGGGCTGC | 21 | gp4 |
| PC-5p-711529_6 | TCCCCTCAGCCTTCTCAGC | 19 | gp4 |
| PC-3p-10124405_1 | TCCCCTGGAAATCTGCTGCCGGGCCA | 26 | gp4 |
| PC-5p-128279_35 | CTTCATCACTCTGGTGTT | 18 | gp4 |
| PC-5p-1455778_3 | TCCACCTGGGCTCTGTATCATGCACT | 26 | gp4 |
| PC-3p-84067_55 | CCGGCGGCGCCGGGCGGGGC | 20 | gp4 |
| PC-5p-351370_12 | CAGCGGTTGACTGGTGTTC | 19 | gp4 |
| PC-3p-836952_5 | AGCCGGCGGGAGCCCCGGG | 19 | gp4 |
| PC-5p-2278198_2 | ATCATCGGGGCAACGTGTGAACATC | 25 | gp4 |
| PC-3p-187994_23 | TCTGTATGCACATGGAGC | 18 | gp4 |
| PC-3p-393805_11 | ACAGAGGCCAAGGCTGACC | 19 | gp4 |
| PC-5p-2142728_2 | ATCCATCAGCCTGGTTACC | 19 | gp4 |
| PC-5p-746967_5 | TCTCTGGGCAGCCTGTGCCAGTGCCA | 26 | gp4 |
| PC-3p-1046612_4 | TCATGTGGAAGGTCAGTC | 18 | gp4 |
| PC-5p-191258_23 | TGCTGTGCTGTATAACACCAACC | 23 | gp4 |
| PC-5p-709756_6 | TCCCACTGACTGCACTATT | 19 | gp4 |
| PC-5p-534914_8 | TGCCTGGCTGGTGGTGCTGT | 20 | gp4 |
| PC-5p-1558429_3 | TCCAAACTTTACTGAGCTT | 19 | gp4 |
| PC-5p-219666_20 | CTGGGGCGCGGGCCCGGGT | 19 | gp4 |
| PC-3p-996330_4 | AAGTGTTGGAAGAGGCTGC | 19 | gp4 |
| PC-5p-258661_17 | AACCGTGAAGGAGGGCAGT | 19 | gp4 |
| PC-5p-582426_7 | CCCAAGAAACAGGACACCT | 19 | gp4 |
| PC-3p-358708_12 | AGAGTGTCTGTTACCTGACCAC | 22 | gp4 |
| PC-5p-1230257_3 | TGCAAGAGGAAGAGAGAAT | 19 | gp4 |
| PC-5p-1008303_4 | TCTCGCTTTGTTGGATTT | 18 | gp4 |
| PC-3p-357559_12 | CCATCCTCACGTCCGCCCGTCT | 22 | gp4 |
| PC-3p-173245_25 | CCCTGACAGTGGACAGTTG | 19 | gp4 |
| PC-5p-100831_45 | AGATGATGCAGGCTGTGTC | 19 | gp4 |
| PC-3p-282885_15 | CCGGGCGCGACCCGCTCC | 18 | gp4 |
| PC-5p-411212_10 | GCCCCCATCGATTTGCTCTAC | 21 | gp4 |
| PC-3p-582247_7 | TTGGGGCTAATAAATCAAA | 19 | gp4 |
| PC-3p-1271749_3 | CGGTGAATCCATGCTGACT | 19 | gp4 |
| PC-3p-436462_9 | GGGCGCGGGCCCGGGTGGA | 19 | gp4 |
| PC-3p-1745879_2 | CAACGCTCCCAGAAATCTCCTGTTTC | 26 | gp4 |
| PC-3p-1019974_4 | TGGGAGCACTCTGAGCCTTGGGACTC | 26 | gp4 |
| PC-5p-629482_6 | GCTGTGCTTCCCGGCCCTGTGCTCTC | 26 | gp4 |
| PC-5p-1534164_3 | GAGGACAATAGCAACAGGAGGAGT | 24 | gp4 |
| PC-5p-996655_4 | GCACTCGGGCTGTGCTTTGGCTGT | 24 | gp4 |
| PC-5p-855302_5 | TATTGTGTTGGCTGCTAT | 18 | gp4 |
| PC-5p-2411193_2 | TGCAGGTGCAGACAGACACGT | 21 | gp4 |
| PC-3p-338480_12 | TTCAGGGTGATGACGGAGGGTG | 22 | gp4 |
| PC-5p-615850_7 | TGCACGGCTGCAGCCTCCTCACC | 23 | gp4 |
| PC-3p-2259216_2 | TGTAATGATACAGTGCTGCGATT | 23 | gp4 |
| PC-3p-214993_20 | GGGAAGGGCTGCCGGCGG | 18 | gp4 |
| PC-5p-175928_25 | CTTTCCCCGCTCCCGCTC | 18 | gp4 |
| PC-5p-792809_5 | CTGTTCAGCCTGCACAAGA | 19 | gp4 |
| PC-3p-1094950_4 | CGGACGAGGCTTCCCGCGCTGT | 22 | gp4 |
| PC-3p-459246_9 | TTACTCCTGAACACCTGC | 18 | gp4 |
| PC-3p-122497_37 | AGCCGGGGATGATTTCTGC | 19 | gp4 |
| PC-5p-69343_67 | AACCTTCCCCCTTCTGTT | 18 | gp4 |
| PC-3p-457530_9 | ATAAAGAAGGCGTTCCTTG | 19 | gp4 |
| PC-5p-317585_13 | TGAGTGTGCTGTGGGCTGCGT | 21 | gp4 |
| PC-5p-145884_31 | GTTGACAAGATCTCTCAGTATGGACC | 26 | gp4 |
| PC-5p-750323_5 | TTGGTGGAGAATGCGGGCAAACAGCT | 26 | gp4 |
| PC-3p-409533_10 | ACCAGAGTACACTGCTGAC | 19 | gp4 |
| PC-3p-3122_828 | TCTGATCGTTTTTTCACT | 18 | gp4 |
| PC-5p-487639_8 | TGGTGTTGAGCTGGGAGCCAC | 21 | gp4 |
| PC-5p-1218828_3 | TGGATGCAGTGCGGCAGGTGG | 21 | gp4 |
| PC-5p-2617817_2 | TCTCTGGAGTCATGTGAGGCCA | 22 | gp4 |
| PC-3p-1562923_3 | GCCGGTCGGTTGTTGAGTG | 19 | gp4 |
| PC-3p-748472_5 | TCGGGAGGAGGCGGGCGGG | 19 | gp4 |
| PC-3p-293495_14 | GACCTCTGTAGGATTCTGCGGC | 22 | gp4 |
| PC-3p-841572_5 | CACATGGGAAGAGGCACTG | 19 | gp4 |
| PC-5p-233284_18 | TGTGTTCTTTGTTGTGTTGACCTCTT | 26 | gp4 |
| PC-5p-802070_5 | GAAATGGGAGCAGCTCTGGGGCACC | 25 | gp4 |
| PC-5p-502995_8 | GCTGTGCGGATTGAGCAAG | 19 | gp4 |
| PC-3p-795040_5 | CCTTGGTGGAGAGCAGTCCTGGAGC | 25 | gp4 |
| PC-3p-423580_10 | ACAAGTGACTTGCTTTATC | 19 | gp4 |
| PC-3p-1099852_4 | TGCAGTCAGAATGACAGAA | 19 | gp4 |
| PC-3p-8457892_1 | TTTGCAGTCAGAACTTCTTACAGCA | 25 | gp4 |
| PC-3p-865997_5 | TACCCAGCATGTACCTGTTAACTTGC | 26 | gp4 |
| PC-5p-2347472_2 | CAGGCAAGGCAAGACAAGGC | 20 | gp4 |
| PC-3p-1174470_3 | TGTGATTGCTCTCTTTGTGT | 20 | gp4 |
| PC-3p-70220_66 | CAGAGCAGCTCCCTCGCT | 18 | gp4 |
| PC-3p-391950_11 | CCAGGCTGCAGGAGCACAC | 19 | gp4 |
| PC-5p-914382_4 | ATGAACAAGAACCTGAGT | 18 | gp4 |
| PC-5p-679131_6 | TCATGCTGTAGATAAGGGGGTTCACT | 26 | gp4 |
| PC-3p-9281_392 | CGGCGTCGGGCCGGCCGC | 18 | gp4 |
| PC-5p-427705_10 | TTGTGTTCTTTGTTGTGTTGACCT | 24 | gp4 |
| PC-5p-479610_9 | ATGGTCACAGTTGATCACTGA | 21 | gp4 |
| PC-3p-58075_80 | CAAAGCAGGCTGGCCGCC | 18 | gp4 |
| PC-3p-714559_6 | TCTGACTGGAGATCCCTGC | 19 | gp4 |
| PC-3p-142505_31 | GCGGGCCCGGGTGGAGCC | 18 | gp4 |
| PC-3p-816752_5 | ATGAAGATGCTGTAGGATG | 19 | gp4 |
| PC-3p-76563_60 | CGGGCGGGCGGTCGGTGC | 18 | gp4 |
| PC-5p-849309_5 | TGGCTGCGGATGTGGCTTCCGTCCCT | 26 | gp4 |
| PC-5p-141169_32 | TTTAGGGTGCAGTCTCTT | 18 | gp4 |
| PC-3p-1365681_3 | TGGAGAAGGACTTGGGTT | 18 | gp4 |
| PC-3p-465678_9 | CAGGAACAACTGAAGTGAATT | 21 | gp4 |
| PC-5p-4603_641 | AGACATGAGAGGTGTAGA | 18 | gp4 |
| PC-5p-118249_38 | CGCCGCTGTCCGACCGGGGC | 20 | gp4 |
| PC-5p-91695_50 | TTGAGTCTTCGTAGCAGCCATTTCC | 25 | gp4 |
| PC-5p-167610_26 | CCCAGCCCGCGGACGGTG | 18 | gp4 |
| PC-3p-404187_10 | TGTCACTGAATTCCTCTGCA | 20 | gp4 |
| PC-5p-575754_7 | ACCAGATTCCACTCAGAACCACCT | 24 | gp4 |
| PC-3p-115599_39 | CTGCAGGACACCTTGAGCC | 19 | gp4 |
| PC-3p-1257890_3 | TCCTGGGAATGGAGACAAC | 19 | gp4 |
| PC-5p-273595_16 | TCACAGGGCATTGGCACTGGCACC | 24 | gp4 |
| PC-3p-353599_12 | AACATTCTCCTGTTTCTTC | 19 | gp4 |
| PC-5p-567138_7 | TGTGTAGGTCTGCAGGAAA | 19 | gp4 |
| PC-5p-973200_4 | CACTGTATGGTGCTGTACGCATT | 23 | gp4 |
| PC-5p-715718_6 | CAGAACGGGAACTGCAGGC | 19 | gp4 |
| PC-5p-456869_9 | TAGGACAATAACAACAGGAGGAGC | 24 | gp4 |
| PC-5p-2530020_2 | GATGATGCTCTGGACCAACAGC | 22 | gp4 |
| PC-5p-1514103_3 | GATGAACTATGCCTGGGC | 18 | gp4 |
| PC-5p-997110_4 | CTTCTTCTGCTTGGTTCTT | 19 | gp4 |
| PC-3p-489758_8 | TCCATGGGCTCTTCTGGGT | 19 | gp4 |
| PC-3p-400624_10 | TACTTCAGGACAGTAGGGGA | 20 | gp4 |
| PC-3p-2415732_2 | TGCTGCTCAGCCTCTCTGACCT | 22 | gp4 |
| PC-5p-530277_8 | GCTGTGCTTCCCGGCCCTGTGCTCT | 25 | gp4 |
| PC-3p-1044867_4 | TCAGCAGTGACGGCATTCTGT | 21 | gp4 |
| PC-5p-5773_549 | CGGCTGGGAAGGGCTGCC | 18 | gp4 |
| PC-5p-574155_7 | TTCTGCGATCAAGAGGGATC | 20 | gp4 |
| PC-3p-183872_24 | CAATGCCCCTGCCATGCAGCCT | 22 | gp4 |
| PC-3p-539275_8 | AACGCCTGGAGACTGGGAG | 19 | gp4 |
| PC-5p-2587554_2 | TGACCTTTGTTGGGCAATGGATGA | 24 | gp4 |
| PC-3p-954719_4 | CTCATGCCCTCTCTGTGCCAGT | 22 | gp4 |
| PC-3p-1165075_3 | TCAGCCACTGTCAGTGCTCAGA | 22 | gp4 |
| PC-5p-771600_5 | TGTGGAGCAGTGCCTGACA | 19 | gp4 |
| PC-5p-1811147_2 | TGGAGGGCAGAAGCGGCA | 18 | gp4 |
| PC-3p-1266128_3 | AGCAACAGGACAAGAAGGA | 19 | gp4 |
| PC-3p-387912_11 | TCTGACTCCTGCTTCTCATCCCATT | 25 | gp4 |
| PC-3p-173380_25 | TCGGCCCTGGGAGTGTCGTTCT | 22 | gp4 |
| PC-3p-189410_23 | CCTCTGTGTGCATGAGTTC | 19 | gp4 |
| PC-3p-865968_5 | GTGAGCGCTGTGGGGCCGGGAGC | 23 | gp4 |
| PC-3p-2333827_2 | GAAGCTGGAAGTAGAGTT | 18 | gp4 |
| PC-5p-418733_10 | CCATTAGCGGCAGCTTATC | 19 | gp4 |
| PC-5p-840215_5 | TGCTGATGCAAGCACCGGG | 19 | gp4 |
| PC-3p-360843_12 | AAGAACTTTGAAGAGAGT | 18 | gp4 |
| PC-3p-50034_93 | TGCCTGGGCAGGGCGAAGC | 19 | gp4 |
| PC-5p-8157_430 | TGGTGTATGTGCTTGGCT | 18 | gp4 |

**S2 Table. Conservative testicular differential expressed miRNAs induced by APS supplements in breeder cock compared with goat, cow and mouse testicular miRNAs.**

| **miRNAs name** | **miRNAs sequences** |
| --- | --- |
| gga-miR-34b-5p_L-1R+1 | AGGCAGTGTAGTTAGCTGATTGT |
| chi-miR-16b-5p_R+1 | TAGCAGCACGTAAATATTGGGGA |
| gga-miR-33-3p_L+1R+1 | CAATGTTCCTGCAGTGCAGTAT |
| aca-miR-363-3p_R+1 | AATTGCACGGTATCCATCTGTA |
| oan-miR-363-3p_R+1 | AATTGCACGGTATCCATCTGTA |
| aca-miR-18a-5p_R-2 | TAAGGTGCATCTAGTGCAGAT |
| gga-let-7g-3p_1ss22CT | CTGTACAGGCCACTGCCTTGCT |
| gga-miR-1a-3p_R+1_1ss2GT | TTGAATGTAAAGAAGTATGTAT |
| gga-miR-30a-5p_R+2_1ss13CT | TGTAAACATCCTTGACTGGAAGCT |
| gga-miR-30e-5p_R+5 | TGTAAACATCCTTGACTGGAAGCT |
| hsa-miR-483-3p_L-1R+2 | CACTCCTCTCCTCCCGTCTTCT |
| gga-miR-16-5p | TAGCAGCACGTAAATATTGGTG |
| aca-miR-425-3p_L-2R+1_1ss3AT | TCGGGGATGTCGTGTCTGTCC |

S3 Table. Target genes of the 33 differential expression miRNAs induced by APS.

| Symbol | Gene Annoation | miRNA ID |
| --- | --- | --- |
| RFK | riboflavin kinase | PC-5p-113875_40 |
| RFK | riboflavin kinase | PC-5p-97606_47 |
| RFK | riboflavin kinase | chi-miR-16b-5p_R+1 |
| RFK | riboflavin kinase | gga-let-7g-3p_1ss22CT |
| RFK | riboflavin kinase | gga-miR-16-5p |
| RFK | riboflavin kinase | gga-miR-1677-3p_L+1 |
| ARID4A | AT rich interactive domain 4A (RBP1-like) | gga-let-7g-3p_1ss22CT |
| ARID4A | AT rich interactive domain 4A (RBP1-like) | gga-miR-30a-5p_R+2_1ss13CT |
| ARID4A | AT rich interactive domain 4A (RBP1-like) | gga-miR-30e-5p_R+5 |
| LAMTOR3 | late endosomal/lysosomal adaptor, MAPK and MTOR activator 3 | PC-5p-113875_40 |
| ARMC1 | armadillo repeat containing 1 | PC-5p-233284_18 |
| ARMC1 | armadillo repeat containing 1 | aca-miR-363-3p_R+1 |
| ARMC1 | armadillo repeat containing 1 | gga-miR-1782_L+2R-1 |
| ARMC1 | armadillo repeat containing 1 | oan-miR-363-3p_R+1 |
| SPR | sepiapterin reductase (7,8-dihydrobiopterin:NADP+ oxidoreductase) | PC-5p-37716_121 |
| GFPT1 | glutamine--fructose-6-phosphate transaminase 1 | PC-5p-113875_40 |
| GFPT1 | glutamine--fructose-6-phosphate transaminase 1 | PC-5p-71982_65 |
| GFPT1 | glutamine--fructose-6-phosphate transaminase 1 | gga-miR-1782_L+2R-1 |
| TIMM17A | translocase of inner mitochondrial membrane 17 homolog A (yeast) | PC-3p-338480_12 |
| TIMM17A | translocase of inner mitochondrial membrane 17 homolog A (yeast) | PC-5p-113875_40 |
| UBE2T | ubiquitin-conjugating enzyme E2T (putative) | PC-5p-113875_40 |
| TRIM39 | tripartite motif containing 39 | PC-5p-92816_49 |
| TRIM39 | tripartite motif containing 39 | gga-miR-1456-3p_R+1 |
| BMP10 | bone morphogenetic protein 10 | PC-5p-582426_7 |
| ARHGAP25 | Rho GTPase activating protein 25 | PC-3p-338480_12 |
| ARHGAP25 | Rho GTPase activating protein 25 | PC-5p-113875_40 |
| ARHGAP25 | Rho GTPase activating protein 25 | PC-5p-92816_49 |
| ARHGAP25 | Rho GTPase activating protein 25 | chi-miR-16b-5p_R+1 |
| ARHGAP25 | Rho GTPase activating protein 25 | gga-miR-16-5p |
| ARHGAP25 | Rho GTPase activating protein 25 | gga-miR-7468-3p_L+3 |
| SNRPE | small nuclear ribonucleoprotein polypeptide E | tgu-miR-2970-5p_1ss21GT |
| CEPT1 | choline/ethanolamine phosphotransferase 1 | PC-5p-113875_40 |
| CEPT1 | choline/ethanolamine phosphotransferase 1 | PC-5p-582426_7 |
| ADAMTS19 | ADAM metallopeptidase with thrombospondin type 1 motif, 19 | PC-5p-113875_40 |
| C22H20ORF30 | chromosome 22 open reading frame, human C20orf30 | PC-3p-173245_25 |
| C22H20ORF30 | chromosome 22 open reading frame, human C20orf30 | PC-3p-52432_89 |
| C22H20ORF30 | chromosome 22 open reading frame, human C20orf30 | PC-5p-37716_121 |
| C22H20ORF30 | chromosome 22 open reading frame, human C20orf30 | PC-5p-97606_47 |
| C22H20ORF30 | chromosome 22 open reading frame, human C20orf30 | chi-miR-16b-5p_R+1 |
| C22H20ORF30 | chromosome 22 open reading frame, human C20orf30 | gga-miR-16-5p |
| LOH11CR2A | loss of heterozygosity, 11, chromosomal region 2, gene A | chi-miR-16b-5p_R+1 |
| LOH11CR2A | loss of heterozygosity, 11, chromosomal region 2, gene A | gga-miR-16-5p |
| LOH11CR2A | loss of heterozygosity, 11, chromosomal region 2, gene A | gga-miR-1782_L+2R-1 |
| SLC23A2 | solute carrier family 23 (nucleobase transporters), member 2 | gga-miR-1782_L+2R-1 |
| DAD1 | defender against cell death 1 | gga-miR-1677-3p_L+1 |
| PRNP | prion protein | PC-5p-113875_40 |
| PRNP | prion protein | chi-miR-16b-5p_R+1 |
| PRNP | prion protein | gga-miR-16-5p |
| PRNP | prion protein | gga-miR-34b-5p_L-1R+1 |
| TMEM9 | transmembrane protein 9 | chi-miR-16b-5p_R+1 |
| TMEM9 | transmembrane protein 9 | gga-miR-16-5p |
| BNIP3L | BCL2/adenovirus E1B 19kDa interacting protein 3-like | PC-3p-338480_12 |
| BNIP3L | BCL2/adenovirus E1B 19kDa interacting protein 3-like | PC-5p-113875_40 |
| BNIP3L | BCL2/adenovirus E1B 19kDa interacting protein 3-like | aca-miR-18a-5p_R-2 |
| BNIP3L | BCL2/adenovirus E1B 19kDa interacting protein 3-like | gga-let-7g-3p_1ss22CT |
| BNIP3L | BCL2/adenovirus E1B 19kDa interacting protein 3-like | gga-miR-1677-3p_L+1 |
| BNIP3L | BCL2/adenovirus E1B 19kDa interacting protein 3-like | gga-miR-1805-5p_L-1 |
| BNIP3L | BCL2/adenovirus E1B 19kDa interacting protein 3-like | gga-miR-30a-5p_R+2_1ss13CT |
| BNIP3L | BCL2/adenovirus E1B 19kDa interacting protein 3-like | gga-miR-30e-5p_R+5 |
| BNIP3L | BCL2/adenovirus E1B 19kDa interacting protein 3-like | tgu-miR-2970-5p_1ss21GT |
| STARD4 | StAR-related lipid transfer (START) domain containing 4 | PC-3p-457530_9 |
| STARD4 | StAR-related lipid transfer (START) domain containing 4 | aca-miR-363-3p_R+1 |
| STARD4 | StAR-related lipid transfer (START) domain containing 4 | oan-miR-363-3p_R+1 |
| EBF2 | early B-cell factor 2 | PC-5p-233284_18 |
| EBF2 | early B-cell factor 2 | PC-5p-37716_121 |
| EBF2 | early B-cell factor 2 | gga-miR-33-3p_L+1R+1 |
| RDM1 | RAD52 motif 1 | PC-5p-233284_18 |
| WDR36 | WD repeat domain 36 | PC-5p-233284_18 |
| PSMF1 | proteasome (prosome, macropain) inhibitor subunit 1 (PI31) | PC-5p-113875_40 |
| PSMF1 | proteasome (prosome, macropain) inhibitor subunit 1 (PI31) | PC-5p-582426_7 |
| PSMF1 | proteasome (prosome, macropain) inhibitor subunit 1 (PI31) | chi-miR-16b-5p_R+1 |
| PSMF1 | proteasome (prosome, macropain) inhibitor subunit 1 (PI31) | gga-miR-16-5p |
| KCTD9 | potassium channel tetramerisation domain containing 9 | PC-3p-457530_9 |
| KCTD9 | potassium channel tetramerisation domain containing 9 | chi-miR-16b-5p_R+1 |
| KCTD9 | potassium channel tetramerisation domain containing 9 | gga-miR-16-5p |
| KCTD9 | potassium channel tetramerisation domain containing 9 | gga-miR-1677-3p_L+1 |
| KCTD9 | potassium channel tetramerisation domain containing 9 | gga-miR-7468-3p_L+3 |
| DPAGT1 | dolichyl-phosphate (UDP-N-acetylglucosamine) N-acetylglucosaminephosphotransferase 1 (GlcNAc-1-P transferase) | gga-let-7g-3p_1ss22CT |
| HMBS | hydroxymethylbilane synthase | gga-miR-1805-5p_L-1 |
| PCNX | pecanex homolog (Drosophila) | PC-3p-338480_12 |
| PCNX | pecanex homolog (Drosophila) | gga-miR-1a-3p_R+1_1ss2GT |
| TNNI1 | troponin I type 1 (skeletal, slow) | PC-5p-113875_40 |
| NEFM | neurofilament, medium polypeptide | aca-miR-363-3p_R+1 |
| NEFM | neurofilament, medium polypeptide | gga-miR-30a-5p_R+2_1ss13CT |
| NEFM | neurofilament, medium polypeptide | gga-miR-30e-5p_R+5 |
| NEFM | neurofilament, medium polypeptide | oan-miR-363-3p_R+1 |
| PSMC5 | proteasome (prosome, macropain) 26S subunit, ATPase, 5 | chi-miR-16b-5p_R+1 |
| PSMC5 | proteasome (prosome, macropain) 26S subunit, ATPase, 5 | gga-miR-16-5p |
| ADAM28 | ADAM metallopeptidase domain 28 | aca-miR-425-3p_L-2R+1_1ss3AT |
| ADAM28 | ADAM metallopeptidase domain 28 | gga-miR-34b-5p_L-1R+1 |
| ADAM28 | ADAM metallopeptidase domain 28 | tgu-miR-2970-5p_1ss21GT |
| RPS6KA1 | ribosomal protein S6 kinase, 90kDa, polypeptide 1 | PC-5p-71982_65 |
| RPS6KA1 | ribosomal protein S6 kinase, 90kDa, polypeptide 1 | gga-miR-7468-3p_L+3 |
| HMGN2 | high mobility group nucleosomal binding domain 2 | PC-3p-173245_25 |
| HMGN2 | high mobility group nucleosomal binding domain 2 | PC-5p-582426_7 |
| HMGN2 | high mobility group nucleosomal binding domain 2 | gga-let-7g-3p_1ss22CT |
| HMGN2 | high mobility group nucleosomal binding domain 2 | tgu-miR-2970-5p_1ss21GT |
| RNPEP | arginyl aminopeptidase (aminopeptidase B) | aca-miR-425-3p_L-2R+1_1ss3AT |
| ITGB3 | integrin, beta 3 (platelet glycoprotein IIIa, antigen CD61) | PC-3p-52432_89 |
| DHDDS | dehydrodolichyl diphosphate synthase | chi-miR-16b-5p_R+1 |
| DHDDS | dehydrodolichyl diphosphate synthase | gga-miR-1456-5p_L-1 |
| DHDDS | dehydrodolichyl diphosphate synthase | gga-miR-16-5p |
| LIN28A | lin-28 homolog A (C. elegans) | PC-3p-457530_9 |
| IPO9 | importin 9 | PC-5p-582426_7 |
| LSM7 | LSM7 homolog, U6 small nuclear RNA associated (S. cerevisiae) | PC-5p-71982_65 |
| LSM7 | LSM7 homolog, U6 small nuclear RNA associated (S. cerevisiae) | hsa-miR-483-3p_L-1R+2 |
| TLK2 | tousled-like kinase 2 | gga-miR-7468-3p_L+3 |
| CCDC15 | coiled-coil domain containing 15 | PC-5p-71982_65 |
| CCDC15 | coiled-coil domain containing 15 | gga-miR-1a-3p_R+1_1ss2GT |
| HINT1 | histidine triad nucleotide binding protein 1 | PC-5p-582426_7 |
| SPPL2B | signal peptide peptidase like 2B | aca-miR-363-3p_R+1 |
| SPPL2B | signal peptide peptidase like 2B | chi-miR-16b-5p_R+1 |
| SPPL2B | signal peptide peptidase like 2B | gga-let-7g-3p_1ss22CT |
| SPPL2B | signal peptide peptidase like 2B | gga-miR-1329-3p |
| SPPL2B | signal peptide peptidase like 2B | gga-miR-16-5p |
| SPPL2B | signal peptide peptidase like 2B | gga-miR-1782_L+2R-1 |
| SPPL2B | signal peptide peptidase like 2B | gga-miR-1a-3p_R+1_1ss2GT |
| SPPL2B | signal peptide peptidase like 2B | oan-miR-363-3p_R+1 |
| PADI3 | peptidyl arginine deiminase, type III | PC-5p-97606_47 |
| PGM2 | phosphoglucomutase 2 | PC-3p-173245_25 |
| DCAF7 | DDB1 and CUL4 associated factor 7 | gga-miR-1a-3p_R+1_1ss2GT |
| DCAF7 | DDB1 and CUL4 associated factor 7 | gga-miR-30a-5p_R+2_1ss13CT |
| DCAF7 | DDB1 and CUL4 associated factor 7 | gga-miR-30e-5p_R+5 |
| DCAF7 | DDB1 and CUL4 associated factor 7 | gga-miR-33-3p_L+1R+1 |
| DCAF7 | DDB1 and CUL4 associated factor 7 | tgu-miR-2970-5p_1ss21GT |
| FANCA | Fanconi anemia, complementation group A | PC-5p-233284_18 |
| FANCA | Fanconi anemia, complementation group A | gga-miR-34b-5p_L-1R+1 |
| GALK2 | galactokinase 2 | PC-5p-97606_47 |
| SRSF3 | serine/arginine-rich splicing factor 3 | gga-miR-33-3p_L+1R+1 |
| ZNF593 | zinc finger protein 593 | PC-5p-113875_40 |
| ZNF593 | zinc finger protein 593 | chi-miR-16b-5p_R+1 |
| ZNF593 | zinc finger protein 593 | gga-miR-16-5p |
| STRADA | STE20-related kinase adaptor alpha | PC-5p-233284_18 |
| STRADA | STE20-related kinase adaptor alpha | PC-5p-97606_47 |
| STRADA | STE20-related kinase adaptor alpha | gga-miR-1782_L+2R-1 |
| STRADA | STE20-related kinase adaptor alpha | gga-miR-7468-3p_L+3 |
| LAPTM5 | lysosomal protein transmembrane 5 | PC-5p-97606_47 |
| LAPTM5 | lysosomal protein transmembrane 5 | gga-miR-7468-3p_L+3 |
| CCDC47 | coiled-coil domain containing 47 | gga-miR-7468-3p_L+3 |
| RANBP3 | RAN binding protein 3 | PC-5p-582426_7 |
| RANBP3 | RAN binding protein 3 | aca-miR-425-3p_L-2R+1_1ss3AT |
| RANBP3 | RAN binding protein 3 | tgu-miR-2970-5p_1ss21GT |
| DDX42 | DEAD (Asp-Glu-Ala-Asp) box polypeptide 42 | PC-3p-173245_25 |
| DDX42 | DEAD (Asp-Glu-Ala-Asp) box polypeptide 42 | PC-3p-457530_9 |
| DDX42 | DEAD (Asp-Glu-Ala-Asp) box polypeptide 42 | PC-5p-582426_7 |
| DDX42 | DEAD (Asp-Glu-Ala-Asp) box polypeptide 42 | tgu-miR-2970-5p_1ss21GT |
| MYL4 | myosin, light chain 4, alkali; atrial, embryonic | PC-5p-582426_7 |
| MYL4 | myosin, light chain 4, alkali; atrial, embryonic | aca-miR-18a-5p_R-2 |
| CDC27 | cell division cycle 27 homolog (S. cerevisiae) | PC-5p-582426_7 |
| CDC27 | cell division cycle 27 homolog (S. cerevisiae) | aca-miR-363-3p_R+1 |
| CDC27 | cell division cycle 27 homolog (S. cerevisiae) | chi-miR-16b-5p_R+1 |
| CDC27 | cell division cycle 27 homolog (S. cerevisiae) | gga-miR-16-5p |
| CDC27 | cell division cycle 27 homolog (S. cerevisiae) | oan-miR-363-3p_R+1 |
| CAMTA1 | calmodulin binding transcription activator 1 | PC-5p-71982_65 |
| CAMTA1 | calmodulin binding transcription activator 1 | gga-let-7g-3p_1ss22CT |
| PPP1R15B | protein phosphatase 1, regulatory subunit 15B | PC-3p-338480_12 |
| RAB11B | RAB11B, member RAS oncogene family | PC-3p-338480_12 |
| RAB11B | RAB11B, member RAS oncogene family | chi-miR-16b-5p_R+1 |
| RAB11B | RAB11B, member RAS oncogene family | gga-miR-16-5p |
| RAB11B | RAB11B, member RAS oncogene family | gga-miR-1782_L+2R-1 |
| ELF3 | E74-like factor 3 (ets domain transcription factor, epithelial-specific ) | PC-5p-113875_40 |
| CHTF8 | CTF8, chromosome transmission fidelity factor 8 homolog (S. cerevisiae) | PC-3p-52432_89 |
| CHTF8 | CTF8, chromosome transmission fidelity factor 8 homolog (S. cerevisiae) | PC-5p-113875_40 |
| ZBTB48 | zinc finger and BTB domain containing 48 | gga-miR-33-3p_L+1R+1 |
| CNTN2 | contactin 2 (axonal) | aca-miR-363-3p_R+1 |
| CNTN2 | contactin 2 (axonal) | gga-miR-1456-3p_R+1 |
| CNTN2 | contactin 2 (axonal) | oan-miR-363-3p_R+1 |
| TERF2 | telomeric repeat binding factor 2 | PC-5p-92816_49 |
| RBBP5 | retinoblastoma binding protein 5 | PC-5p-92816_49 |
| RBBP5 | retinoblastoma binding protein 5 | chi-miR-16b-5p_R+1 |
| RBBP5 | retinoblastoma binding protein 5 | gga-miR-16-5p |
| RBBP5 | retinoblastoma binding protein 5 | tgu-miR-2970-5p_1ss21GT |
| KIAA0556 | KIAA0556 ortholog | chi-miR-16b-5p_R+1 |
| KIAA0556 | KIAA0556 ortholog | gga-miR-16-5p |
| GDPD4 | glycerophosphodiester phosphodiesterase domain containing 4 | gga-miR-1805-5p_L-1 |
| NOB1 | NIN1/RPN12 binding protein 1 homolog (S. cerevisiae) | PC-5p-37716_121 |
| WWP2 | WW domain containing E3 ubiquitin protein ligase 2 | aca-miR-363-3p_R+1 |
| WWP2 | WW domain containing E3 ubiquitin protein ligase 2 | gga-let-7g-3p_1ss22CT |
| WWP2 | WW domain containing E3 ubiquitin protein ligase 2 | oan-miR-363-3p_R+1 |
| SLC41A1 | solute carrier family 41, member 1 | PC-5p-113875_40 |
| SLC41A1 | solute carrier family 41, member 1 | aca-miR-363-3p_R+1 |
| SLC41A1 | solute carrier family 41, member 1 | oan-miR-363-3p_R+1 |
| PM20D1 | peptidase M20 domain containing 1 | chi-miR-16b-5p_R+1 |
| PM20D1 | peptidase M20 domain containing 1 | gga-miR-16-5p |
| PM20D1 | peptidase M20 domain containing 1 | gga-miR-1677-3p_L+1 |
| PPP1R8 | protein phosphatase 1, regulatory subunit 8 | PC-3p-338480_12 |
| PPP1R8 | protein phosphatase 1, regulatory subunit 8 | gga-miR-1805-5p_L-1 |
| CACNA1S | calcium channel, voltage-dependent, L type, alpha 1S subunit | PC-3p-173245_25 |
| SLC26A9 | solute carrier family 26, member 9 | PC-5p-233284_18 |
| TJP3 | tight junction protein 3 (zona occludens 3) | gga-miR-1456-3p_R+1 |
| TBC1D22B | TBC1 domain family, member 22B | gga-let-7g-3p_1ss22CT |
| SSR2 | signal sequence receptor, beta (translocon-associated protein beta) | PC-5p-71982_65 |
| ZMAT2 | zinc finger, matrin-type 2 | PC-5p-97606_47 |
| ZMAT2 | zinc finger, matrin-type 2 | gga-let-7g-3p_1ss22CT |
| ZMAT2 | zinc finger, matrin-type 2 | hsa-miR-483-3p_L-1R+2 |
| CTSS | cathepsin S | PC-5p-113875_40 |
| CTSS | cathepsin S | PC-5p-582426_7 |
| EYA3 | eyes absent homolog 3 (Drosophila) | PC-5p-37716_121 |
| FAM72A | family with sequence similarity 72, member A | gga-miR-1456-5p_L-1 |
| HARS | histidyl-tRNA synthetase | gga-miR-34b-5p_L-1R+1 |
| HNRNPR | heterogeneous nuclear ribonucleoprotein R | gga-let-7g-3p_1ss22CT |
| HNRNPR | heterogeneous nuclear ribonucleoprotein R | gga-miR-1805-5p_L-1 |
| RASSF5 | Ras association (RalGDS/AF-6) domain family member 5 | PC-5p-233284_18 |
| RASSF5 | Ras association (RalGDS/AF-6) domain family member 5 | PC-5p-37716_121 |
| RASSF5 | Ras association (RalGDS/AF-6) domain family member 5 | PC-5p-97606_47 |
| RASSF5 | Ras association (RalGDS/AF-6) domain family member 5 | chi-miR-16b-5p_R+1 |
| RASSF5 | Ras association (RalGDS/AF-6) domain family member 5 | gga-miR-16-5p |
| WNT11 | wingless-type MMTV integration site family, member 11 | gga-miR-7468-3p_L+3 |
| KDM1A | lysine (K)-specific demethylase 1A | gga-miR-1677-3p_L+1 |
| NMT1 | N-myristoyltransferase 1 | gga-let-7g-3p_1ss22CT |
| SLC7A6 | solute carrier family 7 (amino acid transporter light chain, y+L system), member 6 | aca-miR-18a-5p_R-2 |
| FBXW2 | F-box and WD repeat domain containing 2 | PC-5p-92816_49 |
| FBXW2 | F-box and WD repeat domain containing 2 | gga-miR-1677-3p_L+1 |
| LOC100857226 | selenium-binding protein 1-A-like | aca-miR-363-3p_R+1 |
| LOC100857226 | selenium-binding protein 1-A-like | oan-miR-363-3p_R+1 |
| KIF18B | kinesin family member 18B | chi-miR-16b-5p_R+1 |
| KIF18B | kinesin family member 18B | gga-miR-16-5p |
| NR0B2 | nuclear receptor subfamily 0, group B, member 2 | PC-5p-92816_49 |
| NR0B2 | nuclear receptor subfamily 0, group B, member 2 | gga-miR-34b-5p_L-1R+1 |
| CSNK2A2 | casein kinase 2, alpha prime polypeptide | gga-miR-30a-5p_R+2_1ss13CT |
| CSNK2A2 | casein kinase 2, alpha prime polypeptide | gga-miR-30e-5p_R+5 |
| IL10 | interleukin 10 | PC-3p-338480_12 |
| IL10 | interleukin 10 | gga-miR-30a-5p_R+2_1ss13CT |
| IL10 | interleukin 10 | gga-miR-30e-5p_R+5 |
| TAT | tyrosine aminotransferase | PC-5p-37716_121 |
| NUDC | nuclear distribution C homolog (A. nidulans) | PC-5p-71982_65 |
| NUDC | nuclear distribution C homolog (A. nidulans) | chi-miR-16b-5p_R+1 |
| NUDC | nuclear distribution C homolog (A. nidulans) | gga-miR-16-5p |
| LHFPL5 | lipoma HMGIC fusion partner-like 5 | PC-5p-113875_40 |
| C11H16ORF80 | chromosome 11 open reading frame, human C16orf80 | PC-5p-113875_40 |
| C11H16ORF80 | chromosome 11 open reading frame, human C16orf80 | PC-5p-582426_7 |
| C11H16ORF80 | chromosome 11 open reading frame, human C16orf80 | tgu-miR-2970-5p_1ss21GT |
| PIGR | polymeric immunoglobulin receptor | PC-5p-71982_65 |
| SUPT4H1 | suppressor of Ty 4 homolog 1 (S. cerevisiae) | PC-3p-52432_89 |
| PFDN1 | prefoldin subunit 1 | PC-5p-113875_40 |
| HBEGF | heparin-binding EGF-like growth factor | PC-3p-52432_89 |
| FAM125B | family with sequence similarity 125, member B | PC-5p-71982_65 |
| FAM125B | family with sequence similarity 125, member B | PC-5p-97606_47 |
| FAM125B | family with sequence similarity 125, member B | gga-let-7g-3p_1ss22CT |
| FAM125B | family with sequence similarity 125, member B | gga-miR-1329-3p |
| FAM125B | family with sequence similarity 125, member B | gga-miR-1782_L+2R-1 |
| TMEM222 | transmembrane protein 222 | PC-5p-97606_47 |
| MYH1B | myosin, heavy chain 1B, skeletal muscle (similar to human myosin, heavy chain 1, skeletal muscle, adult) | hsa-miR-483-3p_L-1R+2 |
| AP3D1 | adaptor-related protein complex 3, delta 1 subunit | PC-5p-37716_121 |
| AP3D1 | adaptor-related protein complex 3, delta 1 subunit | gga-miR-1329-3p |
| GJC1 | gap junction protein, gamma 1, 45kDa | PC-3p-173245_25 |
| GJC1 | gap junction protein, gamma 1, 45kDa | tgu-miR-2970-5p_1ss21GT |
| HSPA5 | heat shock 70kDa protein 5 (glucose-regulated protein, 78kDa) | gga-let-7g-3p_1ss22CT |
| HSPA5 | heat shock 70kDa protein 5 (glucose-regulated protein, 78kDa) | gga-miR-1782_L+2R-1 |
| PPP6C | protein phosphatase 6, catalytic subunit | PC-5p-97606_47 |
| PPP6C | protein phosphatase 6, catalytic subunit | chi-miR-16b-5p_R+1 |
| PPP6C | protein phosphatase 6, catalytic subunit | gga-miR-16-5p |
| CCDC43 | coiled-coil domain containing 43 | PC-5p-71982_65 |
| CCDC43 | coiled-coil domain containing 43 | chi-miR-16b-5p_R+1 |
| CCDC43 | coiled-coil domain containing 43 | gga-miR-16-5p |
| TPRG1L | tumor protein p63 regulated 1-like | PC-3p-173245_25 |
| TPRG1L | tumor protein p63 regulated 1-like | aca-miR-363-3p_R+1 |
| TPRG1L | tumor protein p63 regulated 1-like | oan-miR-363-3p_R+1 |
| MAN1C1 | mannosidase, alpha, class 1C, member 1 | aca-miR-18a-5p_R-2 |
| MAN1C1 | mannosidase, alpha, class 1C, member 1 | gga-let-7g-3p_1ss22CT |
| MTMR4 | myotubularin related protein 4 | gga-let-7g-3p_1ss22CT |
| SRPR | signal recognition particle receptor (docking protein) | aca-miR-363-3p_R+1 |
| SRPR | signal recognition particle receptor (docking protein) | oan-miR-363-3p_R+1 |
| C20H20ORF24 | chromosome 20 open reading frame, human C20orf24 | PC-5p-582426_7 |
| C20H20ORF24 | chromosome 20 open reading frame, human C20orf24 | PC-5p-97606_47 |
| C20H20ORF24 | chromosome 20 open reading frame, human C20orf24 | aca-miR-363-3p_R+1 |
| C20H20ORF24 | chromosome 20 open reading frame, human C20orf24 | gga-miR-1782_L+2R-1 |
| C20H20ORF24 | chromosome 20 open reading frame, human C20orf24 | hsa-miR-483-3p_L-1R+2 |
| C20H20ORF24 | chromosome 20 open reading frame, human C20orf24 | oan-miR-363-3p_R+1 |
| TMEM57 | transmembrane protein 57 | aca-miR-363-3p_R+1 |
| TMEM57 | transmembrane protein 57 | oan-miR-363-3p_R+1 |
| TIRAP | toll-interleukin 1 receptor (TIR) domain containing adaptor protein | PC-5p-71982_65 |
| WNT3 | wingless-type MMTV integration site family, member 3 | gga-miR-7468-3p_L+3 |
| NR5A1 | nuclear receptor subfamily 5, group A, member 1 | PC-3p-173245_25 |
| NR5A1 | nuclear receptor subfamily 5, group A, member 1 | PC-3p-52432_89 |
| DCPS | decapping enzyme, scavenger | gga-miR-1677-3p_L+1 |
| ANKHD1 | ankyrin repeat and KH domain containing 1 | gga-miR-30a-5p_R+2_1ss13CT |
| ANKHD1 | ankyrin repeat and KH domain containing 1 | gga-miR-30e-5p_R+5 |
| NEK6 | NIMA (never in mitosis gene a)-related kinase 6 | PC-3p-457530_9 |
| NEK6 | NIMA (never in mitosis gene a)-related kinase 6 | PC-5p-582426_7 |
| WBSCR17 | Williams-Beuren syndrome chromosome region 17 | hsa-miR-483-3p_L-1R+2 |
| HES5 | hairy and enhancer of split 5 (Drosophila) | PC-5p-92816_49 |
| HES5 | hairy and enhancer of split 5 (Drosophila) | PC-5p-97606_47 |
| UBXN6 | UBX domain protein 6 | tgu-miR-2970-5p_1ss21GT |
| PHB | prohibitin | PC-5p-113875_40 |
| PANK4 | pantothenate kinase 4 | gga-miR-1a-3p_R+1_1ss2GT |
| GATSL2 | GATS protein-like 2 | PC-5p-71982_65 |
| KBP | kainate binding protein | aca-miR-363-3p_R+1 |
| KBP | kainate binding protein | oan-miR-363-3p_R+1 |
| WBSCR16 | Williams-Beuren syndrome chromosome region 16 | PC-5p-233284_18 |
| WBSCR16 | Williams-Beuren syndrome chromosome region 16 | PC-5p-582426_7 |
| C4BPA | complement component 4 binding protein, alpha | aca-miR-363-3p_R+1 |
| C4BPA | complement component 4 binding protein, alpha | chi-miR-16b-5p_R+1 |
| C4BPA | complement component 4 binding protein, alpha | gga-miR-16-5p |
| C4BPA | complement component 4 binding protein, alpha | oan-miR-363-3p_R+1 |
| C4BPA | complement component 4 binding protein, alpha | tgu-miR-2970-5p_1ss21GT |
| ARMC8 | armadillo repeat containing 8 | gga-miR-1782_L+2R-1 |
| NCOA6 | nuclear receptor coactivator 6 | gga-let-7g-3p_1ss22CT |
| FAM192A | family with sequence similarity 192, member A | PC-5p-71982_65 |
| FAM192A | family with sequence similarity 192, member A | PC-5p-97606_47 |
| FAM192A | family with sequence similarity 192, member A | gga-miR-1a-3p_R+1_1ss2GT |
| STRBP | spermatid perinuclear RNA binding protein | PC-5p-97606_47 |
| DBR1 | debranching enzyme homolog 1 (S. cerevisiae) | PC-5p-582426_7 |
| DBR1 | debranching enzyme homolog 1 (S. cerevisiae) | gga-miR-1782_L+2R-1 |
| CIAPIN1 | cytokine induced apoptosis inhibitor 1 | PC-3p-338480_12 |
| CIAPIN1 | cytokine induced apoptosis inhibitor 1 | PC-5p-233284_18 |
| CIAPIN1 | cytokine induced apoptosis inhibitor 1 | PC-5p-92816_49 |
| TMEM45B | transmembrane protein 45B | tgu-miR-2970-5p_1ss21GT |
| ENKD1 | chromosome 11 open reading frame, human C16orf48 | PC-3p-282885_15 |
| PPP2R3A | protein phosphatase 2, regulatory subunit B'', alpha | aca-miR-363-3p_R+1 |
| PPP2R3A | protein phosphatase 2, regulatory subunit B'', alpha | oan-miR-363-3p_R+1 |
| PCCB | propionyl CoA carboxylase, beta polypeptide | PC-5p-92816_49 |
| HERPUD1 | homocysteine-inducible, endoplasmic reticulum stress-inducible, ubiquitin-like domain member 1 | gga-miR-1782_L+2R-1 |
| PEX10 | peroxisomal biogenesis factor 10 | gga-let-7g-3p_1ss22CT |
| PIK3R5 | phosphoinositide 3-kinase regulatory subunit 5 | PC-3p-173245_25 |
| PIK3R5 | phosphoinositide 3-kinase regulatory subunit 5 | aca-miR-18a-5p_R-2 |
| PIK3R5 | phosphoinositide 3-kinase regulatory subunit 5 | aca-miR-363-3p_R+1 |
| PIK3R5 | phosphoinositide 3-kinase regulatory subunit 5 | gga-miR-1456-5p_L-1 |
| PIK3R5 | phosphoinositide 3-kinase regulatory subunit 5 | oan-miR-363-3p_R+1 |
| SAMHD1 | SAM domain and HD domain 1 | chi-miR-16b-5p_R+1 |
| SAMHD1 | SAM domain and HD domain 1 | gga-miR-16-5p |
| SAMHD1 | SAM domain and HD domain 1 | tgu-miR-2970-5p_1ss21GT |
| SLC25A1 | solute carrier family 25 (mitochondrial carrier; citrate transporter), member 1 | PC-5p-37716_121 |
| APLP2 | amyloid beta (A4) precursor-like protein 2 | PC-3p-457530_9 |
| APLP2 | amyloid beta (A4) precursor-like protein 2 | gga-miR-1782_L+2R-1 |
| APLP2 | amyloid beta (A4) precursor-like protein 2 | gga-miR-1a-3p_R+1_1ss2GT |
| CREB3L3 | cAMP responsive element binding protein 3-like 3 | PC-5p-37716_121 |
| CREB3L3 | cAMP responsive element binding protein 3-like 3 | PC-5p-92816_49 |
| WDR16 | WD repeat domain 16 | chi-miR-16b-5p_R+1 |
| WDR16 | WD repeat domain 16 | gga-miR-16-5p |
| PLXNA2 | plexin A2 | chi-miR-16b-5p_R+1 |
| PLXNA2 | plexin A2 | gga-miR-16-5p |
| MAP2K2 | mitogen-activated protein kinase kinase 2 | chi-miR-16b-5p_R+1 |
| MAP2K2 | mitogen-activated protein kinase kinase 2 | gga-miR-16-5p |
| PRKCZ | protein kinase C, zeta | chi-miR-16b-5p_R+1 |
| PRKCZ | protein kinase C, zeta | gga-miR-16-5p |
| SRRM1 | serine/arginine repetitive matrix 1 | PC-5p-113875_40 |
| CLTCL1 | clathrin, heavy chain-like 1 | gga-miR-1782_L+2R-1 |
| LOC100857709 | linker for activation of T-cells family member 2-like | PC-3p-173245_25 |
| SNAP29 | synaptosomal-associated protein, 29kDa | PC-5p-97606_47 |
| SNAP29 | synaptosomal-associated protein, 29kDa | gga-miR-1782_L+2R-1 |
| SNAP29 | synaptosomal-associated protein, 29kDa | gga-miR-30a-5p_R+2_1ss13CT |
| SNAP29 | synaptosomal-associated protein, 29kDa | gga-miR-30e-5p_R+5 |
| LOC428335 | hyperpolarization activated cyclic nucleotide-gated potassium channel 2 | PC-3p-457530_9 |
| CAMK1G | calcium/calmodulin-dependent protein kinase IG | PC-5p-113875_40 |
| CAMK1G | calcium/calmodulin-dependent protein kinase IG | chi-miR-16b-5p_R+1 |
| CAMK1G | calcium/calmodulin-dependent protein kinase IG | gga-miR-16-5p |
| BSG | basigin (Ok blood group) | PC-5p-97606_47 |
| ATP5G1 | ATP synthase, H+ transporting, mitochondrial Fo complex, subunit C1 (subunit 9) | PC-5p-92816_49 |
| ATP5G1 | ATP synthase, H+ transporting, mitochondrial Fo complex, subunit C1 (subunit 9) | gga-miR-1677-3p_L+1 |
| ST14 | suppression of tumorigenicity 14 (colon carcinoma) | PC-3p-52432_89 |
| ST14 | suppression of tumorigenicity 14 (colon carcinoma) | gga-miR-34b-5p_L-1R+1 |
| FAM18B1 | family with sequence similarity 18, member B1 | PC-5p-97606_47 |
| FAM18B1 | family with sequence similarity 18, member B1 | gga-let-7g-3p_1ss22CT |
| FAM18B1 | family with sequence similarity 18, member B1 | gga-miR-1677-3p_L+1 |
| FAM18B1 | family with sequence similarity 18, member B1 | gga-miR-34b-5p_L-1R+1 |
| EDC3 | enhancer of mRNA decapping 3 homolog (S. cerevisiae) | PC-3p-173245_25 |
| MYBBP1A | MYB binding protein (P160) 1a | PC-5p-71982_65 |
| HSD11B1 | hydroxysteroid (11-beta) dehydrogenase 1 | PC-3p-338480_12 |
| HSD11B1 | hydroxysteroid (11-beta) dehydrogenase 1 | PC-5p-37716_121 |
| HSD11B1 | hydroxysteroid (11-beta) dehydrogenase 1 | hsa-miR-483-3p_L-1R+2 |
| HSD11B1 | hydroxysteroid (11-beta) dehydrogenase 1 | tgu-miR-2970-5p_1ss21GT |
| POLRMT | polymerase (RNA) mitochondrial (DNA directed) | chi-miR-16b-5p_R+1 |
| POLRMT | polymerase (RNA) mitochondrial (DNA directed) | gga-miR-16-5p |
| PI4KA | phosphatidylinositol 4-kinase, catalytic, alpha | PC-5p-97606_47 |
| UBL7 | ubiquitin-like 7 (bone marrow stromal cell-derived) | PC-3p-52432_89 |
| UBL7 | ubiquitin-like 7 (bone marrow stromal cell-derived) | gga-miR-1456-3p_R+1 |
| UBL7 | ubiquitin-like 7 (bone marrow stromal cell-derived) | tgu-miR-2970-5p_1ss21GT |
| SERPIND1 | serpin peptidase inhibitor, clade D (heparin cofactor), member 1 | aca-miR-363-3p_R+1 |
| SERPIND1 | serpin peptidase inhibitor, clade D (heparin cofactor), member 1 | oan-miR-363-3p_R+1 |
| SPNS3 | spinster homolog 3 (Drosophila) | gga-miR-1805-5p_L-1 |
| UBE2L3 | ubiquitin-conjugating enzyme E2L 3 | gga-miR-30a-5p_R+2_1ss13CT |
| UBE2L3 | ubiquitin-conjugating enzyme E2L 3 | gga-miR-30e-5p_R+5 |
| ADRA1B | adrenergic, alpha-1B-, receptor | chi-miR-16b-5p_R+1 |
| ADRA1B | adrenergic, alpha-1B-, receptor | gga-miR-16-5p |
| ADRA1B | adrenergic, alpha-1B-, receptor | tgu-miR-2970-5p_1ss21GT |
| RALGAPB | Ral GTPase activating protein, beta subunit (non-catalytic) | PC-5p-233284_18 |
| RALGAPB | Ral GTPase activating protein, beta subunit (non-catalytic) | PC-5p-97606_47 |
| MECR | mitochondrial trans-2-enoyl-CoA reductase | PC-5p-71982_65 |
| MECR | mitochondrial trans-2-enoyl-CoA reductase | chi-miR-16b-5p_R+1 |
| MECR | mitochondrial trans-2-enoyl-CoA reductase | gga-miR-16-5p |
| TTC1 | tetratricopeptide repeat domain 1 | PC-5p-582426_7 |
| GUCA1A | guanylate cyclase activator 1A (retina) | PC-5p-113875_40 |
| GUCA1A | guanylate cyclase activator 1A (retina) | PC-5p-71982_65 |
| GSN | gelsolin | aca-miR-363-3p_R+1 |
| GSN | gelsolin | oan-miR-363-3p_R+1 |
| JAM3 | junctional adhesion molecule 3 | gga-miR-34b-5p_L-1R+1 |
| MIB2 | mindbomb E3 ubiquitin protein ligase 2 | chi-miR-16b-5p_R+1 |
| MIB2 | mindbomb E3 ubiquitin protein ligase 2 | gga-miR-16-5p |
| RAB14 | RAB14, member RAS oncogene family | PC-3p-173245_25 |
| RAB14 | RAB14, member RAS oncogene family | aca-miR-363-3p_R+1 |
| RAB14 | RAB14, member RAS oncogene family | gga-let-7g-3p_1ss22CT |
| RAB14 | RAB14, member RAS oncogene family | oan-miR-363-3p_R+1 |
| STMN1 | stathmin 1 | PC-5p-113875_40 |
| STMN1 | stathmin 1 | tgu-miR-2970-5p_1ss21GT |
| RAP1A | RAP1A, member of RAS oncogene family | aca-miR-425-3p_L-2R+1_1ss3AT |
| IRF1 | interferon regulatory factor 1 | chi-miR-16b-5p_R+1 |
| IRF1 | interferon regulatory factor 1 | gga-miR-16-5p |
| IRF1 | interferon regulatory factor 1 | tgu-miR-2970-5p_1ss21GT |
| LOC419409 | Golgi integral membrane protein 4-like | chi-miR-16b-5p_R+1 |
| LOC419409 | Golgi integral membrane protein 4-like | gga-miR-16-5p |
| RRAGC | Ras-related GTP binding C | PC-5p-113875_40 |
| SSU72 | SSU72 RNA polymerase II CTD phosphatase homolog (S. cerevisiae) | gga-let-7g-3p_1ss22CT |
| NCAPD3 | non-SMC condensin II complex, subunit D3 | chi-miR-16b-5p_R+1 |
| NCAPD3 | non-SMC condensin II complex, subunit D3 | gga-miR-16-5p |
| NCAPD3 | non-SMC condensin II complex, subunit D3 | gga-miR-34b-5p_L-1R+1 |
| NISCH | nischarin | PC-5p-71982_65 |
| CNTRL | centriolin | gga-miR-1782_L+2R-1 |
| CALCOCO2 | calcium binding and coiled-coil domain 2 | PC-5p-71982_65 |
| VWA1 | von Willebrand factor A domain containing 1 | PC-3p-338480_12 |
| CCNL2 | cyclin L2 | PC-3p-457530_9 |
| CCNL2 | cyclin L2 | aca-miR-363-3p_R+1 |
| CCNL2 | cyclin L2 | oan-miR-363-3p_R+1 |
| PPIL2 | peptidylprolyl isomerase (cyclophilin)-like 2 | aca-miR-425-3p_L-2R+1_1ss3AT |
| PPIL2 | peptidylprolyl isomerase (cyclophilin)-like 2 | chi-miR-16b-5p_R+1 |
| PPIL2 | peptidylprolyl isomerase (cyclophilin)-like 2 | gga-miR-16-5p |
| WDR45L | WDR45-like | PC-5p-97606_47 |
| ATP2A3 | ATPase, Ca++ transporting, ubiquitous | PC-3p-338480_12 |
| NFS1 | NFS1 nitrogen fixation 1 homolog (S. cerevisiae) | PC-5p-582426_7 |
| NFS1 | NFS1 nitrogen fixation 1 homolog (S. cerevisiae) | PC-5p-92816_49 |
| NFS1 | NFS1 nitrogen fixation 1 homolog (S. cerevisiae) | aca-miR-18a-5p_R-2 |
| NFS1 | NFS1 nitrogen fixation 1 homolog (S. cerevisiae) | chi-miR-16b-5p_R+1 |
| NFS1 | NFS1 nitrogen fixation 1 homolog (S. cerevisiae) | gga-let-7g-3p_1ss22CT |
| NFS1 | NFS1 nitrogen fixation 1 homolog (S. cerevisiae) | gga-miR-16-5p |
| MYO1F | myosin IF | chi-miR-16b-5p_R+1 |
| MYO1F | myosin IF | gga-miR-16-5p |
| MYO1F | myosin IF | gga-miR-7468-3p_L+3 |
| ANP32B | acidic (leucine-rich) nuclear phosphoprotein 32 family, member B | PC-3p-173245_25 |
| ANP32B | acidic (leucine-rich) nuclear phosphoprotein 32 family, member B | PC-5p-97606_47 |
| ANP32B | acidic (leucine-rich) nuclear phosphoprotein 32 family, member B | tgu-miR-2970-5p_1ss21GT |
| PBRM1 | polybromo 1 | gga-let-7g-3p_1ss22CT |
| PPM1J | protein phosphatase, Mg2+/Mn2+ dependent, 1J | PC-5p-582426_7 |
| PIP4K2B | phosphatidylinositol-5-phosphate 4-kinase, type II, beta | PC-5p-113875_40 |
| MAN2C1 | mannosidase, alpha, class 2C, member 1 | PC-3p-52432_89 |
| MAN2C1 | mannosidase, alpha, class 2C, member 1 | PC-5p-233284_18 |
| ATG16L1 | autophagy related 16-like 1 (S. cerevisiae) | PC-5p-97606_47 |
| CWC25 | CWC25 spliceosome-associated protein homolog (S. cerevisiae) | PC-3p-338480_12 |
| CWC25 | CWC25 spliceosome-associated protein homolog (S. cerevisiae) | PC-5p-71982_65 |
| LOC417345 | uncharacterized LOC417345 | PC-5p-113875_40 |
| PSMD5 | proteasome (prosome, macropain) 26S subunit, non-ATPase, 5 | PC-3p-52432_89 |
| PSMD5 | proteasome (prosome, macropain) 26S subunit, non-ATPase, 5 | PC-5p-97606_47 |
| PSMD5 | proteasome (prosome, macropain) 26S subunit, non-ATPase, 5 | gga-miR-30a-5p_R+2_1ss13CT |
| PSMD5 | proteasome (prosome, macropain) 26S subunit, non-ATPase, 5 | gga-miR-30e-5p_R+5 |
| SLC22A7 | solute carrier family 22 (organic anion transporter), member 7 | aca-miR-18a-5p_R-2 |
| SEC14L1 | SEC14-like 1 (S. cerevisiae) | PC-3p-173245_25 |
| SCAMP2 | secretory carrier membrane protein 2 | gga-miR-1456-3p_R+1 |
| CPSF3L | cleavage and polyadenylation specific factor 3-like | gga-miR-30a-5p_R+2_1ss13CT |
| CPSF3L | cleavage and polyadenylation specific factor 3-like | gga-miR-30e-5p_R+5 |
| ITIH3 | inter-alpha-trypsin inhibitor heavy chain 3 | PC-3p-173245_25 |
| ATP6V1B2 | ATPase, H+ transporting, lysosomal 56/58kDa, V1 subunit B2 | aca-miR-363-3p_R+1 |
| ATP6V1B2 | ATPase, H+ transporting, lysosomal 56/58kDa, V1 subunit B2 | chi-miR-16b-5p_R+1 |
| ATP6V1B2 | ATPase, H+ transporting, lysosomal 56/58kDa, V1 subunit B2 | gga-miR-16-5p |
| ATP6V1B2 | ATPase, H+ transporting, lysosomal 56/58kDa, V1 subunit B2 | gga-miR-30a-5p_R+2_1ss13CT |
| ATP6V1B2 | ATPase, H+ transporting, lysosomal 56/58kDa, V1 subunit B2 | gga-miR-30e-5p_R+5 |
| ATP6V1B2 | ATPase, H+ transporting, lysosomal 56/58kDa, V1 subunit B2 | oan-miR-363-3p_R+1 |
| UBAC1 | UBA domain containing 1 | gga-miR-30a-5p_R+2_1ss13CT |
| UBAC1 | UBA domain containing 1 | gga-miR-30e-5p_R+5 |
| PUSL1 | pseudouridylate synthase-like 1 | tgu-miR-2970-5p_1ss21GT |
| SLC16A1 | solute carrier family 16, member 1 (monocarboxylic acid transporter 1) | PC-3p-173245_25 |
| SLC16A1 | solute carrier family 16, member 1 (monocarboxylic acid transporter 1) | chi-miR-16b-5p_R+1 |
| SLC16A1 | solute carrier family 16, member 1 (monocarboxylic acid transporter 1) | gga-let-7g-3p_1ss22CT |
| SLC16A1 | solute carrier family 16, member 1 (monocarboxylic acid transporter 1) | gga-miR-16-5p |
| E2F4 | E2F transcription factor 4, p107/p130-binding | PC-5p-37716_121 |
| DGKD | diacylglycerol kinase, delta 130kDa | PC-5p-71982_65 |
| DGKD | diacylglycerol kinase, delta 130kDa | PC-5p-92816_49 |
| DGKD | diacylglycerol kinase, delta 130kDa | aca-miR-18a-5p_R-2 |
| DGKD | diacylglycerol kinase, delta 130kDa | gga-miR-33-3p_L+1R+1 |
| LRIG2 | leucine-rich repeats and immunoglobulin-like domains 2 | PC-5p-233284_18 |
| ACSBG2 | acyl-CoA synthetase bubblegum family member 2 | aca-miR-363-3p_R+1 |
| ACSBG2 | acyl-CoA synthetase bubblegum family member 2 | gga-let-7g-3p_1ss22CT |
| ACSBG2 | acyl-CoA synthetase bubblegum family member 2 | oan-miR-363-3p_R+1 |
| ELMO3 | engulfment and cell motility 3 | PC-5p-113875_40 |
| ELMO3 | engulfment and cell motility 3 | aca-miR-425-3p_L-2R+1_1ss3AT |
| ODZ2 | odz, odd Oz/ten-m homolog 2 (Drosophila) | PC-3p-338480_12 |
| ODZ2 | odz, odd Oz/ten-m homolog 2 (Drosophila) | gga-let-7g-3p_1ss22CT |
| ODZ2 | odz, odd Oz/ten-m homolog 2 (Drosophila) | gga-miR-1a-3p_R+1_1ss2GT |
| MAPK8IP3 | mitogen-activated protein kinase 8 interacting protein 3 | aca-miR-425-3p_L-2R+1_1ss3AT |
| MAPK8IP3 | mitogen-activated protein kinase 8 interacting protein 3 | gga-miR-1456-3p_R+1 |
| METTL23 | methyltransferase like 23 | aca-miR-425-3p_L-2R+1_1ss3AT |
| MYL10 | myosin, light chain 10, regulatory | PC-5p-92816_49 |
| MYL10 | myosin, light chain 10, regulatory | gga-miR-1a-3p_R+1_1ss2GT |
| MYL10 | myosin, light chain 10, regulatory | gga-miR-33-3p_L+1R+1 |
| UQCC | ubiquinol-cytochrome c reductase complex chaperone | PC-3p-457530_9 |
| UQCC | ubiquinol-cytochrome c reductase complex chaperone | tgu-miR-2970-5p_1ss21GT |
| JMJD6 | jumonji domain containing 6 | PC-5p-37716_121 |
| JMJD6 | jumonji domain containing 6 | PC-5p-97606_47 |
| JMJD6 | jumonji domain containing 6 | aca-miR-18a-5p_R-2 |
| JMJD6 | jumonji domain containing 6 | gga-miR-30a-5p_R+2_1ss13CT |
| JMJD6 | jumonji domain containing 6 | gga-miR-30e-5p_R+5 |
| EIF6 | eukaryotic translation initiation factor 6 | PC-5p-582426_7 |
| EIF6 | eukaryotic translation initiation factor 6 | gga-miR-1677-3p_L+1 |
| WWC1 | WW and C2 domain containing 1 | gga-miR-1805-5p_L-1 |
| GLYR1 | glyoxylate reductase 1 homolog (Arabidopsis) | chi-miR-16b-5p_R+1 |
| GLYR1 | glyoxylate reductase 1 homolog (Arabidopsis) | gga-miR-16-5p |
| PRKRIP1 | PRKR interacting protein 1 (IL11 inducible) | PC-5p-71982_65 |
| ORAI2 | ORAI calcium release-activated calcium modulator 2 | PC-3p-173245_25 |
| ORAI2 | ORAI calcium release-activated calcium modulator 2 | PC-5p-113875_40 |
| ORAI2 | ORAI calcium release-activated calcium modulator 2 | PC-5p-582426_7 |
| ORAI2 | ORAI calcium release-activated calcium modulator 2 | PC-5p-71982_65 |
| ORAI2 | ORAI calcium release-activated calcium modulator 2 | gga-miR-30a-5p_R+2_1ss13CT |
| ORAI2 | ORAI calcium release-activated calcium modulator 2 | gga-miR-30e-5p_R+5 |
| ORAI2 | ORAI calcium release-activated calcium modulator 2 | hsa-miR-483-3p_L-1R+2 |
| PHTF1 | putative homeodomain transcription factor 1 | gga-miR-1677-3p_L+1 |
| LRWD1 | leucine-rich repeats and WD repeat domain containing 1 | tgu-miR-2970-5p_1ss21GT |
| RASA4 | RAS p21 protein activator 4 | PC-5p-71982_65 |
| ST6GALNAC1 | ST6 (alpha-N-acetyl-neuraminyl-2,3-beta-galactosyl-1,3)-N-acetylgalactosaminide alpha-2,6-sialyltransferase 1 | PC-5p-113875_40 |
| ST6GALNAC1 | ST6 (alpha-N-acetyl-neuraminyl-2,3-beta-galactosyl-1,3)-N-acetylgalactosaminide alpha-2,6-sialyltransferase 1 | PC-5p-97606_47 |
| SDF4 | stromal cell derived factor 4 | PC-5p-92816_49 |
| SDF4 | stromal cell derived factor 4 | chi-miR-16b-5p_R+1 |
| SDF4 | stromal cell derived factor 4 | gga-miR-16-5p |
| SDF4 | stromal cell derived factor 4 | gga-miR-30a-5p_R+2_1ss13CT |
| SDF4 | stromal cell derived factor 4 | gga-miR-30e-5p_R+5 |
| TNFRSF18 | tumor necrosis factor receptor superfamily, member 18 | PC-5p-97606_47 |
| TNFRSF18 | tumor necrosis factor receptor superfamily, member 18 | gga-miR-34b-5p_L-1R+1 |
| TNFRSF18 | tumor necrosis factor receptor superfamily, member 18 | gga-miR-7468-3p_L+3 |
| TNFRSF18 | tumor necrosis factor receptor superfamily, member 18 | hsa-miR-483-3p_L-1R+2 |
| ST6GALNAC2 | ST6 (alpha-N-acetyl-neuraminyl-2,3-beta-galactosyl-1,3)-N-acetylgalactosaminide alpha-2,6-sialyltransferase 2 | PC-5p-582426_7 |
| VAV3 | vav 3 guanine nucleotide exchange factor | PC-3p-457530_9 |
| VAV3 | vav 3 guanine nucleotide exchange factor | gga-miR-1782_L+2R-1 |
| WAPAL | wings apart-like homolog (Drosophila) | chi-miR-16b-5p_R+1 |
| WAPAL | wings apart-like homolog (Drosophila) | gga-miR-16-5p |
| WAPAL | wings apart-like homolog (Drosophila) | gga-miR-1677-3p_L+1 |
| DYNLRB1 | dynein, light chain, roadblock-type 1 | gga-let-7g-3p_1ss22CT |
| GNB1L | guanine nucleotide binding protein (G protein), beta polypeptide 1-like | chi-miR-16b-5p_R+1 |
| GNB1L | guanine nucleotide binding protein (G protein), beta polypeptide 1-like | gga-miR-16-5p |
| GNB1L | guanine nucleotide binding protein (G protein), beta polypeptide 1-like | gga-miR-1677-3p_L+1 |
| GNB1L | guanine nucleotide binding protein (G protein), beta polypeptide 1-like | gga-miR-1782_L+2R-1 |
| GNB1L | guanine nucleotide binding protein (G protein), beta polypeptide 1-like | hsa-miR-483-3p_L-1R+2 |
| HEXA | hexosaminidase A (alpha polypeptide) | gga-miR-34b-5p_L-1R+1 |
| CCDC99 | coiled-coil domain containing 99 | aca-miR-18a-5p_R-2 |
| PMPCA | peptidase (mitochondrial processing) alpha | chi-miR-16b-5p_R+1 |
| PMPCA | peptidase (mitochondrial processing) alpha | gga-miR-16-5p |
| PTBP1 | polypyrimidine tract binding protein 1 | PC-5p-97606_47 |
| PTBP1 | polypyrimidine tract binding protein 1 | gga-miR-1782_L+2R-1 |
| PTBP1 | polypyrimidine tract binding protein 1 | gga-miR-7468-3p_L+3 |
| PTBP1 | polypyrimidine tract binding protein 1 | tgu-miR-2970-5p_1ss21GT |
| MED16 | mediator complex subunit 16 | PC-5p-97606_47 |
| R3HDM4 | chromosome 28 open reading frame, human C19orf22 | PC-5p-233284_18 |
| STXBP3 | syntaxin binding protein 3 | chi-miR-16b-5p_R+1 |
| STXBP3 | syntaxin binding protein 3 | gga-miR-16-5p |
| RNF157 | ring finger protein 157 | gga-miR-34b-5p_L-1R+1 |
| SMU1 | smu-1 suppressor of mec-8 and unc-52 homolog (C. elegans) | PC-5p-582426_7 |
| GLUD1 | glutamate dehydrogenase 1 | PC-5p-582426_7 |
| COMT | catechol-O-methyltransferase | tgu-miR-2970-5p_1ss21GT |
| ARVCF | armadillo repeat gene deleted in velocardiofacial syndrome | PC-5p-92816_49 |
| ARVCF | armadillo repeat gene deleted in velocardiofacial syndrome | gga-miR-34b-5p_L-1R+1 |
| FAM86A | family with sequence similarity 86, member A | PC-5p-71982_65 |
| FAM86A | family with sequence similarity 86, member A | gga-let-7g-3p_1ss22CT |
| FAM86A | family with sequence similarity 86, member A | gga-miR-1329-3p |
| GPRIN2 | G protein regulated inducer of neurite outgrowth 2 | tgu-miR-2970-5p_1ss21GT |
| C15H22ORF25 | chromosome 15 open reading frame, human C22orf25 | PC-3p-457530_9 |
| C15H22ORF25 | chromosome 15 open reading frame, human C22orf25 | aca-miR-363-3p_R+1 |
| C15H22ORF25 | chromosome 15 open reading frame, human C22orf25 | oan-miR-363-3p_R+1 |
| POLR2D | polymerase (RNA) II (DNA directed) polypeptide D | PC-5p-582426_7 |
| POLR2D | polymerase (RNA) II (DNA directed) polypeptide D | PC-5p-71982_65 |
| POLR2D | polymerase (RNA) II (DNA directed) polypeptide D | PC-5p-92816_49 |
| POLR2D | polymerase (RNA) II (DNA directed) polypeptide D | gga-miR-34b-5p_L-1R+1 |
| SRP68 | signal recognition particle 68kDa | PC-3p-457530_9 |
| SAP130 | Sin3A-associated protein, 130kDa | PC-5p-71982_65 |
| GPSM2 | G-protein signaling modulator 2 | gga-let-7g-3p_1ss22CT |
| LOC415324 | epididymal protein-like | gga-miR-1782_L+2R-1 |
| LOC100859653 | 6-phosphofructo-2-kinase/fructose-2,6-biphosphatase 4-like | chi-miR-16b-5p_R+1 |
| LOC100859653 | 6-phosphofructo-2-kinase/fructose-2,6-biphosphatase 4-like | gga-miR-16-5p |
| LOC100859653 | 6-phosphofructo-2-kinase/fructose-2,6-biphosphatase 4-like | gga-miR-1782_L+2R-1 |
| LOC415325 | uncharacterized LOC415325 | gga-miR-34b-5p_L-1R+1 |
| CLCC1 | chloride channel CLIC-like 1 | PC-5p-71982_65 |
| CLCC1 | chloride channel CLIC-like 1 | PC-5p-92816_49 |
| PARG | poly (ADP-ribose) glycohydrolase | gga-miR-1a-3p_R+1_1ss2GT |
| KCNMB1 | potassium large conductance calcium-activated channel, subfamily M, beta member 1 | PC-3p-173245_25 |
| KCNMB1 | potassium large conductance calcium-activated channel, subfamily M, beta member 1 | gga-miR-7468-3p_L+3 |
| ZDHHC8 | zinc finger, DHHC-type containing 8 | PC-5p-71982_65 |
| CPEB1 | cytoplasmic polyadenylation element binding protein 1 | PC-5p-37716_121 |
| CPEB1 | cytoplasmic polyadenylation element binding protein 1 | gga-miR-1a-3p_R+1_1ss2GT |
| GOLGA3 | golgin A3 | PC-3p-457530_9 |
| GOLGA3 | golgin A3 | PC-5p-233284_18 |
| ACOX1 | acyl-CoA oxidase 1, palmitoyl | PC-3p-173245_25 |
| ACOX1 | acyl-CoA oxidase 1, palmitoyl | PC-5p-71982_65 |
| ACOX1 | acyl-CoA oxidase 1, palmitoyl | PC-5p-97606_47 |
| ACOX1 | acyl-CoA oxidase 1, palmitoyl | aca-miR-363-3p_R+1 |
| ACOX1 | acyl-CoA oxidase 1, palmitoyl | chi-miR-16b-5p_R+1 |
| ACOX1 | acyl-CoA oxidase 1, palmitoyl | gga-miR-16-5p |
| ACOX1 | acyl-CoA oxidase 1, palmitoyl | oan-miR-363-3p_R+1 |
| ACO1 | aconitase 1, soluble | PC-5p-233284_18 |
| ACO1 | aconitase 1, soluble | gga-miR-1677-3p_L+1 |
| PLEKHB2 | pleckstrin homology domain containing, family B (evectins) member 2 | PC-5p-582426_7 |
| PLEKHB2 | pleckstrin homology domain containing, family B (evectins) member 2 | aca-miR-363-3p_R+1 |
| PLEKHB2 | pleckstrin homology domain containing, family B (evectins) member 2 | chi-miR-16b-5p_R+1 |
| PLEKHB2 | pleckstrin homology domain containing, family B (evectins) member 2 | gga-miR-16-5p |
| PLEKHB2 | pleckstrin homology domain containing, family B (evectins) member 2 | oan-miR-363-3p_R+1 |
| CHFR | checkpoint with forkhead and ring finger domains, E3 ubiquitin protein ligase | gga-miR-1805-5p_L-1 |
| NR5A2 | nuclear receptor subfamily 5, group A, member 2 | PC-5p-233284_18 |
| NR5A2 | nuclear receptor subfamily 5, group A, member 2 | gga-miR-30a-5p_R+2_1ss13CT |
| NR5A2 | nuclear receptor subfamily 5, group A, member 2 | gga-miR-30e-5p_R+5 |
| HSD17B4 | hydroxysteroid (17-beta) dehydrogenase 4 | aca-miR-363-3p_R+1 |
| HSD17B4 | hydroxysteroid (17-beta) dehydrogenase 4 | oan-miR-363-3p_R+1 |
| SNX22 | sorting nexin 22 | tgu-miR-2970-5p_1ss21GT |
| WBP2 | WW domain binding protein 2 | PC-5p-71982_65 |
| WBP2 | WW domain binding protein 2 | hsa-miR-483-3p_L-1R+2 |
| NOC2L | nucleolar complex associated 2 homolog (S. cerevisiae) | PC-5p-113875_40 |
| RFFL | ring finger and FYVE-like domain containing E3 ubiquitin protein ligase | aca-miR-18a-5p_R-2 |
| RFFL | ring finger and FYVE-like domain containing E3 ubiquitin protein ligase | chi-miR-16b-5p_R+1 |
| RFFL | ring finger and FYVE-like domain containing E3 ubiquitin protein ligase | gga-let-7g-3p_1ss22CT |
| RFFL | ring finger and FYVE-like domain containing E3 ubiquitin protein ligase | gga-miR-16-5p |
| RFFL | ring finger and FYVE-like domain containing E3 ubiquitin protein ligase | gga-miR-1782_L+2R-1 |
| DMXL1 | Dmx-like 1 | PC-5p-97606_47 |
| DMXL1 | Dmx-like 1 | aca-miR-425-3p_L-2R+1_1ss3AT |
| FBXW11 | F-box and WD repeat domain containing 11 | PC-5p-113875_40 |
| FBXW11 | F-box and WD repeat domain containing 11 | gga-let-7g-3p_1ss22CT |
| FBXW11 | F-box and WD repeat domain containing 11 | gga-miR-1456-5p_L-1 |
| FBXW11 | F-box and WD repeat domain containing 11 | gga-miR-7468-3p_L+3 |
| AGO3 | eukaryotic translation initiation factor 2C, 3 | gga-miR-30a-5p_R+2_1ss13CT |
| AGO3 | eukaryotic translation initiation factor 2C, 3 | gga-miR-30e-5p_R+5 |
| DTWD2 | DTW domain containing 2 | PC-5p-582426_7 |
| DTWD2 | DTW domain containing 2 | PC-5p-97606_47 |
| HEMK1 | HemK methyltransferase family member 1 | gga-miR-1456-5p_L-1 |
| HEMK1 | HemK methyltransferase family member 1 | hsa-miR-483-3p_L-1R+2 |
| ETF1 | eukaryotic translation termination factor 1 | gga-miR-33-3p_L+1R+1 |
| KLHL6 | kelch-like 6 (Drosophila) | PC-5p-113875_40 |
| KLHL6 | kelch-like 6 (Drosophila) | gga-let-7g-3p_1ss22CT |
| KLHL6 | kelch-like 6 (Drosophila) | gga-miR-1456-5p_L-1 |
| TMEM194B | transmembrane protein 194B | PC-5p-37716_121 |
| TMEM194B | transmembrane protein 194B | PC-5p-97606_47 |
| TMEM194B | transmembrane protein 194B | gga-miR-1456-5p_L-1 |
| EP400 | E1A binding protein p400 | PC-5p-113875_40 |
| EP400 | E1A binding protein p400 | chi-miR-16b-5p_R+1 |
| EP400 | E1A binding protein p400 | gga-miR-16-5p |
| MAPKAPK3 | mitogen-activated protein kinase-activated protein kinase 3 | PC-5p-113875_40 |
| TRAPPC3 | trafficking protein particle complex 3 | PC-5p-92816_49 |
| TRAPPC3 | trafficking protein particle complex 3 | tgu-miR-2970-5p_1ss21GT |
| SPSB3 | splA/ryanodine receptor domain and SOCS box containing 3 | PC-5p-113875_40 |
| H3F3B | H3 histone, family 3B | gga-miR-1805-5p_L-1 |
| H3F3B | H3 histone, family 3B | gga-miR-1a-3p_R+1_1ss2GT |
| H3F3B | H3 histone, family 3B | tgu-miR-2970-5p_1ss21GT |
| LIG3 | ligase III, DNA, ATP-dependent | PC-5p-92816_49 |
| CHAT | choline O-acetyltransferase | PC-3p-338480_12 |
| CNOT1 | CCR4-NOT transcription complex subunit 1-like | PC-5p-37716_121 |
| CNOT1 | CCR4-NOT transcription complex subunit 1-like | gga-miR-1805-5p_L-1 |
| ADPRHL2 | ADP-ribosylhydrolase like 2 | gga-miR-1782_L+2R-1 |
| HIBCH | 3-hydroxyisobutyryl-CoA hydrolase | PC-5p-233284_18 |
| HIBCH | 3-hydroxyisobutyryl-CoA hydrolase | aca-miR-363-3p_R+1 |
| HIBCH | 3-hydroxyisobutyryl-CoA hydrolase | chi-miR-16b-5p_R+1 |
| HIBCH | 3-hydroxyisobutyryl-CoA hydrolase | gga-miR-16-5p |
| HIBCH | 3-hydroxyisobutyryl-CoA hydrolase | oan-miR-363-3p_R+1 |
| NUBP2 | nucleotide binding protein 2 | chi-miR-16b-5p_R+1 |
| NUBP2 | nucleotide binding protein 2 | gga-miR-16-5p |
| NUBP2 | nucleotide binding protein 2 | gga-miR-1782_L+2R-1 |
| SORD | sorbitol dehydrogenase | gga-miR-1a-3p_R+1_1ss2GT |
| SORD | sorbitol dehydrogenase | gga-miR-30a-5p_R+2_1ss13CT |
| SORD | sorbitol dehydrogenase | gga-miR-30e-5p_R+5 |
| SORD | sorbitol dehydrogenase | gga-miR-33-3p_L+1R+1 |
| GOT2 | glutamic-oxaloacetic transaminase 2, mitochondrial (aspartate aminotransferase 2) | PC-3p-52432_89 |
| CCL1 | chemokine (C-C motif) ligand 1 | PC-5p-582426_7 |
| CCL1 | chemokine (C-C motif) ligand 1 | gga-let-7g-3p_1ss22CT |
| AP3S1 | adaptor-related protein complex 3, sigma 1 subunit | PC-5p-113875_40 |
| AP3S1 | adaptor-related protein complex 3, sigma 1 subunit | gga-miR-30a-5p_R+2_1ss13CT |
| AP3S1 | adaptor-related protein complex 3, sigma 1 subunit | gga-miR-30e-5p_R+5 |
| AP3S1 | adaptor-related protein complex 3, sigma 1 subunit | gga-miR-7468-3p_L+3 |
| AP3S1 | adaptor-related protein complex 3, sigma 1 subunit | tgu-miR-2970-5p_1ss21GT |
| ATG12 | autophagy related 12 | PC-3p-52432_89 |
| ATG12 | autophagy related 12 | tgu-miR-2970-5p_1ss21GT |
| PMS1 | PMS1 postmeiotic segregation increased 1 (S. cerevisiae) | gga-miR-33-3p_L+1R+1 |
| HSPA9 | heat shock 70kDa protein 9 (mortalin) | PC-5p-71982_65 |
| HSPA9 | heat shock 70kDa protein 9 (mortalin) | PC-5p-97606_47 |
| SEC16A | SEC16 homolog A (S. cerevisiae) | PC-3p-457530_9 |
| ENO1 | enolase 1, (alpha) | chi-miR-16b-5p_R+1 |
| ENO1 | enolase 1, (alpha) | gga-miR-16-5p |
| MRPS17 | mitochondrial ribosomal protein S17 | PC-5p-113875_40 |
| OSGEPL1 | O-sialoglycoprotein endopeptidase-like 1 | PC-3p-173245_25 |
| GBAS | glioblastoma amplified sequence | gga-let-7g-3p_1ss22CT |
| MPP3 | membrane protein, palmitoylated 3 (MAGUK p55 subfamily member 3) | gga-miR-34b-5p_L-1R+1 |
| VPRBP | Vpr (HIV-1) binding protein | PC-5p-113875_40 |
| ULK1 | unc-51-like kinase 1 (C. elegans) | tgu-miR-2970-5p_1ss21GT |
| C10ORF58 | chromosome 6 open reading frame, human C10orf58 | PC-5p-233284_18 |
| C10ORF58 | chromosome 6 open reading frame, human C10orf58 | aca-miR-363-3p_R+1 |
| C10ORF58 | chromosome 6 open reading frame, human C10orf58 | oan-miR-363-3p_R+1 |
| C10ORF58 | chromosome 6 open reading frame, human C10orf58 | tgu-miR-2970-5p_1ss21GT |
| SMN | survival of motor neuron | gga-let-7g-3p_1ss22CT |
| SMN | survival of motor neuron | gga-miR-7468-3p_L+3 |
| CFH | complement factor H | PC-5p-92816_49 |
| CFH | complement factor H | gga-miR-1a-3p_R+1_1ss2GT |
| CFH | complement factor H | gga-miR-30a-5p_R+2_1ss13CT |
| CFH | complement factor H | gga-miR-30e-5p_R+5 |
| DYDC1 | DPY30 domain containing 1 | PC-3p-338480_12 |
| DYDC1 | DPY30 domain containing 1 | PC-5p-582426_7 |
| AQP3 | aquaporin 3 (Gill blood group) | hsa-miR-483-3p_L-1R+2 |
| ABCA7 | ATP-binding cassette, sub-family A (ABC1), member 7 | PC-3p-52432_89 |
| VAC14 | Vac14 homolog (S. cerevisiae) | PC-5p-71982_65 |
| CDC73 | cell division cycle 73, Paf1/RNA polymerase II complex component, homolog (S. cerevisiae) | gga-miR-1456-5p_L-1 |
| GPR157 | G protein-coupled receptor 157 | PC-5p-97606_47 |
| MAT1A | methionine adenosyltransferase I, alpha | PC-5p-582426_7 |
| MAT1A | methionine adenosyltransferase I, alpha | chi-miR-16b-5p_R+1 |
| MAT1A | methionine adenosyltransferase I, alpha | gga-miR-16-5p |
| CRYM | crystallin, mu | chi-miR-16b-5p_R+1 |
| CRYM | crystallin, mu | gga-miR-16-5p |
| PAIP2 | poly(A) binding protein interacting protein 2 | PC-5p-92816_49 |
| PAIP2 | poly(A) binding protein interacting protein 2 | PC-5p-97606_47 |
| PAIP2 | poly(A) binding protein interacting protein 2 | gga-miR-1805-5p_L-1 |
| SPSB1 | splA/ryanodine receptor domain and SOCS box containing 1 | gga-miR-1677-3p_L+1 |
| OLFM1 | olfactomedin 1 | tgu-miR-2970-5p_1ss21GT |
| SLC25A33 | solute carrier family 25 (pyrimidine nucleotide carrier), member 33 | chi-miR-16b-5p_R+1 |
| SLC25A33 | solute carrier family 25 (pyrimidine nucleotide carrier), member 33 | gga-miR-16-5p |
| SLC25A33 | solute carrier family 25 (pyrimidine nucleotide carrier), member 33 | gga-miR-7468-3p_L+3 |
| SLC25A33 | solute carrier family 25 (pyrimidine nucleotide carrier), member 33 | tgu-miR-2970-5p_1ss21GT |
| UCHL5 | ubiquitin carboxyl-terminal hydrolase L5 | gga-miR-1782_L+2R-1 |
| MCCC2 | methylcrotonoyl-CoA carboxylase 2 (beta) | gga-miR-1805-5p_L-1 |
| GJA4 | gap junction protein, alpha 4, 37kDa | PC-3p-52432_89 |
| RGS2 | regulator of G-protein signaling 2, 24kDa | PC-5p-582426_7 |
| RGS4 | regulator of G-protein signaling 4 | PC-5p-582426_7 |
| RGS4 | regulator of G-protein signaling 4 | PC-5p-71982_65 |
| RGS4 | regulator of G-protein signaling 4 | PC-5p-97606_47 |
| RGS4 | regulator of G-protein signaling 4 | chi-miR-16b-5p_R+1 |
| RGS4 | regulator of G-protein signaling 4 | gga-miR-16-5p |
| RAN | RAN, member RAS oncogene family | PC-5p-71982_65 |
| KTN1 | kinectin 1 (kinesin receptor) | PC-5p-92816_49 |
| KTN1 | kinectin 1 (kinesin receptor) | gga-miR-1677-3p_L+1 |
| KTN1 | kinectin 1 (kinesin receptor) | gga-miR-33-3p_L+1R+1 |
| APH1A | anterior pharynx defective 1 homolog A (C. elegans) | chi-miR-16b-5p_R+1 |
| APH1A | anterior pharynx defective 1 homolog A (C. elegans) | gga-miR-16-5p |
| PIK3CD | phosphoinositide-3-kinase, catalytic, delta polypeptide | PC-3p-52432_89 |
| PIK3CD | phosphoinositide-3-kinase, catalytic, delta polypeptide | PC-5p-97606_47 |
| PIK3CD | phosphoinositide-3-kinase, catalytic, delta polypeptide | gga-miR-1677-3p_L+1 |
| POLR2E | polymerase (RNA) II (DNA directed) polypeptide E, 25kDa | PC-5p-97606_47 |
| POLR2E | polymerase (RNA) II (DNA directed) polypeptide E, 25kDa | aca-miR-18a-5p_R-2 |
| CZH9ORF100 | chromosome Z open reading frame, human C9orf100 | PC-5p-113875_40 |
| CZH9ORF100 | chromosome Z open reading frame, human C9orf100 | gga-miR-1a-3p_R+1_1ss2GT |
| COG4 | component of oligomeric golgi complex 4 | PC-3p-338480_12 |
| COG4 | component of oligomeric golgi complex 4 | gga-miR-1782_L+2R-1 |
| DEF6 | differentially expressed in FDCP 6 homolog (mouse) | PC-5p-71982_65 |
| DEF6 | differentially expressed in FDCP 6 homolog (mouse) | gga-miR-34b-5p_L-1R+1 |
| LZIC | leucine zipper and CTNNBIP1 domain containing | gga-let-7g-3p_1ss22CT |
| LZIC | leucine zipper and CTNNBIP1 domain containing | tgu-miR-2970-5p_1ss21GT |
| KCTD7 | potassium channel tetramerisation domain containing 7 | PC-3p-457530_9 |
| KCTD7 | potassium channel tetramerisation domain containing 7 | aca-miR-18a-5p_R-2 |
| KCTD7 | potassium channel tetramerisation domain containing 7 | aca-miR-363-3p_R+1 |
| KCTD7 | potassium channel tetramerisation domain containing 7 | chi-miR-16b-5p_R+1 |
| KCTD7 | potassium channel tetramerisation domain containing 7 | gga-miR-16-5p |
| KCTD7 | potassium channel tetramerisation domain containing 7 | gga-miR-30a-5p_R+2_1ss13CT |
| KCTD7 | potassium channel tetramerisation domain containing 7 | gga-miR-30e-5p_R+5 |
| KCTD7 | potassium channel tetramerisation domain containing 7 | hsa-miR-483-3p_L-1R+2 |
| KCTD7 | potassium channel tetramerisation domain containing 7 | oan-miR-363-3p_R+1 |
| BMS1 | BMS1 homolog, ribosome assembly protein (yeast) | aca-miR-363-3p_R+1 |
| BMS1 | BMS1 homolog, ribosome assembly protein (yeast) | oan-miR-363-3p_R+1 |
| RXRA | retinoid X receptor, alpha | hsa-miR-483-3p_L-1R+2 |
| ZNF76 | zinc finger protein 76 | PC-5p-71982_65 |
| HDAC3 | histone deacetylase 3 | PC-3p-52432_89 |
| TRPC1 | transient receptor potential cation channel, subfamily C, member 1 | chi-miR-16b-5p_R+1 |
| TRPC1 | transient receptor potential cation channel, subfamily C, member 1 | gga-miR-16-5p |
| PLS1 | plastin 1 | PC-3p-338480_12 |
| C19H17ORF85 | chromosome 19 open reading frame, human C17orf85 | PC-5p-92816_49 |
| C19H17ORF85 | chromosome 19 open reading frame, human C17orf85 | aca-miR-18a-5p_R-2 |
| C19H17ORF85 | chromosome 19 open reading frame, human C17orf85 | gga-miR-1a-3p_R+1_1ss2GT |
| ITGAV | integrin, alpha V | PC-3p-173245_25 |
| ITGAV | integrin, alpha V | tgu-miR-2970-5p_1ss21GT |
| WDR5 | WD repeat domain 5 | PC-3p-173245_25 |
| WDR5 | WD repeat domain 5 | PC-5p-113875_40 |
| WDR5 | WD repeat domain 5 | PC-5p-97606_47 |
| ATR | ataxia telangiectasia and Rad3 related | gga-let-7g-3p_1ss22CT |
| ANXA11 | annexin A11 | PC-3p-457530_9 |
| ZC3H15 | zinc finger CCCH-type containing 15 | PC-3p-173245_25 |
| ZC3H15 | zinc finger CCCH-type containing 15 | gga-miR-1782_L+2R-1 |
| SCUBE3 | signal peptide, CUB domain, EGF-like 3 | PC-3p-338480_12 |
| NUP35 | nucleoporin 35kDa | PC-5p-233284_18 |
| SLC15A4 | solute carrier family 15, member 4 | PC-3p-52432_89 |
| SLC15A4 | solute carrier family 15, member 4 | gga-miR-30a-5p_R+2_1ss13CT |
| SLC15A4 | solute carrier family 15, member 4 | gga-miR-30e-5p_R+5 |
| DNAJC9 | DnaJ (Hsp40) homolog, subfamily C, member 9 | PC-3p-173245_25 |
| DNAJC9 | DnaJ (Hsp40) homolog, subfamily C, member 9 | PC-5p-582426_7 |
| DNAJC9 | DnaJ (Hsp40) homolog, subfamily C, member 9 | PC-5p-97606_47 |
| TFAM | transcription factor A, mitochondrial | chi-miR-16b-5p_R+1 |
| TFAM | transcription factor A, mitochondrial | gga-miR-16-5p |
| XPO6 | exportin 6 | PC-3p-282885_15 |
| XPO6 | exportin 6 | PC-5p-113875_40 |
| SLC16A3 | solute carrier family 16, member 3 (monocarboxylic acid transporter 4) | PC-5p-113875_40 |
| SLC16A3 | solute carrier family 16, member 3 (monocarboxylic acid transporter 4) | gga-let-7g-3p_1ss22CT |
| GK5 | glycerol kinase 5 (putative) | gga-miR-30a-5p_R+2_1ss13CT |
| GK5 | glycerol kinase 5 (putative) | gga-miR-30e-5p_R+5 |
| TAF11 | TAF11 RNA polymerase II, TATA box binding protein (TBP)-associated factor, 28kDa | PC-5p-97606_47 |
| TAF11 | TAF11 RNA polymerase II, TATA box binding protein (TBP)-associated factor, 28kDa | gga-let-7g-3p_1ss22CT |
| TAF11 | TAF11 RNA polymerase II, TATA box binding protein (TBP)-associated factor, 28kDa | gga-miR-1782_L+2R-1 |
| TAF11 | TAF11 RNA polymerase II, TATA box binding protein (TBP)-associated factor, 28kDa | gga-miR-7468-3p_L+3 |
| INPP5K | inositol polyphosphate-5-phosphatase K | PC-5p-233284_18 |
| INPP5K | inositol polyphosphate-5-phosphatase K | aca-miR-18a-5p_R-2 |
| INPP5K | inositol polyphosphate-5-phosphatase K | gga-miR-30a-5p_R+2_1ss13CT |
| INPP5K | inositol polyphosphate-5-phosphatase K | gga-miR-30e-5p_R+5 |
| RFWD3 | ring finger and WD repeat domain 3 | PC-3p-52432_89 |
| RFWD3 | ring finger and WD repeat domain 3 | PC-5p-37716_121 |
| RFWD3 | ring finger and WD repeat domain 3 | aca-miR-18a-5p_R-2 |
| FRZB | frizzled-related protein | PC-5p-92816_49 |
| CRIP1 | cysteine-rich protein 1 (intestinal) | gga-miR-1456-5p_L-1 |
| C26H6ORF106 | chromosome 26 open reading frame, human C6orf106 | PC-5p-97606_47 |
| C26H6ORF106 | chromosome 26 open reading frame, human C6orf106 | gga-miR-1456-5p_L-1 |
| C26H6ORF106 | chromosome 26 open reading frame, human C6orf106 | gga-miR-1677-3p_L+1 |
| MED23 | mediator complex subunit 23 | PC-3p-338480_12 |
| MED23 | mediator complex subunit 23 | PC-5p-97606_47 |
| MAP2 | microtubule-associated protein 2 | gga-miR-1a-3p_R+1_1ss2GT |
| PDXDC1 | pyridoxal-dependent decarboxylase domain containing 1 | gga-let-7g-3p_1ss22CT |
| WDR59 | WD repeat domain 59 | PC-5p-233284_18 |
| WDR59 | WD repeat domain 59 | PC-5p-71982_65 |
| VAT1 | vesicle amine transport protein 1 homolog (T. californica) | chi-miR-16b-5p_R+1 |
| VAT1 | vesicle amine transport protein 1 homolog (T. californica) | gga-miR-16-5p |
| DFFA | DNA fragmentation factor, 45kDa, alpha polypeptide | PC-3p-173245_25 |
| NTAN1 | N-terminal asparagine amidase | chi-miR-16b-5p_R+1 |
| NTAN1 | N-terminal asparagine amidase | gga-miR-16-5p |
| GRM4 | glutamate receptor, metabotropic 4 | PC-5p-97606_47 |
| GRM4 | glutamate receptor, metabotropic 4 | gga-miR-1456-3p_R+1 |
| RFNG | RFNG O-fucosylpeptide 3-beta-N-acetylglucosaminyltransferase | PC-3p-52432_89 |
| RFNG | RFNG O-fucosylpeptide 3-beta-N-acetylglucosaminyltransferase | gga-let-7g-3p_1ss22CT |
| RCN2 | reticulocalbin 2, EF-hand calcium binding domain | gga-let-7g-3p_1ss22CT |
| RCN2 | reticulocalbin 2, EF-hand calcium binding domain | gga-miR-1a-3p_R+1_1ss2GT |
| RCN2 | reticulocalbin 2, EF-hand calcium binding domain | tgu-miR-2970-5p_1ss21GT |
| CTNNA3 | catenin (cadherin-associated protein), alpha 3 | PC-5p-97606_47 |
| RHO | rhodopsin (opsin 2, rod pigment) (retinitis pigmentosa 4, autosomal dominant) | gga-let-7g-3p_1ss22CT |
| RASA2 | RAS p21 protein activator 2 | PC-3p-457530_9 |
| RASA2 | RAS p21 protein activator 2 | gga-let-7g-3p_1ss22CT |
| RASA2 | RAS p21 protein activator 2 | gga-miR-30a-5p_R+2_1ss13CT |
| RASA2 | RAS p21 protein activator 2 | gga-miR-30e-5p_R+5 |
| LOC100857356 | zinc finger protein 532-like | PC-5p-233284_18 |
| LOC100857356 | zinc finger protein 532-like | gga-miR-7468-3p_L+3 |
| TMEM170A | transmembrane protein 170A | gga-miR-1456-5p_L-1 |
| ERGIC1 | endoplasmic reticulum-golgi intermediate compartment (ERGIC) 1 | gga-miR-7468-3p_L+3 |
| PHACTR4 | phosphatase and actin regulator 4 | gga-miR-1456-3p_R+1 |
| LRRC45 | leucine rich repeat containing 45 | PC-3p-173245_25 |
| LRRC45 | leucine rich repeat containing 45 | PC-5p-113875_40 |
| LRRC45 | leucine rich repeat containing 45 | hsa-miR-483-3p_L-1R+2 |
| SCAPER | S-phase cyclin A-associated protein in the ER | PC-5p-71982_65 |
| ACADL | acyl-CoA dehydrogenase, long chain | tgu-miR-2970-5p_1ss21GT |
| ENPP3 | ectonucleotide pyrophosphatase/phosphodiesterase 3 | gga-miR-1a-3p_R+1_1ss2GT |
| ENPP3 | ectonucleotide pyrophosphatase/phosphodiesterase 3 | gga-miR-7468-3p_L+3 |
| ENPP1 | ectonucleotide pyrophosphatase/phosphodiesterase 1 | gga-miR-33-3p_L+1R+1 |
| TRAIP | TRAF interacting protein | PC-3p-52432_89 |
| TRAIP | TRAF interacting protein | tgu-miR-2970-5p_1ss21GT |
| BOD1 | biorientation of chromosomes in cell division 1 | PC-5p-233284_18 |
| TARDBP | TAR DNA binding protein | gga-miR-1782_L+2R-1 |
| BRI3BP | BRI3 binding protein | PC-5p-92816_49 |
| NEDD4L | neural precursor cell expressed, developmentally down-regulated 4-like, E3 ubiquitin protein ligase | gga-miR-30a-5p_R+2_1ss13CT |
| NEDD4L | neural precursor cell expressed, developmentally down-regulated 4-like, E3 ubiquitin protein ligase | gga-miR-30e-5p_R+5 |
| TRIP12 | thyroid hormone receptor interactor 12 | aca-miR-363-3p_R+1 |
| TRIP12 | thyroid hormone receptor interactor 12 | oan-miR-363-3p_R+1 |
| ETFA | electron-transfer-flavoprotein, alpha polypeptide | PC-3p-52432_89 |
| NRG4 | neuregulin 4 | PC-5p-92816_49 |
| DNER | delta/notch-like EGF repeat containing | PC-5p-582426_7 |
| DNER | delta/notch-like EGF repeat containing | hsa-miR-483-3p_L-1R+2 |
| BECN1 | beclin 1, autophagy related | gga-miR-30a-5p_R+2_1ss13CT |
| BECN1 | beclin 1, autophagy related | gga-miR-30e-5p_R+5 |
| SPHKAP | SPHK1 interactor, AKAP domain containing | PC-5p-71982_65 |
| SPHKAP | SPHK1 interactor, AKAP domain containing | PC-5p-92816_49 |
| SPHKAP | SPHK1 interactor, AKAP domain containing | PC-5p-97606_47 |
| SPHKAP | SPHK1 interactor, AKAP domain containing | gga-miR-1a-3p_R+1_1ss2GT |
| SERPINF2 | serpin peptidase inhibitor, clade F (alpha-2 antiplasmin, pigment epithelium derived factor), member 2 | PC-3p-338480_12 |
| SERPINF2 | serpin peptidase inhibitor, clade F (alpha-2 antiplasmin, pigment epithelium derived factor), member 2 | PC-3p-52432_89 |
| ARID5B | AT rich interactive domain 5B (MRF1-like) | PC-3p-457530_9 |
| ARID5B | AT rich interactive domain 5B (MRF1-like) | PC-5p-582426_7 |
| ARID5B | AT rich interactive domain 5B (MRF1-like) | tgu-miR-2970-5p_1ss21GT |
| SUGP1 | SURP and G patch domain containing 1 | gga-miR-1456-3p_R+1 |
| SUGP1 | SURP and G patch domain containing 1 | gga-miR-1677-3p_L+1 |
| MASP2 | mannan-binding lectin serine peptidase 2 | PC-5p-582426_7 |
| ARMC6 | armadillo repeat containing 6 | PC-5p-113875_40 |
| SMYD4 | SET and MYND domain containing 4 | PC-5p-92816_49 |
| SRM | spermidine synthase | PC-3p-338480_12 |
| SUGP2 | SURP and G patch domain containing 2 | PC-5p-582426_7 |
| SUGP2 | SURP and G patch domain containing 2 | gga-miR-1782_L+2R-1 |
| AGFG1 | ArfGAP with FG repeats 1 | PC-5p-233284_18 |
| AGFG1 | ArfGAP with FG repeats 1 | gga-let-7g-3p_1ss22CT |
| AGFG1 | ArfGAP with FG repeats 1 | gga-miR-1677-3p_L+1 |
| TADA1L | transcriptional adaptor 1 (HFI1 homolog, yeast)-like | chi-miR-16b-5p_R+1 |
| TADA1L | transcriptional adaptor 1 (HFI1 homolog, yeast)-like | gga-miR-16-5p |
| FECH | ferrochelatase | hsa-miR-483-3p_L-1R+2 |
| MRPL44 | mitochondrial ribosomal protein L44 | PC-5p-582426_7 |
| OGFOD1 | 2-oxoglutarate and iron-dependent oxygenase domain containing 1 | tgu-miR-2970-5p_1ss21GT |
| MMD | monocyte to macrophage differentiation-associated | PC-3p-52432_89 |
| MMD | monocyte to macrophage differentiation-associated | aca-miR-363-3p_R+1 |
| MMD | monocyte to macrophage differentiation-associated | gga-let-7g-3p_1ss22CT |
| MMD | monocyte to macrophage differentiation-associated | gga-miR-1677-3p_L+1 |
| MMD | monocyte to macrophage differentiation-associated | gga-miR-30a-5p_R+2_1ss13CT |
| MMD | monocyte to macrophage differentiation-associated | gga-miR-30e-5p_R+5 |
| MMD | monocyte to macrophage differentiation-associated | oan-miR-363-3p_R+1 |
| EXOSC10 | exosome component 10 | chi-miR-16b-5p_R+1 |
| EXOSC10 | exosome component 10 | gga-miR-16-5p |
| PARN | poly(A)-specific ribonuclease | chi-miR-16b-5p_R+1 |
| PARN | poly(A)-specific ribonuclease | gga-miR-16-5p |
| PARN | poly(A)-specific ribonuclease | gga-miR-30a-5p_R+2_1ss13CT |
| PARN | poly(A)-specific ribonuclease | gga-miR-30e-5p_R+5 |
| ERLIN2 | ER lipid raft associated 2 | chi-miR-16b-5p_R+1 |
| ERLIN2 | ER lipid raft associated 2 | gga-miR-16-5p |
| PROSC | proline synthetase co-transcribed homolog (bacterial) | PC-3p-173245_25 |
| PROSC | proline synthetase co-transcribed homolog (bacterial) | tgu-miR-2970-5p_1ss21GT |
| PINK1 | PTEN induced putative kinase 1 | tgu-miR-2970-5p_1ss21GT |
| DDX49 | DEAD (Asp-Glu-Ala-Asp) box polypeptide 49 | PC-3p-457530_9 |
| DDX49 | DEAD (Asp-Glu-Ala-Asp) box polypeptide 49 | PC-5p-113875_40 |
| DDX49 | DEAD (Asp-Glu-Ala-Asp) box polypeptide 49 | PC-5p-233284_18 |
| DDX49 | DEAD (Asp-Glu-Ala-Asp) box polypeptide 49 | gga-miR-16-5p |
| DDX49 | DEAD (Asp-Glu-Ala-Asp) box polypeptide 49 | gga-miR-33-3p_L+1R+1 |
| ZC3H7A | zinc finger CCCH-type containing 7A | PC-5p-113875_40 |
| TRPC4AP | transient receptor potential cation channel, subfamily C, member 4 associated protein | PC-5p-582426_7 |
| TUBG1 | tubulin, gamma 1 | gga-miR-34b-5p_L-1R+1 |
| FAM134C | family with sequence similarity 134, member C | PC-5p-97606_47 |
| FAM134C | family with sequence similarity 134, member C | gga-miR-1677-3p_L+1 |
| REXO4 | REX4, RNA exonuclease 4 homolog (S. cerevisiae) | gga-miR-33-3p_L+1R+1 |
| SLC16A9 | solute carrier family 16, member 9 (monocarboxylic acid transporter 9) | PC-5p-97606_47 |
| MLX | MAX-like protein X | gga-miR-1456-5p_L-1 |
| MLX | MAX-like protein X | gga-miR-1782_L+2R-1 |
| MLX | MAX-like protein X | gga-miR-34b-5p_L-1R+1 |
| MLX | MAX-like protein X | hsa-miR-483-3p_L-1R+2 |
| EIF4EBP1 | eukaryotic translation initiation factor 4E binding protein 1 | PC-5p-97606_47 |
| TSR1 | TSR1, 20S rRNA accumulation, homolog (S. cerevisiae) | PC-5p-582426_7 |
| TSR1 | TSR1, 20S rRNA accumulation, homolog (S. cerevisiae) | gga-let-7g-3p_1ss22CT |
| TSR1 | TSR1, 20S rRNA accumulation, homolog (S. cerevisiae) | gga-miR-16-5p |
| TMEM231 | transmembrane protein 231 | gga-let-7g-3p_1ss22CT |
| KIF20A | kinesin family member 20A | PC-5p-113875_40 |
| KIF20A | kinesin family member 20A | PC-5p-97606_47 |
| KIF20A | kinesin family member 20A | gga-miR-1677-3p_L+1 |
| KIF20A | kinesin family member 20A | gga-miR-1782_L+2R-1 |
| KIF20A | kinesin family member 20A | tgu-miR-2970-5p_1ss21GT |
| STAR | steroidogenic acute regulatory protein | PC-5p-233284_18 |
| STAR | steroidogenic acute regulatory protein | gga-let-7g-3p_1ss22CT |
| RAD54L2 | RAD54-like 2 (S. cerevisiae) | aca-miR-425-3p_L-2R+1_1ss3AT |
| RAD54L2 | RAD54-like 2 (S. cerevisiae) | gga-miR-1456-3p_R+1 |
| NFYA | nuclear transcription factor Y, alpha | PC-5p-113875_40 |
| NFYA | nuclear transcription factor Y, alpha | PC-5p-582426_7 |
| NFYA | nuclear transcription factor Y, alpha | PC-5p-97606_47 |
| NFYA | nuclear transcription factor Y, alpha | gga-let-7g-3p_1ss22CT |
| NFYA | nuclear transcription factor Y, alpha | gga-miR-1805-5p_L-1 |
| NFYA | nuclear transcription factor Y, alpha | gga-miR-30a-5p_R+2_1ss13CT |
| NFYA | nuclear transcription factor Y, alpha | gga-miR-30e-5p_R+5 |
| SUB1 | SUB1 homolog (S. cerevisiae) | gga-miR-1782_L+2R-1 |
| SUB1 | SUB1 homolog (S. cerevisiae) | tgu-miR-2970-5p_1ss21GT |
| ITPA | inosine triphosphatase (nucleoside triphosphate pyrophosphatase) | chi-miR-16b-5p_R+1 |
| ITPA | inosine triphosphatase (nucleoside triphosphate pyrophosphatase) | gga-miR-16-5p |
| DNAJA4 | DnaJ (Hsp40) homolog, subfamily A, member 4 | PC-3p-457530_9 |
| DNAJA4 | DnaJ (Hsp40) homolog, subfamily A, member 4 | PC-5p-71982_65 |
| DNAJA4 | DnaJ (Hsp40) homolog, subfamily A, member 4 | gga-miR-1a-3p_R+1_1ss2GT |
| EIF2B1 | eukaryotic translation initiation factor 2B, subunit 1 alpha, 26kDa | aca-miR-363-3p_R+1 |
| EIF2B1 | eukaryotic translation initiation factor 2B, subunit 1 alpha, 26kDa | oan-miR-363-3p_R+1 |
| VAMP4 | vesicle-associated membrane protein 4 | chi-miR-16b-5p_R+1 |
| VAMP4 | vesicle-associated membrane protein 4 | gga-miR-16-5p |
| VAMP4 | vesicle-associated membrane protein 4 | tgu-miR-2970-5p_1ss21GT |
| STAT3 | signal transducer and activator of transcription 3 (acute-phase response factor) | PC-5p-582426_7 |
| HSDL1 | hydroxysteroid dehydrogenase like 1 | PC-5p-71982_65 |
| HSDL1 | hydroxysteroid dehydrogenase like 1 | tgu-miR-2970-5p_1ss21GT |
| COMP | cartilage oligomeric matrix protein | PC-5p-582426_7 |
| COMP | cartilage oligomeric matrix protein | chi-miR-16b-5p_R+1 |
| COMP | cartilage oligomeric matrix protein | gga-miR-16-5p |
| COMP | cartilage oligomeric matrix protein | gga-miR-1782_L+2R-1 |
| COMP | cartilage oligomeric matrix protein | gga-miR-1805-5p_L-1 |
| COMP | cartilage oligomeric matrix protein | gga-miR-7468-3p_L+3 |
| CRLF3 | cytokine receptor-like factor 3 | PC-5p-71982_65 |
| CRLF3 | cytokine receptor-like factor 3 | chi-miR-16b-5p_R+1 |
| CRLF3 | cytokine receptor-like factor 3 | gga-miR-16-5p |
| CRLF3 | cytokine receptor-like factor 3 | gga-miR-1a-3p_R+1_1ss2GT |
| CRLF3 | cytokine receptor-like factor 3 | gga-miR-34b-5p_L-1R+1 |
| ACSBG1 | acyl-CoA synthetase bubblegum family member 1 | gga-let-7g-3p_1ss22CT |
| HDAC1 | histone deacetylase 1 | gga-miR-34b-5p_L-1R+1 |
| DDX55 | DEAD (Asp-Glu-Ala-Asp) box polypeptide 55 | PC-5p-92816_49 |
| DDX55 | DEAD (Asp-Glu-Ala-Asp) box polypeptide 55 | gga-miR-34b-5p_L-1R+1 |
| TMED2 | transmembrane emp24 domain trafficking protein 2 | gga-let-7g-3p_1ss22CT |
| ERLIN1 | ER lipid raft associated 1 | PC-5p-71982_65 |
| ERLIN1 | ER lipid raft associated 1 | aca-miR-18a-5p_R-2 |
| ERLIN1 | ER lipid raft associated 1 | chi-miR-16b-5p_R+1 |
| ERLIN1 | ER lipid raft associated 1 | gga-miR-16-5p |
| ERLIN1 | ER lipid raft associated 1 | gga-miR-34b-5p_L-1R+1 |
| ADAP2 | ArfGAP with dual PH domains 2 | tgu-miR-2970-5p_1ss21GT |
| C17H9ORF174 | chromosome 17 open reading frame, human C9orf174 | PC-5p-97606_47 |
| GHDC | GH3 domain containing | PC-5p-113875_40 |
| GBGT1 | globoside alpha-1,3-N-acetylgalactosaminyltransferase 1 | PC-5p-37716_121 |
| GBGT1 | globoside alpha-1,3-N-acetylgalactosaminyltransferase 1 | gga-miR-1782_L+2R-1 |
| GBGT1 | globoside alpha-1,3-N-acetylgalactosaminyltransferase 1 | gga-miR-7468-3p_L+3 |
| SCYL3 | SCY1-like 3 (S. cerevisiae) | gga-let-7g-3p_1ss22CT |
| TM2D2 | TM2 domain containing 2 | PC-5p-582426_7 |
| TM2D2 | TM2 domain containing 2 | gga-let-7g-3p_1ss22CT |
| UBIAD1 | UbiA prenyltransferase domain containing 1 | PC-5p-582426_7 |
| UBIAD1 | UbiA prenyltransferase domain containing 1 | PC-5p-71982_65 |
| UBIAD1 | UbiA prenyltransferase domain containing 1 | PC-5p-92816_49 |
| UBIAD1 | UbiA prenyltransferase domain containing 1 | PC-5p-97606_47 |
| UBIAD1 | UbiA prenyltransferase domain containing 1 | gga-miR-1677-3p_L+1 |
| UBIAD1 | UbiA prenyltransferase domain containing 1 | gga-miR-1a-3p_R+1_1ss2GT |
| UBIAD1 | UbiA prenyltransferase domain containing 1 | gga-miR-34b-5p_L-1R+1 |
| UBIAD1 | UbiA prenyltransferase domain containing 1 | hsa-miR-483-3p_L-1R+2 |
| RAB5C | RAB5C, member RAS oncogene family | aca-miR-18a-5p_R-2 |
| RAB5C | RAB5C, member RAS oncogene family | gga-let-7g-3p_1ss22CT |
| ARL10 | ADP-ribosylation factor-like 10 | aca-miR-18a-5p_R-2 |
| ACSS2 | acyl-CoA synthetase short-chain family member 2 | hsa-miR-483-3p_L-1R+2 |
| KIAA1191 | KIAA1191 | PC-5p-71982_65 |
| KIAA1191 | KIAA1191 | PC-5p-92816_49 |
| KIAA1191 | KIAA1191 | aca-miR-18a-5p_R-2 |
| KIAA1191 | KIAA1191 | aca-miR-363-3p_R+1 |
| KIAA1191 | KIAA1191 | gga-miR-30a-5p_R+2_1ss13CT |
| KIAA1191 | KIAA1191 | gga-miR-30e-5p_R+5 |
| KIAA1191 | KIAA1191 | oan-miR-363-3p_R+1 |
| KIAA1191 | KIAA1191 | tgu-miR-2970-5p_1ss21GT |
| ELL | elongation factor RNA polymerase II | PC-5p-97606_47 |
| ELL | elongation factor RNA polymerase II | aca-miR-425-3p_L-2R+1_1ss3AT |
| ESRP2 | epithelial splicing regulatory protein 2 | gga-miR-30a-5p_R+2_1ss13CT |
| ESRP2 | epithelial splicing regulatory protein 2 | gga-miR-30e-5p_R+5 |
| KPNA6 | karyopherin alpha 6 (importin alpha 7) | PC-5p-233284_18 |
| KPNA6 | karyopherin alpha 6 (importin alpha 7) | chi-miR-16b-5p_R+1 |
| KPNA6 | karyopherin alpha 6 (importin alpha 7) | gga-miR-16-5p |
| DNAJC21 | DnaJ (Hsp40) homolog, subfamily C, member 21 | PC-5p-97606_47 |
| DNAJC21 | DnaJ (Hsp40) homolog, subfamily C, member 21 | gga-miR-1782_L+2R-1 |
| SBNO1 | strawberry notch homolog 1 (Drosophila) | PC-5p-97606_47 |
| RHOT1 | ras homolog family member T1 | chi-miR-16b-5p_R+1 |
| RHOT1 | ras homolog family member T1 | gga-miR-16-5p |
| BSDC1 | BSD domain containing 1 | PC-5p-37716_121 |
| BSDC1 | BSD domain containing 1 | aca-miR-363-3p_R+1 |
| BSDC1 | BSD domain containing 1 | chi-miR-16b-5p_R+1 |
| BSDC1 | BSD domain containing 1 | gga-miR-1456-3p_R+1 |
| BSDC1 | BSD domain containing 1 | gga-miR-16-5p |
| BSDC1 | BSD domain containing 1 | gga-miR-1a-3p_R+1_1ss2GT |
| BSDC1 | BSD domain containing 1 | gga-miR-34b-5p_L-1R+1 |
| BSDC1 | BSD domain containing 1 | oan-miR-363-3p_R+1 |
| DUS2L | dihydrouridine synthase 2-like, SMM1 homolog (S. cerevisiae) | gga-miR-33-3p_L+1R+1 |
| CDK2AP1 | cyclin-dependent kinase 2 associated protein 1 | gga-miR-1a-3p_R+1_1ss2GT |
| LMX1A | LIM homeobox transcription factor 1, alpha | gga-let-7g-3p_1ss22CT |
| C18H17ORF75 | chromosome 18 open reading frame, human C17orf75 | PC-5p-113875_40 |
| MGST3 | microsomal glutathione S-transferase 3 | gga-miR-33-3p_L+1R+1 |
| DNAJC7 | DnaJ (Hsp40) homolog, subfamily C, member 7 | chi-miR-16b-5p_R+1 |
| DNAJC7 | DnaJ (Hsp40) homolog, subfamily C, member 7 | gga-miR-16-5p |
| DNAJC7 | DnaJ (Hsp40) homolog, subfamily C, member 7 | gga-miR-30a-5p_R+2_1ss13CT |
| DNAJC7 | DnaJ (Hsp40) homolog, subfamily C, member 7 | gga-miR-30e-5p_R+5 |
| ZNF207 | zinc finger protein 207 | PC-5p-92816_49 |
| OCM | oncomodulin 2 | PC-3p-338480_12 |
| OCM | oncomodulin 2 | gga-miR-1677-3p_L+1 |
| SFRP1 | secreted frizzled-related protein 1 | chi-miR-16b-5p_R+1 |
| SFRP1 | secreted frizzled-related protein 1 | gga-miR-16-5p |
| SFRP1 | secreted frizzled-related protein 1 | tgu-miR-2970-5p_1ss21GT |
| MED20 | mediator complex subunit 20 | PC-5p-92816_49 |
| GFI1B | growth factor independent 1B transcription repressor | gga-miR-34b-5p_L-1R+1 |
| GOLGA7 | golgin A7 | PC-5p-582426_7 |
| GOLGA7 | golgin A7 | PC-5p-71982_65 |
| BARD1 | BRCA1 associated RING domain 1 | PC-5p-97606_47 |
| BARD1 | BRCA1 associated RING domain 1 | aca-miR-363-3p_R+1 |
| BARD1 | BRCA1 associated RING domain 1 | gga-miR-1782_L+2R-1 |
| BARD1 | BRCA1 associated RING domain 1 | oan-miR-363-3p_R+1 |
| CCND3 | cyclin D3 | PC-5p-37716_121 |
| CCND3 | cyclin D3 | chi-miR-16b-5p_R+1 |
| CCND3 | cyclin D3 | gga-miR-16-5p |
| ALDH9A1 | aldehyde dehydrogenase 9 family, member A1 | PC-5p-71982_65 |
| TAF8 | TAF8 RNA polymerase II, TATA box binding protein (TBP)-associated factor, 43kDa | PC-3p-457530_9 |
| TAF8 | TAF8 RNA polymerase II, TATA box binding protein (TBP)-associated factor, 43kDa | PC-5p-233284_18 |
| TAF8 | TAF8 RNA polymerase II, TATA box binding protein (TBP)-associated factor, 43kDa | PC-5p-582426_7 |
| TAF8 | TAF8 RNA polymerase II, TATA box binding protein (TBP)-associated factor, 43kDa | chi-miR-16b-5p_R+1 |
| TAF8 | TAF8 RNA polymerase II, TATA box binding protein (TBP)-associated factor, 43kDa | gga-miR-16-5p |
| TSC1 | tuberous sclerosis 1 | gga-miR-1677-3p_L+1 |
| TSC1 | tuberous sclerosis 1 | tgu-miR-2970-5p_1ss21GT |
| SPINK7 | serine peptidase inhibitor, Kazal type 7 (putative) | PC-5p-113875_40 |
| ZBTB8OS | zinc finger and BTB domain containing 8 opposite strand | gga-miR-1782_L+2R-1 |
| RBBP4 | retinoblastoma binding protein 4 | PC-5p-71982_65 |
| ZNF511 | zinc finger protein 511 | chi-miR-16b-5p_R+1 |
| ZNF511 | zinc finger protein 511 | gga-miR-16-5p |
| ZNF511 | zinc finger protein 511 | hsa-miR-483-3p_L-1R+2 |
| DDX31 | DEAD (Asp-Glu-Ala-Asp) box polypeptide 31 | PC-3p-173245_25 |
| SGK2 | serum/glucocorticoid regulated kinase 2 | PC-3p-52432_89 |
| SLC12A4 | solute carrier family 12 (potassium/chloride transporters), member 4 | gga-miR-34b-5p_L-1R+1 |
| SKP2 | S-phase kinase-associated protein 2, E3 ubiquitin protein ligase | gga-let-7g-3p_1ss22CT |
| SKP2 | S-phase kinase-associated protein 2, E3 ubiquitin protein ligase | hsa-miR-483-3p_L-1R+2 |
| BAIAP2L1 | BAI1-associated protein 2-like 1 | PC-5p-233284_18 |
| BAIAP2L1 | BAI1-associated protein 2-like 1 | gga-let-7g-3p_1ss22CT |
| SLC6A2 | solute carrier family 6 (neurotransmitter transporter, noradrenalin), member 2 | PC-5p-97606_47 |
| SLC6A2 | solute carrier family 6 (neurotransmitter transporter, noradrenalin), member 2 | gga-miR-1782_L+2R-1 |
| YARS | tyrosyl-tRNA synthetase | PC-3p-52432_89 |
| YARS | tyrosyl-tRNA synthetase | chi-miR-16b-5p_R+1 |
| YARS | tyrosyl-tRNA synthetase | gga-miR-16-5p |
| YARS | tyrosyl-tRNA synthetase | gga-miR-34b-5p_L-1R+1 |
| FNDC5 | fibronectin type III domain containing 5 | PC-5p-71982_65 |
| PPP1R16B | protein phosphatase 1, regulatory subunit 16B | gga-miR-1677-3p_L+1 |
| HPCA | hippocalcin | gga-miR-34b-5p_L-1R+1 |
| LPCAT2 | lysophosphatidylcholine acyltransferase 2 | PC-3p-173245_25 |
| LPCAT2 | lysophosphatidylcholine acyltransferase 2 | PC-5p-233284_18 |
| KPNA2 | karyopherin alpha 2 (RAG cohort 1, importin alpha 1) | gga-let-7g-3p_1ss22CT |
| TRRAP | transformation/transcription domain-associated protein | chi-miR-16b-5p_R+1 |
| TRRAP | transformation/transcription domain-associated protein | gga-miR-16-5p |
| TRRAP | transformation/transcription domain-associated protein | gga-miR-30a-5p_R+2_1ss13CT |
| TRRAP | transformation/transcription domain-associated protein | gga-miR-30e-5p_R+5 |
| AKTIP | AKT interacting protein | PC-3p-173245_25 |
| AKTIP | AKT interacting protein | PC-5p-233284_18 |
| AKTIP | AKT interacting protein | tgu-miR-2970-5p_1ss21GT |
| BPTF | bromodomain PHD finger transcription factor | tgu-miR-2970-5p_1ss21GT |
| LOC100859426 | nucleolar protein 11-like | PC-5p-582426_7 |
| NT5C3L | 5'-nucleotidase, cytosolic III-like | chi-miR-16b-5p_R+1 |
| NT5C3L | 5'-nucleotidase, cytosolic III-like | gga-miR-16-5p |
| ATAD1 | ATPase family, AAA domain containing 1 | PC-5p-113875_40 |
| GAS2 | growth arrest-specific 2 | tgu-miR-2970-5p_1ss21GT |
| AP3M2 | adaptor-related protein complex 3, mu 2 subunit | PC-3p-338480_12 |
| PIGS | phosphatidylinositol glycan anchor biosynthesis, class S | PC-3p-338480_12 |
| PIGS | phosphatidylinositol glycan anchor biosynthesis, class S | PC-3p-52432_89 |
| PIGS | phosphatidylinositol glycan anchor biosynthesis, class S | PC-5p-37716_121 |
| UBLCP1 | ubiquitin-like domain containing CTD phosphatase 1 | gga-miR-1a-3p_R+1_1ss2GT |
| TOP1 | topoisomerase (DNA) I | PC-5p-71982_65 |
| TOP1 | topoisomerase (DNA) I | gga-miR-1782_L+2R-1 |
| PAPSS2 | 3'-phosphoadenosine 5'-phosphosulfate synthase 2 | PC-5p-71982_65 |
| LOC101749631 | gypsy retrotransposon integrase-like protein 1-like | gga-miR-1805-5p_L-1 |
| MINPP1 | multiple inositol-polyphosphate phosphatase 1 | PC-5p-113875_40 |
| MINPP1 | multiple inositol-polyphosphate phosphatase 1 | chi-miR-16b-5p_R+1 |
| MINPP1 | multiple inositol-polyphosphate phosphatase 1 | gga-miR-16-5p |
| CLINT1 | clathrin interactor 1 | PC-5p-97606_47 |
| CLINT1 | clathrin interactor 1 | gga-let-7g-3p_1ss22CT |
| CLINT1 | clathrin interactor 1 | gga-miR-1677-3p_L+1 |
| USE1 | unconventional SNARE in the ER 1 homolog (S. cerevisiae) | PC-5p-71982_65 |
| USE1 | unconventional SNARE in the ER 1 homolog (S. cerevisiae) | gga-miR-7468-3p_L+3 |
| RAPGEF1 | Rap guanine nucleotide exchange factor (GEF) 1 | gga-miR-1456-3p_R+1 |
| RAPGEF1 | Rap guanine nucleotide exchange factor (GEF) 1 | gga-miR-30a-5p_R+2_1ss13CT |
| RAPGEF1 | Rap guanine nucleotide exchange factor (GEF) 1 | gga-miR-30e-5p_R+5 |
| THG1L | tRNA-histidine guanylyltransferase 1-like (S. cerevisiae) | gga-miR-1782_L+2R-1 |
| EGFLAM | EGF-like, fibronectin type III and laminin G domains | gga-miR-1a-3p_R+1_1ss2GT |
| LIFR | leukemia inhibitory factor receptor alpha | PC-5p-582426_7 |
| LIFR | leukemia inhibitory factor receptor alpha | gga-miR-1782_L+2R-1 |
| C21H1ORF144 | chromosome 21 open reading frame, human C1orf144 | PC-3p-282885_15 |
| C21H1ORF144 | chromosome 21 open reading frame, human C1orf144 | chi-miR-16b-5p_R+1 |
| C21H1ORF144 | chromosome 21 open reading frame, human C1orf144 | gga-miR-16-5p |
| C21H1ORF144 | chromosome 21 open reading frame, human C1orf144 | gga-miR-34b-5p_L-1R+1 |
| SALL1 | sal-like 1 (Drosophila) | PC-3p-457530_9 |
| SALL1 | sal-like 1 (Drosophila) | chi-miR-16b-5p_R+1 |
| SALL1 | sal-like 1 (Drosophila) | gga-let-7g-3p_1ss22CT |
| SALL1 | sal-like 1 (Drosophila) | gga-miR-16-5p |
| SALL1 | sal-like 1 (Drosophila) | gga-miR-1805-5p_L-1 |
| OSMR | oncostatin M receptor | hsa-miR-483-3p_L-1R+2 |
| ANO5 | anoctamin 5 | gga-miR-1782_L+2R-1 |
| NELL1 | NEL-like 1 | PC-5p-113875_40 |
| TMEM38A | transmembrane protein 38A | PC-3p-338480_12 |
| TMEM38A | transmembrane protein 38A | PC-5p-582426_7 |
| TMEM38A | transmembrane protein 38A | chi-miR-16b-5p_R+1 |
| TMEM38A | transmembrane protein 38A | gga-miR-16-5p |
| TMEM38A | transmembrane protein 38A | gga-miR-34b-5p_L-1R+1 |
| SUN1 | Sad1 and UNC84 domain containing 1 | PC-5p-233284_18 |
| GTF2A2 | general transcription factor IIA, 2, 12kDa | PC-5p-233284_18 |
| PABPC4 | poly(A) binding protein, cytoplasmic 4 (inducible form) | PC-5p-113875_40 |
| PABPC4 | poly(A) binding protein, cytoplasmic 4 (inducible form) | PC-5p-71982_65 |
| GPN3 | GPN-loop GTPase 3 | gga-miR-1782_L+2R-1 |
| DKK1 | dickkopf homolog 1 (Xenopus laevis) | PC-5p-71982_65 |
| ARPC3 | actin related protein 2/3 complex, subunit 3, 21kDa | gga-miR-1805-5p_L-1 |
| KRT20 | keratin 20 | PC-5p-92816_49 |
| KRT14 | keratin 14 | gga-let-7g-3p_1ss22CT |
| KRT15 | keratin 15 | gga-let-7g-3p_1ss22CT |
| TRIT1 | tRNA isopentenyltransferase 1 | gga-miR-34b-5p_L-1R+1 |
| IFT81 | intraflagellar transport 81 homolog (Chlamydomonas) | PC-5p-92816_49 |
| IFT81 | intraflagellar transport 81 homolog (Chlamydomonas) | gga-miR-7468-3p_L+3 |
| LRRFIP1 | leucine rich repeat (in FLII) interacting protein 1 | PC-5p-97606_47 |
| FIBCD1 | fibrinogen C domain containing 1 | gga-miR-1456-3p_R+1 |
| MINA | MYC induced nuclear antigen | PC-3p-52432_89 |
| MINA | MYC induced nuclear antigen | PC-5p-71982_65 |
| MFSD2A | major facilitator superfamily domain containing 2A | gga-let-7g-3p_1ss22CT |
| STX6 | syntaxin 6 | PC-3p-173245_25 |
| SMARCE1 | SWI/SNF related, matrix associated, actin dependent regulator of chromatin, subfamily e, member 1 | PC-5p-582426_7 |
| SMARCE1 | SWI/SNF related, matrix associated, actin dependent regulator of chromatin, subfamily e, member 1 | chi-miR-16b-5p_R+1 |
| SMARCE1 | SWI/SNF related, matrix associated, actin dependent regulator of chromatin, subfamily e, member 1 | gga-miR-16-5p |
| SGCD | sarcoglycan, delta (35kDa dystrophin-associated glycoprotein) | PC-5p-233284_18 |
| XPR1 | xenotropic and polytropic retrovirus receptor 1 | gga-miR-30a-5p_R+2_1ss13CT |
| XPR1 | xenotropic and polytropic retrovirus receptor 1 | gga-miR-30e-5p_R+5 |
| RAB17 | RAB17, member RAS oncogene family | PC-3p-52432_89 |
| RAB17 | RAB17, member RAS oncogene family | PC-5p-37716_121 |
| RAB17 | RAB17, member RAS oncogene family | PC-5p-97606_47 |
| P2RX4 | purinergic receptor P2X, ligand-gated ion channel, 4 | PC-3p-52432_89 |
| P2RX4 | purinergic receptor P2X, ligand-gated ion channel, 4 | chi-miR-16b-5p_R+1 |
| P2RX4 | purinergic receptor P2X, ligand-gated ion channel, 4 | gga-miR-16-5p |
| P2RX4 | purinergic receptor P2X, ligand-gated ion channel, 4 | gga-miR-1782_L+2R-1 |
| N4BP1 | NEDD4 binding protein 1 | chi-miR-16b-5p_R+1 |
| N4BP1 | NEDD4 binding protein 1 | gga-miR-16-5p |
| N4BP1 | NEDD4 binding protein 1 | gga-miR-1782_L+2R-1 |
| PIGT | phosphatidylinositol glycan anchor biosynthesis, class T | PC-5p-71982_65 |
| UBR4 | ubiquitin protein ligase E3 component n-recognin 4 | aca-miR-425-3p_L-2R+1_1ss3AT |
| DBNDD2 | dysbindin (dystrobrevin binding protein 1) domain containing 2 | PC-5p-97606_47 |
| DBNDD2 | dysbindin (dystrobrevin binding protein 1) domain containing 2 | chi-miR-16b-5p_R+1 |
| DBNDD2 | dysbindin (dystrobrevin binding protein 1) domain containing 2 | gga-miR-16-5p |
| COL6A3 | collagen, type VI, alpha 3 | PC-3p-338480_12 |
| MYPN | myopalladin | aca-miR-363-3p_R+1 |
| MYPN | myopalladin | chi-miR-16b-5p_R+1 |
| MYPN | myopalladin | gga-miR-16-5p |
| MYPN | myopalladin | oan-miR-363-3p_R+1 |
| SDC4 | syndecan 4 | PC-3p-52432_89 |
| GEMIN5 | gem (nuclear organelle) associated protein 5 | PC-3p-457530_9 |
| GEMIN5 | gem (nuclear organelle) associated protein 5 | gga-miR-1a-3p_R+1_1ss2GT |
| GEMIN5 | gem (nuclear organelle) associated protein 5 | hsa-miR-483-3p_L-1R+2 |
| COPS8 | COP9 constitutive photomorphogenic homolog subunit 8 (Arabidopsis) | gga-miR-1782_L+2R-1 |
| LOC101749801 | protein kinase C alpha type-like | PC-5p-71982_65 |
| LOC101749801 | protein kinase C alpha type-like | gga-miR-1677-3p_L+1 |
| LOC101749801 | protein kinase C alpha type-like | hsa-miR-483-3p_L-1R+2 |
| FAM189A1 | family with sequence similarity 189, member A1 | PC-5p-233284_18 |
| FAM189A1 | family with sequence similarity 189, member A1 | gga-miR-1a-3p_R+1_1ss2GT |
| FAM189A1 | family with sequence similarity 189, member A1 | gga-miR-33-3p_L+1R+1 |
| CNOT8 | CCR4-NOT transcription complex, subunit 8 | gga-let-7g-3p_1ss22CT |
| CNOT8 | CCR4-NOT transcription complex, subunit 8 | gga-miR-7468-3p_L+3 |
| AP1M1 | adaptor-related protein complex 1, mu 1 subunit | PC-3p-338480_12 |
| AP1M1 | adaptor-related protein complex 1, mu 1 subunit | aca-miR-363-3p_R+1 |
| AP1M1 | adaptor-related protein complex 1, mu 1 subunit | chi-miR-16b-5p_R+1 |
| AP1M1 | adaptor-related protein complex 1, mu 1 subunit | gga-miR-16-5p |
| AP1M1 | adaptor-related protein complex 1, mu 1 subunit | gga-miR-1782_L+2R-1 |
| AP1M1 | adaptor-related protein complex 1, mu 1 subunit | gga-miR-30a-5p_R+2_1ss13CT |
| AP1M1 | adaptor-related protein complex 1, mu 1 subunit | gga-miR-30e-5p_R+5 |
| AP1M1 | adaptor-related protein complex 1, mu 1 subunit | oan-miR-363-3p_R+1 |
| AP1M1 | adaptor-related protein complex 1, mu 1 subunit | tgu-miR-2970-5p_1ss21GT |
| RPL23A | ribosomal protein L23a | gga-let-7g-3p_1ss22CT |
| TARSL2 | threonyl-tRNA synthetase-like 2 | PC-5p-71982_65 |
| ASB18 | ankyrin repeat and SOCS box containing 18 | chi-miR-16b-5p_R+1 |
| ASB18 | ankyrin repeat and SOCS box containing 18 | gga-miR-16-5p |
| ASB18 | ankyrin repeat and SOCS box containing 18 | hsa-miR-483-3p_L-1R+2 |
| PPM1M | protein phosphatase, Mg2+/Mn2+ dependent, 1M | PC-5p-233284_18 |
| PPM1M | protein phosphatase, Mg2+/Mn2+ dependent, 1M | chi-miR-16b-5p_R+1 |
| PPM1M | protein phosphatase, Mg2+/Mn2+ dependent, 1M | gga-miR-16-5p |
| PHKB | phosphorylase kinase, beta | gga-miR-1456-5p_L-1 |
| AKR7A2 | aldo-keto reductase family 7, member A2 (aflatoxin aldehyde reductase) | PC-5p-71982_65 |
| CAPZB | capping protein (actin filament) muscle Z-line, beta | gga-let-7g-3p_1ss22CT |
| DNA2 | DNA replication helicase 2 homolog (yeast) | chi-miR-16b-5p_R+1 |
| DNA2 | DNA replication helicase 2 homolog (yeast) | gga-miR-16-5p |
| DNA2 | DNA replication helicase 2 homolog (yeast) | gga-miR-30a-5p_R+2_1ss13CT |
| DNA2 | DNA replication helicase 2 homolog (yeast) | gga-miR-30e-5p_R+5 |
| MFAP3 | microfibrillar-associated protein 3 | PC-3p-457530_9 |
| MFAP3 | microfibrillar-associated protein 3 | PC-5p-113875_40 |
| MFAP3 | microfibrillar-associated protein 3 | PC-5p-71982_65 |
| MFAP3 | microfibrillar-associated protein 3 | hsa-miR-483-3p_L-1R+2 |
| CSRP3 | cysteine and glycine-rich protein 3 (cardiac LIM protein) | PC-5p-97606_47 |
| CSRP3 | cysteine and glycine-rich protein 3 (cardiac LIM protein) | gga-miR-30a-5p_R+2_1ss13CT |
| CSRP3 | cysteine and glycine-rich protein 3 (cardiac LIM protein) | gga-miR-30e-5p_R+5 |
| CSRP3 | cysteine and glycine-rich protein 3 (cardiac LIM protein) | gga-miR-33-3p_L+1R+1 |
| GALE | UDP-galactose-4-epimerase | PC-3p-52432_89 |
| GPR107 | G protein-coupled receptor 107 | PC-3p-173245_25 |
| FAM114A2 | family with sequence similarity 114, member A2 | gga-miR-1677-3p_L+1 |
| MICALL2 | MICAL-like 2 | chi-miR-16b-5p_R+1 |
| MICALL2 | MICAL-like 2 | gga-miR-16-5p |
| CHMP1B | charged multivesicular body protein 1B | PC-5p-113875_40 |
| CHMP1B | charged multivesicular body protein 1B | gga-let-7g-3p_1ss22CT |
| KDM4B | lysine (K)-specific demethylase 4B | PC-5p-92816_49 |
| KDM4B | lysine (K)-specific demethylase 4B | aca-miR-363-3p_R+1 |
| KDM4B | lysine (K)-specific demethylase 4B | oan-miR-363-3p_R+1 |
| CHRNA7 | cholinergic receptor, nicotinic, alpha 7 (neuronal) | PC-5p-97606_47 |
| STK4 | serine/threonine kinase 4 | PC-3p-338480_12 |
| STK4 | serine/threonine kinase 4 | PC-5p-113875_40 |
| STK4 | serine/threonine kinase 4 | aca-miR-18a-5p_R-2 |
| STK4 | serine/threonine kinase 4 | hsa-miR-483-3p_L-1R+2 |
| STK4 | serine/threonine kinase 4 | tgu-miR-2970-5p_1ss21GT |
| FAM81A | family with sequence similarity 81, member A | chi-miR-16b-5p_R+1 |
| FAM81A | family with sequence similarity 81, member A | gga-miR-16-5p |
| FAM81A | family with sequence similarity 81, member A | gga-miR-30a-5p_R+2_1ss13CT |
| FAM81A | family with sequence similarity 81, member A | gga-miR-30e-5p_R+5 |
| INTS1 | integrator complex subunit 1 | PC-5p-71982_65 |
| ITM2A | integral membrane protein 2A | aca-miR-363-3p_R+1 |
| ITM2A | integral membrane protein 2A | oan-miR-363-3p_R+1 |
| ITM2A | integral membrane protein 2A | tgu-miR-2970-5p_1ss21GT |
| TOMM34 | translocase of outer mitochondrial membrane 34 | PC-5p-97606_47 |
| TOMM34 | translocase of outer mitochondrial membrane 34 | chi-miR-16b-5p_R+1 |
| TOMM34 | translocase of outer mitochondrial membrane 34 | gga-miR-16-5p |
| GPR174 | G protein-coupled receptor 174 | aca-miR-18a-5p_R-2 |
| DNAJA2 | DnaJ (Hsp40) homolog, subfamily A, member 2 | tgu-miR-2970-5p_1ss21GT |
| PNRC2 | proline-rich nuclear receptor coactivator 2 | PC-5p-233284_18 |
| PNRC2 | proline-rich nuclear receptor coactivator 2 | gga-miR-1782_L+2R-1 |
| PNRC2 | proline-rich nuclear receptor coactivator 2 | gga-miR-1a-3p_R+1_1ss2GT |
| PNRC2 | proline-rich nuclear receptor coactivator 2 | gga-miR-30a-5p_R+2_1ss13CT |
| PNRC2 | proline-rich nuclear receptor coactivator 2 | gga-miR-30e-5p_R+5 |
| PNRC2 | proline-rich nuclear receptor coactivator 2 | gga-miR-7468-3p_L+3 |
| PABPC1L | poly(A) binding protein, cytoplasmic 1-like | PC-5p-71982_65 |
| UHRF1 | ubiquitin-like with PHD and ring finger domains 1 | PC-5p-37716_121 |
| UHRF1 | ubiquitin-like with PHD and ring finger domains 1 | PC-5p-582426_7 |
| SRSF10 | serine/arginine-rich splicing factor 10 | PC-3p-173245_25 |
| SRSF10 | serine/arginine-rich splicing factor 10 | tgu-miR-2970-5p_1ss21GT |
| TDRD5 | tudor domain containing 5 | chi-miR-16b-5p_R+1 |
| TDRD5 | tudor domain containing 5 | gga-miR-16-5p |
| USP20 | ubiquitin specific peptidase 20 | hsa-miR-483-3p_L-1R+2 |
| YWHAB | tyrosine 3-monooxygenase/tryptophan 5-monooxygenase activation protein, beta polypeptide | PC-3p-173245_25 |
| YWHAB | tyrosine 3-monooxygenase/tryptophan 5-monooxygenase activation protein, beta polypeptide | PC-5p-113875_40 |
| YWHAB | tyrosine 3-monooxygenase/tryptophan 5-monooxygenase activation protein, beta polypeptide | PC-5p-582426_7 |
| DDX50 | DEAD (Asp-Glu-Ala-Asp) box polypeptide 50 | PC-5p-92816_49 |
| DDX50 | DEAD (Asp-Glu-Ala-Asp) box polypeptide 50 | gga-let-7g-3p_1ss22CT |
| C17H9ORF78 | chromosome 17 open reading frame, human C9orf78 | PC-3p-338480_12 |
| NADSYN1 | NAD synthetase 1 | PC-5p-92816_49 |
| KIAA1279 | KIAA1279 | PC-5p-97606_47 |
| KIAA1279 | KIAA1279 | gga-let-7g-3p_1ss22CT |
| GPT2 | glutamic pyruvate transaminase (alanine aminotransferase) 2 | gga-miR-1805-5p_L-1 |
| CCNB2 | cyclin B2 | gga-miR-1805-5p_L-1 |
| TOR1A | torsin family 1, member A (torsin A) | PC-5p-71982_65 |
| ADA | adenosine deaminase | PC-5p-71982_65 |
| RNF111 | ring finger protein 111 | chi-miR-16b-5p_R+1 |
| RNF111 | ring finger protein 111 | gga-miR-16-5p |
| RNF111 | ring finger protein 111 | gga-miR-30a-5p_R+2_1ss13CT |
| RNF111 | ring finger protein 111 | gga-miR-30e-5p_R+5 |
| BET1L | blocked early in transport 1 homolog (S. cerevisiae)-like | aca-miR-18a-5p_R-2 |
| VPS26A | vacuolar protein sorting 26 homolog A (S. pombe) | gga-miR-1a-3p_R+1_1ss2GT |
| ATOX1 | ATX1 antioxidant protein 1 homolog (yeast) | chi-miR-16b-5p_R+1 |
| ATOX1 | ATX1 antioxidant protein 1 homolog (yeast) | gga-miR-16-5p |
| TAF9 | TAF9 RNA polymerase II, TATA box binding protein (TBP)-associated factor, 32kDa | hsa-miR-483-3p_L-1R+2 |
| RNF34 | ring finger protein 34, E3 ubiquitin protein ligase | PC-5p-97606_47 |
| RNF34 | ring finger protein 34, E3 ubiquitin protein ligase | chi-miR-16b-5p_R+1 |
| RNF34 | ring finger protein 34, E3 ubiquitin protein ligase | gga-miR-16-5p |
| RNF34 | ring finger protein 34, E3 ubiquitin protein ligase | gga-miR-30a-5p_R+2_1ss13CT |
| RNF34 | ring finger protein 34, E3 ubiquitin protein ligase | gga-miR-30e-5p_R+5 |
| RNF34 | ring finger protein 34, E3 ubiquitin protein ligase | gga-miR-34b-5p_L-1R+1 |
| RIC8A | resistance to inhibitors of cholinesterase 8 homolog A (C. elegans) | PC-5p-97606_47 |
| RIC8A | resistance to inhibitors of cholinesterase 8 homolog A (C. elegans) | aca-miR-363-3p_R+1 |
| RIC8A | resistance to inhibitors of cholinesterase 8 homolog A (C. elegans) | oan-miR-363-3p_R+1 |
| KIF4A | kinesin family member 4A | gga-miR-30a-5p_R+2_1ss13CT |
| KIF4A | kinesin family member 4A | gga-miR-30e-5p_R+5 |
| SIRT3 | sirtuin (silent mating type information regulation 2 homolog) 3 (S. cerevisiae) | PC-5p-71982_65 |
| SIRT3 | sirtuin (silent mating type information regulation 2 homolog) 3 (S. cerevisiae) | chi-miR-16b-5p_R+1 |
| SIRT3 | sirtuin (silent mating type information regulation 2 homolog) 3 (S. cerevisiae) | gga-miR-16-5p |
| SIRT3 | sirtuin (silent mating type information regulation 2 homolog) 3 (S. cerevisiae) | gga-miR-34b-5p_L-1R+1 |
| TRMU | tRNA 5-methylaminomethyl-2-thiouridylate methyltransferase | PC-5p-113875_40 |
| ADAM10 | ADAM metallopeptidase domain 10 | aca-miR-363-3p_R+1 |
| ADAM10 | ADAM metallopeptidase domain 10 | gga-miR-1782_L+2R-1 |
| ADAM10 | ADAM metallopeptidase domain 10 | oan-miR-363-3p_R+1 |
| HK1 | hexokinase 1 | chi-miR-16b-5p_R+1 |
| HK1 | hexokinase 1 | gga-miR-16-5p |
| FAM20B | family with sequence similarity 20, member B | PC-3p-338480_12 |
| FAM20B | family with sequence similarity 20, member B | aca-miR-363-3p_R+1 |
| FAM20B | family with sequence similarity 20, member B | gga-miR-33-3p_L+1R+1 |
| FAM20B | family with sequence similarity 20, member B | oan-miR-363-3p_R+1 |
| ATHL1 | ATH1, acid trehalase-like 1 (yeast) | PC-5p-582426_7 |
| LOC771090 | STAM-binding protein-like | PC-5p-97606_47 |
| TTPAL | tocopherol (alpha) transfer protein-like | PC-3p-338480_12 |
| TTPAL | tocopherol (alpha) transfer protein-like | PC-5p-97606_47 |
| TTPAL | tocopherol (alpha) transfer protein-like | gga-let-7g-3p_1ss22CT |
| GRHL3 | grainyhead-like 3 (Drosophila) | PC-3p-52432_89 |
| GRHL3 | grainyhead-like 3 (Drosophila) | PC-5p-97606_47 |
| LOC420160 | cathepsin L1-like | PC-5p-97606_47 |
| DHRS3 | dehydrogenase/reductase (SDR family) member 3 | PC-5p-97606_47 |
| TSPAN15 | tetraspanin 15 | gga-miR-34b-5p_L-1R+1 |
| BLMH | bleomycin hydrolase | gga-miR-7468-3p_L+3 |
| TMIGD1 | transmembrane and immunoglobulin domain containing 1 | aca-miR-18a-5p_R-2 |
| TMIGD1 | transmembrane and immunoglobulin domain containing 1 | chi-miR-16b-5p_R+1 |
| TMIGD1 | transmembrane and immunoglobulin domain containing 1 | gga-miR-16-5p |
| TNFAIP8L1 | tumor necrosis factor, alpha-induced protein 8-like 1 | PC-5p-582426_7 |
| SIGIRR | single immunoglobulin and toll-interleukin 1 receptor (TIR) domain | chi-miR-16b-5p_R+1 |
| SIGIRR | single immunoglobulin and toll-interleukin 1 receptor (TIR) domain | gga-miR-16-5p |
| ASB1 | ankyrin repeat and SOCS box containing 1 | PC-3p-52432_89 |
| RALGPS2 | Ral GEF with PH domain and SH3 binding motif 2 | PC-5p-582426_7 |
| RALGPS2 | Ral GEF with PH domain and SH3 binding motif 2 | gga-miR-1329-3p |
| RALGPS2 | Ral GEF with PH domain and SH3 binding motif 2 | gga-miR-34b-5p_L-1R+1 |
| RALGPS2 | Ral GEF with PH domain and SH3 binding motif 2 | tgu-miR-2970-5p_1ss21GT |
| ANO9 | anoctamin 9 | gga-miR-7468-3p_L+3 |
| RCAN3 | RCAN family member 3 | PC-3p-338480_12 |
| RCAN3 | RCAN family member 3 | aca-miR-18a-5p_R-2 |
| RCAN3 | RCAN family member 3 | chi-miR-16b-5p_R+1 |
| RCAN3 | RCAN family member 3 | gga-miR-16-5p |
| RCAN3 | RCAN family member 3 | tgu-miR-2970-5p_1ss21GT |
| HNF4A | hepatocyte nuclear factor 4, alpha | PC-5p-37716_121 |
| COL13A1 | collagen, type XIII, alpha 1 | aca-miR-425-3p_L-2R+1_1ss3AT |
| PLG | plasminogen | aca-miR-363-3p_R+1 |
| PLG | plasminogen | oan-miR-363-3p_R+1 |
| CPD | carboxypeptidase D | PC-5p-97606_47 |
| GOSR1 | golgi SNAP receptor complex member 1 | PC-5p-113875_40 |
| GOSR1 | golgi SNAP receptor complex member 1 | gga-miR-1329-3p |
| GOSR1 | golgi SNAP receptor complex member 1 | hsa-miR-483-3p_L-1R+2 |
| ORC6 | origin recognition complex, subunit 6 | PC-5p-71982_65 |
| OIT3 | oncoprotein induced transcript 3 | PC-5p-582426_7 |
| OIT3 | oncoprotein induced transcript 3 | gga-let-7g-3p_1ss22CT |
| PSMD9 | proteasome (prosome, macropain) 26S subunit, non-ATPase, 9 | gga-miR-1677-3p_L+1 |
| CCDC69 | coiled-coil domain containing 69 | PC-3p-282885_15 |
| MAP2K6 | mitogen-activated protein kinase kinase 6 | PC-5p-97606_47 |
| VPS35 | vacuolar protein sorting 35 homolog (S. cerevisiae) | PC-3p-173245_25 |
| VPS35 | vacuolar protein sorting 35 homolog (S. cerevisiae) | PC-5p-92816_49 |
| VPS35 | vacuolar protein sorting 35 homolog (S. cerevisiae) | gga-miR-1a-3p_R+1_1ss2GT |
| VPS35 | vacuolar protein sorting 35 homolog (S. cerevisiae) | gga-miR-7468-3p_L+3 |
| KCNJ2 | potassium inwardly-rectifying channel, subfamily J, member 2 | chi-miR-16b-5p_R+1 |
| KCNJ2 | potassium inwardly-rectifying channel, subfamily J, member 2 | gga-miR-16-5p |
| KCNJ2 | potassium inwardly-rectifying channel, subfamily J, member 2 | gga-miR-1782_L+2R-1 |
| ASB6 | ankyrin repeat and SOCS box containing 6 | PC-5p-71982_65 |
| ASB6 | ankyrin repeat and SOCS box containing 6 | gga-miR-34b-5p_L-1R+1 |
| ASB6 | ankyrin repeat and SOCS box containing 6 | tgu-miR-2970-5p_1ss21GT |
| SLC19A1 | solute carrier family 19 (folate transporter), member 1 | PC-3p-457530_9 |
| SLC19A1 | solute carrier family 19 (folate transporter), member 1 | PC-5p-97606_47 |
| DYX1C1 | dyslexia susceptibility 1 candidate 1 | gga-let-7g-3p_1ss22CT |
| CCPG1 | cell cycle progression 1 | gga-let-7g-3p_1ss22CT |
| ANAPC16 | anaphase promoting complex subunit 16 | PC-3p-338480_12 |
| ANAPC16 | anaphase promoting complex subunit 16 | PC-5p-37716_121 |
| ANAPC16 | anaphase promoting complex subunit 16 | PC-5p-71982_65 |
| SLC39A11 | solute carrier family 39 (metal ion transporter), member 11 | gga-miR-7468-3p_L+3 |
| ASCC1 | activating signal cointegrator 1 complex subunit 1 | PC-5p-97606_47 |
| TNFRSF1B | tumor necrosis factor receptor superfamily, member 1B | PC-5p-92816_49 |
| SAR1A | SAR1 homolog A (S. cerevisiae) | PC-3p-282885_15 |
| CYP3A4 | cytochrome P450, family 3, subfamily A, polypeptide 4 | PC-5p-97606_47 |
| PPA1 | pyrophosphatase (inorganic) 1 | chi-miR-16b-5p_R+1 |
| PPA1 | pyrophosphatase (inorganic) 1 | gga-miR-16-5p |
| SLC13A3 | solute carrier family 13 (sodium-dependent dicarboxylate transporter), member 3 | PC-5p-92816_49 |
| SLC13A3 | solute carrier family 13 (sodium-dependent dicarboxylate transporter), member 3 | gga-miR-1329-3p |
| CYP3A7 | cytochrome P450 A 37 | PC-5p-582426_7 |
| CLIP1 | CAP-GLY domain containing linker protein 1 | PC-3p-173245_25 |
| FAM82A2 | family with sequence similarity 82, member A2 | PC-5p-113875_40 |
| NCOR1 | nuclear receptor corepressor 1 | PC-5p-97606_47 |
| C11H19ORF12 | chromosome 11 open reading frame, human C19orf12 | gga-miR-34b-5p_L-1R+1 |
| DOLPP1 | dolichyl pyrophosphate phosphatase 1 | PC-5p-71982_65 |
| DMGDH | dimethylglycine dehydrogenase | PC-5p-71982_65 |
| CCNE1 | cyclin E1 | PC-5p-233284_18 |
| CCNE1 | cyclin E1 | chi-miR-16b-5p_R+1 |
| CCNE1 | cyclin E1 | gga-miR-16-5p |
| CCNE1 | cyclin E1 | gga-miR-1a-3p_R+1_1ss2GT |
| DNAI2 | dynein, axonemal, intermediate chain 2 | PC-5p-233284_18 |
| SLC2A10 | solute carrier family 2 (facilitated glucose transporter), member 10 | PC-3p-52432_89 |
| URI1 | chromosome 11 open reading frame, human C19orf2 | aca-miR-18a-5p_R-2 |
| EYA2 | eyes absent homolog 2 (Drosophila) | gga-miR-33-3p_L+1R+1 |
| SH3GLB1 | SH3-domain GRB2-like endophilin B1 | gga-miR-1782_L+2R-1 |
| SH3GLB2 | SH3-domain GRB2-like endophilin B2 | PC-5p-97606_47 |
| SH3GLB2 | SH3-domain GRB2-like endophilin B2 | gga-miR-34b-5p_L-1R+1 |
| PLOD1 | procollagen-lysine, 2-oxoglutarate 5-dioxygenase 1 | PC-5p-582426_7 |
| PLOD1 | procollagen-lysine, 2-oxoglutarate 5-dioxygenase 1 | gga-miR-34b-5p_L-1R+1 |
| DCTN4 | dynactin 4 (p62) | PC-5p-97606_47 |
| DCTN4 | dynactin 4 (p62) | gga-miR-34b-5p_L-1R+1 |
| PPTC7 | PTC7 protein phosphatase homolog (S. cerevisiae) | aca-miR-425-3p_L-2R+1_1ss3AT |
| SGPL1 | sphingosine-1-phosphate lyase 1 | gga-miR-1782_L+2R-1 |
| CACYBP | calcyclin binding protein | gga-miR-30a-5p_R+2_1ss13CT |
| CACYBP | calcyclin binding protein | gga-miR-30e-5p_R+5 |
| WIPI2 | WD repeat domain, phosphoinositide interacting 2 | PC-5p-97606_47 |
| WIPI2 | WD repeat domain, phosphoinositide interacting 2 | chi-miR-16b-5p_R+1 |
| WIPI2 | WD repeat domain, phosphoinositide interacting 2 | gga-miR-16-5p |
| WIPI2 | WD repeat domain, phosphoinositide interacting 2 | gga-miR-1782_L+2R-1 |
| HVCN1 | hydrogen voltage-gated channel 1 | PC-3p-52432_89 |
| HVCN1 | hydrogen voltage-gated channel 1 | PC-5p-92816_49 |
| HVCN1 | hydrogen voltage-gated channel 1 | chi-miR-16b-5p_R+1 |
| HVCN1 | hydrogen voltage-gated channel 1 | gga-let-7g-3p_1ss22CT |
| HVCN1 | hydrogen voltage-gated channel 1 | gga-miR-16-5p |
| HVCN1 | hydrogen voltage-gated channel 1 | gga-miR-1677-3p_L+1 |
| HVCN1 | hydrogen voltage-gated channel 1 | gga-miR-1a-3p_R+1_1ss2GT |
| HVCN1 | hydrogen voltage-gated channel 1 | gga-miR-30a-5p_R+2_1ss13CT |
| HVCN1 | hydrogen voltage-gated channel 1 | gga-miR-30e-5p_R+5 |
| FAM214A | family with sequence similarity 214, member A | gga-miR-30a-5p_R+2_1ss13CT |
| FAM214A | family with sequence similarity 214, member A | gga-miR-30e-5p_R+5 |
| CELA2A | chymotrypsin-like elastase family, member 2A | gga-miR-1677-3p_L+1 |
| NCOA3 | nuclear receptor coactivator 3 | gga-let-7g-3p_1ss22CT |
| PPP1CC | protein phosphatase 1, catalytic subunit, gamma isozyme | PC-3p-52432_89 |
| PPP1CC | protein phosphatase 1, catalytic subunit, gamma isozyme | gga-let-7g-3p_1ss22CT |
| YIPF6 | Yip1 domain family, member 6 | gga-miR-34b-5p_L-1R+1 |
| AR | androgen receptor | PC-3p-52432_89 |
| AR | androgen receptor | PC-5p-233284_18 |
| AR | androgen receptor | gga-miR-1782_L+2R-1 |
| LOC100859686 | kynurenine--oxoglutarate transaminase 1-like | gga-miR-34b-5p_L-1R+1 |
| MTHFR | methylenetetrahydrofolate reductase (NAD(P)H) | PC-3p-173245_25 |
| MTHFR | methylenetetrahydrofolate reductase (NAD(P)H) | chi-miR-16b-5p_R+1 |
| MTHFR | methylenetetrahydrofolate reductase (NAD(P)H) | gga-miR-16-5p |
| P2RX5 | purinergic receptor P2X, ligand-gated ion channel, 5 | PC-3p-173245_25 |
| P2RX5 | purinergic receptor P2X, ligand-gated ion channel, 5 | PC-5p-233284_18 |
| P2RX5 | purinergic receptor P2X, ligand-gated ion channel, 5 | PC-5p-582426_7 |
| P2RX5 | purinergic receptor P2X, ligand-gated ion channel, 5 | chi-miR-16b-5p_R+1 |
| P2RX5 | purinergic receptor P2X, ligand-gated ion channel, 5 | gga-miR-16-5p |
| P2RX5 | purinergic receptor P2X, ligand-gated ion channel, 5 | gga-miR-1782_L+2R-1 |
| P2RX5 | purinergic receptor P2X, ligand-gated ion channel, 5 | hsa-miR-483-3p_L-1R+2 |
| P2RX5 | purinergic receptor P2X, ligand-gated ion channel, 5 | tgu-miR-2970-5p_1ss21GT |
| NPL | N-acetylneuraminate pyruvate lyase (dihydrodipicolinate synthase) | gga-miR-34b-5p_L-1R+1 |
| TAX1BP3 | Tax1 (human T-cell leukemia virus type I) binding protein 3 | chi-miR-16b-5p_R+1 |
| TAX1BP3 | Tax1 (human T-cell leukemia virus type I) binding protein 3 | gga-miR-16-5p |
| AGTRAP | angiotensin II receptor-associated protein | PC-5p-582426_7 |
| AGTRAP | angiotensin II receptor-associated protein | PC-5p-97606_47 |
| AGTRAP | angiotensin II receptor-associated protein | aca-miR-18a-5p_R-2 |
| ATXN2 | ataxin 2 | chi-miR-16b-5p_R+1 |
| ATXN2 | ataxin 2 | gga-miR-16-5p |
| C21H1ORF187 | chromosome 21 open reading frame, human C1orf187 | gga-miR-1677-3p_L+1 |
| MAD2L2 | MAD2 mitotic arrest deficient-like 2 (yeast) | PC-5p-113875_40 |
| LOC423008 | protein-L-isoaspartate (D-aspartate) O-methyltransferase-like | chi-miR-16b-5p_R+1 |
| LOC423008 | protein-L-isoaspartate (D-aspartate) O-methyltransferase-like | gga-miR-16-5p |
| LOC423008 | protein-L-isoaspartate (D-aspartate) O-methyltransferase-like | gga-miR-1782_L+2R-1 |
| C5H15ORF57 | chromosome 5 open reading frame, human C15orf57 | PC-3p-338480_12 |
| PDCD5 | programmed cell death 5 | chi-miR-16b-5p_R+1 |
| PDCD5 | programmed cell death 5 | gga-miR-16-5p |
| PDCD5 | programmed cell death 5 | gga-miR-30a-5p_R+2_1ss13CT |
| PDCD5 | programmed cell death 5 | gga-miR-30e-5p_R+5 |
| BRAP | BRCA1 associated protein | gga-let-7g-3p_1ss22CT |
| BRAP | BRCA1 associated protein | gga-miR-33-3p_L+1R+1 |
| SHISA5 | shisa homolog 5 (Xenopus laevis) | PC-5p-113875_40 |
| SHISA5 | shisa homolog 5 (Xenopus laevis) | PC-5p-71982_65 |
| SHISA5 | shisa homolog 5 (Xenopus laevis) | PC-5p-97606_47 |
| SHISA5 | shisa homolog 5 (Xenopus laevis) | aca-miR-18a-5p_R-2 |
| SHISA5 | shisa homolog 5 (Xenopus laevis) | aca-miR-363-3p_R+1 |
| SHISA5 | shisa homolog 5 (Xenopus laevis) | gga-let-7g-3p_1ss22CT |
| SHISA5 | shisa homolog 5 (Xenopus laevis) | oan-miR-363-3p_R+1 |
| KPNA7 | karyopherin alpha 7 (importin alpha 8) | hsa-miR-483-3p_L-1R+2 |
| SMG7 | Smg-7 homolog, nonsense mediated mRNA decay factor (C. elegans) | PC-3p-173245_25 |
| RGS9BP | regulator of G protein signaling 9 binding protein | PC-5p-92816_49 |
| RGS9BP | regulator of G protein signaling 9 binding protein | chi-miR-16b-5p_R+1 |
| RGS9BP | regulator of G protein signaling 9 binding protein | gga-miR-16-5p |
| RGS9BP | regulator of G protein signaling 9 binding protein | gga-miR-1677-3p_L+1 |
| RGS9BP | regulator of G protein signaling 9 binding protein | gga-miR-30a-5p_R+2_1ss13CT |
| RGS9BP | regulator of G protein signaling 9 binding protein | gga-miR-30e-5p_R+5 |
| CENPP | centromere protein P | gga-let-7g-3p_1ss22CT |
| MAPK6 | mitogen-activated protein kinase 6 | PC-3p-457530_9 |
| MAPK6 | mitogen-activated protein kinase 6 | PC-5p-92816_49 |
| MAPK6 | mitogen-activated protein kinase 6 | PC-5p-97606_47 |
| MAPK6 | mitogen-activated protein kinase 6 | gga-let-7g-3p_1ss22CT |
| MAPK6 | mitogen-activated protein kinase 6 | gga-miR-1a-3p_R+1_1ss2GT |
| PDAP1 | PDGFA associated protein 1 | gga-miR-1782_L+2R-1 |
| SET | SET nuclear oncogene | PC-5p-233284_18 |
| SET | SET nuclear oncogene | PC-5p-71982_65 |
| SET | SET nuclear oncogene | PC-5p-92816_49 |
| SET | SET nuclear oncogene | PC-5p-97606_47 |
| SET | SET nuclear oncogene | chi-miR-16b-5p_R+1 |
| SET | SET nuclear oncogene | gga-miR-16-5p |
| BUD31 | BUD31 homolog (S. cerevisiae) | PC-5p-97606_47 |
| BUD31 | BUD31 homolog (S. cerevisiae) | aca-miR-363-3p_R+1 |
| BUD31 | BUD31 homolog (S. cerevisiae) | oan-miR-363-3p_R+1 |
| CPSF4 | cleavage and polyadenylation specific factor 4, 30kDa | PC-5p-97606_47 |
| CPSF4 | cleavage and polyadenylation specific factor 4, 30kDa | chi-miR-16b-5p_R+1 |
| CPSF4 | cleavage and polyadenylation specific factor 4, 30kDa | gga-miR-16-5p |
| ULK2 | unc-51-like kinase 2 (C. elegans) | PC-5p-97606_47 |
| RGL1 | ral guanine nucleotide dissociation stimulator-like 1 | PC-5p-71982_65 |
| RGL1 | ral guanine nucleotide dissociation stimulator-like 1 | aca-miR-363-3p_R+1 |
| RGL1 | ral guanine nucleotide dissociation stimulator-like 1 | oan-miR-363-3p_R+1 |
| SLC7A9 | solute carrier family 7 (glycoprotein-associated amino acid transporter light chain, bo,+ system), member 9 | gga-miR-1782_L+2R-1 |
| CLIC2 | chloride intracellular channel 2 | gga-let-7g-3p_1ss22CT |
| CLIC2 | chloride intracellular channel 2 | gga-miR-1677-3p_L+1 |
| CLIC2 | chloride intracellular channel 2 | gga-miR-34b-5p_L-1R+1 |
| GLE1 | GLE1 RNA export mediator | PC-5p-233284_18 |
| C14H17ORF103 | chromosome 14 open reading frame, human C17orf103 | gga-miR-16-5p |
| NOL8 | nucleolar protein 8 | PC-5p-582426_7 |
| NOL8 | nucleolar protein 8 | gga-miR-1782_L+2R-1 |
| NOL8 | nucleolar protein 8 | gga-miR-30a-5p_R+2_1ss13CT |
| NOL8 | nucleolar protein 8 | gga-miR-30e-5p_R+5 |
| DHRS7B | dehydrogenase/reductase (SDR family) member 7B | PC-3p-52432_89 |
| TMEM116 | transmembrane protein 116 | gga-miR-1329-3p |
| SCG3 | secretogranin III | aca-miR-18a-5p_R-2 |
| SCG3 | secretogranin III | gga-let-7g-3p_1ss22CT |
| PSAP | prosaposin | PC-5p-97606_47 |
| PSAP | prosaposin | chi-miR-16b-5p_R+1 |
| PSAP | prosaposin | gga-let-7g-3p_1ss22CT |
| PSAP | prosaposin | gga-miR-16-5p |
| PSAP | prosaposin | gga-miR-33-3p_L+1R+1 |
| PSAP | prosaposin | tgu-miR-2970-5p_1ss21GT |
| USP22 | ubiquitin specific peptidase 22 | PC-5p-113875_40 |
| USP22 | ubiquitin specific peptidase 22 | PC-5p-97606_47 |
| AIFM2 | apoptosis-inducing factor, mitochondrion-associated, 2 | PC-5p-92816_49 |
| PTGES2 | prostaglandin E synthase 2 | PC-5p-71982_65 |
| TSEN15 | tRNA splicing endonuclease 15 homolog (S. cerevisiae) | PC-5p-97606_47 |
| C17H9ORF16 | chromosome 17 open reading frame, human C9orf16 | hsa-miR-483-3p_L-1R+2 |
| PHKA1 | phosphorylase kinase, alpha 1 (muscle) | chi-miR-16b-5p_R+1 |
| PHKA1 | phosphorylase kinase, alpha 1 (muscle) | gga-miR-16-5p |
| PPIF | peptidylprolyl isomerase F | PC-3p-173245_25 |
| PPIF | peptidylprolyl isomerase F | aca-miR-363-3p_R+1 |
| PPIF | peptidylprolyl isomerase F | hsa-miR-483-3p_L-1R+2 |
| PPIF | peptidylprolyl isomerase F | oan-miR-363-3p_R+1 |
| FAM129A | family with sequence similarity 129, member A | aca-miR-425-3p_L-2R+1_1ss3AT |
| FAM129A | family with sequence similarity 129, member A | hsa-miR-483-3p_L-1R+2 |
| IARS | isoleucyl-tRNA synthetase | chi-miR-16b-5p_R+1 |
| IARS | isoleucyl-tRNA synthetase | gga-miR-16-5p |
| FLCN | folliculin | chi-miR-16b-5p_R+1 |
| FLCN | folliculin | gga-miR-16-5p |
| RNF2 | ring finger protein 2 | PC-3p-457530_9 |
| COPS3 | COP9 constitutive photomorphogenic homolog subunit 3 (Arabidopsis) | PC-5p-233284_18 |
| COPS3 | COP9 constitutive photomorphogenic homolog subunit 3 (Arabidopsis) | PC-5p-71982_65 |
| COPS3 | COP9 constitutive photomorphogenic homolog subunit 3 (Arabidopsis) | gga-miR-1782_L+2R-1 |
| COPS3 | COP9 constitutive photomorphogenic homolog subunit 3 (Arabidopsis) | hsa-miR-483-3p_L-1R+2 |
| WSCD2 | WSC domain containing 2 | chi-miR-16b-5p_R+1 |
| WSCD2 | WSC domain containing 2 | gga-miR-1329-3p |
| WSCD2 | WSC domain containing 2 | gga-miR-16-5p |
| MED9 | mediator complex subunit 9 | PC-5p-71982_65 |
| NUP62 | nucleoporin 62kDa | aca-miR-425-3p_L-2R+1_1ss3AT |
| RASD1 | RAS, dexamethasone-induced 1 | PC-5p-97606_47 |
| RASD1 | RAS, dexamethasone-induced 1 | gga-miR-30a-5p_R+2_1ss13CT |
| RASD1 | RAS, dexamethasone-induced 1 | gga-miR-30e-5p_R+5 |
| CEBPG | CCAAT/enhancer binding protein (C/EBP), gamma | PC-3p-457530_9 |
| CEBPG | CCAAT/enhancer binding protein (C/EBP), gamma | PC-5p-113875_40 |
| RPS24 | ribosomal protein S24 | gga-miR-7468-3p_L+3 |
| PHACTR3 | phosphatase and actin regulator 3 | chi-miR-16b-5p_R+1 |
| PHACTR3 | phosphatase and actin regulator 3 | gga-miR-16-5p |
| PEMT | phosphatidylethanolamine N-methyltransferase | gga-miR-30a-5p_R+2_1ss13CT |
| PEMT | phosphatidylethanolamine N-methyltransferase | gga-miR-30e-5p_R+5 |
| PEMT | phosphatidylethanolamine N-methyltransferase | gga-miR-34b-5p_L-1R+1 |
| VPS53 | vacuolar protein sorting 53 homolog (S. cerevisiae) | chi-miR-16b-5p_R+1 |
| VPS53 | vacuolar protein sorting 53 homolog (S. cerevisiae) | gga-miR-16-5p |
| MTFR1 | mitochondrial fission regulator 1 | chi-miR-16b-5p_R+1 |
| MTFR1 | mitochondrial fission regulator 1 | gga-miR-16-5p |
| PEPD | peptidase D | PC-5p-113875_40 |
| PEPD | peptidase D | chi-miR-16b-5p_R+1 |
| PEPD | peptidase D | gga-miR-16-5p |
| MYEF2 | myelin expression factor 2 | PC-5p-37716_121 |
| MYEF2 | myelin expression factor 2 | PC-5p-92816_49 |
| MYEF2 | myelin expression factor 2 | chi-miR-16b-5p_R+1 |
| MYEF2 | myelin expression factor 2 | gga-miR-16-5p |
| MYEF2 | myelin expression factor 2 | gga-miR-1677-3p_L+1 |
| MYEF2 | myelin expression factor 2 | gga-miR-33-3p_L+1R+1 |
| OPNP | opsin, pineal | PC-5p-113875_40 |
| C17ORF39 | chromosome 14 open reading frame, human C17orf39 | gga-miR-1782_L+2R-1 |
| CDC25A | cell division cycle 25 homolog A (S. pombe) | gga-miR-1456-3p_R+1 |
| LSM14A | LSM14A, SCD6 homolog A (S. cerevisiae) | PC-5p-233284_18 |
| LSM14A | LSM14A, SCD6 homolog A (S. cerevisiae) | PC-5p-92816_49 |
| SLC12A1 | solute carrier family 12 (sodium/potassium/chloride transporters), member 1 | PC-5p-71982_65 |
| COL4A4 | collagen, type IV, alpha 4 | PC-5p-233284_18 |
| DRG2 | developmentally regulated GTP binding protein 2 | PC-5p-97606_47 |
| DRG2 | developmentally regulated GTP binding protein 2 | chi-miR-16b-5p_R+1 |
| DRG2 | developmentally regulated GTP binding protein 2 | gga-miR-16-5p |
| DUT | deoxyuridine triphosphatase | PC-5p-113875_40 |
| DUT | deoxyuridine triphosphatase | PC-5p-233284_18 |
| DUT | deoxyuridine triphosphatase | PC-5p-71982_65 |
| DUT | deoxyuridine triphosphatase | chi-miR-16b-5p_R+1 |
| DUT | deoxyuridine triphosphatase | gga-miR-16-5p |
| DUT | deoxyuridine triphosphatase | gga-miR-1782_L+2R-1 |
| DUT | deoxyuridine triphosphatase | gga-miR-1805-5p_L-1 |
| DUT | deoxyuridine triphosphatase | gga-miR-1a-3p_R+1_1ss2GT |
| RHBDD1 | rhomboid domain containing 1 | PC-5p-233284_18 |
| RHBDD1 | rhomboid domain containing 1 | gga-let-7g-3p_1ss22CT |
| URM1 | ubiquitin related modifier 1 | gga-miR-1456-3p_R+1 |
| PPARG | peroxisome proliferator-activated receptor gamma | PC-3p-173245_25 |
| ALKBH5 | alkB, alkylation repair homolog 5 (E. coli) | PC-5p-233284_18 |
| TSEN2 | tRNA splicing endonuclease 2 homolog (S. cerevisiae) | PC-5p-92816_49 |
| TSEN2 | tRNA splicing endonuclease 2 homolog (S. cerevisiae) | aca-miR-425-3p_L-2R+1_1ss3AT |
| TSEN2 | tRNA splicing endonuclease 2 homolog (S. cerevisiae) | gga-let-7g-3p_1ss22CT |
| TSEN2 | tRNA splicing endonuclease 2 homolog (S. cerevisiae) | tgu-miR-2970-5p_1ss21GT |
| ALKBH2 | alkB, alkylation repair homolog 2 (E. coli) | hsa-miR-483-3p_L-1R+2 |
| TAF7 | TAF7 RNA polymerase II, TATA box binding protein (TBP)-associated factor, 55kDa | PC-3p-173245_25 |
| TAF7 | TAF7 RNA polymerase II, TATA box binding protein (TBP)-associated factor, 55kDa | PC-3p-52432_89 |
| SHC4 | SHC (Src homology 2 domain containing) family, member 4 | gga-miR-33-3p_L+1R+1 |
| ISY1 | ISY1 splicing factor homolog (S. cerevisiae) | PC-3p-457530_9 |
| ISY1 | ISY1 splicing factor homolog (S. cerevisiae) | tgu-miR-2970-5p_1ss21GT |
| DHX30 | DEAH (Asp-Glu-Ala-His) box polypeptide 30 | gga-miR-1677-3p_L+1 |
| DHX30 | DEAH (Asp-Glu-Ala-His) box polypeptide 30 | gga-miR-34b-5p_L-1R+1 |
| TOP3A | topoisomerase (DNA) III alpha | PC-5p-37716_121 |
| TOP3A | topoisomerase (DNA) III alpha | chi-miR-16b-5p_R+1 |
| TOP3A | topoisomerase (DNA) III alpha | gga-miR-16-5p |
| CENPI | centromere protein I | hsa-miR-483-3p_L-1R+2 |
| CDK9 | cyclin-dependent kinase 9 | chi-miR-16b-5p_R+1 |
| CDK9 | cyclin-dependent kinase 9 | gga-miR-16-5p |
| MAP4K4 | mitogen-activated protein kinase kinase kinase kinase 4 | PC-5p-113875_40 |
| DKC1 | dyskeratosis congenita 1, dyskerin | hsa-miR-483-3p_L-1R+2 |
| ADK | adenosine kinase | tgu-miR-2970-5p_1ss21GT |
| PRPSAP2 | phosphoribosyl pyrophosphate synthetase-associated protein 2 | tgu-miR-2970-5p_1ss21GT |
| PLA2G4A | phospholipase A2, group IVA (cytosolic, calcium-dependent) | chi-miR-16b-5p_R+1 |
| PLA2G4A | phospholipase A2, group IVA (cytosolic, calcium-dependent) | gga-miR-16-5p |
| DPM2 | dolichyl-phosphate mannosyltransferase polypeptide 2, regulatory subunit | PC-5p-97606_47 |
| DPM2 | dolichyl-phosphate mannosyltransferase polypeptide 2, regulatory subunit | gga-miR-34b-5p_L-1R+1 |
| PTGS2 | prostaglandin-endoperoxide synthase 2 (prostaglandin G/H synthase and cyclooxygenase) | PC-5p-92816_49 |
| PTGS2 | prostaglandin-endoperoxide synthase 2 (prostaglandin G/H synthase and cyclooxygenase) | PC-5p-97606_47 |
| PTGS2 | prostaglandin-endoperoxide synthase 2 (prostaglandin G/H synthase and cyclooxygenase) | gga-let-7g-3p_1ss22CT |
| PTGS2 | prostaglandin-endoperoxide synthase 2 (prostaglandin G/H synthase and cyclooxygenase) | gga-miR-1782_L+2R-1 |
| PTGS2 | prostaglandin-endoperoxide synthase 2 (prostaglandin G/H synthase and cyclooxygenase) | tgu-miR-2970-5p_1ss21GT |
| AP3M1 | adaptor-related protein complex 3, mu 1 subunit | PC-3p-52432_89 |
| AP3M1 | adaptor-related protein complex 3, mu 1 subunit | gga-miR-30a-5p_R+2_1ss13CT |
| AP3M1 | adaptor-related protein complex 3, mu 1 subunit | gga-miR-30e-5p_R+5 |
| AP3M1 | adaptor-related protein complex 3, mu 1 subunit | gga-miR-34b-5p_L-1R+1 |
| MPP1 | membrane protein, palmitoylated 1, 55kDa | aca-miR-363-3p_R+1 |
| MPP1 | membrane protein, palmitoylated 1, 55kDa | oan-miR-363-3p_R+1 |
| NUP210 | nucleoporin 210kDa | chi-miR-16b-5p_R+1 |
| NUP210 | nucleoporin 210kDa | gga-miR-16-5p |
| C8H1ORF27 | chromosome 8 open reading frame, human C1orf27 | aca-miR-363-3p_R+1 |
| C8H1ORF27 | chromosome 8 open reading frame, human C1orf27 | oan-miR-363-3p_R+1 |
| C8H1ORF27 | chromosome 8 open reading frame, human C1orf27 | tgu-miR-2970-5p_1ss21GT |
| TRIM37 | tripartite motif containing 37 | gga-let-7g-3p_1ss22CT |
| TRIM37 | tripartite motif containing 37 | gga-miR-1782_L+2R-1 |
| PLAU | plasminogen activator, urokinase | gga-let-7g-3p_1ss22CT |
| HDAC11 | histone deacetylase 11 | PC-5p-97606_47 |
| NDST2 | N-deacetylase/N-sulfotransferase (heparan glucosaminyl) 2 | PC-5p-233284_18 |
| NDST2 | N-deacetylase/N-sulfotransferase (heparan glucosaminyl) 2 | gga-miR-34b-5p_L-1R+1 |
| CES1 | carboxylesterase 1 (monocyte/macrophage serine esterase 1) | gga-miR-7468-3p_L+3 |
| SWT1 | SWT1 RNA endoribonuclease homolog (S. cerevisiae) | gga-miR-1677-3p_L+1 |
| FAM96B | family with sequence similarity 96, member B | gga-let-7g-3p_1ss22CT |
| FAM96B | family with sequence similarity 96, member B | gga-miR-1329-3p |
| SERPINE2 | serpin peptidase inhibitor, clade E (nexin, plasminogen activator inhibitor type 1), member 2 | PC-5p-582426_7 |
| SERPINE2 | serpin peptidase inhibitor, clade E (nexin, plasminogen activator inhibitor type 1), member 2 | gga-miR-7468-3p_L+3 |
| CLTC | clathrin, heavy chain (Hc) | aca-miR-363-3p_R+1 |
| CLTC | clathrin, heavy chain (Hc) | oan-miR-363-3p_R+1 |
| LOC423740 | neurotrypsin-like | PC-5p-71982_65 |
| GIT2 | G protein-coupled receptor kinase interactor 2 | gga-miR-34b-5p_L-1R+1 |
| WDFY1 | WD repeat and FYVE domain containing 1 | PC-3p-173245_25 |
| WDFY1 | WD repeat and FYVE domain containing 1 | aca-miR-18a-5p_R-2 |
| WDFY1 | WD repeat and FYVE domain containing 1 | chi-miR-16b-5p_R+1 |
| WDFY1 | WD repeat and FYVE domain containing 1 | gga-miR-16-5p |
| WDFY1 | WD repeat and FYVE domain containing 1 | gga-miR-1a-3p_R+1_1ss2GT |
| FUT11 | fucosyltransferase 11 (alpha (1,3) fucosyltransferase) | PC-5p-113875_40 |
| TUBD1 | tubulin, delta 1 | gga-miR-1805-5p_L-1 |
| FAM120A | family with sequence similarity 120A | PC-3p-457530_9 |
| SEC24C | SEC24 family, member C (S. cerevisiae) | gga-let-7g-3p_1ss22CT |
| MRPL16 | mitochondrial ribosomal protein L16 | PC-5p-97606_47 |
| RPS6KB1 | ribosomal protein S6 kinase, 70kDa, polypeptide 1 | PC-5p-113875_40 |
| RPS6KB1 | ribosomal protein S6 kinase, 70kDa, polypeptide 1 | PC-5p-92816_49 |
| RPS6KB1 | ribosomal protein S6 kinase, 70kDa, polypeptide 1 | chi-miR-16b-5p_R+1 |
| RPS6KB1 | ribosomal protein S6 kinase, 70kDa, polypeptide 1 | gga-miR-16-5p |
| RPS6KB1 | ribosomal protein S6 kinase, 70kDa, polypeptide 1 | gga-miR-1a-3p_R+1_1ss2GT |
| ACSL3 | acyl-CoA synthetase long-chain family member 3 | PC-3p-173245_25 |
| ACSL3 | acyl-CoA synthetase long-chain family member 3 | PC-3p-338480_12 |
| MED13 | mediator complex subunit 13 | gga-miR-7468-3p_L+3 |
| CDC14A | CDC14 cell division cycle 14 homolog A (S. cerevisiae) | chi-miR-16b-5p_R+1 |
| CDC14A | CDC14 cell division cycle 14 homolog A (S. cerevisiae) | gga-miR-16-5p |
| BICD2 | bicaudal D homolog 2 (Drosophila) | chi-miR-16b-5p_R+1 |
| BICD2 | bicaudal D homolog 2 (Drosophila) | gga-miR-16-5p |
| BICD2 | bicaudal D homolog 2 (Drosophila) | gga-miR-34b-5p_L-1R+1 |
| EPHA4 | EPH receptor A4 | PC-5p-582426_7 |
| VCAM1 | vascular cell adhesion molecule 1 | gga-let-7g-3p_1ss22CT |
| TK2 | thymidine kinase 2, mitochondrial | PC-3p-173245_25 |
| TK2 | thymidine kinase 2, mitochondrial | gga-miR-1805-5p_L-1 |
| BEAN1 | brain expressed, associated with NEDD4, 1 | PC-5p-233284_18 |
| SEC22C | SEC22 vesicle trafficking protein homolog C (S. cerevisiae) | PC-3p-173245_25 |
| INTS2 | integrator complex subunit 2 | PC-5p-92816_49 |
| INTS2 | integrator complex subunit 2 | aca-miR-363-3p_R+1 |
| INTS2 | integrator complex subunit 2 | gga-miR-1782_L+2R-1 |
| INTS2 | integrator complex subunit 2 | gga-miR-34b-5p_L-1R+1 |
| INTS2 | integrator complex subunit 2 | oan-miR-363-3p_R+1 |
| CDH5 | cadherin 5, type 2 (vascular endothelium) | PC-5p-71982_65 |
| CDH5 | cadherin 5, type 2 (vascular endothelium) | PC-5p-92816_49 |
| CDH5 | cadherin 5, type 2 (vascular endothelium) | chi-miR-16b-5p_R+1 |
| CDH5 | cadherin 5, type 2 (vascular endothelium) | gga-miR-16-5p |
| CDH11 | cadherin 11, type 2, OB-cadherin (osteoblast) | PC-5p-113875_40 |
| CDH11 | cadherin 11, type 2, OB-cadherin (osteoblast) | gga-miR-33-3p_L+1R+1 |
| DBT | dihydrolipoamide branched chain transacylase E2 | PC-3p-457530_9 |
| LMF1 | lipase maturation factor 1 | PC-5p-113875_40 |
| SLC25A36 | solute carrier family 25 (pyrimidine nucleotide carrier ), member 36 | PC-5p-582426_7 |
| SLC25A36 | solute carrier family 25 (pyrimidine nucleotide carrier ), member 36 | chi-miR-16b-5p_R+1 |
| SLC25A36 | solute carrier family 25 (pyrimidine nucleotide carrier ), member 36 | gga-miR-16-5p |
| SLC25A36 | solute carrier family 25 (pyrimidine nucleotide carrier ), member 36 | gga-miR-30a-5p_R+2_1ss13CT |
| SLC25A36 | solute carrier family 25 (pyrimidine nucleotide carrier ), member 36 | gga-miR-30e-5p_R+5 |
| RRP1B | ribosomal RNA processing 1 homolog B (S. cerevisiae) | PC-5p-71982_65 |
| LOC416530 | uncharacterized LOC416530 | aca-miR-18a-5p_R-2 |
| CYB5R2 | cytochrome b5 reductase 2 | PC-3p-457530_9 |
| CYB5R2 | cytochrome b5 reductase 2 | aca-miR-18a-5p_R-2 |
| BCAS3 | breast carcinoma amplified sequence 3 | PC-3p-338480_12 |
| BCAS3 | breast carcinoma amplified sequence 3 | aca-miR-363-3p_R+1 |
| BCAS3 | breast carcinoma amplified sequence 3 | oan-miR-363-3p_R+1 |
| HHATL | hedgehog acyltransferase-like | gga-miR-1677-3p_L+1 |
| SASS6 | spindle assembly 6 homolog (C. elegans) | gga-miR-1677-3p_L+1 |
| APPBP2 | amyloid beta precursor protein (cytoplasmic tail) binding protein 2 | PC-5p-233284_18 |
| APPBP2 | amyloid beta precursor protein (cytoplasmic tail) binding protein 2 | gga-miR-1677-3p_L+1 |
| APPBP2 | amyloid beta precursor protein (cytoplasmic tail) binding protein 2 | gga-miR-33-3p_L+1R+1 |
| RBP2 | retinol binding protein 2, cellular | PC-5p-233284_18 |
| IBA57 | IBA57, iron-sulfur cluster assembly homolog (S. cerevisiae) | chi-miR-16b-5p_R+1 |
| IBA57 | IBA57, iron-sulfur cluster assembly homolog (S. cerevisiae) | gga-miR-16-5p |
| SLC35A3 | solute carrier family 35 (UDP-N-acetylglucosamine (UDP-GlcNAc) transporter), member A3 | gga-miR-1782_L+2R-1 |
| SLC35A3 | solute carrier family 35 (UDP-N-acetylglucosamine (UDP-GlcNAc) transporter), member A3 | gga-miR-30a-5p_R+2_1ss13CT |
| SLC35A3 | solute carrier family 35 (UDP-N-acetylglucosamine (UDP-GlcNAc) transporter), member A3 | gga-miR-30e-5p_R+5 |
| PPIC | peptidylprolyl isomerase C (cyclophilin C) | aca-miR-363-3p_R+1 |
| PPIC | peptidylprolyl isomerase C (cyclophilin C) | gga-miR-7468-3p_L+3 |
| PPIC | peptidylprolyl isomerase C (cyclophilin C) | oan-miR-363-3p_R+1 |
| ADAMTS18 | ADAM metallopeptidase with thrombospondin type 1 motif, 18 | gga-miR-1782_L+2R-1 |
| USP32 | ubiquitin specific peptidase 32 | PC-3p-338480_12 |
| GATA5 | GATA binding protein 5 | PC-5p-97606_47 |
| GATA5 | GATA binding protein 5 | aca-miR-363-3p_R+1 |
| GATA5 | GATA binding protein 5 | oan-miR-363-3p_R+1 |
| PTH | parathyroid hormone | PC-3p-338480_12 |
| PTH | parathyroid hormone | PC-5p-71982_65 |
| PTH | parathyroid hormone | chi-miR-16b-5p_R+1 |
| PTH | parathyroid hormone | gga-miR-16-5p |
| CA4 | carbonic anhydrase IV | chi-miR-16b-5p_R+1 |
| CA4 | carbonic anhydrase IV | gga-miR-16-5p |
| CHDH | choline dehydrogenase | PC-5p-92816_49 |
| BTBD10 | BTB (POZ) domain containing 10 | gga-miR-30a-5p_R+2_1ss13CT |
| BTBD10 | BTB (POZ) domain containing 10 | gga-miR-30e-5p_R+5 |
| SELK | selenoprotein K | gga-miR-33-3p_L+1R+1 |
| GGNBP2 | gametogenetin binding protein 2 | gga-let-7g-3p_1ss22CT |
| HAGHL | hydroxyacylglutathione hydrolase-like | PC-3p-457530_9 |
| HAGHL | hydroxyacylglutathione hydrolase-like | PC-3p-52432_89 |
| HAGHL | hydroxyacylglutathione hydrolase-like | PC-5p-233284_18 |
| DHRS11 | dehydrogenase/reductase (SDR family) member 11 | PC-5p-92816_49 |
| MRM1 | mitochondrial rRNA methyltransferase 1 homolog (S. cerevisiae) | chi-miR-16b-5p_R+1 |
| MRM1 | mitochondrial rRNA methyltransferase 1 homolog (S. cerevisiae) | gga-miR-16-5p |
| BCMO1 | beta-carotene 15,15'-monooxygenase 1 | gga-miR-1805-5p_L-1 |
| BCMO1 | beta-carotene 15,15'-monooxygenase 1 | gga-miR-1a-3p_R+1_1ss2GT |
| BCMO1 | beta-carotene 15,15'-monooxygenase 1 | gga-miR-34b-5p_L-1R+1 |
| HS3ST6 | heparan sulfate (glucosamine) 3-O-sulfotransferase 6 | PC-5p-37716_121 |
| JMJD4 | jumonji domain containing 4 | PC-3p-52432_89 |
| JMJD4 | jumonji domain containing 4 | PC-5p-97606_47 |
| JMJD4 | jumonji domain containing 4 | gga-miR-1782_L+2R-1 |
| FRRS1 | ferric-chelate reductase 1 | gga-miR-1782_L+2R-1 |
| PDLIM1 | PDZ and LIM domain 1 | PC-5p-113875_40 |
| PDLIM1 | PDZ and LIM domain 1 | chi-miR-16b-5p_R+1 |
| MYL3 | myosin, light chain 3, alkali; ventricular, skeletal, slow | PC-5p-582426_7 |
| MYL3 | myosin, light chain 3, alkali; ventricular, skeletal, slow | PC-5p-92816_49 |
| TADA2A | transcriptional adaptor 2A | chi-miR-16b-5p_R+1 |
| TADA2A | transcriptional adaptor 2A | gga-miR-16-5p |
| TADA2A | transcriptional adaptor 2A | gga-miR-1677-3p_L+1 |
| PLCG2 | phospholipase C, gamma 2 (phosphatidylinositol-specific) | gga-let-7g-3p_1ss22CT |
| PIK3CB | phosphoinositide-3-kinase, catalytic, beta polypeptide | PC-5p-233284_18 |
| PIK3CB | phosphoinositide-3-kinase, catalytic, beta polypeptide | aca-miR-363-3p_R+1 |
| PIK3CB | phosphoinositide-3-kinase, catalytic, beta polypeptide | gga-miR-1677-3p_L+1 |
| PIK3CB | phosphoinositide-3-kinase, catalytic, beta polypeptide | oan-miR-363-3p_R+1 |
| TAF1 | TAF1 RNA polymerase II, TATA box binding protein (TBP)-associated factor, 250kDa | PC-5p-37716_121 |
| NDUFB10 | NADH dehydrogenase (ubiquinone) 1 beta subcomplex, 10, 22kDa | PC-3p-338480_12 |
| NDUFB10 | NADH dehydrogenase (ubiquinone) 1 beta subcomplex, 10, 22kDa | gga-let-7g-3p_1ss22CT |
| SYNRG | synergin, gamma | aca-miR-363-3p_R+1 |
| SYNRG | synergin, gamma | oan-miR-363-3p_R+1 |
| LPPR5 | lipid phosphate phosphatase-related protein type 5 | PC-5p-97606_47 |
| PNAT10 | N-acetyltransferase, pineal gland isozyme NAT-10 | PC-3p-173245_25 |
| PNAT10 | N-acetyltransferase, pineal gland isozyme NAT-10 | PC-5p-92816_49 |
| PNAT10 | N-acetyltransferase, pineal gland isozyme NAT-10 | PC-5p-97606_47 |
| PNAT10 | N-acetyltransferase, pineal gland isozyme NAT-10 | aca-miR-363-3p_R+1 |
| PNAT10 | N-acetyltransferase, pineal gland isozyme NAT-10 | gga-miR-33-3p_L+1R+1 |
| PNAT10 | N-acetyltransferase, pineal gland isozyme NAT-10 | oan-miR-363-3p_R+1 |
| RHOG | ras homolog family member G | PC-3p-338480_12 |
| RHOG | ras homolog family member G | PC-3p-52432_89 |
| RHOG | ras homolog family member G | PC-5p-71982_65 |
| RHOG | ras homolog family member G | gga-miR-30a-5p_R+2_1ss13CT |
| RHOG | ras homolog family member G | gga-miR-30e-5p_R+5 |
| RHOG | ras homolog family member G | hsa-miR-483-3p_L-1R+2 |
| MPHOSPH6 | M-phase phosphoprotein 6 | chi-miR-16b-5p_R+1 |
| MPHOSPH6 | M-phase phosphoprotein 6 | gga-miR-16-5p |
| ARHGEF3 | Rho guanine nucleotide exchange factor (GEF) 3 | gga-let-7g-3p_1ss22CT |
| ARHGEF3 | Rho guanine nucleotide exchange factor (GEF) 3 | gga-miR-30a-5p_R+2_1ss13CT |
| ARHGEF3 | Rho guanine nucleotide exchange factor (GEF) 3 | gga-miR-30e-5p_R+5 |
| CCDC12 | coiled-coil domain containing 12 | PC-3p-173245_25 |
| WFDC1 | WAP four-disulfide core domain 1 | PC-3p-338480_12 |
| WFDC1 | WAP four-disulfide core domain 1 | PC-5p-233284_18 |
| WFDC1 | WAP four-disulfide core domain 1 | gga-miR-7468-3p_L+3 |
| NONO | non-POU domain containing, octamer-binding | gga-let-7g-3p_1ss22CT |
| NONO | non-POU domain containing, octamer-binding | gga-miR-1782_L+2R-1 |
| LOC768701 | uncharacterized protein KIAA1671 homolog | PC-5p-113875_40 |
| LOC768701 | uncharacterized protein KIAA1671 homolog | chi-miR-16b-5p_R+1 |
| LOC768701 | uncharacterized protein KIAA1671 homolog | gga-miR-16-5p |
| LOC768701 | uncharacterized protein KIAA1671 homolog | gga-miR-1782_L+2R-1 |
| ADRBK2 | adrenergic, beta, receptor kinase 2 | gga-miR-30a-5p_R+2_1ss13CT |
| ADRBK2 | adrenergic, beta, receptor kinase 2 | gga-miR-30e-5p_R+5 |
| ZMYM3 | zinc finger, MYM-type 3 | PC-5p-113875_40 |
| ASB14 | ankyrin repeat and SOCS box containing 14 | gga-miR-34b-5p_L-1R+1 |
| PIK3AP1 | phosphoinositide-3-kinase adaptor protein 1 | gga-miR-1677-3p_L+1 |
| ST6GAL1 | ST6 beta-galactosamide alpha-2,6-sialyltranferase 1 | PC-5p-113875_40 |
| ST6GAL1 | ST6 beta-galactosamide alpha-2,6-sialyltranferase 1 | aca-miR-363-3p_R+1 |
| ST6GAL1 | ST6 beta-galactosamide alpha-2,6-sialyltranferase 1 | oan-miR-363-3p_R+1 |
| ST6GAL1 | ST6 beta-galactosamide alpha-2,6-sialyltranferase 1 | tgu-miR-2970-5p_1ss21GT |
| DKK3 | dickkopf homolog 3 (Xenopus laevis) | aca-miR-363-3p_R+1 |
| DKK3 | dickkopf homolog 3 (Xenopus laevis) | gga-let-7g-3p_1ss22CT |
| DKK3 | dickkopf homolog 3 (Xenopus laevis) | oan-miR-363-3p_R+1 |
| EIF4G2 | eukaryotic translation initiation factor 4 gamma, 2 | PC-5p-71982_65 |
| EVI2A | ecotropic viral integration site 2A | gga-miR-1a-3p_R+1_1ss2GT |
| SYNGR3 | synaptogyrin 3 | PC-5p-37716_121 |
| SYNGR3 | synaptogyrin 3 | chi-miR-16b-5p_R+1 |
| SYNGR3 | synaptogyrin 3 | gga-let-7g-3p_1ss22CT |
| SYNGR3 | synaptogyrin 3 | gga-miR-16-5p |
| KLHL18 | kelch-like 18 (Drosophila) | PC-3p-173245_25 |
| KLHL18 | kelch-like 18 (Drosophila) | PC-5p-113875_40 |
| KLHL18 | kelch-like 18 (Drosophila) | PC-5p-233284_18 |
| KLHL18 | kelch-like 18 (Drosophila) | PC-5p-37716_121 |
| KLHL18 | kelch-like 18 (Drosophila) | PC-5p-97606_47 |
| KLHL18 | kelch-like 18 (Drosophila) | chi-miR-16b-5p_R+1 |
| KLHL18 | kelch-like 18 (Drosophila) | gga-miR-16-5p |
| SLC44A3 | solute carrier family 44, member 3 | PC-5p-71982_65 |
| NTHL1 | nth endonuclease III-like 1 (E. coli) | PC-3p-173245_25 |
| NTHL1 | nth endonuclease III-like 1 (E. coli) | chi-miR-16b-5p_R+1 |
| NTHL1 | nth endonuclease III-like 1 (E. coli) | gga-miR-16-5p |
| CTR9 | Ctr9, Paf1/RNA polymerase II complex component, homolog (S. cerevisiae) | gga-miR-33-3p_L+1R+1 |
| TFIP11 | tuftelin interacting protein 11 | PC-5p-582426_7 |
| TFIP11 | tuftelin interacting protein 11 | PC-5p-71982_65 |
| TPST2 | tyrosylprotein sulfotransferase 2 | hsa-miR-483-3p_L-1R+2 |
| LYVE1 | lymphatic vessel endothelial hyaluronan receptor 1 | tgu-miR-2970-5p_1ss21GT |
| RNF141 | ring finger protein 141 | PC-3p-173245_25 |
| RNF141 | ring finger protein 141 | PC-5p-582426_7 |
| RNF141 | ring finger protein 141 | aca-miR-363-3p_R+1 |
| RNF141 | ring finger protein 141 | gga-let-7g-3p_1ss22CT |
| RNF141 | ring finger protein 141 | gga-miR-1a-3p_R+1_1ss2GT |
| RNF141 | ring finger protein 141 | gga-miR-33-3p_L+1R+1 |
| RNF141 | ring finger protein 141 | gga-miR-7468-3p_L+3 |
| RNF141 | ring finger protein 141 | oan-miR-363-3p_R+1 |
| ABCD3 | ATP-binding cassette, sub-family D (ALD), member 3 | PC-5p-92816_49 |
| ABCD3 | ATP-binding cassette, sub-family D (ALD), member 3 | PC-5p-97606_47 |
| ABCD3 | ATP-binding cassette, sub-family D (ALD), member 3 | gga-let-7g-3p_1ss22CT |
| ABCD3 | ATP-binding cassette, sub-family D (ALD), member 3 | gga-miR-30a-5p_R+2_1ss13CT |
| ABCD3 | ATP-binding cassette, sub-family D (ALD), member 3 | gga-miR-30e-5p_R+5 |
| ABCD3 | ATP-binding cassette, sub-family D (ALD), member 3 | tgu-miR-2970-5p_1ss21GT |
| DENND6A | family with sequence similarity 116, member A | PC-5p-97606_47 |
| DENND6A | family with sequence similarity 116, member A | chi-miR-16b-5p_R+1 |
| DENND6A | family with sequence similarity 116, member A | gga-miR-16-5p |
| DENND6A | family with sequence similarity 116, member A | gga-miR-1782_L+2R-1 |
| SNX12 | sorting nexin 12 | PC-5p-37716_121 |
| WSB1 | WD repeat and SOCS box containing 1 | PC-3p-52432_89 |
| CDX1 | caudal type homeobox 1 | PC-3p-52432_89 |
| CDX1 | caudal type homeobox 1 | PC-5p-113875_40 |
| CRISPLD2 | cysteine-rich secretory protein LCCL domain containing 2 | PC-5p-113875_40 |
| CHEK2 | checkpoint kinase 2 | PC-5p-71982_65 |
| ABHD6 | abhydrolase domain containing 6 | PC-5p-71982_65 |
| HIF1AN | hypoxia inducible factor 1, alpha subunit inhibitor | aca-miR-18a-5p_R-2 |
| NLK | nemo-like kinase | PC-3p-282885_15 |
| NLK | nemo-like kinase | gga-let-7g-3p_1ss22CT |
| C20H20ORF11 | chromosome 20 open reading frame, human C20orf11 | PC-5p-92816_49 |
| C20H20ORF11 | chromosome 20 open reading frame, human C20orf11 | gga-let-7g-3p_1ss22CT |
| TMEM97 | transmembrane protein 97 | PC-5p-37716_121 |
| SLC30A4 | solute carrier family 30 (zinc transporter), member 4 | PC-3p-457530_9 |
| HSCB | HscB iron-sulfur cluster co-chaperone homolog (E. coli) | PC-5p-113875_40 |
| CTDSPL | CTD (carboxy-terminal domain, RNA polymerase II, polypeptide A) small phosphatase-like | PC-5p-113875_40 |
| TNFAIP1 | tumor necrosis factor, alpha-induced protein 1 (endothelial) | PC-5p-582426_7 |
| TNFAIP1 | tumor necrosis factor, alpha-induced protein 1 (endothelial) | gga-miR-1677-3p_L+1 |
| TNFAIP1 | tumor necrosis factor, alpha-induced protein 1 (endothelial) | gga-miR-1782_L+2R-1 |
| TNFAIP1 | tumor necrosis factor, alpha-induced protein 1 (endothelial) | gga-miR-1a-3p_R+1_1ss2GT |
| TNFAIP1 | tumor necrosis factor, alpha-induced protein 1 (endothelial) | gga-miR-33-3p_L+1R+1 |
| TNFAIP1 | tumor necrosis factor, alpha-induced protein 1 (endothelial) | tgu-miR-2970-5p_1ss21GT |
| SQRDL | sulfide quinone reductase-like (yeast) | gga-miR-7468-3p_L+3 |
| SQRDL | sulfide quinone reductase-like (yeast) | tgu-miR-2970-5p_1ss21GT |
| PLDN | pallidin homolog (mouse) | gga-miR-1782_L+2R-1 |
| YTHDF1 | YTH domain family, member 1 | PC-5p-113875_40 |
| SPPL2A | signal peptide peptidase like 2A | PC-5p-582426_7 |
| SPPL2A | signal peptide peptidase like 2A | gga-miR-30a-5p_R+2_1ss13CT |
| SPPL2A | signal peptide peptidase like 2A | gga-miR-30e-5p_R+5 |
| SPPL2A | signal peptide peptidase like 2A | hsa-miR-483-3p_L-1R+2 |
| TRAF7 | TNF receptor-associated factor 7, E3 ubiquitin protein ligase | PC-3p-173245_25 |
| TRAF7 | TNF receptor-associated factor 7, E3 ubiquitin protein ligase | gga-miR-34b-5p_L-1R+1 |
| STK25 | serine/threonine kinase 25 | chi-miR-16b-5p_R+1 |
| STK25 | serine/threonine kinase 25 | gga-miR-16-5p |
| ARFGAP1 | ADP-ribosylation factor GTPase activating protein 1 | PC-5p-71982_65 |
| ARFGAP1 | ADP-ribosylation factor GTPase activating protein 1 | PC-5p-97606_47 |
| ARFGAP1 | ADP-ribosylation factor GTPase activating protein 1 | chi-miR-16b-5p_R+1 |
| ARFGAP1 | ADP-ribosylation factor GTPase activating protein 1 | gga-let-7g-3p_1ss22CT |
| ARFGAP1 | ADP-ribosylation factor GTPase activating protein 1 | gga-miR-16-5p |
| ARFGAP1 | ADP-ribosylation factor GTPase activating protein 1 | gga-miR-1782_L+2R-1 |
| GCLM | glutamate-cysteine ligase, modifier subunit | PC-3p-457530_9 |
| GCLM | glutamate-cysteine ligase, modifier subunit | PC-3p-52432_89 |
| GCLM | glutamate-cysteine ligase, modifier subunit | PC-5p-113875_40 |
| GCLM | glutamate-cysteine ligase, modifier subunit | PC-5p-97606_47 |
| GCLM | glutamate-cysteine ligase, modifier subunit | aca-miR-18a-5p_R-2 |
| GCLM | glutamate-cysteine ligase, modifier subunit | gga-miR-1456-5p_L-1 |
| GCLM | glutamate-cysteine ligase, modifier subunit | gga-miR-1782_L+2R-1 |
| XBP1 | X-box binding protein 1 | aca-miR-363-3p_R+1 |
| XBP1 | X-box binding protein 1 | oan-miR-363-3p_R+1 |
| XBP1 | X-box binding protein 1 | tgu-miR-2970-5p_1ss21GT |
| DCAF12 | DDB1 and CUL4 associated factor 12 | PC-5p-37716_121 |
| DCAF12 | DDB1 and CUL4 associated factor 12 | PC-5p-582426_7 |
| DCAF12 | DDB1 and CUL4 associated factor 12 | chi-miR-16b-5p_R+1 |
| DCAF12 | DDB1 and CUL4 associated factor 12 | gga-miR-16-5p |
| DCAF12 | DDB1 and CUL4 associated factor 12 | gga-miR-1677-3p_L+1 |
| CHRNA4 | cholinergic receptor, nicotinic, alpha 4 | PC-5p-71982_65 |
| CHRNA4 | cholinergic receptor, nicotinic, alpha 4 | gga-miR-1677-3p_L+1 |
| SEPT2 | septin 2 | PC-3p-457530_9 |
| TMEM41B | transmembrane protein 41B | PC-5p-71982_65 |
| TMEM41B | transmembrane protein 41B | PC-5p-97606_47 |
| TMEM41B | transmembrane protein 41B | gga-miR-1a-3p_R+1_1ss2GT |
| TMEM41B | transmembrane protein 41B | gga-miR-30a-5p_R+2_1ss13CT |
| TMEM41B | transmembrane protein 41B | gga-miR-30e-5p_R+5 |
| BCAR3 | breast cancer anti-estrogen resistance 3 | gga-miR-1805-5p_L-1 |
| HDLBP | high density lipoprotein binding protein | PC-5p-113875_40 |
| PAFAH1B1 | platelet-activating factor acetylhydrolase 1b, regulatory subunit 1 (45kDa) | PC-5p-97606_47 |
| PAFAH1B1 | platelet-activating factor acetylhydrolase 1b, regulatory subunit 1 (45kDa) | aca-miR-363-3p_R+1 |
| PAFAH1B1 | platelet-activating factor acetylhydrolase 1b, regulatory subunit 1 (45kDa) | gga-let-7g-3p_1ss22CT |
| PAFAH1B1 | platelet-activating factor acetylhydrolase 1b, regulatory subunit 1 (45kDa) | oan-miR-363-3p_R+1 |
| ALOX5 | arachidonate 5-lipoxygenase | hsa-miR-483-3p_L-1R+2 |
| DR1 | down-regulator of transcription 1, TBP-binding (negative cofactor 2) | PC-5p-71982_65 |
| DR1 | down-regulator of transcription 1, TBP-binding (negative cofactor 2) | gga-let-7g-3p_1ss22CT |
| DR1 | down-regulator of transcription 1, TBP-binding (negative cofactor 2) | gga-miR-1782_L+2R-1 |
| RAP1GAP2 | RAP1 GTPase activating protein 2 | PC-5p-97606_47 |
| BANP | BTG3 associated nuclear protein | PC-5p-233284_18 |
| LOC420419 | mitogen-activated protein kinase kinase kinase 3-like | PC-5p-97606_47 |
| LOC420419 | mitogen-activated protein kinase kinase kinase 3-like | gga-let-7g-3p_1ss22CT |
| RAB7A | RAB7A, member RAS oncogene family | aca-miR-425-3p_L-2R+1_1ss3AT |
| RAB7A | RAB7A, member RAS oncogene family | gga-miR-30a-5p_R+2_1ss13CT |
| RAB7A | RAB7A, member RAS oncogene family | gga-miR-30e-5p_R+5 |
| TMED5 | transmembrane emp24 protein transport domain containing 5 | gga-miR-7468-3p_L+3 |
| TMED5 | transmembrane emp24 protein transport domain containing 5 | tgu-miR-2970-5p_1ss21GT |
| MTF2 | metal response element binding transcription factor 2 | gga-miR-1782_L+2R-1 |
| MTF2 | metal response element binding transcription factor 2 | gga-miR-33-3p_L+1R+1 |
| MGAT4B | mannosyl (alpha-1,3-)-glycoprotein beta-1,4-N-acetylglucosaminyltransferase, isozyme B | PC-3p-52432_89 |
| MGAT4B | mannosyl (alpha-1,3-)-glycoprotein beta-1,4-N-acetylglucosaminyltransferase, isozyme B | aca-miR-18a-5p_R-2 |
| ST5 | suppression of tumorigenicity 5 | gga-miR-1456-5p_L-1 |
| PLS3 | plastin 3 | chi-miR-16b-5p_R+1 |
| PLS3 | plastin 3 | gga-miR-16-5p |
| PLS3 | plastin 3 | hsa-miR-483-3p_L-1R+2 |
| RUVBL1 | RuvB-like 1 (E. coli) | PC-3p-173245_25 |
| RUVBL1 | RuvB-like 1 (E. coli) | PC-3p-457530_9 |
| GFI1 | growth factor independent 1 transcription repressor | gga-miR-1805-5p_L-1 |
| MYD88 | myeloid differentiation primary response gene (88) | PC-5p-71982_65 |
| MYD88 | myeloid differentiation primary response gene (88) | PC-5p-92816_49 |
| MYD88 | myeloid differentiation primary response gene (88) | gga-miR-1677-3p_L+1 |
| MYD88 | myeloid differentiation primary response gene (88) | gga-miR-30a-5p_R+2_1ss13CT |
| MYD88 | myeloid differentiation primary response gene (88) | gga-miR-30e-5p_R+5 |
| LOC416931 | vacuolar protein sorting 29 homolog | gga-miR-34b-5p_L-1R+1 |
| LOC100858792 | rhombotin-1-like | gga-miR-1782_L+2R-1 |
| LOC100858792 | rhombotin-1-like | gga-miR-33-3p_L+1R+1 |
| GDF2 | growth differentiation factor 2 | chi-miR-16b-5p_R+1 |
| GDF2 | growth differentiation factor 2 | gga-let-7g-3p_1ss22CT |
| GDF2 | growth differentiation factor 2 | gga-miR-16-5p |
| KLHL13 | kelch-like 9 (Drosophila) | gga-miR-1782_L+2R-1 |
| AQP8 | aquaporin 8 | gga-miR-34b-5p_L-1R+1 |
| SLC13A5 | solute carrier family 13 (sodium-dependent citrate transporter), member 5 | aca-miR-18a-5p_R-2 |
| SLC13A5 | solute carrier family 13 (sodium-dependent citrate transporter), member 5 | gga-let-7g-3p_1ss22CT |
| RRAS2 | related RAS viral (r-ras) oncogene homolog 2 | PC-3p-457530_9 |
| RRAS2 | related RAS viral (r-ras) oncogene homolog 2 | PC-5p-92816_49 |
| RRAS2 | related RAS viral (r-ras) oncogene homolog 2 | aca-miR-18a-5p_R-2 |
| ABTB1 | ankyrin repeat and BTB (POZ) domain containing 1 | PC-5p-582426_7 |
| ABTB1 | ankyrin repeat and BTB (POZ) domain containing 1 | PC-5p-71982_65 |
| ABTB1 | ankyrin repeat and BTB (POZ) domain containing 1 | aca-miR-363-3p_R+1 |
| ABTB1 | ankyrin repeat and BTB (POZ) domain containing 1 | chi-miR-16b-5p_R+1 |
| ABTB1 | ankyrin repeat and BTB (POZ) domain containing 1 | gga-miR-16-5p |
| ABTB1 | ankyrin repeat and BTB (POZ) domain containing 1 | gga-miR-1782_L+2R-1 |
| ABTB1 | ankyrin repeat and BTB (POZ) domain containing 1 | oan-miR-363-3p_R+1 |
| PRPF6 | PRP6 pre-mRNA processing factor 6 homolog (S. cerevisiae) | PC-5p-97606_47 |
| PRPF6 | PRP6 pre-mRNA processing factor 6 homolog (S. cerevisiae) | chi-miR-16b-5p_R+1 |
| PRPF6 | PRP6 pre-mRNA processing factor 6 homolog (S. cerevisiae) | gga-miR-16-5p |
| MED31 | mediator complex subunit 31 | PC-3p-173245_25 |
| KIAA0753 | KIAA0753 | gga-miR-1782_L+2R-1 |
| COPB1 | coatomer protein complex, subunit beta 1 | gga-miR-1677-3p_L+1 |
| PASK | PAS domain containing serine/threonine kinase | PC-5p-37716_121 |
| PASK | PAS domain containing serine/threonine kinase | gga-miR-7468-3p_L+3 |
| DOCK11 | dedicator of cytokinesis 11 | PC-5p-71982_65 |
| DOCK11 | dedicator of cytokinesis 11 | chi-miR-16b-5p_R+1 |
| DOCK11 | dedicator of cytokinesis 11 | gga-miR-16-5p |
| BTBD1 | BTB (POZ) domain containing 1 | PC-5p-92816_49 |
| BTBD1 | BTB (POZ) domain containing 1 | gga-let-7g-3p_1ss22CT |
| CNOT6 | CCR4-NOT transcription complex, subunit 6 | aca-miR-18a-5p_R-2 |
| CNOT6 | CCR4-NOT transcription complex, subunit 6 | gga-miR-33-3p_L+1R+1 |
| CNOT6 | CCR4-NOT transcription complex, subunit 6 | hsa-miR-483-3p_L-1R+2 |
| GORASP1 | golgi reassembly stacking protein 1, 65kDa | aca-miR-18a-5p_R-2 |
| GORASP1 | golgi reassembly stacking protein 1, 65kDa | chi-miR-16b-5p_R+1 |
| GORASP1 | golgi reassembly stacking protein 1, 65kDa | gga-miR-1329-3p |
| GORASP1 | golgi reassembly stacking protein 1, 65kDa | gga-miR-16-5p |
| GORASP1 | golgi reassembly stacking protein 1, 65kDa | gga-miR-1677-3p_L+1 |
| GORASP1 | golgi reassembly stacking protein 1, 65kDa | gga-miR-30a-5p_R+2_1ss13CT |
| GORASP1 | golgi reassembly stacking protein 1, 65kDa | gga-miR-30e-5p_R+5 |
| MCM2 | minichromosome maintenance complex component 2 | aca-miR-18a-5p_R-2 |
| MCM2 | minichromosome maintenance complex component 2 | chi-miR-16b-5p_R+1 |
| MCM2 | minichromosome maintenance complex component 2 | gga-miR-16-5p |
| UCKL1 | uridine-cytidine kinase 1-like 1 | aca-miR-18a-5p_R-2 |
| DNAJC5 | DnaJ (Hsp40) homolog, subfamily C, member 5 | gga-miR-33-3p_L+1R+1 |
| TPRA1 | transmembrane protein, adipocyte asscociated 1 | PC-5p-71982_65 |
| TPRA1 | transmembrane protein, adipocyte asscociated 1 | PC-5p-97606_47 |
| TPRA1 | transmembrane protein, adipocyte asscociated 1 | tgu-miR-2970-5p_1ss21GT |
| CALCA | calcitonin | gga-miR-1782_L+2R-1 |
| CALCA | calcitonin | gga-miR-7468-3p_L+3 |
| SMTNL2 | smoothelin-like 2 | PC-5p-113875_40 |
| SMTNL2 | smoothelin-like 2 | PC-5p-233284_18 |
| SMTNL2 | smoothelin-like 2 | gga-miR-34b-5p_L-1R+1 |
| WNT8A | wingless-type MMTV integration site family, member 8A | chi-miR-16b-5p_R+1 |
| WNT8A | wingless-type MMTV integration site family, member 8A | gga-miR-16-5p |
| WNT8A | wingless-type MMTV integration site family, member 8A | gga-miR-30a-5p_R+2_1ss13CT |
| WNT8A | wingless-type MMTV integration site family, member 8A | gga-miR-30e-5p_R+5 |
| DCTN5 | dynactin 5 (p25) | gga-miR-1805-5p_L-1 |
| PHF6 | PHD finger protein 6 | PC-5p-92816_49 |
| HPRT1 | hypoxanthine phosphoribosyltransferase 1 | aca-miR-18a-5p_R-2 |
| HPRT1 | hypoxanthine phosphoribosyltransferase 1 | aca-miR-363-3p_R+1 |
| HPRT1 | hypoxanthine phosphoribosyltransferase 1 | gga-miR-30a-5p_R+2_1ss13CT |
| HPRT1 | hypoxanthine phosphoribosyltransferase 1 | gga-miR-30e-5p_R+5 |
| HPRT1 | hypoxanthine phosphoribosyltransferase 1 | oan-miR-363-3p_R+1 |
| ARFRP1 | ADP-ribosylation factor related protein 1 | gga-miR-34b-5p_L-1R+1 |
| MAPK8 | mitogen-activated protein kinase 8 | aca-miR-18a-5p_R-2 |
| MAPK8 | mitogen-activated protein kinase 8 | chi-miR-16b-5p_R+1 |
| MAPK8 | mitogen-activated protein kinase 8 | gga-miR-16-5p |
| ZNF326 | zinc finger protein 326 | gga-miR-33-3p_L+1R+1 |
| ZNF326 | zinc finger protein 326 | tgu-miR-2970-5p_1ss21GT |
| PIK3C2A | phosphoinositide-3-kinase, class 2, alpha polypeptide | PC-5p-97606_47 |
| RTEL1 | regulator of telomere elongation helicase 1 | chi-miR-16b-5p_R+1 |
| RTEL1 | regulator of telomere elongation helicase 1 | gga-miR-16-5p |
| COL6A2 | collagen, type VI, alpha 2 | PC-5p-97606_47 |
| COL6A2 | collagen, type VI, alpha 2 | chi-miR-16b-5p_R+1 |
| COL6A2 | collagen, type VI, alpha 2 | gga-miR-16-5p |
| COL6A2 | collagen, type VI, alpha 2 | gga-miR-34b-5p_L-1R+1 |
| FAM122A | family with sequence similarity 122A | chi-miR-16b-5p_R+1 |
| FAM122A | family with sequence similarity 122A | gga-miR-16-5p |
| MOSPD1 | motile sperm domain containing 1 | PC-5p-92816_49 |
| MOSPD1 | motile sperm domain containing 1 | gga-miR-7468-3p_L+3 |
| HELZ2 | helicase with zinc finger 2, transcriptional coactivator | PC-3p-52432_89 |
| POFUT2 | protein O-fucosyltransferase 2 | PC-5p-97606_47 |
| CDK10 | cyclin-dependent kinase 10 | PC-3p-338480_12 |
| CDK10 | cyclin-dependent kinase 10 | PC-5p-97606_47 |
| MORN4 | MORN repeat containing 4 | PC-5p-37716_121 |
| MORN4 | MORN repeat containing 4 | gga-miR-34b-5p_L-1R+1 |
| MORN4 | MORN repeat containing 4 | hsa-miR-483-3p_L-1R+2 |
| XYLB | xylulokinase homolog (H. influenzae) | gga-miR-1805-5p_L-1 |
| XYLB | xylulokinase homolog (H. influenzae) | gga-miR-34b-5p_L-1R+1 |
| CCBL2 | cysteine conjugate-beta lyase 2 | hsa-miR-483-3p_L-1R+2 |
| PRKAG2 | protein kinase, AMP-activated, gamma 2 non-catalytic subunit | gga-miR-33-3p_L+1R+1 |
| KLHL3 | kelch-like 3 (Drosophila) | PC-5p-71982_65 |
| KLHL3 | kelch-like 3 (Drosophila) | gga-miR-1456-5p_L-1 |
| BCL2L1 | BCL2-like 1 | hsa-miR-483-3p_L-1R+2 |
| SLC41A3 | solute carrier family 41, member 3 | PC-3p-52432_89 |
| ERCC6 | excision repair cross-complementing rodent repair deficiency, complementation group 6 | PC-5p-71982_65 |
| HPS5 | Hermansky-Pudlak syndrome 5 | chi-miR-16b-5p_R+1 |
| HPS5 | Hermansky-Pudlak syndrome 5 | gga-miR-16-5p |
| MYLK2 | myosin light chain kinase 2 | PC-5p-113875_40 |
| GINS1 | GINS complex subunit 1 (Psf1 homolog) | PC-5p-113875_40 |
| GINS1 | GINS complex subunit 1 (Psf1 homolog) | aca-miR-18a-5p_R-2 |
| GALNS | galactosamine (N-acetyl)-6-sulfate sulfatase | gga-miR-30a-5p_R+2_1ss13CT |
| GALNS | galactosamine (N-acetyl)-6-sulfate sulfatase | gga-miR-30e-5p_R+5 |
| ACTR3B | ARP3 actin-related protein 3 homolog B (yeast) | PC-3p-173245_25 |
| ACTR3B | ARP3 actin-related protein 3 homolog B (yeast) | PC-5p-582426_7 |
| KDM8 | lysine (K)-specific demethylase 8 | PC-5p-113875_40 |
| IL21R | interleukin 21 receptor | gga-miR-34b-5p_L-1R+1 |
| LOC424523 | epithelial chloride channel protein-like | tgu-miR-2970-5p_1ss21GT |
| MIF | macrophage migration inhibitory factor (glycosylation-inhibiting factor) | chi-miR-16b-5p_R+1 |
| MIF | macrophage migration inhibitory factor (glycosylation-inhibiting factor) | gga-miR-16-5p |
| IL2RA | interleukin 2 receptor, alpha | PC-5p-97606_47 |
| K123 | K123 protein | PC-3p-338480_12 |
| K123 | K123 protein | chi-miR-16b-5p_R+1 |
| K123 | K123 protein | gga-miR-16-5p |
| GSTT1 | glutathione S-transferase theta 1 | gga-miR-1a-3p_R+1_1ss2GT |
| TMEM43 | transmembrane protein 43 | chi-miR-16b-5p_R+1 |
| TMEM43 | transmembrane protein 43 | gga-miR-16-5p |
| TMEM43 | transmembrane protein 43 | hsa-miR-483-3p_L-1R+2 |
| DDT | D-dopachrome tautomerase | PC-5p-113875_40 |
| DDT | D-dopachrome tautomerase | aca-miR-18a-5p_R-2 |
| DDT | D-dopachrome tautomerase | chi-miR-16b-5p_R+1 |
| DDT | D-dopachrome tautomerase | gga-miR-16-5p |
| XPC | xeroderma pigmentosum, complementation group C | gga-let-7g-3p_1ss22CT |
| XPC | xeroderma pigmentosum, complementation group C | gga-miR-34b-5p_L-1R+1 |
| PCBD2 | pterin-4 alpha-carbinolamine dehydratase/dimerization cofactor of hepatocyte nuclear factor 1 alpha (TCF1) 2 | gga-miR-7468-3p_L+3 |
| TBX6 | T-box 6 | PC-5p-92816_49 |
| TBX6 | T-box 6 | hsa-miR-483-3p_L-1R+2 |
| PTPRJ | protein tyrosine phosphatase, receptor type, J | PC-5p-582426_7 |
| CRKL | v-crk sarcoma virus CT10 oncogene homolog-like | chi-miR-16b-5p_R+1 |
| CRKL | v-crk sarcoma virus CT10 oncogene homolog-like | gga-miR-16-5p |
| CRKL | v-crk sarcoma virus CT10 oncogene homolog-like | gga-miR-1677-3p_L+1 |
| CRKL | v-crk sarcoma virus CT10 oncogene homolog-like | gga-miR-34b-5p_L-1R+1 |
| OSBPL5 | oxysterol binding protein-like 5 | PC-5p-233284_18 |
| OSBPL5 | oxysterol binding protein-like 5 | aca-miR-363-3p_R+1 |
| OSBPL5 | oxysterol binding protein-like 5 | oan-miR-363-3p_R+1 |
| DDX46 | DEAD (Asp-Glu-Ala-Asp) box polypeptide 46 | gga-miR-30a-5p_R+2_1ss13CT |
| DDX46 | DEAD (Asp-Glu-Ala-Asp) box polypeptide 46 | gga-miR-30e-5p_R+5 |
| HTATSF1 | HIV-1 Tat specific factor 1 | gga-miR-30a-5p_R+2_1ss13CT |
| HTATSF1 | HIV-1 Tat specific factor 1 | gga-miR-30e-5p_R+5 |
| CAMLG | calcium modulating ligand | PC-5p-97606_47 |
| SEC24A | SEC24 family, member A (S. cerevisiae) | aca-miR-18a-5p_R-2 |
| PDPK1 | 3-phosphoinositide dependent protein kinase-1 | PC-3p-338480_12 |
| PDPK1 | 3-phosphoinositide dependent protein kinase-1 | PC-5p-92816_49 |
| PDPK1 | 3-phosphoinositide dependent protein kinase-1 | chi-miR-16b-5p_R+1 |
| PDPK1 | 3-phosphoinositide dependent protein kinase-1 | gga-miR-16-5p |
| PDPK1 | 3-phosphoinositide dependent protein kinase-1 | tgu-miR-2970-5p_1ss21GT |
| ABHD17C | family with sequence similarity 108, member C1 | PC-3p-338480_12 |
| ABHD17C | family with sequence similarity 108, member C1 | PC-5p-233284_18 |
| ABHD17C | family with sequence similarity 108, member C1 | PC-5p-97606_47 |
| ABHD17C | family with sequence similarity 108, member C1 | gga-let-7g-3p_1ss22CT |
| SLC6A6 | solute carrier family 6 (neurotransmitter transporter, taurine), member 6 | PC-5p-233284_18 |
| CARS | cysteinyl-tRNA synthetase | gga-miR-30a-5p_R+2_1ss13CT |
| CARS | cysteinyl-tRNA synthetase | gga-miR-30e-5p_R+5 |
| UBE2I | ubiquitin-conjugating enzyme E2I | gga-miR-30a-5p_R+2_1ss13CT |
| UBE2I | ubiquitin-conjugating enzyme E2I | gga-miR-30e-5p_R+5 |
| PANK1 | pantothenate kinase 1 | PC-5p-582426_7 |
| PANK1 | pantothenate kinase 1 | chi-miR-16b-5p_R+1 |
| PANK1 | pantothenate kinase 1 | gga-miR-16-5p |
| SRPRB | signal recognition particle receptor, B subunit | PC-5p-92816_49 |
| SRPRB | signal recognition particle receptor, B subunit | PC-5p-97606_47 |
| SRPRB | signal recognition particle receptor, B subunit | gga-miR-34b-5p_L-1R+1 |
| C12H3ORF19 | chromosome 12 open reading frame, human C3orf19 | PC-5p-71982_65 |
| C12H3ORF19 | chromosome 12 open reading frame, human C3orf19 | PC-5p-92816_49 |
| TTLL9 | tubulin tyrosine ligase-like family, member 9 | PC-5p-97606_47 |
| TTLL9 | tubulin tyrosine ligase-like family, member 9 | chi-miR-16b-5p_R+1 |
| TTLL9 | tubulin tyrosine ligase-like family, member 9 | gga-miR-16-5p |
| PPP2CA | protein phosphatase 2, catalytic subunit, alpha isozyme | gga-miR-1a-3p_R+1_1ss2GT |
| PPP2CA | protein phosphatase 2, catalytic subunit, alpha isozyme | gga-miR-33-3p_L+1R+1 |
| PPP2CA | protein phosphatase 2, catalytic subunit, alpha isozyme | tgu-miR-2970-5p_1ss21GT |
| ATP12A | ATPase, H+/K+ transporting, nongastric, alpha polypeptide | aca-miR-363-3p_R+1 |
| ATP12A | ATPase, H+/K+ transporting, nongastric, alpha polypeptide | oan-miR-363-3p_R+1 |
| FAH | fumarylacetoacetate hydrolase (fumarylacetoacetase) | hsa-miR-483-3p_L-1R+2 |
| RPP30 | ribonuclease P/MRP 30kDa subunit | hsa-miR-483-3p_L-1R+2 |
| LOC422249 | transmembrane 9 superfamily member 2-like | PC-5p-71982_65 |
| LOC422249 | transmembrane 9 superfamily member 2-like | gga-miR-30a-5p_R+2_1ss13CT |
| LOC422249 | transmembrane 9 superfamily member 2-like | gga-miR-30e-5p_R+5 |
| ANKRD1 | ankyrin repeat domain 1 (cardiac muscle) | PC-5p-97606_47 |
| ANKRD1 | ankyrin repeat domain 1 (cardiac muscle) | chi-miR-16b-5p_R+1 |
| ANKRD1 | ankyrin repeat domain 1 (cardiac muscle) | gga-let-7g-3p_1ss22CT |
| ANKRD1 | ankyrin repeat domain 1 (cardiac muscle) | gga-miR-16-5p |
| ANKRD1 | ankyrin repeat domain 1 (cardiac muscle) | gga-miR-1677-3p_L+1 |
| NDE1 | nudE nuclear distribution E homolog 1 (A. nidulans) | PC-5p-97606_47 |
| NDE1 | nudE nuclear distribution E homolog 1 (A. nidulans) | gga-miR-34b-5p_L-1R+1 |
| VDAC1 | voltage-dependent anion channel 1 | PC-3p-173245_25 |
| VDAC1 | voltage-dependent anion channel 1 | gga-miR-1782_L+2R-1 |
| C13H5ORF15 | chromosome 13 open reading frame, human C5orf15 | gga-let-7g-3p_1ss22CT |
| KIAA1024 | KIAA1024 | aca-miR-363-3p_R+1 |
| KIAA1024 | KIAA1024 | oan-miR-363-3p_R+1 |
| HCK | hemopoietic cell kinase | PC-5p-97606_47 |
| HCK | hemopoietic cell kinase | gga-miR-34b-5p_L-1R+1 |
| CRTAM | cytotoxic and regulatory T cell molecule | PC-5p-71982_65 |
| CRTAM | cytotoxic and regulatory T cell molecule | gga-miR-34b-5p_L-1R+1 |
| TSSC4 | tumor suppressing subtransferable candidate 4 | PC-3p-173245_25 |
| TSSC4 | tumor suppressing subtransferable candidate 4 | PC-5p-582426_7 |
| FOPNL | FGFR1OP N-terminal like | PC-3p-457530_9 |
| FOPNL | FGFR1OP N-terminal like | chi-miR-16b-5p_R+1 |
| FOPNL | FGFR1OP N-terminal like | gga-miR-16-5p |
| C9H21ORF2 | chromosome 9 open reading frame, human C21orf2 | gga-miR-34b-5p_L-1R+1 |
| TH | tyrosine hydroxylase | gga-miR-1677-3p_L+1 |
| INS | insulin | aca-miR-18a-5p_R-2 |
| SEMA3A | sema domain, immunoglobulin domain (Ig), short basic domain, secreted, (semaphorin) 3A | gga-miR-30a-5p_R+2_1ss13CT |
| SEMA3A | sema domain, immunoglobulin domain (Ig), short basic domain, secreted, (semaphorin) 3A | gga-miR-30e-5p_R+5 |
| SEMA3D | sema domain, immunoglobulin domain (Ig), short basic domain, secreted, (semaphorin) 3D | gga-let-7g-3p_1ss22CT |
| SEMA3D | sema domain, immunoglobulin domain (Ig), short basic domain, secreted, (semaphorin) 3D | tgu-miR-2970-5p_1ss21GT |
| DNAJB11 | DnaJ (Hsp40) homolog, subfamily B, member 11 | PC-5p-113875_40 |
| DNAJB11 | DnaJ (Hsp40) homolog, subfamily B, member 11 | chi-miR-16b-5p_R+1 |
| DNAJB11 | DnaJ (Hsp40) homolog, subfamily B, member 11 | gga-miR-16-5p |
| GGT1 | gamma-glutamyltransferase 1 | PC-3p-52432_89 |
| TBCCD1 | TBCC domain containing 1 | chi-miR-16b-5p_R+1 |
| TBCCD1 | TBCC domain containing 1 | gga-miR-16-5p |
| SETD5 | SET domain containing 5 | PC-5p-37716_121 |
| TNNT3 | troponin T type 3 (skeletal, fast) | chi-miR-16b-5p_R+1 |
| TNNT3 | troponin T type 3 (skeletal, fast) | gga-miR-16-5p |
| VIPR2 | vasoactive intestinal peptide receptor 2 | gga-miR-1a-3p_R+1_1ss2GT |
| GRM3 | glutamate receptor, metabotropic 3 | PC-5p-97606_47 |
| ZMYND11 | zinc finger, MYND-type containing 11 | PC-5p-71982_65 |
| KIF3B | kinesin family member 3B | PC-5p-71982_65 |
| KIF3B | kinesin family member 3B | gga-miR-1782_L+2R-1 |
| KIF3B | kinesin family member 3B | gga-miR-30a-5p_R+2_1ss13CT |
| KIF3B | kinesin family member 3B | gga-miR-30e-5p_R+5 |
| CTSD | cathepsin D | PC-5p-97606_47 |
| BCCIP | BRCA2 and CDKN1A interacting protein | chi-miR-16b-5p_R+1 |
| BCCIP | BRCA2 and CDKN1A interacting protein | gga-let-7g-3p_1ss22CT |
| BCCIP | BRCA2 and CDKN1A interacting protein | gga-miR-16-5p |
| BCCIP | BRCA2 and CDKN1A interacting protein | tgu-miR-2970-5p_1ss21GT |
| PDE6C | phosphodiesterase 6C, cGMP-specific, cone, alpha prime | PC-5p-233284_18 |
| PDE6C | phosphodiesterase 6C, cGMP-specific, cone, alpha prime | aca-miR-18a-5p_R-2 |
| PDE6C | phosphodiesterase 6C, cGMP-specific, cone, alpha prime | gga-miR-1a-3p_R+1_1ss2GT |
| PDE6C | phosphodiesterase 6C, cGMP-specific, cone, alpha prime | gga-miR-33-3p_L+1R+1 |
| UPB1 | ureidopropionase, beta | gga-miR-30a-5p_R+2_1ss13CT |
| UPB1 | ureidopropionase, beta | gga-miR-30e-5p_R+5 |
| ABCC1 | ATP-binding cassette, sub-family C (CFTR/MRP), member 1 | PC-3p-457530_9 |
| ABCC1 | ATP-binding cassette, sub-family C (CFTR/MRP), member 1 | chi-miR-16b-5p_R+1 |
| ABCC1 | ATP-binding cassette, sub-family C (CFTR/MRP), member 1 | gga-let-7g-3p_1ss22CT |
| ABCC1 | ATP-binding cassette, sub-family C (CFTR/MRP), member 1 | gga-miR-16-5p |
| ABCC1 | ATP-binding cassette, sub-family C (CFTR/MRP), member 1 | gga-miR-1782_L+2R-1 |
| ABCC1 | ATP-binding cassette, sub-family C (CFTR/MRP), member 1 | gga-miR-33-3p_L+1R+1 |
| MAP3K13 | mitogen-activated protein kinase kinase kinase 13 | gga-miR-7468-3p_L+3 |
| MAPRE1 | microtubule-associated protein, RP/EB family, member 1 | PC-3p-457530_9 |
| FANCI | Fanconi anemia, complementation group I | PC-5p-97606_47 |
| FANCI | Fanconi anemia, complementation group I | aca-miR-18a-5p_R-2 |
| FANCI | Fanconi anemia, complementation group I | chi-miR-16b-5p_R+1 |
| FANCI | Fanconi anemia, complementation group I | gga-miR-16-5p |
| EHHADH | enoyl-CoA, hydratase/3-hydroxyacyl CoA dehydrogenase | PC-5p-71982_65 |
| BRSK2 | BR serine/threonine kinase 2 | gga-miR-1782_L+2R-1 |
| BRSK2 | BR serine/threonine kinase 2 | tgu-miR-2970-5p_1ss21GT |
| TOLLIP | toll interacting protein | aca-miR-363-3p_R+1 |
| TOLLIP | toll interacting protein | chi-miR-16b-5p_R+1 |
| TOLLIP | toll interacting protein | gga-miR-16-5p |
| TOLLIP | toll interacting protein | gga-miR-1805-5p_L-1 |
| TOLLIP | toll interacting protein | oan-miR-363-3p_R+1 |
| CCDC61 | coiled-coil domain containing 61 | PC-5p-37716_121 |
| OAF | OAF homolog (Drosophila) | PC-3p-338480_12 |
| LOC395381 | ovomucin, alpha subunit | PC-5p-113875_40 |
| IDI1 | isopentenyl-diphosphate delta isomerase 1 | PC-5p-97606_47 |
| TAF3 | TAF3 RNA polymerase II, TATA box binding protein (TBP)-associated factor, 140kDa | PC-3p-457530_9 |
| TAF3 | TAF3 RNA polymerase II, TATA box binding protein (TBP)-associated factor, 140kDa | PC-5p-97606_47 |
| TAF3 | TAF3 RNA polymerase II, TATA box binding protein (TBP)-associated factor, 140kDa | gga-miR-1782_L+2R-1 |
| YWHAH | tyrosine 3-monooxygenase/tryptophan 5-monooxygenase activation protein, eta polypeptide | chi-miR-16b-5p_R+1 |
| YWHAH | tyrosine 3-monooxygenase/tryptophan 5-monooxygenase activation protein, eta polypeptide | gga-miR-16-5p |
| ATP5C1 | ATP synthase, H+ transporting, mitochondrial F1 complex, gamma polypeptide 1 | gga-let-7g-3p_1ss22CT |
| ATP5C1 | ATP synthase, H+ transporting, mitochondrial F1 complex, gamma polypeptide 1 | gga-miR-1a-3p_R+1_1ss2GT |
| BPI | bactericidal/permeability-increasing protein | gga-miR-7468-3p_L+3 |
| DEPDC5 | DEP domain containing 5 | PC-5p-71982_65 |
| CBL | Cbl proto-oncogene, E3 ubiquitin protein ligase | chi-miR-16b-5p_R+1 |
| CBL | Cbl proto-oncogene, E3 ubiquitin protein ligase | gga-miR-16-5p |
| KIN | KIN, antigenic determinant of recA protein homolog (mouse) | PC-3p-173245_25 |
| LOC419755 | NLR family member X1-like | PC-3p-52432_89 |
| ARL6IP1 | ADP-ribosylation factor-like 6 interacting protein 1 | PC-5p-582426_7 |
| PLOD2 | procollagen-lysine, 2-oxoglutarate 5-dioxygenase 2 | PC-5p-97606_47 |
| RPRD1B | regulation of nuclear pre-mRNA domain containing 1B | PC-3p-338480_12 |
| RPRD1B | regulation of nuclear pre-mRNA domain containing 1B | PC-5p-113875_40 |
| RPRD1B | regulation of nuclear pre-mRNA domain containing 1B | PC-5p-37716_121 |
| KIF11 | kinesin family member 11 | PC-3p-457530_9 |
| AP2A2 | adaptor-related protein complex 2, alpha 2 subunit | PC-5p-113875_40 |
| AP2A2 | adaptor-related protein complex 2, alpha 2 subunit | PC-5p-71982_65 |
| IL13 | interleukin 13 | PC-5p-113875_40 |
| IL13 | interleukin 13 | gga-miR-1677-3p_L+1 |
| TSPAN6 | tetraspanin 6 | chi-miR-16b-5p_R+1 |
| TSPAN6 | tetraspanin 6 | gga-miR-16-5p |
| POU2AF1 | POU class 2 associating factor 1 | PC-5p-97606_47 |
| TTC36 | tetratricopeptide repeat domain 36 | hsa-miR-483-3p_L-1R+2 |
| CTNNBL1 | catenin, beta like 1 | PC-5p-97606_47 |
| CTNNBL1 | catenin, beta like 1 | chi-miR-16b-5p_R+1 |
| CTNNBL1 | catenin, beta like 1 | gga-miR-16-5p |
| CHID1 | chitinase domain containing 1 | PC-3p-52432_89 |
| NCBP2 | nuclear cap binding protein subunit 2, 20kDa | chi-miR-16b-5p_R+1 |
| NCBP2 | nuclear cap binding protein subunit 2, 20kDa | gga-miR-16-5p |
| NCBP2 | nuclear cap binding protein subunit 2, 20kDa | gga-miR-30a-5p_R+2_1ss13CT |
| NCBP2 | nuclear cap binding protein subunit 2, 20kDa | gga-miR-30e-5p_R+5 |
| KIF3A | kinesin family member 3A | PC-5p-113875_40 |
| KIF3A | kinesin family member 3A | PC-5p-233284_18 |
| KIF3A | kinesin family member 3A | gga-let-7g-3p_1ss22CT |
| COQ7 | coenzyme Q7 homolog, ubiquinone (yeast) | gga-let-7g-3p_1ss22CT |
| IDE | insulin-degrading enzyme | PC-5p-582426_7 |
| PISD | phosphatidylserine decarboxylase | chi-miR-16b-5p_R+1 |
| PISD | phosphatidylserine decarboxylase | gga-miR-16-5p |
| RASSF7 | Ras association (RalGDS/AF-6) domain family (N-terminal) member 7 | PC-5p-97606_47 |
| CTSA | cathepsin A | gga-miR-34b-5p_L-1R+1 |
| KLHL4 | kelch-like 4 (Drosophila) | gga-let-7g-3p_1ss22CT |
| MARCH5 | membrane-associated ring finger (C3HC4) 5 | chi-miR-16b-5p_R+1 |
| MARCH5 | membrane-associated ring finger (C3HC4) 5 | gga-let-7g-3p_1ss22CT |
| MARCH5 | membrane-associated ring finger (C3HC4) 5 | gga-miR-16-5p |
| HRAS | v-Ha-ras Harvey rat sarcoma viral oncogene homolog | gga-let-7g-3p_1ss22CT |
| FAM174B | family with sequence similarity 174, member B | PC-5p-113875_40 |
| ITIH5 | inter-alpha-trypsin inhibitor heavy chain family, member 5 | PC-5p-97606_47 |
| RNH1 | ribonuclease/angiogenin inhibitor 1 | gga-miR-34b-5p_L-1R+1 |
| CHM | choroideremia (Rab escort protein 1) | gga-miR-1677-3p_L+1 |
| CHM | choroideremia (Rab escort protein 1) | gga-miR-1782_L+2R-1 |
| CHKA | choline kinase alpha | aca-miR-363-3p_R+1 |
| CHKA | choline kinase alpha | gga-miR-1782_L+2R-1 |
| CHKA | choline kinase alpha | oan-miR-363-3p_R+1 |
| RPTOR | regulatory associated protein of MTOR, complex 1 | gga-miR-1782_L+2R-1 |
| PFKP | phosphofructokinase, platelet | chi-miR-16b-5p_R+1 |
| PFKP | phosphofructokinase, platelet | gga-miR-16-5p |
| PFKP | phosphofructokinase, platelet | gga-miR-34b-5p_L-1R+1 |
| PFKP | phosphofructokinase, platelet | hsa-miR-483-3p_L-1R+2 |
| LOC395991 | crescent | PC-5p-37716_121 |
| LOC395991 | crescent | PC-5p-71982_65 |
| BDH1 | 3-hydroxybutyrate dehydrogenase, type 1 | PC-3p-457530_9 |
| BDH1 | 3-hydroxybutyrate dehydrogenase, type 1 | gga-miR-30a-5p_R+2_1ss13CT |
| BDH1 | 3-hydroxybutyrate dehydrogenase, type 1 | gga-miR-30e-5p_R+5 |
| ARIH2 | ariadne homolog 2 (Drosophila) | PC-3p-52432_89 |
| RNF185 | ring finger protein 185 | PC-5p-113875_40 |
| RNF185 | ring finger protein 185 | PC-5p-71982_65 |
| RNF185 | ring finger protein 185 | gga-miR-1677-3p_L+1 |
| SDR42E2 | short chain dehydrogenase/reductase family 42E, member 2 | PC-5p-71982_65 |
| BLNK | B-cell linker | PC-5p-97606_47 |
| APOOL | apolipoprotein O-like | gga-miR-1a-3p_R+1_1ss2GT |
| PPP1R2 | protein phosphatase 1, regulatory (inhibitor) subunit 2 | gga-miR-1782_L+2R-1 |
| PPP1R2 | protein phosphatase 1, regulatory (inhibitor) subunit 2 | gga-miR-30a-5p_R+2_1ss13CT |
| PPP1R2 | protein phosphatase 1, regulatory (inhibitor) subunit 2 | gga-miR-30e-5p_R+5 |
| POLR3E | polymerase (RNA) III (DNA directed) polypeptide E (80kD) | gga-miR-30a-5p_R+2_1ss13CT |
| POLR3E | polymerase (RNA) III (DNA directed) polypeptide E (80kD) | gga-miR-30e-5p_R+5 |
| SLC26A11 | solute carrier family 26, member 11 | gga-miR-34b-5p_L-1R+1 |
| LOC100858827 | cAMP-dependent protein kinase type II-alpha regulatory subunit-like | PC-5p-582426_7 |
| LOC100858827 | cAMP-dependent protein kinase type II-alpha regulatory subunit-like | PC-5p-97606_47 |
| LOC100858827 | cAMP-dependent protein kinase type II-alpha regulatory subunit-like | chi-miR-16b-5p_R+1 |
| LOC100858827 | cAMP-dependent protein kinase type II-alpha regulatory subunit-like | gga-miR-16-5p |
| PITRM1 | pitrilysin metallopeptidase 1 | chi-miR-16b-5p_R+1 |
| PITRM1 | pitrilysin metallopeptidase 1 | gga-miR-16-5p |
| ACAP2 | ArfGAP with coiled-coil, ankyrin repeat and PH domains 2 | PC-5p-582426_7 |
| ACAP2 | ArfGAP with coiled-coil, ankyrin repeat and PH domains 2 | PC-5p-97606_47 |
| ACAP2 | ArfGAP with coiled-coil, ankyrin repeat and PH domains 2 | gga-let-7g-3p_1ss22CT |
| METTL9 | methyltransferase like 9 | PC-5p-113875_40 |
| BUD13 | BUD13 homolog (S. cerevisiae) | gga-miR-34b-5p_L-1R+1 |
| PXK | PX domain containing serine/threonine kinase | PC-3p-457530_9 |
| PXK | PX domain containing serine/threonine kinase | PC-5p-97606_47 |
| LRRC28 | leucine rich repeat containing 28 | gga-miR-33-3p_L+1R+1 |
| ACADS | acyl-CoA dehydrogenase, C-2 to C-3 short chain | PC-5p-97606_47 |
| DUSP12 | dual specificity phosphatase 12 | gga-miR-1a-3p_R+1_1ss2GT |
| CPN1 | carboxypeptidase N, polypeptide 1 | PC-5p-71982_65 |
| CABP1 | calcium binding protein 1 | aca-miR-18a-5p_R-2 |
| CABP1 | calcium binding protein 1 | gga-miR-1805-5p_L-1 |
| CABP1 | calcium binding protein 1 | gga-miR-30a-5p_R+2_1ss13CT |
| CABP1 | calcium binding protein 1 | gga-miR-30e-5p_R+5 |
| POP5 | processing of precursor 5, ribonuclease P/MRP subunit (S. cerevisiae) | gga-miR-7468-3p_L+3 |
| ALDH1A3 | aldehyde dehydrogenase 1 family, member A3 | gga-let-7g-3p_1ss22CT |
| ALDH1A3 | aldehyde dehydrogenase 1 family, member A3 | gga-miR-1782_L+2R-1 |
| RAC1 | ras-related C3 botulinum toxin substrate 1 (rho family, small GTP binding protein Rac1) | gga-miR-1a-3p_R+1_1ss2GT |
| NRP1 | neuropilin 1 | PC-5p-113875_40 |
| NRP1 | neuropilin 1 | gga-let-7g-3p_1ss22CT |
| NRP1 | neuropilin 1 | gga-miR-30a-5p_R+2_1ss13CT |
| NRP1 | neuropilin 1 | gga-miR-30e-5p_R+5 |
| RNF10 | ring finger protein 10 | PC-5p-97606_47 |
| RNF10 | ring finger protein 10 | chi-miR-16b-5p_R+1 |
| RNF10 | ring finger protein 10 | gga-miR-16-5p |
| RNF10 | ring finger protein 10 | gga-miR-30a-5p_R+2_1ss13CT |
| RNF10 | ring finger protein 10 | gga-miR-30e-5p_R+5 |
| BRWD3 | bromodomain and WD repeat domain containing 3 | aca-miR-363-3p_R+1 |
| BRWD3 | bromodomain and WD repeat domain containing 3 | oan-miR-363-3p_R+1 |
| RMI2 | RMI2, RecQ mediated genome instability 2, homolog (S. cerevisiae) | PC-5p-97606_47 |
| RMI2 | RMI2, RecQ mediated genome instability 2, homolog (S. cerevisiae) | chi-miR-16b-5p_R+1 |
| RMI2 | RMI2, RecQ mediated genome instability 2, homolog (S. cerevisiae) | gga-miR-16-5p |
| EPC1 | enhancer of polycomb homolog 1 (Drosophila) | gga-let-7g-3p_1ss22CT |
| PTPRG | protein tyrosine phosphatase, receptor type, G | PC-5p-71982_65 |
| PTPRG | protein tyrosine phosphatase, receptor type, G | PC-5p-92816_49 |
| PGS1 | phosphatidylglycerophosphate synthase 1 | tgu-miR-2970-5p_1ss21GT |
| ERGIC2 | ERGIC and golgi 2 | aca-miR-363-3p_R+1 |
| ERGIC2 | ERGIC and golgi 2 | gga-miR-33-3p_L+1R+1 |
| ERGIC2 | ERGIC and golgi 2 | hsa-miR-483-3p_L-1R+2 |
| ERGIC2 | ERGIC and golgi 2 | oan-miR-363-3p_R+1 |
| ERGIC2 | ERGIC and golgi 2 | tgu-miR-2970-5p_1ss21GT |
| UTS2D | urotensin 2 domain containing | aca-miR-363-3p_R+1 |
| UTS2D | urotensin 2 domain containing | oan-miR-363-3p_R+1 |
| P4HB | prolyl 4-hydroxylase, beta polypeptide | PC-5p-97606_47 |
| ANKDD1A | ankyrin repeat and death domain containing 1A | chi-miR-16b-5p_R+1 |
| ANKDD1A | ankyrin repeat and death domain containing 1A | gga-miR-16-5p |
| ZEB1 | zinc finger E-box binding homeobox 1 | PC-5p-233284_18 |
| CADPS | Ca++-dependent secretion activator | PC-5p-113875_40 |
| GABRE | gamma-aminobutyric acid (GABA) A receptor, epsilon | gga-miR-33-3p_L+1R+1 |
| RNF166 | ring finger protein 166 | PC-3p-338480_12 |
| RNF166 | ring finger protein 166 | PC-3p-52432_89 |
| RNF166 | ring finger protein 166 | gga-miR-1456-5p_L-1 |
| RNF166 | ring finger protein 166 | gga-miR-34b-5p_L-1R+1 |
| HSPA4 | heat shock 70kDa protein 4 | gga-miR-1677-3p_L+1 |
| GCN1L1 | GCN1 general control of amino-acid synthesis 1-like 1 (yeast) | chi-miR-16b-5p_R+1 |
| GCN1L1 | GCN1 general control of amino-acid synthesis 1-like 1 (yeast) | gga-miR-16-5p |
| RNF214 | ring finger protein 214 | hsa-miR-483-3p_L-1R+2 |
| SYNPR | synaptoporin | PC-5p-97606_47 |
| SYNPR | synaptoporin | aca-miR-18a-5p_R-2 |
| SYNPR | synaptoporin | aca-miR-363-3p_R+1 |
| SYNPR | synaptoporin | gga-miR-30a-5p_R+2_1ss13CT |
| SYNPR | synaptoporin | gga-miR-30e-5p_R+5 |
| SYNPR | synaptoporin | oan-miR-363-3p_R+1 |
| CLPX | ClpX caseinolytic peptidase X homolog (E. coli) | PC-3p-338480_12 |
| CLPX | ClpX caseinolytic peptidase X homolog (E. coli) | PC-5p-113875_40 |
| CLPX | ClpX caseinolytic peptidase X homolog (E. coli) | PC-5p-97606_47 |
| CLPX | ClpX caseinolytic peptidase X homolog (E. coli) | gga-miR-1456-5p_L-1 |
| CLPX | ClpX caseinolytic peptidase X homolog (E. coli) | gga-miR-7468-3p_L+3 |
| THOC7 | THO complex 7 homolog (Drosophila) | gga-let-7g-3p_1ss22CT |
| THOC7 | THO complex 7 homolog (Drosophila) | gga-miR-30a-5p_R+2_1ss13CT |
| THOC7 | THO complex 7 homolog (Drosophila) | gga-miR-30e-5p_R+5 |
| NME1 | non-metastatic cells 1, protein (NM23A) expressed in | gga-miR-30a-5p_R+2_1ss13CT |
| NME1 | non-metastatic cells 1, protein (NM23A) expressed in | gga-miR-30e-5p_R+5 |
| RAB35 | RAB35, member RAS oncogene family | PC-3p-52432_89 |
| RAB35 | RAB35, member RAS oncogene family | aca-miR-18a-5p_R-2 |
| RAB35 | RAB35, member RAS oncogene family | hsa-miR-483-3p_L-1R+2 |
| SVIL | supervillin | PC-5p-113875_40 |
| SVIL | supervillin | chi-miR-16b-5p_R+1 |
| SVIL | supervillin | gga-miR-16-5p |
| LPP | LIM domain containing preferred translocation partner in lipoma | PC-3p-52432_89 |
| LPP | LIM domain containing preferred translocation partner in lipoma | PC-5p-71982_65 |
| FGF1 | fibroblast growth factor 1 (acidic) | PC-5p-71982_65 |
| SPAG9 | sperm associated antigen 9 | aca-miR-18a-5p_R-2 |
| MAP3K8 | mitogen-activated protein kinase kinase kinase 8 | PC-5p-233284_18 |
| ZDHHC5 | zinc finger, DHHC-type containing 5 | aca-miR-363-3p_R+1 |
| ZDHHC5 | zinc finger, DHHC-type containing 5 | oan-miR-363-3p_R+1 |
| TOB1 | transducer of ERBB2, 1 | gga-miR-1805-5p_L-1 |
| TOB1 | transducer of ERBB2, 1 | gga-miR-1a-3p_R+1_1ss2GT |
| TOB1 | transducer of ERBB2, 1 | tgu-miR-2970-5p_1ss21GT |
| PRKAB1 | protein kinase, AMP-activated, beta 1 non-catalytic subunit | PC-5p-113875_40 |
| PRKAB1 | protein kinase, AMP-activated, beta 1 non-catalytic subunit | PC-5p-582426_7 |
| PRKAB1 | protein kinase, AMP-activated, beta 1 non-catalytic subunit | aca-miR-363-3p_R+1 |
| PRKAB1 | protein kinase, AMP-activated, beta 1 non-catalytic subunit | oan-miR-363-3p_R+1 |
| PRKAB1 | protein kinase, AMP-activated, beta 1 non-catalytic subunit | tgu-miR-2970-5p_1ss21GT |
| CDA | cytidine deaminase | PC-5p-113875_40 |
| HSPB8 | heat shock 22kDa protein 8 | PC-5p-37716_121 |
| HSPB8 | heat shock 22kDa protein 8 | gga-miR-1456-3p_R+1 |
| ANKRD40 | ankyrin repeat domain 40 | PC-3p-173245_25 |
| ANKRD40 | ankyrin repeat domain 40 | PC-5p-37716_121 |
| ANKRD40 | ankyrin repeat domain 40 | chi-miR-16b-5p_R+1 |
| ANKRD40 | ankyrin repeat domain 40 | gga-miR-16-5p |
| SUDS3 | suppressor of defective silencing 3 homolog (S. cerevisiae) | PC-5p-97606_47 |
| ABCC2 | ATP-binding cassette, sub-family C (CFTR/MRP), member 2 | PC-3p-173245_25 |
| ABCC2 | ATP-binding cassette, sub-family C (CFTR/MRP), member 2 | chi-miR-16b-5p_R+1 |
| ABCC2 | ATP-binding cassette, sub-family C (CFTR/MRP), member 2 | gga-miR-16-5p |
| ABCC2 | ATP-binding cassette, sub-family C (CFTR/MRP), member 2 | gga-miR-1782_L+2R-1 |
| MPZL2 | myelin protein zero-like 2 | gga-miR-1456-5p_L-1 |
| CD3E | CD3e molecule, epsilon (CD3-TCR complex) | PC-3p-173245_25 |
| CD3E | CD3e molecule, epsilon (CD3-TCR complex) | PC-5p-113875_40 |
| CD3E | CD3e molecule, epsilon (CD3-TCR complex) | PC-5p-37716_121 |
| CD3D | CD3d molecule, delta (CD3-TCR complex) | chi-miR-16b-5p_R+1 |
| CD3D | CD3d molecule, delta (CD3-TCR complex) | gga-miR-16-5p |
| MASP1 | mannan-binding lectin serine peptidase 1 (C4/C2 activating component of Ra-reactive factor) | PC-5p-71982_65 |
| CUTC | cutC copper transporter homolog (E. coli) | PC-5p-582426_7 |
| CUTC | cutC copper transporter homolog (E. coli) | gga-miR-7468-3p_L+3 |
| PPRC1 | peroxisome proliferator-activated receptor gamma, coactivator-related 1 | PC-3p-282885_15 |
| ARCN1 | archain 1 | PC-3p-457530_9 |
| ARCN1 | archain 1 | gga-let-7g-3p_1ss22CT |
| ARCN1 | archain 1 | gga-miR-1782_L+2R-1 |
| RAB18 | RAB18, member RAS oncogene family | PC-5p-92816_49 |
| RAB18 | RAB18, member RAS oncogene family | PC-5p-97606_47 |
| RAB18 | RAB18, member RAS oncogene family | gga-miR-1782_L+2R-1 |
| RAB18 | RAB18, member RAS oncogene family | tgu-miR-2970-5p_1ss21GT |
| TMLHE | trimethyllysine hydroxylase, epsilon | PC-5p-113875_40 |
| TMLHE | trimethyllysine hydroxylase, epsilon | PC-5p-71982_65 |
| TMLHE | trimethyllysine hydroxylase, epsilon | gga-miR-7468-3p_L+3 |
| TMLHE | trimethyllysine hydroxylase, epsilon | hsa-miR-483-3p_L-1R+2 |
| TUBB1 | tubulin, beta 1 class VI | PC-5p-97606_47 |
| TUBB1 | tubulin, beta 1 class VI | chi-miR-16b-5p_R+1 |
| TUBB1 | tubulin, beta 1 class VI | gga-miR-16-5p |
| EDNRB2 | endothelin receptor B subtype 2 | PC-5p-113875_40 |
| SLC25A26 | solute carrier family 25 (S-adenosylmethionine carrier), member 26 | gga-miR-1677-3p_L+1 |
| CNNM1 | cyclin M1 | PC-3p-52432_89 |
| C10H15ORF44 | chromosome 10 open reading frame, human C15orf44 | PC-3p-457530_9 |
| C10H15ORF44 | chromosome 10 open reading frame, human C15orf44 | gga-let-7g-3p_1ss22CT |
| C10H15ORF44 | chromosome 10 open reading frame, human C15orf44 | gga-miR-34b-5p_L-1R+1 |
| YME1L1 | YME1-like 1 (S. cerevisiae) | PC-5p-582426_7 |
| YME1L1 | YME1-like 1 (S. cerevisiae) | gga-miR-1782_L+2R-1 |
| ITGB2 | integrin, beta 2 (complement component 3 receptor 3 and 4 subunit) | PC-5p-113875_40 |
| ITGB2 | integrin, beta 2 (complement component 3 receptor 3 and 4 subunit) | PC-5p-97606_47 |
| ITGB2 | integrin, beta 2 (complement component 3 receptor 3 and 4 subunit) | hsa-miR-483-3p_L-1R+2 |
| ACBD5 | acyl-CoA binding domain containing 5 | PC-5p-97606_47 |
| ACBD5 | acyl-CoA binding domain containing 5 | aca-miR-18a-5p_R-2 |
| ACBD5 | acyl-CoA binding domain containing 5 | chi-miR-16b-5p_R+1 |
| ACBD5 | acyl-CoA binding domain containing 5 | gga-miR-16-5p |
| TCERG1 | transcription elongation regulator 1 | PC-3p-173245_25 |
| ZFYVE27 | zinc finger, FYVE domain containing 27 | PC-5p-37716_121 |
| ZFYVE27 | zinc finger, FYVE domain containing 27 | chi-miR-16b-5p_R+1 |
| ZFYVE27 | zinc finger, FYVE domain containing 27 | gga-miR-16-5p |
| NPEPL1 | aminopeptidase-like 1 | PC-5p-97606_47 |
| NPEPL1 | aminopeptidase-like 1 | tgu-miR-2970-5p_1ss21GT |
| STX16 | syntaxin 16 | gga-miR-30a-5p_R+2_1ss13CT |
| STX16 | syntaxin 16 | gga-miR-30e-5p_R+5 |
| STX16 | syntaxin 16 | gga-miR-33-3p_L+1R+1 |
| RAB22A | RAB22A, member RAS oncogene family | PC-5p-92816_49 |
| RAB22A | RAB22A, member RAS oncogene family | gga-let-7g-3p_1ss22CT |
| RAB22A | RAB22A, member RAS oncogene family | gga-miR-30a-5p_R+2_1ss13CT |
| RAB22A | RAB22A, member RAS oncogene family | gga-miR-30e-5p_R+5 |
| KIAA0226 | KIAA0226 | PC-3p-52432_89 |
| CLCN7 | chloride channel, voltage-sensitive 7 | aca-miR-363-3p_R+1 |
| CLCN7 | chloride channel, voltage-sensitive 7 | gga-miR-34b-5p_L-1R+1 |
| CLCN7 | chloride channel, voltage-sensitive 7 | oan-miR-363-3p_R+1 |
| FYTTD1 | forty-two-three domain containing 1 | PC-3p-338480_12 |
| FYTTD1 | forty-two-three domain containing 1 | PC-5p-582426_7 |
| FYTTD1 | forty-two-three domain containing 1 | PC-5p-92816_49 |
| FYTTD1 | forty-two-three domain containing 1 | tgu-miR-2970-5p_1ss21GT |
| NIT1 | nitrilase 1 | gga-miR-1677-3p_L+1 |
| NIT1 | nitrilase 1 | tgu-miR-2970-5p_1ss21GT |
| MTMR8 | myotubularin related protein 8 | PC-3p-338480_12 |
| MTMR8 | myotubularin related protein 8 | aca-miR-363-3p_R+1 |
| MTMR8 | myotubularin related protein 8 | gga-miR-1456-5p_L-1 |
| MTMR8 | myotubularin related protein 8 | gga-miR-1677-3p_L+1 |
| MTMR8 | myotubularin related protein 8 | gga-miR-30a-5p_R+2_1ss13CT |
| MTMR8 | myotubularin related protein 8 | gga-miR-30e-5p_R+5 |
| MTMR8 | myotubularin related protein 8 | gga-miR-34b-5p_L-1R+1 |
| MTMR8 | myotubularin related protein 8 | hsa-miR-483-3p_L-1R+2 |
| MTMR8 | myotubularin related protein 8 | oan-miR-363-3p_R+1 |
| MTMR8 | myotubularin related protein 8 | tgu-miR-2970-5p_1ss21GT |
| RRP12 | ribosomal RNA processing 12 homolog (S. cerevisiae) | chi-miR-16b-5p_R+1 |
| RRP12 | ribosomal RNA processing 12 homolog (S. cerevisiae) | gga-miR-16-5p |
| RAB11A | RAB11A, member RAS oncogene family | PC-3p-173245_25 |
| RAB11A | RAB11A, member RAS oncogene family | PC-5p-233284_18 |
| RAB11A | RAB11A, member RAS oncogene family | PC-5p-97606_47 |
| RAB11A | RAB11A, member RAS oncogene family | aca-miR-363-3p_R+1 |
| RAB11A | RAB11A, member RAS oncogene family | gga-let-7g-3p_1ss22CT |
| RAB11A | RAB11A, member RAS oncogene family | oan-miR-363-3p_R+1 |
| BTG4 | B-cell translocation gene 4 | gga-miR-7468-3p_L+3 |
| HMOX2 | heme oxygenase (decycling) 2 | PC-3p-338480_12 |
| HMOX2 | heme oxygenase (decycling) 2 | hsa-miR-483-3p_L-1R+2 |
| LOC416354 | C-terminal binding protein-like | PC-3p-173245_25 |
| LOC416354 | C-terminal binding protein-like | PC-3p-457530_9 |
| LOC416354 | C-terminal binding protein-like | PC-5p-113875_40 |
| LOC416354 | C-terminal binding protein-like | PC-5p-233284_18 |
| LOC416354 | C-terminal binding protein-like | PC-5p-71982_65 |
| LOC416354 | C-terminal binding protein-like | hsa-miR-483-3p_L-1R+2 |
| LOC416354 | C-terminal binding protein-like | tgu-miR-2970-5p_1ss21GT |
| DIS3L | DIS3 mitotic control homolog (S. cerevisiae)-like | gga-miR-1677-3p_L+1 |
| TIPIN | TIMELESS interacting protein | PC-5p-233284_18 |
| SLC9A3R1 | solute carrier family 9, subfamily A (NHE3, cation proton antiporter 3), member 3 regulator 1 | PC-3p-282885_15 |
| STAT1 | signal transducer and activator of transcription 1, 91kDa | PC-5p-71982_65 |
| STAT1 | signal transducer and activator of transcription 1, 91kDa | gga-let-7g-3p_1ss22CT |
| STAT1 | signal transducer and activator of transcription 1, 91kDa | gga-miR-34b-5p_L-1R+1 |
| STAT1 | signal transducer and activator of transcription 1, 91kDa | hsa-miR-483-3p_L-1R+2 |
| SUCLG2 | succinate-CoA ligase, GDP-forming, beta subunit | PC-3p-173245_25 |
| SUCLG2 | succinate-CoA ligase, GDP-forming, beta subunit | gga-miR-30a-5p_R+2_1ss13CT |
| SUCLG2 | succinate-CoA ligase, GDP-forming, beta subunit | gga-miR-30e-5p_R+5 |
| CDX4 | caudal type homeobox 4 | chi-miR-16b-5p_R+1 |
| CDX4 | caudal type homeobox 4 | gga-miR-16-5p |
| C6H10ORF76 | chromosome 6 open reading frame, human C10orf76 | chi-miR-16b-5p_R+1 |
| C6H10ORF76 | chromosome 6 open reading frame, human C10orf76 | gga-miR-16-5p |
| CHIC1 | cysteine-rich hydrophobic domain 1 | PC-3p-173245_25 |
| CHIC1 | cysteine-rich hydrophobic domain 1 | PC-3p-338480_12 |
| CHIC1 | cysteine-rich hydrophobic domain 1 | aca-miR-363-3p_R+1 |
| CHIC1 | cysteine-rich hydrophobic domain 1 | chi-miR-16b-5p_R+1 |
| CHIC1 | cysteine-rich hydrophobic domain 1 | gga-miR-16-5p |
| CHIC1 | cysteine-rich hydrophobic domain 1 | oan-miR-363-3p_R+1 |
| EGR1 | early growth response 1 | tgu-miR-2970-5p_1ss21GT |
| PPFIA1 | protein tyrosine phosphatase, receptor type, f polypeptide (PTPRF), interacting protein (liprin), alpha 1 | gga-miR-33-3p_L+1R+1 |
| TRAP1 | TNF receptor-associated protein 1 | PC-3p-173245_25 |
| TRAP1 | TNF receptor-associated protein 1 | chi-miR-16b-5p_R+1 |
| TRAP1 | TNF receptor-associated protein 1 | gga-miR-16-5p |
| MAP2K1 | mitogen-activated protein kinase kinase 1 | chi-miR-16b-5p_R+1 |
| MAP2K1 | mitogen-activated protein kinase kinase 1 | gga-miR-16-5p |
| SNAPC5 | small nuclear RNA activating complex, polypeptide 5, 19kDa | gga-miR-1456-5p_L-1 |
| NPM3 | nucleophosmin/nucleoplasmin 3 | gga-miR-30a-5p_R+2_1ss13CT |
| NPM3 | nucleophosmin/nucleoplasmin 3 | gga-miR-30e-5p_R+5 |
| NCL | nucleolin | gga-miR-1782_L+2R-1 |
| C20H20ORF43 | chromosome 20 open reading frame, human C20orf43 | gga-let-7g-3p_1ss22CT |
| MYH7B | myosin, heavy chain 7B, cardiac muscle, beta | gga-miR-34b-5p_L-1R+1 |
| USP12P1 | ubiquitin specific peptidase 12 pseudogene 1 | PC-5p-97606_47 |
| ZWILCH | Zwilch, kinetochore associated, homolog (Drosophila) | aca-miR-363-3p_R+1 |
| ZWILCH | Zwilch, kinetochore associated, homolog (Drosophila) | gga-miR-1a-3p_R+1_1ss2GT |
| ZWILCH | Zwilch, kinetochore associated, homolog (Drosophila) | oan-miR-363-3p_R+1 |
| ECI1 | enoyl-CoA delta isomerase 1 | PC-5p-113875_40 |
| TMEM104 | transmembrane protein 104 | PC-5p-113875_40 |
| TMEM104 | transmembrane protein 104 | PC-5p-37716_121 |
| TMEM104 | transmembrane protein 104 | hsa-miR-483-3p_L-1R+2 |
| KIAA2022 | KIAA2022 | tgu-miR-2970-5p_1ss21GT |
| CSTF1 | cleavage stimulation factor, 3' pre-RNA, subunit 1, 50kDa | aca-miR-363-3p_R+1 |
| CSTF1 | cleavage stimulation factor, 3' pre-RNA, subunit 1, 50kDa | gga-miR-1782_L+2R-1 |
| CSTF1 | cleavage stimulation factor, 3' pre-RNA, subunit 1, 50kDa | oan-miR-363-3p_R+1 |
| FOXP1 | forkhead box P1 | PC-5p-233284_18 |
| FOXP1 | forkhead box P1 | gga-miR-7468-3p_L+3 |
| CBLN4 | cerebellin 4 precursor | PC-3p-52432_89 |
| CBLN4 | cerebellin 4 precursor | PC-5p-71982_65 |
| CBLN4 | cerebellin 4 precursor | PC-5p-97606_47 |
| CBLN4 | cerebellin 4 precursor | aca-miR-363-3p_R+1 |
| CBLN4 | cerebellin 4 precursor | oan-miR-363-3p_R+1 |
| EIF4E3 | eukaryotic translation initiation factor 4E family member 3 | chi-miR-16b-5p_R+1 |
| EIF4E3 | eukaryotic translation initiation factor 4E family member 3 | gga-miR-16-5p |
| UPRT | uracil phosphoribosyltransferase (FUR1) homolog (S. cerevisiae) | tgu-miR-2970-5p_1ss21GT |
| BCAS1 | breast carcinoma amplified sequence 1 | gga-miR-7468-3p_L+3 |
| C17ORF28 | chromosome 18 open reading frame, human C17orf28 | PC-5p-113875_40 |
| C17ORF28 | chromosome 18 open reading frame, human C17orf28 | PC-5p-97606_47 |
| APIP | APAF1 interacting protein | PC-3p-457530_9 |
| APIP | APAF1 interacting protein | PC-5p-113875_40 |
| APIP | APAF1 interacting protein | PC-5p-582426_7 |
| APIP | APAF1 interacting protein | PC-5p-97606_47 |
| APIP | APAF1 interacting protein | tgu-miR-2970-5p_1ss21GT |
| SALL4 | sal-like 4 (Drosophila) | chi-miR-16b-5p_R+1 |
| SALL4 | sal-like 4 (Drosophila) | gga-miR-16-5p |
| SALL4 | sal-like 4 (Drosophila) | gga-miR-1805-5p_L-1 |
| PUS10 | pseudouridylate synthase 10 | gga-let-7g-3p_1ss22CT |
| PUS10 | pseudouridylate synthase 10 | gga-miR-30a-5p_R+2_1ss13CT |
| PUS10 | pseudouridylate synthase 10 | gga-miR-30e-5p_R+5 |
| DNAH7 | dynein, axonemal, heavy chain 7 | gga-miR-1677-3p_L+1 |
| MAGT1 | magnesium transporter 1 | PC-5p-97606_47 |
| MAGT1 | magnesium transporter 1 | gga-let-7g-3p_1ss22CT |
| BCL11A | B-cell CLL/lymphoma 11A (zinc finger protein) | PC-5p-233284_18 |
| BCL11A | B-cell CLL/lymphoma 11A (zinc finger protein) | aca-miR-363-3p_R+1 |
| BCL11A | B-cell CLL/lymphoma 11A (zinc finger protein) | gga-let-7g-3p_1ss22CT |
| BCL11A | B-cell CLL/lymphoma 11A (zinc finger protein) | hsa-miR-483-3p_L-1R+2 |
| BCL11A | B-cell CLL/lymphoma 11A (zinc finger protein) | oan-miR-363-3p_R+1 |
| BCO2 | beta-carotene oxygenase 2 | PC-5p-233284_18 |
| BCO2 | beta-carotene oxygenase 2 | gga-let-7g-3p_1ss22CT |
| BCO2 | beta-carotene oxygenase 2 | gga-miR-34b-5p_L-1R+1 |
| VRK2 | vaccinia related kinase 2 | chi-miR-16b-5p_R+1 |
| VRK2 | vaccinia related kinase 2 | gga-miR-16-5p |
| SPAG6 | sperm associated antigen 6 | tgu-miR-2970-5p_1ss21GT |
| ATP7A | ATPase, Cu++ transporting, alpha polypeptide | PC-3p-457530_9 |
| ATP7A | ATPase, Cu++ transporting, alpha polypeptide | gga-miR-30a-5p_R+2_1ss13CT |
| ATP7A | ATPase, Cu++ transporting, alpha polypeptide | gga-miR-30e-5p_R+5 |
| CHL1 | cell adhesion molecule with homology to L1CAM (close homolog of L1) | PC-5p-97606_47 |
| CHL1 | cell adhesion molecule with homology to L1CAM (close homolog of L1) | gga-miR-34b-5p_L-1R+1 |
| STK17B | serine/threonine kinase 17b | PC-3p-338480_12 |
| STK17B | serine/threonine kinase 17b | PC-3p-457530_9 |
| STK17B | serine/threonine kinase 17b | gga-miR-30a-5p_R+2_1ss13CT |
| STK17B | serine/threonine kinase 17b | gga-miR-30e-5p_R+5 |
| ATP5H | ATP synthase, H+ transporting, mitochondrial Fo complex, subunit d | tgu-miR-2970-5p_1ss21GT |
| DLD | dihydrolipoamide dehydrogenase | PC-5p-113875_40 |
| DLD | dihydrolipoamide dehydrogenase | aca-miR-18a-5p_R-2 |
| KCTD2 | potassium channel tetramerisation domain containing 2 | PC-5p-97606_47 |
| KCTD2 | potassium channel tetramerisation domain containing 2 | chi-miR-16b-5p_R+1 |
| KCTD2 | potassium channel tetramerisation domain containing 2 | gga-let-7g-3p_1ss22CT |
| KCTD2 | potassium channel tetramerisation domain containing 2 | gga-miR-16-5p |
| KCTD2 | potassium channel tetramerisation domain containing 2 | gga-miR-1805-5p_L-1 |
| SF3A1 | splicing factor 3a, subunit 1, 120kDa | PC-3p-457530_9 |
| SF3A1 | splicing factor 3a, subunit 1, 120kDa | chi-miR-16b-5p_R+1 |
| SF3A1 | splicing factor 3a, subunit 1, 120kDa | gga-let-7g-3p_1ss22CT |
| SF3A1 | splicing factor 3a, subunit 1, 120kDa | gga-miR-16-5p |
| AMOT | angiomotin | PC-5p-233284_18 |
| AMOT | angiomotin | gga-miR-34b-5p_L-1R+1 |
| SLC16A5 | solute carrier family 16, member 5 (monocarboxylic acid transporter 6) | gga-let-7g-3p_1ss22CT |
| GTF3C3 | general transcription factor IIIC, polypeptide 3, 102kDa | aca-miR-363-3p_R+1 |
| GTF3C3 | general transcription factor IIIC, polypeptide 3, 102kDa | oan-miR-363-3p_R+1 |
| AHCYL2 | adenosylhomocysteinase-like 2 | gga-let-7g-3p_1ss22CT |
| HN1 | hematological and neurological expressed 1 | gga-let-7g-3p_1ss22CT |
| HN1 | hematological and neurological expressed 1 | tgu-miR-2970-5p_1ss21GT |
| UBE2H | ubiquitin-conjugating enzyme E2H | PC-5p-113875_40 |
| UBE2H | ubiquitin-conjugating enzyme E2H | PC-5p-582426_7 |
| UBE2H | ubiquitin-conjugating enzyme E2H | PC-5p-97606_47 |
| GGA3 | golgi-associated, gamma adaptin ear containing, ARF binding protein 3 | gga-miR-1782_L+2R-1 |
| GGA3 | golgi-associated, gamma adaptin ear containing, ARF binding protein 3 | gga-miR-30a-5p_R+2_1ss13CT |
| GGA3 | golgi-associated, gamma adaptin ear containing, ARF binding protein 3 | gga-miR-30e-5p_R+5 |
| SLC26A4 | solute carrier family 26, member 4 | gga-miR-33-3p_L+1R+1 |
| MRPS7 | mitochondrial ribosomal protein S7 | PC-5p-92816_49 |
| MRPS7 | mitochondrial ribosomal protein S7 | PC-5p-97606_47 |
| MRPS7 | mitochondrial ribosomal protein S7 | gga-let-7g-3p_1ss22CT |
| MRPS7 | mitochondrial ribosomal protein S7 | gga-miR-7468-3p_L+3 |
| ZC3HC1 | zinc finger, C3HC-type containing 1 | chi-miR-16b-5p_R+1 |
| ZC3HC1 | zinc finger, C3HC-type containing 1 | gga-miR-16-5p |
| BCAP29 | B-cell receptor-associated protein 29 | chi-miR-16b-5p_R+1 |
| BCAP29 | B-cell receptor-associated protein 29 | gga-miR-16-5p |
| PTPN1 | protein tyrosine phosphatase, non-receptor type 1 | gga-let-7g-3p_1ss22CT |
| UBE2V1 | ubiquitin-conjugating enzyme E2 variant 1 | chi-miR-16b-5p_R+1 |
| UBE2V1 | ubiquitin-conjugating enzyme E2 variant 1 | gga-miR-16-5p |
| UBE2V1 | ubiquitin-conjugating enzyme E2 variant 1 | gga-miR-34b-5p_L-1R+1 |
| RNF114 | ring finger protein 114 | PC-5p-97606_47 |
| RNF114 | ring finger protein 114 | chi-miR-16b-5p_R+1 |
| RNF114 | ring finger protein 114 | gga-miR-16-5p |
| RNF114 | ring finger protein 114 | gga-miR-1a-3p_R+1_1ss2GT |
| RNF114 | ring finger protein 114 | gga-miR-30a-5p_R+2_1ss13CT |
| RNF114 | ring finger protein 114 | gga-miR-30e-5p_R+5 |
| RNF114 | ring finger protein 114 | tgu-miR-2970-5p_1ss21GT |
| SPATA2 | spermatogenesis associated 2 | PC-5p-233284_18 |
| SPATA2 | spermatogenesis associated 2 | PC-5p-582426_7 |
| SPATA2 | spermatogenesis associated 2 | chi-miR-16b-5p_R+1 |
| SPATA2 | spermatogenesis associated 2 | gga-miR-16-5p |
| TMEM209 | transmembrane protein 209 | PC-5p-71982_65 |
| SF3B1 | splicing factor 3b, subunit 1, 155kDa | aca-miR-363-3p_R+1 |
| SF3B1 | splicing factor 3b, subunit 1, 155kDa | oan-miR-363-3p_R+1 |
| TMEM180 | transmembrane protein 180 | PC-3p-52432_89 |
| TMEM180 | transmembrane protein 180 | PC-5p-582426_7 |
| TMEM180 | transmembrane protein 180 | gga-miR-30a-5p_R+2_1ss13CT |
| TMEM180 | transmembrane protein 180 | gga-miR-30e-5p_R+5 |
| TMEM180 | transmembrane protein 180 | hsa-miR-483-3p_L-1R+2 |
| LOC417013 | putative acyl-CoA dehydrogenase AidB-like | chi-miR-16b-5p_R+1 |
| LOC417013 | putative acyl-CoA dehydrogenase AidB-like | gga-miR-16-5p |
| CD82 | CD82 molecule | aca-miR-18a-5p_R-2 |
| CD82 | CD82 molecule | chi-miR-16b-5p_R+1 |
| CD82 | CD82 molecule | gga-miR-16-5p |
| SLC9A8 | solute carrier family 9, subfamily A (NHE8, cation proton antiporter 8), member 8 | gga-let-7g-3p_1ss22CT |
| COQ10B | coenzyme Q10 homolog B (S. cerevisiae) | aca-miR-363-3p_R+1 |
| COQ10B | coenzyme Q10 homolog B (S. cerevisiae) | oan-miR-363-3p_R+1 |
| ARL3 | ADP-ribosylation factor-like 3 | PC-5p-71982_65 |
| ARL3 | ADP-ribosylation factor-like 3 | chi-miR-16b-5p_R+1 |
| ARL3 | ADP-ribosylation factor-like 3 | gga-miR-16-5p |
| ANP32A | acidic (leucine-rich) nuclear phosphoprotein 32 family, member A | gga-miR-34b-5p_L-1R+1 |
| ANP32A | acidic (leucine-rich) nuclear phosphoprotein 32 family, member A | tgu-miR-2970-5p_1ss21GT |
| ASTL | astacin-like metallo-endopeptidase (M12 family) | PC-5p-582426_7 |
| UQCR10 | ubiquinol-cytochrome c reductase, complex III subunit X | chi-miR-16b-5p_R+1 |
| UQCR10 | ubiquinol-cytochrome c reductase, complex III subunit X | gga-miR-16-5p |
| CHRDL1 | chordin-like 1 | PC-3p-457530_9 |
| CHRDL1 | chordin-like 1 | PC-5p-233284_18 |
| CHRDL1 | chordin-like 1 | PC-5p-71982_65 |
| CHRDL1 | chordin-like 1 | chi-miR-16b-5p_R+1 |
| CHRDL1 | chordin-like 1 | gga-miR-16-5p |
| TMEM164 | transmembrane protein 164 | gga-let-7g-3p_1ss22CT |
| TMEM164 | transmembrane protein 164 | gga-miR-1a-3p_R+1_1ss2GT |
| TMEM164 | transmembrane protein 164 | gga-miR-34b-5p_L-1R+1 |
| TMEM164 | transmembrane protein 164 | tgu-miR-2970-5p_1ss21GT |
| NDUFS3 | NADH dehydrogenase (ubiquinone) Fe-S protein 3, 30kDa (NADH-coenzyme Q reductase) | PC-5p-113875_40 |
| NDUFS3 | NADH dehydrogenase (ubiquinone) Fe-S protein 3, 30kDa (NADH-coenzyme Q reductase) | chi-miR-16b-5p_R+1 |
| NDUFS3 | NADH dehydrogenase (ubiquinone) Fe-S protein 3, 30kDa (NADH-coenzyme Q reductase) | gga-miR-16-5p |
| ACTR1A | ARP1 actin-related protein 1 homolog A, centractin alpha (yeast) | gga-miR-30a-5p_R+2_1ss13CT |
| ACTR1A | ARP1 actin-related protein 1 homolog A, centractin alpha (yeast) | gga-miR-30e-5p_R+5 |
| ACSL4 | acyl-CoA synthetase long-chain family member 4 | gga-miR-1782_L+2R-1 |
| NXT2 | nuclear transport factor 2-like export factor 2 | PC-3p-173245_25 |
| HSPD1 | heat shock 60kDa protein 1 (chaperonin) | gga-miR-1782_L+2R-1 |
| HSPD1 | heat shock 60kDa protein 1 (chaperonin) | gga-miR-30a-5p_R+2_1ss13CT |
| HSPD1 | heat shock 60kDa protein 1 (chaperonin) | gga-miR-30e-5p_R+5 |
| KIF23 | kinesin family member 23 | PC-5p-113875_40 |
| KIF23 | kinesin family member 23 | PC-5p-582426_7 |
| KIF23 | kinesin family member 23 | PC-5p-97606_47 |
| CELF1 | CUGBP, Elav-like family member 1 | PC-3p-457530_9 |
| CELF1 | CUGBP, Elav-like family member 1 | gga-miR-1782_L+2R-1 |
| CELF1 | CUGBP, Elav-like family member 1 | gga-miR-34b-5p_L-1R+1 |
| TRIM8 | tripartite motif containing 8 | PC-5p-97606_47 |
| TRIM8 | tripartite motif containing 8 | hsa-miR-483-3p_L-1R+2 |
| CEP41 | centrosomal protein 41kDa | PC-5p-71982_65 |
| PLCL1 | phospholipase C-like 1 | PC-5p-97606_47 |
| SFXN2 | sideroflexin 2 | PC-3p-52432_89 |
| SFXN2 | sideroflexin 2 | PC-5p-113875_40 |
| SFXN2 | sideroflexin 2 | gga-miR-34b-5p_L-1R+1 |
| SLC39A13 | solute carrier family 39 (zinc transporter), member 13 | PC-5p-71982_65 |
| SLC39A13 | solute carrier family 39 (zinc transporter), member 13 | hsa-miR-483-3p_L-1R+2 |
| C6H10ORF32 | chromosome 6 open reading frame, human C10orf32 | PC-5p-97606_47 |
| MOB4 | MOB family member 4, phocein | PC-5p-233284_18 |
| MOB4 | MOB family member 4, phocein | chi-miR-16b-5p_R+1 |
| MOB4 | MOB family member 4, phocein | gga-miR-16-5p |
| MOB4 | MOB family member 4, phocein | gga-miR-30a-5p_R+2_1ss13CT |
| MOB4 | MOB family member 4, phocein | gga-miR-30e-5p_R+5 |
| RTN4 | reticulon 4 | PC-3p-457530_9 |
| RTN4 | reticulon 4 | PC-5p-92816_49 |
| RTN4 | reticulon 4 | aca-miR-363-3p_R+1 |
| RTN4 | reticulon 4 | gga-let-7g-3p_1ss22CT |
| RTN4 | reticulon 4 | oan-miR-363-3p_R+1 |
| PUS7 | pseudouridylate synthase 7 homolog (S. cerevisiae) | gga-let-7g-3p_1ss22CT |
| UACA | uveal autoantigen with coiled-coil domains and ankyrin repeats | tgu-miR-2970-5p_1ss21GT |
| COPG2 | coatomer protein complex, subunit gamma 2 | PC-5p-97606_47 |
| COPG2 | coatomer protein complex, subunit gamma 2 | tgu-miR-2970-5p_1ss21GT |
| WSB2 | WD repeat and SOCS box containing 2 | aca-miR-363-3p_R+1 |
| WSB2 | WD repeat and SOCS box containing 2 | oan-miR-363-3p_R+1 |
| SRPK2 | SRSF protein kinase 2 | gga-let-7g-3p_1ss22CT |
| SRPK2 | SRSF protein kinase 2 | gga-miR-1677-3p_L+1 |
| SRPK2 | SRSF protein kinase 2 | tgu-miR-2970-5p_1ss21GT |
| FBXO18 | F-box protein, helicase, 18 | PC-5p-113875_40 |
| FBXO18 | F-box protein, helicase, 18 | gga-miR-1677-3p_L+1 |
| ADAMTS7 | ADAM metallopeptidase with thrombospondin type 1 motif, 7 | PC-5p-97606_47 |
| ANKRD16 | ankyrin repeat domain 16 | PC-5p-113875_40 |
| GDI2 | GDP dissociation inhibitor 2 | PC-3p-52432_89 |
| GDI2 | GDP dissociation inhibitor 2 | gga-miR-30a-5p_R+2_1ss13CT |
| GDI2 | GDP dissociation inhibitor 2 | gga-miR-30e-5p_R+5 |
| GDI2 | GDP dissociation inhibitor 2 | gga-miR-33-3p_L+1R+1 |
| MTAP | methylthioadenosine phosphorylase | gga-miR-1782_L+2R-1 |
| ORC5 | origin recognition complex, subunit 5 | PC-3p-338480_12 |
| ORC5 | origin recognition complex, subunit 5 | PC-5p-71982_65 |
| ORC5 | origin recognition complex, subunit 5 | PC-5p-97606_47 |
| ORC5 | origin recognition complex, subunit 5 | gga-miR-33-3p_L+1R+1 |
| NT5C2 | 5'-nucleotidase, cytosolic II | chi-miR-16b-5p_R+1 |
| NT5C2 | 5'-nucleotidase, cytosolic II | gga-miR-16-5p |
| NT5C2 | 5'-nucleotidase, cytosolic II | gga-miR-1a-3p_R+1_1ss2GT |
| AOX2P | aldehyde oxidase 2 pseudogene | gga-let-7g-3p_1ss22CT |
| AOX2P | aldehyde oxidase 2 pseudogene | gga-miR-34b-5p_L-1R+1 |
| EIF3J | eukaryotic translation initiation factor 3, subunit J | PC-3p-52432_89 |
| EIF3J | eukaryotic translation initiation factor 3, subunit J | PC-5p-37716_121 |
| TRIM36 | tripartite motif containing 36 | PC-5p-71982_65 |
| RELN | reelin | PC-5p-113875_40 |
| RELN | reelin | PC-5p-97606_47 |
| RELN | reelin | chi-miR-16b-5p_R+1 |
| RELN | reelin | gga-miR-16-5p |
| RELN | reelin | gga-miR-1805-5p_L-1 |
| PGGT1B | protein geranylgeranyltransferase type I, beta subunit | gga-miR-30a-5p_R+2_1ss13CT |
| PGGT1B | protein geranylgeranyltransferase type I, beta subunit | gga-miR-30e-5p_R+5 |
| NR1H3 | nuclear receptor subfamily 1, group H, member 3 | PC-3p-173245_25 |
| NR1H3 | nuclear receptor subfamily 1, group H, member 3 | PC-3p-457530_9 |
| NR1H3 | nuclear receptor subfamily 1, group H, member 3 | aca-miR-18a-5p_R-2 |
| FBXW8 | F-box and WD repeat domain containing 8 | PC-5p-71982_65 |
| CASC4 | cancer susceptibility candidate 4 | PC-3p-338480_12 |
| CASC4 | cancer susceptibility candidate 4 | PC-5p-113875_40 |
| CASC4 | cancer susceptibility candidate 4 | gga-let-7g-3p_1ss22CT |
| ACP2 | acid phosphatase 2, lysosomal | tgu-miR-2970-5p_1ss21GT |
| RNFT2 | ring finger protein, transmembrane 2 | PC-5p-71982_65 |
| C15H12ORF49 | chromosome 15 open reading frame, human C12orf49 | PC-5p-582426_7 |
| C15H12ORF49 | chromosome 15 open reading frame, human C12orf49 | aca-miR-363-3p_R+1 |
| C15H12ORF49 | chromosome 15 open reading frame, human C12orf49 | chi-miR-16b-5p_R+1 |
| C15H12ORF49 | chromosome 15 open reading frame, human C12orf49 | gga-miR-16-5p |
| C15H12ORF49 | chromosome 15 open reading frame, human C12orf49 | gga-miR-1782_L+2R-1 |
| C15H12ORF49 | chromosome 15 open reading frame, human C12orf49 | gga-miR-7468-3p_L+3 |
| C15H12ORF49 | chromosome 15 open reading frame, human C12orf49 | oan-miR-363-3p_R+1 |
| ALDH7A1 | aldehyde dehydrogenase 7 family, member A1 | gga-miR-1782_L+2R-1 |
| PCGF6 | polycomb group ring finger 6 | gga-let-7g-3p_1ss22CT |
| BUB1 | budding uninhibited by benzimidazoles 1 homolog (yeast) | gga-let-7g-3p_1ss22CT |
| BUB1 | budding uninhibited by benzimidazoles 1 homolog (yeast) | gga-miR-1a-3p_R+1_1ss2GT |
| BUB1 | budding uninhibited by benzimidazoles 1 homolog (yeast) | gga-miR-33-3p_L+1R+1 |
| ORC2 | origin recognition complex, subunit 2 | PC-3p-457530_9 |
| MED13L | mediator complex subunit 13-like | PC-3p-52432_89 |
| CFLAR | CASP8 and FADD-like apoptosis regulator | PC-3p-173245_25 |
| TBX3 | T-box 3 | PC-5p-37716_121 |
| PDCD11 | programmed cell death 11 | gga-miR-1782_L+2R-1 |
| NEURL | neuralized homolog (Drosophila) | PC-3p-52432_89 |
| NEURL | neuralized homolog (Drosophila) | PC-5p-97606_47 |
| NEURL | neuralized homolog (Drosophila) | aca-miR-363-3p_R+1 |
| NEURL | neuralized homolog (Drosophila) | oan-miR-363-3p_R+1 |
| ATG4A | ATG4 autophagy related 4 homolog A (S. cerevisiae) | PC-3p-338480_12 |
| ATG4A | ATG4 autophagy related 4 homolog A (S. cerevisiae) | PC-5p-71982_65 |
| ATG4A | ATG4 autophagy related 4 homolog A (S. cerevisiae) | gga-miR-34b-5p_L-1R+1 |
| NAPEPLD | N-acyl phosphatidylethanolamine phospholipase D | gga-miR-30a-5p_R+2_1ss13CT |
| NAPEPLD | N-acyl phosphatidylethanolamine phospholipase D | gga-miR-30e-5p_R+5 |
| SUMF1 | sulfatase modifying factor 1 | PC-5p-71982_65 |
| PSMD10 | proteasome (prosome, macropain) 26S subunit, non-ATPase, 10 | gga-miR-34b-5p_L-1R+1 |
| PSMD10 | proteasome (prosome, macropain) 26S subunit, non-ATPase, 10 | tgu-miR-2970-5p_1ss21GT |
| ARMC10 | armadillo repeat containing 10 | PC-5p-582426_7 |
| FBXL13 | F-box and leucine-rich repeat protein 13 | gga-miR-34b-5p_L-1R+1 |
| LRRC17 | leucine rich repeat containing 17 | gga-miR-30a-5p_R+2_1ss13CT |
| LRRC17 | leucine rich repeat containing 17 | gga-miR-30e-5p_R+5 |
| LRRC17 | leucine rich repeat containing 17 | gga-miR-33-3p_L+1R+1 |
| FGL2 | fibrinogen-like 2 | PC-3p-338480_12 |
| FGL2 | fibrinogen-like 2 | aca-miR-363-3p_R+1 |
| FGL2 | fibrinogen-like 2 | gga-miR-1677-3p_L+1 |
| FGL2 | fibrinogen-like 2 | oan-miR-363-3p_R+1 |
| NAPB | N-ethylmaleimide-sensitive factor attachment protein, beta | tgu-miR-2970-5p_1ss21GT |
| EDEM1 | ER degradation enhancer, mannosidase alpha-like 1 | PC-3p-173245_25 |
| F2 | coagulation factor II (thrombin) | PC-3p-52432_89 |
| F2 | coagulation factor II (thrombin) | gga-miR-7468-3p_L+3 |
| PHTF2 | putative homeodomain transcription factor 2 | PC-5p-582426_7 |
| PHTF2 | putative homeodomain transcription factor 2 | PC-5p-71982_65 |
| PHTF2 | putative homeodomain transcription factor 2 | PC-5p-92816_49 |
| PHTF2 | putative homeodomain transcription factor 2 | PC-5p-97606_47 |
| PHTF2 | putative homeodomain transcription factor 2 | aca-miR-363-3p_R+1 |
| PHTF2 | putative homeodomain transcription factor 2 | gga-miR-1677-3p_L+1 |
| PHTF2 | putative homeodomain transcription factor 2 | gga-miR-1782_L+2R-1 |
| PHTF2 | putative homeodomain transcription factor 2 | gga-miR-1805-5p_L-1 |
| PHTF2 | putative homeodomain transcription factor 2 | gga-miR-30a-5p_R+2_1ss13CT |
| PHTF2 | putative homeodomain transcription factor 2 | gga-miR-30e-5p_R+5 |
| PHTF2 | putative homeodomain transcription factor 2 | gga-miR-33-3p_L+1R+1 |
| PHTF2 | putative homeodomain transcription factor 2 | oan-miR-363-3p_R+1 |
| PHTF2 | putative homeodomain transcription factor 2 | tgu-miR-2970-5p_1ss21GT |
| ATG13 | autophagy related 13 | gga-miR-1677-3p_L+1 |
| ATG13 | autophagy related 13 | gga-miR-7468-3p_L+3 |
| PDIA3 | protein disulfide isomerase family A, member 3 | PC-3p-173245_25 |
| CKMT1A | creatine kinase, mitochondrial 1A | PC-5p-37716_121 |
| CKMT1A | creatine kinase, mitochondrial 1A | chi-miR-16b-5p_R+1 |
| CKMT1A | creatine kinase, mitochondrial 1A | gga-miR-16-5p |
| MDK | midkine (neurite growth-promoting factor 2) | PC-5p-37716_121 |
| MDK | midkine (neurite growth-promoting factor 2) | PC-5p-71982_65 |
| RAD18 | RAD18 homolog (S. cerevisiae) | chi-miR-16b-5p_R+1 |
| RAD18 | RAD18 homolog (S. cerevisiae) | gga-miR-16-5p |
| STRADB | STE20-related kinase adaptor beta | PC-3p-338480_12 |
| STRADB | STE20-related kinase adaptor beta | PC-5p-92816_49 |
| STRADB | STE20-related kinase adaptor beta | chi-miR-16b-5p_R+1 |
| STRADB | STE20-related kinase adaptor beta | gga-miR-16-5p |
| STRADB | STE20-related kinase adaptor beta | gga-miR-7468-3p_L+3 |
| EIF2B5 | eukaryotic translation initiation factor 2B, subunit 5 epsilon, 82kDa | gga-miR-34b-5p_L-1R+1 |
| VBP1 | von Hippel-Lindau binding protein 1 | gga-miR-1782_L+2R-1 |
| THUMPD3 | THUMP domain containing 3 | PC-5p-71982_65 |
| MPP4 | membrane protein, palmitoylated 4 (MAGUK p55 subfamily member 4) | PC-3p-173245_25 |
| MPP4 | membrane protein, palmitoylated 4 (MAGUK p55 subfamily member 4) | aca-miR-18a-5p_R-2 |
| RALGAPA2 | Ral GTPase activating protein, alpha subunit 2 (catalytic) | gga-miR-1329-3p |
| CDK15 | cyclin-dependent kinase 15 | PC-5p-582426_7 |
| CDK15 | cyclin-dependent kinase 15 | gga-miR-1a-3p_R+1_1ss2GT |
| CRNKL1 | crooked neck pre-mRNA splicing factor-like 1 (Drosophila) | PC-5p-582426_7 |
| SMARCA1 | SWI/SNF related, matrix associated, actin dependent regulator of chromatin, subfamily a, member 1 | PC-3p-457530_9 |
| SMARCA1 | SWI/SNF related, matrix associated, actin dependent regulator of chromatin, subfamily a, member 1 | chi-miR-16b-5p_R+1 |
| SMARCA1 | SWI/SNF related, matrix associated, actin dependent regulator of chromatin, subfamily a, member 1 | gga-miR-16-5p |
| SMARCA1 | SWI/SNF related, matrix associated, actin dependent regulator of chromatin, subfamily a, member 1 | gga-miR-1782_L+2R-1 |
| AP2M1 | adaptor-related protein complex 2, mu 1 subunit | PC-3p-173245_25 |
| AP2M1 | adaptor-related protein complex 2, mu 1 subunit | PC-5p-37716_121 |
| AP2M1 | adaptor-related protein complex 2, mu 1 subunit | chi-miR-16b-5p_R+1 |
| AP2M1 | adaptor-related protein complex 2, mu 1 subunit | gga-miR-16-5p |
| AP2M1 | adaptor-related protein complex 2, mu 1 subunit | gga-miR-1a-3p_R+1_1ss2GT |
| SUMO1 | SMT3 suppressor of mif two 3 homolog 1 (S. cerevisiae) | PC-3p-338480_12 |
| SUMO1 | SMT3 suppressor of mif two 3 homolog 1 (S. cerevisiae) | PC-3p-52432_89 |
| CRY2 | cryptochrome 2 (photolyase-like) | PC-3p-52432_89 |
| NET1 | neuroepithelial cell transforming 1 | PC-5p-233284_18 |
| NET1 | neuroepithelial cell transforming 1 | PC-5p-37716_121 |
| NET1 | neuroepithelial cell transforming 1 | gga-miR-34b-5p_L-1R+1 |
| ARHGDIA | Rho GDP dissociation inhibitor (GDI) alpha | chi-miR-16b-5p_R+1 |
| ARHGDIA | Rho GDP dissociation inhibitor (GDI) alpha | gga-miR-16-5p |
| NOP58 | NOP58 ribonucleoprotein homolog (yeast) | PC-3p-457530_9 |
| ZFYVE19 | zinc finger, FYVE domain containing 19 | gga-miR-1456-5p_L-1 |
| WDR12 | WD repeat domain 12 | PC-5p-71982_65 |
| ARRDC1 | arrestin domain containing 1 | PC-5p-582426_7 |
| ARRDC1 | arrestin domain containing 1 | chi-miR-16b-5p_R+1 |
| ARRDC1 | arrestin domain containing 1 | gga-miR-1456-5p_L-1 |
| ARRDC1 | arrestin domain containing 1 | gga-miR-16-5p |
| STAG2 | stromal antigen 2 | gga-miR-1677-3p_L+1 |
| STAG2 | stromal antigen 2 | gga-miR-1782_L+2R-1 |
| TRH | thyrotropin-releasing hormone | PC-5p-233284_18 |
| TRH | thyrotropin-releasing hormone | PC-5p-71982_65 |
| TRH | thyrotropin-releasing hormone | PC-5p-97606_47 |
| XIAP | X-linked inhibitor of apoptosis | PC-3p-338480_12 |
| SLC38A2 | solute carrier family 38, member 2 | gga-miR-1677-3p_L+1 |
| HNRPLL | heterogeneous nuclear ribonucleoprotein L-like | gga-miR-1a-3p_R+1_1ss2GT |
| GRIA3 | glutamate receptor, ionotropic, AMPA 3 | aca-miR-363-3p_R+1 |
| GRIA3 | glutamate receptor, ionotropic, AMPA 3 | oan-miR-363-3p_R+1 |
| MCTS1 | malignant T cell amplified sequence 1 | PC-5p-71982_65 |
| MCTS1 | malignant T cell amplified sequence 1 | gga-miR-1677-3p_L+1 |
| MCTS1 | malignant T cell amplified sequence 1 | gga-miR-1782_L+2R-1 |
| MCTS1 | malignant T cell amplified sequence 1 | gga-miR-30a-5p_R+2_1ss13CT |
| MCTS1 | malignant T cell amplified sequence 1 | gga-miR-30e-5p_R+5 |
| LOC417253 | glutamine synthetase-like | PC-5p-97606_47 |
| ABHD12 | abhydrolase domain containing 12 | chi-miR-16b-5p_R+1 |
| ABHD12 | abhydrolase domain containing 12 | gga-miR-16-5p |
| FASTKD2 | FAST kinase domains 2 | PC-3p-338480_12 |
| FASTKD2 | FAST kinase domains 2 | PC-3p-457530_9 |
| FASTKD2 | FAST kinase domains 2 | chi-miR-16b-5p_R+1 |
| FASTKD2 | FAST kinase domains 2 | gga-miR-16-5p |
| COBRA1 | cofactor of BRCA1 | gga-let-7g-3p_1ss22CT |
| COBRA1 | cofactor of BRCA1 | tgu-miR-2970-5p_1ss21GT |
| POLR2H | polymerase (RNA) II (DNA directed) polypeptide H | PC-3p-52432_89 |
| POLR2H | polymerase (RNA) II (DNA directed) polypeptide H | PC-5p-97606_47 |
| SENP5 | SUMO1/sentrin specific peptidase 5 | aca-miR-18a-5p_R-2 |
| SENP5 | SUMO1/sentrin specific peptidase 5 | gga-miR-30a-5p_R+2_1ss13CT |
| SENP5 | SUMO1/sentrin specific peptidase 5 | gga-miR-30e-5p_R+5 |
| TUBGCP4 | tubulin, gamma complex associated protein 4 | chi-miR-16b-5p_R+1 |
| TUBGCP4 | tubulin, gamma complex associated protein 4 | gga-miR-16-5p |
| CRELD2 | cysteine-rich with EGF-like domains 2 | PC-3p-457530_9 |
| CUL4B | cullin 4B | hsa-miR-483-3p_L-1R+2 |
| SMNDC1 | survival motor neuron domain containing 1 | chi-miR-16b-5p_R+1 |
| SMNDC1 | survival motor neuron domain containing 1 | gga-miR-16-5p |
| LAMP2 | lysosomal-associated membrane protein 2 | PC-5p-97606_47 |
| CLNS1A | chloride channel, nucleotide-sensitive, 1A | PC-3p-52432_89 |
| CLNS1A | chloride channel, nucleotide-sensitive, 1A | tgu-miR-2970-5p_1ss21GT |
| DUSP5 | dual specificity phosphatase 5 | aca-miR-363-3p_R+1 |
| DUSP5 | dual specificity phosphatase 5 | oan-miR-363-3p_R+1 |
| ADAM23 | ADAM metallopeptidase domain 23 | aca-miR-363-3p_R+1 |
| ADAM23 | ADAM metallopeptidase domain 23 | oan-miR-363-3p_R+1 |
| NUSAP1 | nucleolar and spindle associated protein 1 | gga-miR-1782_L+2R-1 |
| NDUFAF1 | NADH dehydrogenase (ubiquinone) complex I, assembly factor 1 | gga-miR-33-3p_L+1R+1 |
| AHSG | alpha-2-HS-glycoprotein | PC-5p-233284_18 |
| SLC39A12 | solute carrier family 39 (zinc transporter), member 12 | PC-5p-37716_121 |
| ITPKA | inositol 1,4,5-trisphosphate 3-kinase A | hsa-miR-483-3p_L-1R+2 |
| NDUFS1 | NADH dehydrogenase (ubiquinone) Fe-S protein 1, 75kDa (NADH-coenzyme Q reductase) | PC-3p-457530_9 |
| NDUFS1 | NADH dehydrogenase (ubiquinone) Fe-S protein 1, 75kDa (NADH-coenzyme Q reductase) | PC-5p-92816_49 |
| NDUFS1 | NADH dehydrogenase (ubiquinone) Fe-S protein 1, 75kDa (NADH-coenzyme Q reductase) | gga-miR-1782_L+2R-1 |
| PTK7 | PTK7 protein tyrosine kinase 7 | chi-miR-16b-5p_R+1 |
| PTK7 | PTK7 protein tyrosine kinase 7 | gga-miR-1456-3p_R+1 |
| PTK7 | PTK7 protein tyrosine kinase 7 | gga-miR-16-5p |
| TTL | tubulin tyrosine ligase | PC-3p-52432_89 |
| GTF2H4 | general transcription factor IIH, polypeptide 4, 52kDa | PC-3p-173245_25 |
| GTF2H4 | general transcription factor IIH, polypeptide 4, 52kDa | PC-5p-71982_65 |
| GTF2H4 | general transcription factor IIH, polypeptide 4, 52kDa | PC-5p-97606_47 |
| GTF2H4 | general transcription factor IIH, polypeptide 4, 52kDa | aca-miR-363-3p_R+1 |
| GTF2H4 | general transcription factor IIH, polypeptide 4, 52kDa | chi-miR-16b-5p_R+1 |
| GTF2H4 | general transcription factor IIH, polypeptide 4, 52kDa | gga-miR-16-5p |
| GTF2H4 | general transcription factor IIH, polypeptide 4, 52kDa | gga-miR-1782_L+2R-1 |
| GTF2H4 | general transcription factor IIH, polypeptide 4, 52kDa | oan-miR-363-3p_R+1 |
| ANAPC2 | anaphase promoting complex subunit 2 | aca-miR-425-3p_L-2R+1_1ss3AT |
| TYRO3 | TYRO3 protein tyrosine kinase | PC-5p-582426_7 |
| TYRO3 | TYRO3 protein tyrosine kinase | PC-5p-97606_47 |
| ACSS1 | acyl-CoA synthetase short-chain family member 1 | PC-5p-113875_40 |
| CXORF56 | chromosome 4 open reading frame, human CXorf56 | gga-miR-33-3p_L+1R+1 |
| MGA | MAX gene associated | PC-5p-233284_18 |
| MGA | MAX gene associated | gga-miR-7468-3p_L+3 |
| TUBGCP6 | tubulin, gamma complex associated protein 6 | hsa-miR-483-3p_L-1R+2 |
| CYR61 | cysteine-rich, angiogenic inducer, 61 | aca-miR-18a-5p_R-2 |
| DSTN | destrin (actin depolymerizing factor) | aca-miR-425-3p_L-2R+1_1ss3AT |
| KNG1 | kininogen 1 | PC-5p-97606_47 |
| SYDE2 | synapse defective 1, Rho GTPase, homolog 2 (C. elegans) | PC-3p-282885_15 |
| NELF | nasal embryonic LHRH factor | PC-5p-97606_47 |
| NELF | nasal embryonic LHRH factor | gga-miR-1677-3p_L+1 |
| NELF | nasal embryonic LHRH factor | gga-miR-7468-3p_L+3 |
| TRDMT1 | tRNA aspartic acid methyltransferase 1 | PC-5p-71982_65 |
| TRDMT1 | tRNA aspartic acid methyltransferase 1 | gga-miR-1456-5p_L-1 |
| TRDMT1 | tRNA aspartic acid methyltransferase 1 | tgu-miR-2970-5p_1ss21GT |
| RBM20 | RNA binding motif protein 20 | PC-3p-173245_25 |
| RBM20 | RNA binding motif protein 20 | PC-5p-71982_65 |
| RBM20 | RNA binding motif protein 20 | gga-miR-7468-3p_L+3 |
| PDCD4 | programmed cell death 4 (neoplastic transformation inhibitor) | PC-5p-582426_7 |
| PDCD4 | programmed cell death 4 (neoplastic transformation inhibitor) | chi-miR-16b-5p_R+1 |
| PDCD4 | programmed cell death 4 (neoplastic transformation inhibitor) | gga-miR-16-5p |
| XDH | xanthine dehydrogenase | PC-3p-52432_89 |
| TRABD | TraB domain containing | gga-miR-1a-3p_R+1_1ss2GT |
| TRABD | TraB domain containing | gga-miR-30a-5p_R+2_1ss13CT |
| TRABD | TraB domain containing | gga-miR-30e-5p_R+5 |
| CSRP2BP | CSRP2 binding protein | aca-miR-363-3p_R+1 |
| CSRP2BP | CSRP2 binding protein | oan-miR-363-3p_R+1 |
| GRM8 | glutamate receptor, metabotropic 8 | gga-miR-1782_L+2R-1 |
| GRM8 | glutamate receptor, metabotropic 8 | gga-miR-1a-3p_R+1_1ss2GT |
| RSU1 | Ras suppressor protein 1 | PC-3p-338480_12 |
| RSU1 | Ras suppressor protein 1 | chi-miR-16b-5p_R+1 |
| RSU1 | Ras suppressor protein 1 | gga-miR-16-5p |
| CYP20A1 | cytochrome P450, family 20, subfamily A, polypeptide 1 | PC-5p-582426_7 |
| LOC424109 | alpha-aspartyl dipeptidase | PC-5p-92816_49 |
| FAM188A | family with sequence similarity 188, member A | tgu-miR-2970-5p_1ss21GT |
| ITGA8 | integrin, alpha 8 | chi-miR-16b-5p_R+1 |
| ITGA8 | integrin, alpha 8 | gga-let-7g-3p_1ss22CT |
| ITGA8 | integrin, alpha 8 | gga-miR-16-5p |
| ITGA8 | integrin, alpha 8 | gga-miR-34b-5p_L-1R+1 |
| PPP3R1 | protein phosphatase 3, regulatory subunit B, alpha | gga-miR-1782_L+2R-1 |
| PPP3R1 | protein phosphatase 3, regulatory subunit B, alpha | gga-miR-30a-5p_R+2_1ss13CT |
| PPP3R1 | protein phosphatase 3, regulatory subunit B, alpha | gga-miR-30e-5p_R+5 |
| C1D | C1D nuclear receptor corepressor | gga-miR-1782_L+2R-1 |
| SHOC2 | soc-2 suppressor of clear homolog (C. elegans) | PC-5p-582426_7 |
| SHOC2 | soc-2 suppressor of clear homolog (C. elegans) | aca-miR-363-3p_R+1 |
| SHOC2 | soc-2 suppressor of clear homolog (C. elegans) | gga-miR-30a-5p_R+2_1ss13CT |
| SHOC2 | soc-2 suppressor of clear homolog (C. elegans) | gga-miR-30e-5p_R+5 |
| SHOC2 | soc-2 suppressor of clear homolog (C. elegans) | oan-miR-363-3p_R+1 |
| RPF1 | ribosome production factor 1 homolog (S. cerevisiae) | chi-miR-16b-5p_R+1 |
| RPF1 | ribosome production factor 1 homolog (S. cerevisiae) | gga-miR-16-5p |
| WASL | Wiskott-Aldrich syndrome-like | aca-miR-363-3p_R+1 |
| WASL | Wiskott-Aldrich syndrome-like | gga-miR-1782_L+2R-1 |
| WASL | Wiskott-Aldrich syndrome-like | oan-miR-363-3p_R+1 |
| TECTB | tectorin beta | PC-3p-457530_9 |
| TECTB | tectorin beta | PC-3p-52432_89 |
| LMOD2 | leiomodin 2 (cardiac) | chi-miR-16b-5p_R+1 |
| LMOD2 | leiomodin 2 (cardiac) | gga-miR-16-5p |
| DCUN1D1 | DCN1, defective in cullin neddylation 1, domain containing 1 (S. cerevisiae) | PC-5p-113875_40 |
| DCUN1D1 | DCN1, defective in cullin neddylation 1, domain containing 1 (S. cerevisiae) | PC-5p-92816_49 |
| DCUN1D1 | DCN1, defective in cullin neddylation 1, domain containing 1 (S. cerevisiae) | chi-miR-16b-5p_R+1 |
| DCUN1D1 | DCN1, defective in cullin neddylation 1, domain containing 1 (S. cerevisiae) | gga-let-7g-3p_1ss22CT |
| DCUN1D1 | DCN1, defective in cullin neddylation 1, domain containing 1 (S. cerevisiae) | gga-miR-16-5p |
| DCUN1D1 | DCN1, defective in cullin neddylation 1, domain containing 1 (S. cerevisiae) | gga-miR-1a-3p_R+1_1ss2GT |
| DTD1 | D-tyrosyl-tRNA deacylase 1 homolog (S. cerevisiae) | gga-miR-33-3p_L+1R+1 |
| LRSAM1 | leucine rich repeat and sterile alpha motif containing 1 | PC-3p-52432_89 |
| NDUFA5 | NADH dehydrogenase (ubiquinone) 1 alpha subcomplex, 5, 13kDa | PC-5p-92816_49 |
| SAMD13 | sterile alpha motif domain containing 13 | PC-3p-457530_9 |
| SAMD13 | sterile alpha motif domain containing 13 | PC-5p-582426_7 |
| SAMD13 | sterile alpha motif domain containing 13 | PC-5p-97606_47 |
| SAMD13 | sterile alpha motif domain containing 13 | gga-let-7g-3p_1ss22CT |
| ATP6AP1 | ATPase, H+ transporting, lysosomal accessory protein 1 | gga-miR-1782_L+2R-1 |
| VPS54 | vacuolar protein sorting 54 homolog (S. cerevisiae) | PC-5p-233284_18 |
| VPS54 | vacuolar protein sorting 54 homolog (S. cerevisiae) | aca-miR-363-3p_R+1 |
| VPS54 | vacuolar protein sorting 54 homolog (S. cerevisiae) | oan-miR-363-3p_R+1 |
| PRPF4 | PRP4 pre-mRNA processing factor 4 homolog (yeast) | PC-3p-52432_89 |
| PRPF4 | PRP4 pre-mRNA processing factor 4 homolog (yeast) | PC-5p-97606_47 |
| WDR31 | WD repeat domain 31 | gga-miR-1677-3p_L+1 |
| WDR31 | WD repeat domain 31 | tgu-miR-2970-5p_1ss21GT |
| DNAJC10 | DnaJ (Hsp40) homolog, subfamily C, member 10 | gga-miR-1a-3p_R+1_1ss2GT |
| FXR1 | fragile X mental retardation, autosomal homolog 1 | aca-miR-363-3p_R+1 |
| FXR1 | fragile X mental retardation, autosomal homolog 1 | gga-let-7g-3p_1ss22CT |
| FXR1 | fragile X mental retardation, autosomal homolog 1 | oan-miR-363-3p_R+1 |
| COMMD5 | COMM domain containing 5 | hsa-miR-483-3p_L-1R+2 |
| LPHN2 | latrophilin 2 | PC-5p-97606_47 |
| TTC14 | tetratricopeptide repeat domain 14 | gga-miR-7468-3p_L+3 |
| GIPC2 | GIPC PDZ domain containing family, member 2 | gga-miR-33-3p_L+1R+1 |
| CERKL | ceramide kinase-like | tgu-miR-2970-5p_1ss21GT |
| B3GNT2 | UDP-GlcNAc:betaGal beta-1,3-N-acetylglucosaminyltransferase 2 | PC-5p-582426_7 |
| PPP1R21 | protein phosphatase 1, regulatory subunit 21 | chi-miR-16b-5p_R+1 |
| PPP1R21 | protein phosphatase 1, regulatory subunit 21 | gga-miR-16-5p |
| PPP1R21 | protein phosphatase 1, regulatory subunit 21 | gga-miR-33-3p_L+1R+1 |
| SPTBN5 | spectrin, beta, non-erythrocytic 5 | PC-5p-233284_18 |
| SPTBN5 | spectrin, beta, non-erythrocytic 5 | PC-5p-37716_121 |
| NOXA1 | NADPH oxidase activator 1 | gga-miR-34b-5p_L-1R+1 |
| EPCAM | epithelial cell adhesion molecule | chi-miR-16b-5p_R+1 |
| EPCAM | epithelial cell adhesion molecule | gga-miR-16-5p |
| NRK | Nik related kinase | PC-5p-71982_65 |
| ADAM22 | ADAM metallopeptidase domain 22 | gga-miR-30a-5p_R+2_1ss13CT |
| ADAM22 | ADAM metallopeptidase domain 22 | gga-miR-30e-5p_R+5 |
| ITGA4 | integrin, alpha 4 (antigen CD49D, alpha 4 subunit of VLA-4 receptor) | PC-5p-233284_18 |
| ITGA4 | integrin, alpha 4 (antigen CD49D, alpha 4 subunit of VLA-4 receptor) | gga-miR-1a-3p_R+1_1ss2GT |
| C4HXORF57 | chromosome 4 open reading frame, human CXorf57 | tgu-miR-2970-5p_1ss21GT |
| VPS39 | vacuolar protein sorting 39 homolog (S. cerevisiae) | gga-miR-1677-3p_L+1 |
| AK5 | adenylate kinase 5 | PC-5p-582426_7 |
| AK5 | adenylate kinase 5 | PC-5p-92816_49 |
| AK5 | adenylate kinase 5 | gga-miR-7468-3p_L+3 |
| PIGK | phosphatidylinositol glycan anchor biosynthesis, class K | PC-5p-582426_7 |
| PIGK | phosphatidylinositol glycan anchor biosynthesis, class K | aca-miR-363-3p_R+1 |
| PIGK | phosphatidylinositol glycan anchor biosynthesis, class K | oan-miR-363-3p_R+1 |
| TMEM87A | transmembrane protein 87A | PC-5p-37716_121 |
| TMEM87A | transmembrane protein 87A | PC-5p-97606_47 |
| TMEM87A | transmembrane protein 87A | aca-miR-18a-5p_R-2 |
| TMEM87A | transmembrane protein 87A | aca-miR-363-3p_R+1 |
| TMEM87A | transmembrane protein 87A | hsa-miR-483-3p_L-1R+2 |
| TMEM87A | transmembrane protein 87A | oan-miR-363-3p_R+1 |
| JAG1 | jagged 1 | gga-miR-1677-3p_L+1 |
| SESTD1 | SEC14 and spectrin domains 1 | gga-let-7g-3p_1ss22CT |
| PIF1 | PIF1 5'-to-3' DNA helicase homolog (S. cerevisiae) | PC-5p-92816_49 |
| PIF1 | PIF1 5'-to-3' DNA helicase homolog (S. cerevisiae) | tgu-miR-2970-5p_1ss21GT |
| GANC | glucosidase, alpha; neutral C | PC-5p-71982_65 |
| SPTLC3 | serine palmitoyltransferase, long chain base subunit 3 | gga-miR-33-3p_L+1R+1 |
| TBC1D8B | TBC1 domain family, member 8B (with GRAM domain) | gga-miR-30a-5p_R+2_1ss13CT |
| TBC1D8B | TBC1 domain family, member 8B (with GRAM domain) | gga-miR-30e-5p_R+5 |
| YPEL5 | yippee-like 5 (Drosophila) | PC-5p-97606_47 |
| YPEL5 | yippee-like 5 (Drosophila) | gga-miR-1782_L+2R-1 |
| LBH | limb bud and heart development homolog (mouse) | PC-5p-113875_40 |
| LBH | limb bud and heart development homolog (mouse) | PC-5p-97606_47 |
| LCLAT1 | lysocardiolipin acyltransferase 1 | PC-5p-97606_47 |
| LCLAT1 | lysocardiolipin acyltransferase 1 | gga-miR-1805-5p_L-1 |
| TASP1 | taspase, threonine aspartase, 1 | aca-miR-363-3p_R+1 |
| TASP1 | taspase, threonine aspartase, 1 | chi-miR-16b-5p_R+1 |
| TASP1 | taspase, threonine aspartase, 1 | gga-miR-16-5p |
| TASP1 | taspase, threonine aspartase, 1 | oan-miR-363-3p_R+1 |
| ENTPD2 | ectonucleoside triphosphate diphosphohydrolase 2 | PC-5p-113875_40 |
| ENTPD2 | ectonucleoside triphosphate diphosphohydrolase 2 | chi-miR-16b-5p_R+1 |
| ENTPD2 | ectonucleoside triphosphate diphosphohydrolase 2 | gga-miR-16-5p |
| VMA21 | VMA21 vacuolar H+-ATPase homolog (S. cerevisiae) | PC-5p-233284_18 |
| ZFP106 | zinc finger protein 106 homolog (mouse) | PC-3p-173245_25 |
| ZFP106 | zinc finger protein 106 homolog (mouse) | gga-miR-30a-5p_R+2_1ss13CT |
| ZFP106 | zinc finger protein 106 homolog (mouse) | gga-miR-30e-5p_R+5 |
| MTMR1 | myotubularin related protein 1 | PC-5p-233284_18 |
| MTMR1 | myotubularin related protein 1 | gga-miR-1782_L+2R-1 |
| EHD3 | EH-domain containing 3 | PC-3p-338480_12 |
| EHD3 | EH-domain containing 3 | PC-5p-71982_65 |
| EHD3 | EH-domain containing 3 | gga-miR-30a-5p_R+2_1ss13CT |
| EHD3 | EH-domain containing 3 | gga-miR-30e-5p_R+5 |
| EHD3 | EH-domain containing 3 | tgu-miR-2970-5p_1ss21GT |
| FSHR | follicle stimulating hormone receptor | PC-5p-71982_65 |
| FSHR | follicle stimulating hormone receptor | PC-5p-97606_47 |
| DPP7 | dipeptidyl-peptidase 7 | gga-miR-1677-3p_L+1 |
| NRXN1 | neurexin 1 | gga-miR-1805-5p_L-1 |
| NRXN1 | neurexin 1 | gga-miR-1a-3p_R+1_1ss2GT |
| NRXN1 | neurexin 1 | gga-miR-7468-3p_L+3 |
| NCSTN | nicastrin | PC-5p-97606_47 |
| CDAN1 | codanin 1 | aca-miR-18a-5p_R-2 |
| CDC42BPA | CDC42 binding protein kinase alpha (DMPK-like) | PC-5p-92816_49 |
| CDC42BPA | CDC42 binding protein kinase alpha (DMPK-like) | gga-let-7g-3p_1ss22CT |
| GFRA1 | GDNF family receptor alpha 1 | PC-3p-173245_25 |
| GFRA1 | GDNF family receptor alpha 1 | PC-3p-457530_9 |
| GFRA1 | GDNF family receptor alpha 1 | gga-miR-30a-5p_R+2_1ss13CT |
| GFRA1 | GDNF family receptor alpha 1 | gga-miR-30e-5p_R+5 |
| CRDS2 | photoreceptor outer segment membrane glycoprotein | PC-3p-457530_9 |
| CRDS2 | photoreceptor outer segment membrane glycoprotein | gga-miR-30a-5p_R+2_1ss13CT |
| CRDS2 | photoreceptor outer segment membrane glycoprotein | gga-miR-30e-5p_R+5 |
| FNDC3B | fibronectin type III domain containing 3B | aca-miR-18a-5p_R-2 |
| KIAA0922 | KIAA0922 | PC-3p-173245_25 |
| LIN9 | lin-9 homolog (C. elegans) | PC-5p-37716_121 |
| LIN9 | lin-9 homolog (C. elegans) | gga-miR-33-3p_L+1R+1 |
| AMDHD2 | amidohydrolase domain containing 2 | PC-3p-173245_25 |
| AMDHD2 | amidohydrolase domain containing 2 | PC-5p-113875_40 |
| SFRP2 | secreted frizzled-related protein 2 | gga-let-7g-3p_1ss22CT |
| ACBD3 | acyl-CoA binding domain containing 3 | PC-3p-338480_12 |
| ACBD3 | acyl-CoA binding domain containing 3 | chi-miR-16b-5p_R+1 |
| ACBD3 | acyl-CoA binding domain containing 3 | gga-let-7g-3p_1ss22CT |
| ACBD3 | acyl-CoA binding domain containing 3 | gga-miR-16-5p |
| ACBD3 | acyl-CoA binding domain containing 3 | gga-miR-34b-5p_L-1R+1 |
| C3H1ORF55 | chromosome 3 open reading frame, human C1orf55 | chi-miR-16b-5p_R+1 |
| C3H1ORF55 | chromosome 3 open reading frame, human C1orf55 | gga-miR-16-5p |
| C3H1ORF55 | chromosome 3 open reading frame, human C1orf55 | gga-miR-1782_L+2R-1 |
| LOC431656 | probable phospholipid-transporting ATPase FetA-like | PC-5p-113875_40 |
| MTX2 | metaxin 2 | tgu-miR-2970-5p_1ss21GT |
| KIAA1715 | KIAA1715 | PC-5p-97606_47 |
| KIAA1715 | KIAA1715 | gga-miR-1805-5p_L-1 |
| ATF2 | activating transcription factor 2 | PC-5p-233284_18 |
| CHRNA1 | cholinergic receptor, nicotinic, alpha 1 (muscle) | gga-miR-1677-3p_L+1 |
| CHRNA1 | cholinergic receptor, nicotinic, alpha 1 (muscle) | gga-miR-33-3p_L+1R+1 |
| LBR | lamin B receptor | tgu-miR-2970-5p_1ss21GT |
| WDR26 | WD repeat domain 26 | gga-miR-1a-3p_R+1_1ss2GT |
| C6H10ORF46 | chromosome 6 open reading frame, human C10orf46 | PC-3p-173245_25 |
| C6H10ORF46 | chromosome 6 open reading frame, human C10orf46 | PC-3p-52432_89 |
| C6H10ORF46 | chromosome 6 open reading frame, human C10orf46 | chi-miR-16b-5p_R+1 |
| C6H10ORF46 | chromosome 6 open reading frame, human C10orf46 | gga-miR-16-5p |
| IFT140 | intraflagellar transport 140 homolog (Chlamydomonas) | PC-5p-71982_65 |
| IFT140 | intraflagellar transport 140 homolog (Chlamydomonas) | PC-5p-97606_47 |
| NVL | nuclear VCP-like | aca-miR-363-3p_R+1 |
| NVL | nuclear VCP-like | oan-miR-363-3p_R+1 |
| AKAP9 | A kinase (PRKA) anchor protein (yotiao) 9 | PC-5p-97606_47 |
| OLA1 | Obg-like ATPase 1 | chi-miR-16b-5p_R+1 |
| OLA1 | Obg-like ATPase 1 | gga-miR-16-5p |
| SP3 | Sp3 transcription factor | gga-miR-33-3p_L+1R+1 |
| DEGS1 | degenerative spermatocyte homolog 1, lipid desaturase (Drosophila) | gga-miR-34b-5p_L-1R+1 |
| RBM25 | RNA binding motif protein 25 | gga-let-7g-3p_1ss22CT |
| FAM45A | family with sequence similarity 45, member A | PC-3p-52432_89 |
| CRAMP1L | Crm, cramped-like (Drosophila) | PC-5p-71982_65 |
| CRAMP1L | Crm, cramped-like (Drosophila) | aca-miR-363-3p_R+1 |
| CRAMP1L | Crm, cramped-like (Drosophila) | oan-miR-363-3p_R+1 |
| DCAF4 | DDB1 and CUL4 associated factor 4 | chi-miR-16b-5p_R+1 |
| DCAF4 | DDB1 and CUL4 associated factor 4 | gga-miR-16-5p |
| DCAF4 | DDB1 and CUL4 associated factor 4 | gga-miR-1782_L+2R-1 |
| DCAF4 | DDB1 and CUL4 associated factor 4 | gga-miR-30a-5p_R+2_1ss13CT |
| DCAF4 | DDB1 and CUL4 associated factor 4 | gga-miR-30e-5p_R+5 |
| DPF3 | D4, zinc and double PHD fingers, family 3 | PC-5p-233284_18 |
| DPF3 | D4, zinc and double PHD fingers, family 3 | chi-miR-16b-5p_R+1 |
| DPF3 | D4, zinc and double PHD fingers, family 3 | gga-miR-16-5p |
| DPF3 | D4, zinc and double PHD fingers, family 3 | gga-miR-1677-3p_L+1 |
| DPF3 | D4, zinc and double PHD fingers, family 3 | gga-miR-1a-3p_R+1_1ss2GT |
| DPF3 | D4, zinc and double PHD fingers, family 3 | gga-miR-34b-5p_L-1R+1 |
| GUCY1A3 | guanylate cyclase 1, soluble, alpha 3 | PC-5p-233284_18 |
| SKIL | SKI-like oncogene | gga-miR-1677-3p_L+1 |
| SKIL | SKI-like oncogene | tgu-miR-2970-5p_1ss21GT |
| GUCY1B3 | guanylate cyclase 1, soluble, beta 3 | aca-miR-363-3p_R+1 |
| GUCY1B3 | guanylate cyclase 1, soluble, beta 3 | gga-miR-1782_L+2R-1 |
| GUCY1B3 | guanylate cyclase 1, soluble, beta 3 | oan-miR-363-3p_R+1 |
| PRKCI | protein kinase C, iota | PC-3p-52432_89 |
| PRKCI | protein kinase C, iota | PC-5p-97606_47 |
| PRKCI | protein kinase C, iota | aca-miR-363-3p_R+1 |
| PRKCI | protein kinase C, iota | gga-let-7g-3p_1ss22CT |
| PRKCI | protein kinase C, iota | gga-miR-1782_L+2R-1 |
| PRKCI | protein kinase C, iota | gga-miR-33-3p_L+1R+1 |
| PRKCI | protein kinase C, iota | gga-miR-34b-5p_L-1R+1 |
| PRKCI | protein kinase C, iota | oan-miR-363-3p_R+1 |
| PRKCI | protein kinase C, iota | tgu-miR-2970-5p_1ss21GT |
| RGS7 | regulator of G-protein signaling 7 | PC-5p-233284_18 |
| CTSO | cathepsin O | PC-3p-173245_25 |
| CTSO | cathepsin O | PC-5p-582426_7 |
| CTSO | cathepsin O | PC-5p-97606_47 |
| CTSO | cathepsin O | gga-miR-1782_L+2R-1 |
| PDGFC | platelet derived growth factor C | PC-5p-97606_47 |
| SEC62 | SEC62 homolog (S. cerevisiae) | gga-let-7g-3p_1ss22CT |
| KRIT1 | KRIT1, ankyrin repeat containing | aca-miR-363-3p_R+1 |
| KRIT1 | KRIT1, ankyrin repeat containing | oan-miR-363-3p_R+1 |
| CAPZA2 | capping protein (actin filament) muscle Z-line, alpha 2 | PC-5p-113875_40 |
| CAPZA2 | capping protein (actin filament) muscle Z-line, alpha 2 | chi-miR-16b-5p_R+1 |
| CAPZA2 | capping protein (actin filament) muscle Z-line, alpha 2 | gga-miR-16-5p |
| CAPZA2 | capping protein (actin filament) muscle Z-line, alpha 2 | gga-miR-1782_L+2R-1 |
| LOC423256 | desumoylating isopeptidase 2-like | PC-5p-37716_121 |
| LOC423256 | desumoylating isopeptidase 2-like | chi-miR-16b-5p_R+1 |
| LOC423256 | desumoylating isopeptidase 2-like | gga-miR-16-5p |
| LOC423256 | desumoylating isopeptidase 2-like | hsa-miR-483-3p_L-1R+2 |
| CAV2 | caveolin 2 | aca-miR-363-3p_R+1 |
| CAV2 | caveolin 2 | chi-miR-16b-5p_R+1 |
| CAV2 | caveolin 2 | gga-miR-16-5p |
| CAV2 | caveolin 2 | gga-miR-7468-3p_L+3 |
| CAV2 | caveolin 2 | oan-miR-363-3p_R+1 |
| SMOC1 | SPARC related modular calcium binding 1 | PC-3p-52432_89 |
| RXFP1 | relaxin/insulin-like family peptide receptor 1 | chi-miR-16b-5p_R+1 |
| RXFP1 | relaxin/insulin-like family peptide receptor 1 | gga-miR-16-5p |
| RXFP1 | relaxin/insulin-like family peptide receptor 1 | gga-miR-1677-3p_L+1 |
| RXFP1 | relaxin/insulin-like family peptide receptor 1 | gga-miR-1782_L+2R-1 |
| BAG3 | BCL2-associated athanogene 3 | PC-3p-457530_9 |
| BAG3 | BCL2-associated athanogene 3 | PC-3p-52432_89 |
| BAG3 | BCL2-associated athanogene 3 | gga-let-7g-3p_1ss22CT |
| MIA3 | melanoma inhibitory activity family, member 3 | aca-miR-363-3p_R+1 |
| MIA3 | melanoma inhibitory activity family, member 3 | oan-miR-363-3p_R+1 |
| MECOM | MDS1 and EVI1 complex locus | PC-5p-71982_65 |
| SLC39A9 | solute carrier family 39 (zinc transporter), member 9 | PC-3p-173245_25 |
| SLC39A9 | solute carrier family 39 (zinc transporter), member 9 | PC-5p-37716_121 |
| SLC39A9 | solute carrier family 39 (zinc transporter), member 9 | aca-miR-425-3p_L-2R+1_1ss3AT |
| SLC39A9 | solute carrier family 39 (zinc transporter), member 9 | chi-miR-16b-5p_R+1 |
| SLC39A9 | solute carrier family 39 (zinc transporter), member 9 | gga-miR-16-5p |
| SLC39A9 | solute carrier family 39 (zinc transporter), member 9 | hsa-miR-483-3p_L-1R+2 |
| ETFDH | electron-transferring-flavoprotein dehydrogenase | PC-5p-113875_40 |
| ETFDH | electron-transferring-flavoprotein dehydrogenase | gga-miR-1782_L+2R-1 |
| ETFDH | electron-transferring-flavoprotein dehydrogenase | gga-miR-33-3p_L+1R+1 |
| ETFDH | electron-transferring-flavoprotein dehydrogenase | gga-miR-34b-5p_L-1R+1 |
| DUSP10 | dual specificity phosphatase 10 | PC-3p-457530_9 |
| DUSP10 | dual specificity phosphatase 10 | PC-5p-71982_65 |
| DUSP10 | dual specificity phosphatase 10 | PC-5p-97606_47 |
| DUSP10 | dual specificity phosphatase 10 | aca-miR-363-3p_R+1 |
| DUSP10 | dual specificity phosphatase 10 | hsa-miR-483-3p_L-1R+2 |
| DUSP10 | dual specificity phosphatase 10 | oan-miR-363-3p_R+1 |
| PPID | peptidylprolyl isomerase D | gga-miR-30a-5p_R+2_1ss13CT |
| PPID | peptidylprolyl isomerase D | gga-miR-30e-5p_R+5 |
| LOC424998 | multiple EGF-like-domains 6-like | PC-3p-52432_89 |
| SEC23IP | SEC23 interacting protein | gga-miR-1782_L+2R-1 |
| C2H7ORF64 | chromosome 2 open reading frame, human C7orf64 | PC-3p-457530_9 |
| C2H7ORF64 | chromosome 2 open reading frame, human C7orf64 | PC-5p-97606_47 |
| C2H7ORF64 | chromosome 2 open reading frame, human C7orf64 | gga-miR-34b-5p_L-1R+1 |
| C2H7ORF64 | chromosome 2 open reading frame, human C7orf64 | gga-miR-7468-3p_L+3 |
| C2H7ORF64 | chromosome 2 open reading frame, human C7orf64 | tgu-miR-2970-5p_1ss21GT |
| SERPINI1 | serpin peptidase inhibitor, clade I (neuroserpin), member 1 | PC-5p-113875_40 |
| SERPINI1 | serpin peptidase inhibitor, clade I (neuroserpin), member 1 | chi-miR-16b-5p_R+1 |
| SERPINI1 | serpin peptidase inhibitor, clade I (neuroserpin), member 1 | gga-miR-16-5p |
| SERPINI1 | serpin peptidase inhibitor, clade I (neuroserpin), member 1 | gga-miR-30a-5p_R+2_1ss13CT |
| SERPINI1 | serpin peptidase inhibitor, clade I (neuroserpin), member 1 | gga-miR-30e-5p_R+5 |
| SERPINI1 | serpin peptidase inhibitor, clade I (neuroserpin), member 1 | gga-miR-7468-3p_L+3 |
| PDCD10 | programmed cell death 10 | tgu-miR-2970-5p_1ss21GT |
| TMEM229B | transmembrane protein 229B | PC-5p-113875_40 |
| TMEM229B | transmembrane protein 229B | aca-miR-363-3p_R+1 |
| TMEM229B | transmembrane protein 229B | oan-miR-363-3p_R+1 |
| THAP5 | THAP domain containing 5 | gga-miR-34b-5p_L-1R+1 |
| THAP5 | THAP domain containing 5 | tgu-miR-2970-5p_1ss21GT |
| CCDC132 | coiled-coil domain containing 132 | PC-5p-113875_40 |
| CCDC132 | coiled-coil domain containing 132 | PC-5p-92816_49 |
| CCDC132 | coiled-coil domain containing 132 | gga-let-7g-3p_1ss22CT |
| RAB3GAP2 | RAB3 GTPase activating protein subunit 2 (non-catalytic) | chi-miR-16b-5p_R+1 |
| RAB3GAP2 | RAB3 GTPase activating protein subunit 2 (non-catalytic) | gga-miR-16-5p |
| PLEKHH1 | pleckstrin homology domain containing, family H (with MyTH4 domain) member 1 | PC-3p-457530_9 |
| PLEKHH1 | pleckstrin homology domain containing, family H (with MyTH4 domain) member 1 | PC-5p-97606_47 |
| PLEKHH1 | pleckstrin homology domain containing, family H (with MyTH4 domain) member 1 | gga-miR-30a-5p_R+2_1ss13CT |
| PLEKHH1 | pleckstrin homology domain containing, family H (with MyTH4 domain) member 1 | gga-miR-30e-5p_R+5 |
| VTI1B | vesicle transport through interaction with t-SNAREs homolog 1B (yeast) | hsa-miR-483-3p_L-1R+2 |
| GXYLT1 | glucoside xylosyltransferase 1 | PC-3p-457530_9 |
| GXYLT1 | glucoside xylosyltransferase 1 | PC-5p-97606_47 |
| GXYLT1 | glucoside xylosyltransferase 1 | gga-miR-30a-5p_R+2_1ss13CT |
| GXYLT1 | glucoside xylosyltransferase 1 | gga-miR-30e-5p_R+5 |
| NMD3 | NMD3 homolog (S. cerevisiae) | gga-miR-1805-5p_L-1 |
| NMD3 | NMD3 homolog (S. cerevisiae) | gga-miR-7468-3p_L+3 |
| YAF2 | YY1 associated factor 2 | PC-3p-457530_9 |
| YAF2 | YY1 associated factor 2 | PC-5p-71982_65 |
| YAF2 | YY1 associated factor 2 | gga-miR-1782_L+2R-1 |
| YAF2 | YY1 associated factor 2 | gga-miR-1a-3p_R+1_1ss2GT |
| YAF2 | YY1 associated factor 2 | gga-miR-30a-5p_R+2_1ss13CT |
| YAF2 | YY1 associated factor 2 | gga-miR-30e-5p_R+5 |
| SLC25A13 | solute carrier family 25, member 13 (citrin) | tgu-miR-2970-5p_1ss21GT |
| SLC25A12 | solute carrier family 25 (mitochondrial carrier, Aralar), member 12 | aca-miR-363-3p_R+1 |
| SLC25A12 | solute carrier family 25 (mitochondrial carrier, Aralar), member 12 | chi-miR-16b-5p_R+1 |
| SLC25A12 | solute carrier family 25 (mitochondrial carrier, Aralar), member 12 | gga-miR-16-5p |
| SLC25A12 | solute carrier family 25 (mitochondrial carrier, Aralar), member 12 | gga-miR-1a-3p_R+1_1ss2GT |
| SLC25A12 | solute carrier family 25 (mitochondrial carrier, Aralar), member 12 | oan-miR-363-3p_R+1 |
| KLHL2 | kelch-like 2, Mayven (Drosophila) | aca-miR-18a-5p_R-2 |
| KLHL2 | kelch-like 2, Mayven (Drosophila) | tgu-miR-2970-5p_1ss21GT |
| DCAF17 | DDB1 and CUL4 associated factor 17 | gga-miR-7468-3p_L+3 |
| MSMO1 | methylsterol monooxygenase 1 | aca-miR-363-3p_R+1 |
| MSMO1 | methylsterol monooxygenase 1 | oan-miR-363-3p_R+1 |
| METTL8 | methyltransferase like 8 | PC-3p-338480_12 |
| METTL8 | methyltransferase like 8 | aca-miR-363-3p_R+1 |
| METTL8 | methyltransferase like 8 | gga-miR-1677-3p_L+1 |
| METTL8 | methyltransferase like 8 | oan-miR-363-3p_R+1 |
| IARS2 | isoleucyl-tRNA synthetase 2, mitochondrial | PC-5p-71982_65 |
| TLK1 | tousled-like kinase 1 | gga-miR-33-3p_L+1R+1 |
| TLK1 | tousled-like kinase 1 | gga-miR-34b-5p_L-1R+1 |
| ATP6V1D | ATPase, H+ transporting, lysosomal 34kDa, V1 subunit D | PC-5p-97606_47 |
| PUS7L | pseudouridylate synthase 7 homolog (S. cerevisiae)-like | gga-miR-1782_L+2R-1 |
| SCHIP1 | schwannomin interacting protein 1 | hsa-miR-483-3p_L-1R+2 |
| GORASP2 | golgi reassembly stacking protein 2, 55kDa | gga-miR-34b-5p_L-1R+1 |
| IRAK4 | interleukin-1 receptor-associated kinase 4 | PC-5p-113875_40 |
| IRAK4 | interleukin-1 receptor-associated kinase 4 | PC-5p-71982_65 |
| GAD1 | glutamate decarboxylase 1 (brain, 67kDa) | PC-3p-173245_25 |
| GAD1 | glutamate decarboxylase 1 (brain, 67kDa) | aca-miR-18a-5p_R-2 |
| GAD1 | glutamate decarboxylase 1 (brain, 67kDa) | tgu-miR-2970-5p_1ss21GT |
| SRP14 | signal recognition particle 14kDa (homologous Alu RNA binding protein) | gga-miR-30a-5p_R+2_1ss13CT |
| SRP14 | signal recognition particle 14kDa (homologous Alu RNA binding protein) | gga-miR-30e-5p_R+5 |
| MYO3B | myosin IIIB | chi-miR-16b-5p_R+1 |
| MYO3B | myosin IIIB | gga-miR-16-5p |
| LOC417800 | prolactin-like protein | PC-5p-92816_49 |
| ADAM5P | ADAM metallopeptidase domain 5, pseudogene | PC-3p-173245_25 |
| ZNF650 | zinc finger protein 650 | PC-5p-113875_40 |
| ZNF650 | zinc finger protein 650 | chi-miR-16b-5p_R+1 |
| ZNF650 | zinc finger protein 650 | gga-miR-16-5p |
| RRP15 | ribosomal RNA processing 15 homolog (S. cerevisiae) | PC-3p-457530_9 |
| GPATCH2 | G patch domain containing 2 | PC-5p-92816_49 |
| GPATCH2 | G patch domain containing 2 | PC-5p-97606_47 |
| GPATCH2 | G patch domain containing 2 | gga-miR-1677-3p_L+1 |
| GPATCH2 | G patch domain containing 2 | gga-miR-1a-3p_R+1_1ss2GT |
| C5H15ORF29 | chromosome 5 open reading frame, human C15orf29 | PC-3p-52432_89 |
| C5H15ORF29 | chromosome 5 open reading frame, human C15orf29 | PC-5p-233284_18 |
| C5H15ORF29 | chromosome 5 open reading frame, human C15orf29 | PC-5p-71982_65 |
| C5H15ORF29 | chromosome 5 open reading frame, human C15orf29 | PC-5p-97606_47 |
| C5H15ORF29 | chromosome 5 open reading frame, human C15orf29 | gga-miR-1677-3p_L+1 |
| C5H15ORF29 | chromosome 5 open reading frame, human C15orf29 | gga-miR-30a-5p_R+2_1ss13CT |
| C5H15ORF29 | chromosome 5 open reading frame, human C15orf29 | gga-miR-30e-5p_R+5 |
| GFM1 | G elongation factor, mitochondrial 1 | chi-miR-16b-5p_R+1 |
| GFM1 | G elongation factor, mitochondrial 1 | gga-miR-16-5p |
| C5H15ORF24 | chromosome 5 open reading frame, human C15orf24 | gga-miR-1782_L+2R-1 |
| COL1A2 | collagen, type I, alpha 2 | aca-miR-363-3p_R+1 |
| COL1A2 | collagen, type I, alpha 2 | oan-miR-363-3p_R+1 |
| AVEN | apoptosis, caspase activation inhibitor | gga-miR-1677-3p_L+1 |
| PALLD | palladin, cytoskeletal associated protein | PC-5p-113875_40 |
| ACADSB | acyl-CoA dehydrogenase, short/branched chain | PC-3p-173245_25 |
| ACADSB | acyl-CoA dehydrogenase, short/branched chain | gga-miR-1782_L+2R-1 |
| ACADSB | acyl-CoA dehydrogenase, short/branched chain | gga-miR-1a-3p_R+1_1ss2GT |
| ACADSB | acyl-CoA dehydrogenase, short/branched chain | tgu-miR-2970-5p_1ss21GT |
| ARID2 | AT rich interactive domain 2 (ARID, RFX-like) | PC-5p-71982_65 |
| CLCN3 | chloride channel, voltage-sensitive 3 | PC-5p-97606_47 |
| CLCN3 | chloride channel, voltage-sensitive 3 | chi-miR-16b-5p_R+1 |
| CLCN3 | chloride channel, voltage-sensitive 3 | gga-miR-16-5p |
| METTL5 | methyltransferase like 5 | PC-5p-97606_47 |
| SSB | Sjogren syndrome antigen B (autoantigen La) | gga-let-7g-3p_1ss22CT |
| VEPH1 | ventricular zone expressed PH domain homolog 1 (zebrafish) | PC-3p-52432_89 |
| GPR26 | G protein-coupled receptor 26 | PC-5p-71982_65 |
| GPR26 | G protein-coupled receptor 26 | aca-miR-363-3p_R+1 |
| GPR26 | G protein-coupled receptor 26 | oan-miR-363-3p_R+1 |
| CCNL1 | cyclin L1 | gga-let-7g-3p_1ss22CT |
| LOC426599 | tetratricopeptide repeat protein 38-like | PC-3p-52432_89 |
| MUM1 | melanoma associated antigen (mutated) 1 | PC-3p-52432_89 |
| DYNC1I1 | dynein, cytoplasmic 1, intermediate chain 1 | PC-5p-97606_47 |
| DYNC1I1 | dynein, cytoplasmic 1, intermediate chain 1 | chi-miR-16b-5p_R+1 |
| DYNC1I1 | dynein, cytoplasmic 1, intermediate chain 1 | gga-miR-16-5p |
| SLC38A4 | solute carrier family 38, member 4 | PC-5p-113875_40 |
| RPAP3 | RNA polymerase II associated protein 3 | gga-miR-1a-3p_R+1_1ss2GT |
| ARSA | arylsulfatase A | PC-5p-113875_40 |
| ASNS | asparagine synthetase (glutamine-hydrolyzing) | aca-miR-18a-5p_R-2 |
| C1GALT1 | core 1 synthase, glycoprotein-N-acetylgalactosamine 3-beta-galactosyltransferase, 1 | PC-3p-457530_9 |
| C1GALT1 | core 1 synthase, glycoprotein-N-acetylgalactosamine 3-beta-galactosyltransferase, 1 | PC-5p-582426_7 |
| C1GALT1 | core 1 synthase, glycoprotein-N-acetylgalactosamine 3-beta-galactosyltransferase, 1 | chi-miR-16b-5p_R+1 |
| C1GALT1 | core 1 synthase, glycoprotein-N-acetylgalactosamine 3-beta-galactosyltransferase, 1 | gga-miR-16-5p |
| C1GALT1 | core 1 synthase, glycoprotein-N-acetylgalactosamine 3-beta-galactosyltransferase, 1 | gga-miR-1782_L+2R-1 |
| C1GALT1 | core 1 synthase, glycoprotein-N-acetylgalactosamine 3-beta-galactosyltransferase, 1 | tgu-miR-2970-5p_1ss21GT |
| HCLS1 | hematopoietic cell-specific Lyn substrate 1 | gga-miR-7468-3p_L+3 |
| DOCK1 | dedicator of cytokinesis 1 | gga-miR-30a-5p_R+2_1ss13CT |
| DOCK1 | dedicator of cytokinesis 1 | gga-miR-30e-5p_R+5 |
| PTPN14 | protein tyrosine phosphatase, non-receptor type 14 | PC-3p-457530_9 |
| PROX1 | prospero homeobox 1 | PC-5p-97606_47 |
| PROX1 | prospero homeobox 1 | gga-miR-1805-5p_L-1 |
| LOC422442 | uncharacterized LOC422442 | chi-miR-16b-5p_R+1 |
| LOC422442 | uncharacterized LOC422442 | gga-let-7g-3p_1ss22CT |
| LOC422442 | uncharacterized LOC422442 | gga-miR-16-5p |
| LOC422442 | uncharacterized LOC422442 | gga-miR-1a-3p_R+1_1ss2GT |
| C5H15ORF41 | chromosome 5 open reading frame, human C15orf41 | PC-3p-173245_25 |
| C5H15ORF41 | chromosome 5 open reading frame, human C15orf41 | PC-5p-97606_47 |
| C5H15ORF41 | chromosome 5 open reading frame, human C15orf41 | hsa-miR-483-3p_L-1R+2 |
| NENF | neudesin neurotrophic factor | PC-5p-233284_18 |
| TMEM206 | transmembrane protein 206 | PC-5p-71982_65 |
| TMEM206 | transmembrane protein 206 | gga-miR-1782_L+2R-1 |
| TMEM206 | transmembrane protein 206 | tgu-miR-2970-5p_1ss21GT |
| PTPRA | protein tyrosine phosphatase, receptor type, A | PC-3p-457530_9 |
| PTPRA | protein tyrosine phosphatase, receptor type, A | PC-5p-113875_40 |
| SRGAP1 | SLIT-ROBO Rho GTPase activating protein 1 | gga-miR-1677-3p_L+1 |
| CLGN | calmegin | hsa-miR-483-3p_L-1R+2 |
| LOC422448 | mannosyl (alpha-1,3-)-glycoprotein beta-1,4-N-acetylglucosaminyltransferase, isozyme B-like | PC-5p-233284_18 |
| LOC422448 | mannosyl (alpha-1,3-)-glycoprotein beta-1,4-N-acetylglucosaminyltransferase, isozyme B-like | PC-5p-37716_121 |
| LOC422448 | mannosyl (alpha-1,3-)-glycoprotein beta-1,4-N-acetylglucosaminyltransferase, isozyme B-like | gga-miR-1805-5p_L-1 |
| ELMOD2 | ELMO/CED-12 domain containing 2 | gga-miR-30a-5p_R+2_1ss13CT |
| ELMOD2 | ELMO/CED-12 domain containing 2 | gga-miR-30e-5p_R+5 |
| DTL | denticleless E3 ubiquitin protein ligase homolog (Drosophila) | gga-miR-1677-3p_L+1 |
| NKTR | natural killer-tumor recognition sequence | PC-5p-113875_40 |
| NKTR | natural killer-tumor recognition sequence | gga-miR-34b-5p_L-1R+1 |
| NKTR | natural killer-tumor recognition sequence | tgu-miR-2970-5p_1ss21GT |
| RASSF3 | Ras association (RalGDS/AF-6) domain family member 3 | PC-3p-457530_9 |
| LPGAT1 | lysophosphatidylglycerol acyltransferase 1 | aca-miR-363-3p_R+1 |
| LPGAT1 | lysophosphatidylglycerol acyltransferase 1 | gga-miR-7468-3p_L+3 |
| LPGAT1 | lysophosphatidylglycerol acyltransferase 1 | oan-miR-363-3p_R+1 |
| GNS | glucosamine (N-acetyl)-6-sulfatase | aca-miR-363-3p_R+1 |
| GNS | glucosamine (N-acetyl)-6-sulfatase | oan-miR-363-3p_R+1 |
| TRAF5 | TNF receptor-associated factor 5 | PC-5p-233284_18 |
| CAMK1D | calcium/calmodulin-dependent protein kinase ID | gga-let-7g-3p_1ss22CT |
| ZNF330 | zinc finger protein 330 | PC-3p-52432_89 |
| HHAT | hedgehog acyltransferase | PC-5p-113875_40 |
| HHAT | hedgehog acyltransferase | gga-miR-1677-3p_L+1 |
| HELB | helicase (DNA) B | PC-3p-457530_9 |
| DIEXF | digestive organ expansion factor homolog (zebrafish) | PC-5p-97606_47 |
| TRERF1 | transcriptional regulating factor 1 | PC-3p-338480_12 |
| GAB1 | GRB2-associated binding protein 1 | tgu-miR-2970-5p_1ss21GT |
| DYRK2 | dual-specificity tyrosine-(Y)-phosphorylation regulated kinase 2 | gga-miR-1782_L+2R-1 |
| WDR65 | WD repeat domain 65 | gga-miR-1329-3p |
| IFNG | interferon, gamma | PC-5p-113875_40 |
| G2E3 | G2/M-phase specific E3 ubiquitin protein ligase | chi-miR-16b-5p_R+1 |
| G2E3 | G2/M-phase specific E3 ubiquitin protein ligase | gga-miR-16-5p |
| G2E3 | G2/M-phase specific E3 ubiquitin protein ligase | gga-miR-1677-3p_L+1 |
| SMARCA5 | SWI/SNF related, matrix associated, actin dependent regulator of chromatin, subfamily a, member 5 | gga-miR-30a-5p_R+2_1ss13CT |
| SMARCA5 | SWI/SNF related, matrix associated, actin dependent regulator of chromatin, subfamily a, member 5 | gga-miR-30e-5p_R+5 |
| RAP1B | RAP1B, member of RAS oncogene family | gga-miR-1805-5p_L-1 |
| RAP1B | RAP1B, member of RAS oncogene family | gga-miR-30a-5p_R+2_1ss13CT |
| RAP1B | RAP1B, member of RAS oncogene family | gga-miR-30e-5p_R+5 |
| STRN3 | striatin, calmodulin binding protein 3 | PC-3p-173245_25 |
| STRN3 | striatin, calmodulin binding protein 3 | gga-miR-34b-5p_L-1R+1 |
| SPOP | speckle-type POZ protein | chi-miR-16b-5p_R+1 |
| SPOP | speckle-type POZ protein | gga-miR-16-5p |
| SLC35E3 | solute carrier family 35, member E3 | PC-3p-173245_25 |
| MYST2 | MYST histone acetyltransferase 2 | PC-5p-113875_40 |
| MYST2 | MYST histone acetyltransferase 2 | PC-5p-97606_47 |
| MYST2 | MYST histone acetyltransferase 2 | chi-miR-16b-5p_R+1 |
| MYST2 | MYST histone acetyltransferase 2 | gga-miR-16-5p |
| MYST2 | MYST histone acetyltransferase 2 | gga-miR-1805-5p_L-1 |
| CPM | carboxypeptidase M | gga-miR-1782_L+2R-1 |
| HECTD1 | HECT domain containing E3 ubiquitin protein ligase 1 | tgu-miR-2970-5p_1ss21GT |
| DYNC2LI1 | dynein, cytoplasmic 2, light intermediate chain 1 | PC-3p-457530_9 |
| TIE1 | tyrosine kinase with immunoglobulin-like and EGF-like domains 1 | PC-3p-52432_89 |
| CPSF6 | cleavage and polyadenylation specific factor 6, 68kDa | gga-let-7g-3p_1ss22CT |
| ABCE1 | ATP-binding cassette, sub-family E (OABP), member 1 | gga-let-7g-3p_1ss22CT |
| LRPPRC | leucine-rich pentatricopeptide repeat containing | gga-miR-1805-5p_L-1 |
| LRPPRC | leucine-rich pentatricopeptide repeat containing | gga-miR-7468-3p_L+3 |
| CDC20 | cell division cycle 20 homolog (S. cerevisiae) | PC-3p-52432_89 |
| CDC20 | cell division cycle 20 homolog (S. cerevisiae) | gga-let-7g-3p_1ss22CT |
| SLC3A1 | solute carrier family 3 (cystine, dibasic and neutral amino acid transporters, activator of cystine, dibasic and neutral amino acid transport), member 1 | gga-let-7g-3p_1ss22CT |
| SMAD1 | SMAD family member 1 | gga-miR-1782_L+2R-1 |
| PREPL | prolyl endopeptidase-like | gga-let-7g-3p_1ss22CT |
| NUBPL | nucleotide binding protein-like | tgu-miR-2970-5p_1ss21GT |
| ELOVL1 | ELOVL fatty acid elongase 1 | PC-5p-97606_47 |
| MED8 | mediator complex subunit 8 | PC-5p-71982_65 |
| EAPP | E2F-associated phosphoprotein | PC-3p-173245_25 |
| EAPP | E2F-associated phosphoprotein | chi-miR-16b-5p_R+1 |
| EAPP | E2F-associated phosphoprotein | gga-miR-16-5p |
| EDNRA | endothelin receptor type A | gga-miR-1a-3p_R+1_1ss2GT |
| CRIPT | cysteine-rich PDZ-binding protein | PC-5p-92816_49 |
| CRIPT | cysteine-rich PDZ-binding protein | chi-miR-16b-5p_R+1 |
| CRIPT | cysteine-rich PDZ-binding protein | gga-miR-16-5p |
| CRIPT | cysteine-rich PDZ-binding protein | gga-miR-7468-3p_L+3 |
| MCFD2 | multiple coagulation factor deficiency 2 | PC-5p-97606_47 |
| MCFD2 | multiple coagulation factor deficiency 2 | chi-miR-16b-5p_R+1 |
| MCFD2 | multiple coagulation factor deficiency 2 | gga-let-7g-3p_1ss22CT |
| MCFD2 | multiple coagulation factor deficiency 2 | gga-miR-16-5p |
| MCFD2 | multiple coagulation factor deficiency 2 | gga-miR-1782_L+2R-1 |
| MCFD2 | multiple coagulation factor deficiency 2 | gga-miR-1805-5p_L-1 |
| LOC417848 | cathepsin E-A-like | hsa-miR-483-3p_L-1R+2 |
| TMEM184C | transmembrane protein 184C | PC-5p-233284_18 |
| TMEM184C | transmembrane protein 184C | tgu-miR-2970-5p_1ss21GT |
| SNX6 | sorting nexin 6 | PC-5p-97606_47 |
| CFL2 | cofilin 2 (muscle) | gga-miR-33-3p_L+1R+1 |
| BAZ1A | bromodomain adjacent to zinc finger domain, 1A | gga-miR-1a-3p_R+1_1ss2GT |
| BRE | brain and reproductive organ-expressed (TNFRSF1A modulator) | PC-3p-338480_12 |
| BRE | brain and reproductive organ-expressed (TNFRSF1A modulator) | gga-miR-33-3p_L+1R+1 |
| CNOT2 | CCR4-NOT transcription complex, subunit 2 | PC-5p-97606_47 |
| CNOT2 | CCR4-NOT transcription complex, subunit 2 | tgu-miR-2970-5p_1ss21GT |
| FAM177A1 | family with sequence similarity 177, member A1 | PC-3p-338480_12 |
| FAM177A1 | family with sequence similarity 177, member A1 | PC-5p-71982_65 |
| FAM177A1 | family with sequence similarity 177, member A1 | aca-miR-425-3p_L-2R+1_1ss3AT |
| FAM177A1 | family with sequence similarity 177, member A1 | gga-miR-1805-5p_L-1 |
| FAM177A1 | family with sequence similarity 177, member A1 | gga-miR-34b-5p_L-1R+1 |
| FAM177A1 | family with sequence similarity 177, member A1 | tgu-miR-2970-5p_1ss21GT |
| SRP54 | signal recognition particle 54kDa | PC-3p-338480_12 |
| SRP54 | signal recognition particle 54kDa | gga-miR-30a-5p_R+2_1ss13CT |
| SRP54 | signal recognition particle 54kDa | gga-miR-30e-5p_R+5 |
| PPP2R3C | protein phosphatase 2, regulatory subunit B', gamma | gga-miR-33-3p_L+1R+1 |
| PTPRF | protein tyrosine phosphatase, receptor type, F | PC-5p-97606_47 |
| PTPRF | protein tyrosine phosphatase, receptor type, F | gga-let-7g-3p_1ss22CT |
| KIAA0391 | KIAA0391 | PC-5p-92816_49 |
| KIF6 | kinesin family member 6 | PC-5p-92816_49 |
| KIF6 | kinesin family member 6 | gga-miR-1782_L+2R-1 |
| PSMA6 | proteasome (prosome, macropain) subunit, alpha type, 6 | gga-let-7g-3p_1ss22CT |
| HP1BP3 | heterochromatin protein 1, binding protein 3 | gga-let-7g-3p_1ss22CT |
| HP1BP3 | heterochromatin protein 1, binding protein 3 | gga-miR-30a-5p_R+2_1ss13CT |
| HP1BP3 | heterochromatin protein 1, binding protein 3 | gga-miR-30e-5p_R+5 |
| ATP6V0B | ATPase, H+ transporting, lysosomal 21kDa, V0 subunit b | aca-miR-363-3p_R+1 |
| ATP6V0B | ATPase, H+ transporting, lysosomal 21kDa, V0 subunit b | oan-miR-363-3p_R+1 |
| BRMS1L | breast cancer metastasis-suppressor 1-like | gga-miR-33-3p_L+1R+1 |
| BRMS1L | breast cancer metastasis-suppressor 1-like | gga-miR-7468-3p_L+3 |
| MBIP | MAP3K12 binding inhibitory protein 1 | PC-5p-97606_47 |
| MBIP | MAP3K12 binding inhibitory protein 1 | gga-miR-1782_L+2R-1 |
| SCLT1 | sodium channel and clathrin linker 1 | gga-miR-33-3p_L+1R+1 |
| BTBD9 | BTB (POZ) domain containing 9 | PC-5p-233284_18 |
| BTBD9 | BTB (POZ) domain containing 9 | PC-5p-71982_65 |
| BTBD9 | BTB (POZ) domain containing 9 | aca-miR-18a-5p_R-2 |
| BTBD9 | BTB (POZ) domain containing 9 | gga-miR-7468-3p_L+3 |
| C4H8ORF40 | chromosome 4 open reading frame, human C8orf40 | PC-5p-113875_40 |
| C4H8ORF40 | chromosome 4 open reading frame, human C8orf40 | PC-5p-582426_7 |
| C4H8ORF40 | chromosome 4 open reading frame, human C8orf40 | aca-miR-18a-5p_R-2 |
| C4H8ORF40 | chromosome 4 open reading frame, human C8orf40 | gga-let-7g-3p_1ss22CT |
| SEC23A | Sec23 homolog A (S. cerevisiae) | PC-5p-97606_47 |
| SEC23A | Sec23 homolog A (S. cerevisiae) | gga-miR-1782_L+2R-1 |
| CCDC167 | coiled-coil domain containing 167 | PC-5p-233284_18 |
| CCDC167 | coiled-coil domain containing 167 | PC-5p-71982_65 |
| CCDC167 | coiled-coil domain containing 167 | gga-miR-1677-3p_L+1 |
| GEMIN2 | gem (nuclear organelle) associated protein 2 | PC-3p-173245_25 |
| GEMIN2 | gem (nuclear organelle) associated protein 2 | chi-miR-16b-5p_R+1 |
| GEMIN2 | gem (nuclear organelle) associated protein 2 | gga-let-7g-3p_1ss22CT |
| GEMIN2 | gem (nuclear organelle) associated protein 2 | gga-miR-16-5p |
| GEMIN2 | gem (nuclear organelle) associated protein 2 | gga-miR-1a-3p_R+1_1ss2GT |
| GEMIN2 | gem (nuclear organelle) associated protein 2 | gga-miR-7468-3p_L+3 |
| GEMIN2 | gem (nuclear organelle) associated protein 2 | tgu-miR-2970-5p_1ss21GT |
| TRAPPC6B | trafficking protein particle complex 6B | PC-5p-71982_65 |
| KANK1 | KN motif and ankyrin repeat domains 1 | chi-miR-16b-5p_R+1 |
| AARS2 | alanyl-tRNA synthetase 2, mitochondrial (putative) | gga-miR-7468-3p_L+3 |
| VLDLR | very low density lipoprotein receptor | PC-5p-582426_7 |
| VLDLR | very low density lipoprotein receptor | gga-let-7g-3p_1ss22CT |
| INTU | inturned planar cell polarity effector homolog (Drosophila) | gga-miR-1a-3p_R+1_1ss2GT |
| ZFC3H1 | zinc finger, C3H1-type containing | gga-miR-30a-5p_R+2_1ss13CT |
| ZFC3H1 | zinc finger, C3H1-type containing | gga-miR-30e-5p_R+5 |
| GLIS3 | GLIS family zinc finger 3 | gga-miR-33-3p_L+1R+1 |
| HSPA4L | heat shock 70kDa protein 4-like | PC-5p-113875_40 |
| HSPA4L | heat shock 70kDa protein 4-like | PC-5p-97606_47 |
| HSPA4L | heat shock 70kDa protein 4-like | chi-miR-16b-5p_R+1 |
| HSPA4L | heat shock 70kDa protein 4-like | gga-miR-16-5p |
| TBC1D15 | TBC1 domain family, member 15 | PC-5p-71982_65 |
| TBC1D15 | TBC1 domain family, member 15 | gga-let-7g-3p_1ss22CT |
| TBC1D15 | TBC1 domain family, member 15 | gga-miR-30a-5p_R+2_1ss13CT |
| TBC1D15 | TBC1 domain family, member 15 | gga-miR-30e-5p_R+5 |
| TBC1D15 | TBC1 domain family, member 15 | tgu-miR-2970-5p_1ss21GT |
| LARP1B | La ribonucleoprotein domain family, member 1B | PC-5p-92816_49 |
| LARP1B | La ribonucleoprotein domain family, member 1B | gga-miR-30a-5p_R+2_1ss13CT |
| LARP1B | La ribonucleoprotein domain family, member 1B | gga-miR-30e-5p_R+5 |
| VSX2 | visual system homeobox 2 | PC-5p-37716_121 |
| VSX2 | visual system homeobox 2 | PC-5p-97606_47 |
| PHF17 | PHD finger protein 17 | gga-miR-34b-5p_L-1R+1 |
| KRR1 | KRR1, small subunit (SSU) processome component, homolog (yeast) | PC-5p-37716_121 |
| MMACHC | methylmalonic aciduria (cobalamin deficiency) cblC type, with homocystinuria | PC-5p-71982_65 |
| MMACHC | methylmalonic aciduria (cobalamin deficiency) cblC type, with homocystinuria | aca-miR-18a-5p_R-2 |
| NAP1L1 | nucleosome assembly protein 1-like 1 | gga-miR-1782_L+2R-1 |
| NAP1L1 | nucleosome assembly protein 1-like 1 | gga-miR-30a-5p_R+2_1ss13CT |
| NAP1L1 | nucleosome assembly protein 1-like 1 | gga-miR-30e-5p_R+5 |
| ZDHHC17 | zinc finger, DHHC-type containing 17 | PC-5p-71982_65 |
| E2F7 | E2F transcription factor 7 | PC-5p-97606_47 |
| FCF1 | FCF1 small subunit (SSU) processome component homolog (S. cerevisiae) | gga-miR-7468-3p_L+3 |
| GTF2E2 | general transcription factor IIE, polypeptide 2, beta 34kDa | PC-5p-71982_65 |
| EIF2B2 | eukaryotic translation initiation factor 2B, subunit 2 beta, 39kDa | PC-5p-233284_18 |
| EIF2B2 | eukaryotic translation initiation factor 2B, subunit 2 beta, 39kDa | chi-miR-16b-5p_R+1 |
| EIF2B2 | eukaryotic translation initiation factor 2B, subunit 2 beta, 39kDa | gga-miR-16-5p |
| EIF2B2 | eukaryotic translation initiation factor 2B, subunit 2 beta, 39kDa | gga-miR-30a-5p_R+2_1ss13CT |
| EIF2B2 | eukaryotic translation initiation factor 2B, subunit 2 beta, 39kDa | gga-miR-30e-5p_R+5 |
| TMED10 | transmembrane emp24-like trafficking protein 10 (yeast) | PC-5p-92816_49 |
| TMED10 | transmembrane emp24-like trafficking protein 10 (yeast) | PC-5p-97606_47 |
| TMED10 | transmembrane emp24-like trafficking protein 10 (yeast) | hsa-miR-483-3p_L-1R+2 |
| TMED10 | transmembrane emp24-like trafficking protein 10 (yeast) | tgu-miR-2970-5p_1ss21GT |
| PPP1R12A | protein phosphatase 1, regulatory subunit 12A | PC-5p-113875_40 |
| MME | membrane metallo-endopeptidase | PC-5p-71982_65 |
| GPR149 | G protein-coupled receptor 149 | aca-miR-363-3p_R+1 |
| GPR149 | G protein-coupled receptor 149 | gga-miR-1782_L+2R-1 |
| GPR149 | G protein-coupled receptor 149 | gga-miR-34b-5p_L-1R+1 |
| GPR149 | G protein-coupled receptor 149 | oan-miR-363-3p_R+1 |
| GPR149 | G protein-coupled receptor 149 | tgu-miR-2970-5p_1ss21GT |
| XPO5 | exportin 5 | PC-5p-92816_49 |
| XPO5 | exportin 5 | gga-miR-1329-3p |
| TSPAN1 | tetraspanin 1 | gga-let-7g-3p_1ss22CT |
| POLR1C | polymerase (RNA) I polypeptide C, 30kDa | chi-miR-16b-5p_R+1 |
| POLR1C | polymerase (RNA) I polypeptide C, 30kDa | gga-miR-16-5p |
| NSUN4 | NOP2/Sun domain family, member 4 | PC-5p-92816_49 |
| MBNL1 | muscleblind-like (Drosophila) | aca-miR-18a-5p_R-2 |
| MBNL1 | muscleblind-like (Drosophila) | gga-miR-1677-3p_L+1 |
| MBNL1 | muscleblind-like (Drosophila) | gga-miR-1805-5p_L-1 |
| MBNL1 | muscleblind-like (Drosophila) | aca-miR-18a-5p_R-2 |
| MBNL1 | muscleblind-like (Drosophila) | gga-miR-1805-5p_L-1 |
| HERC3 | hect domain and RLD 3 | PC-5p-582426_7 |
| HERC3 | hect domain and RLD 3 | aca-miR-18a-5p_R-2 |
| HERC3 | hect domain and RLD 3 | gga-let-7g-3p_1ss22CT |
| MED12L | mediator complex subunit 12-like | tgu-miR-2970-5p_1ss21GT |
| ANGEL1 | angel homolog 1 (Drosophila) | PC-3p-173245_25 |
| ANGEL1 | angel homolog 1 (Drosophila) | PC-3p-457530_9 |
| ANGEL1 | angel homolog 1 (Drosophila) | gga-miR-1782_L+2R-1 |
| SNCA | synuclein, alpha (non A4 component of amyloid precursor) | gga-let-7g-3p_1ss22CT |
| EIF2A | eukaryotic translation initiation factor 2A, 65kDa | PC-5p-97606_47 |
| TMEM63C | transmembrane protein 63C | PC-3p-338480_12 |
| LOC395824 | vitamin D3 hydroxylase associated protein | chi-miR-16b-5p_R+1 |
| LOC395824 | vitamin D3 hydroxylase associated protein | gga-miR-16-5p |
| ABCC10 | ATP-binding cassette, sub-family C (CFTR/MRP), member 10 | PC-5p-97606_47 |
| FAM98A | family with sequence similarity 98, member A | PC-3p-173245_25 |
| TM4SF18 | transmembrane 4 L six family member 18 | PC-3p-173245_25 |
| TM4SF18 | transmembrane 4 L six family member 18 | PC-5p-37716_121 |
| TM4SF18 | transmembrane 4 L six family member 18 | gga-let-7g-3p_1ss22CT |
| FAAH | fatty acid amide hydrolase | gga-miR-30a-5p_R+2_1ss13CT |
| FAAH | fatty acid amide hydrolase | gga-miR-30e-5p_R+5 |
| GYG1 | glycogenin 1 | aca-miR-18a-5p_R-2 |
| ATPAF1 | ATP synthase mitochondrial F1 complex assembly factor 1 | gga-miR-7468-3p_L+3 |
| VIPAR | VPS33B interacting protein, apical-basolateral polarity regulator | PC-5p-582426_7 |
| CYP4B1 | cytochrome P450, family 4, subfamily B, polypeptide 1 | PC-5p-37716_121 |
| CYP4B1 | cytochrome P450, family 4, subfamily B, polypeptide 1 | PC-5p-582426_7 |
| TCERG1L | transcription elongation regulator 1-like | PC-3p-52432_89 |
| LOC424620 | uncharacterized LOC424620 | aca-miR-363-3p_R+1 |
| LOC424620 | uncharacterized LOC424620 | oan-miR-363-3p_R+1 |
| CMPK1 | cytidine monophosphate (UMP-CMP) kinase 1, cytosolic | PC-3p-457530_9 |
| CMPK1 | cytidine monophosphate (UMP-CMP) kinase 1, cytosolic | gga-let-7g-3p_1ss22CT |
| SPTLC2 | serine palmitoyltransferase, long chain base subunit 2 | PC-5p-113875_40 |
| ALKBH1 | alkB, alkylation repair homolog 1 (E. coli) | PC-3p-52432_89 |
| ALKBH1 | alkB, alkylation repair homolog 1 (E. coli) | PC-5p-97606_47 |
| ALKBH1 | alkB, alkylation repair homolog 1 (E. coli) | chi-miR-16b-5p_R+1 |
| ALKBH1 | alkB, alkylation repair homolog 1 (E. coli) | gga-miR-16-5p |
| NKX6-2 | NK6 homeobox 2 | PC-5p-582426_7 |
| ADCK1 | aarF domain containing kinase 1 | PC-3p-173245_25 |
| ADCK1 | aarF domain containing kinase 1 | chi-miR-16b-5p_R+1 |
| ADCK1 | aarF domain containing kinase 1 | gga-miR-16-5p |
| ADCK1 | aarF domain containing kinase 1 | gga-miR-34b-5p_L-1R+1 |
| ADCK1 | aarF domain containing kinase 1 | tgu-miR-2970-5p_1ss21GT |
| AGXT2L1 | alanine-glyoxylate aminotransferase 2-like 1 | aca-miR-363-3p_R+1 |
| AGXT2L1 | alanine-glyoxylate aminotransferase 2-like 1 | oan-miR-363-3p_R+1 |
| OSTC | oligosaccharyltransferase complex subunit | PC-5p-113875_40 |
| OSTC | oligosaccharyltransferase complex subunit | aca-miR-18a-5p_R-2 |
| LEF1 | lymphoid enhancer-binding factor 1 | PC-3p-338480_12 |
| LEF1 | lymphoid enhancer-binding factor 1 | gga-miR-34b-5p_L-1R+1 |
| LOC100859230 | putative cation exchanger C521.04c-like | gga-let-7g-3p_1ss22CT |
| ELAVL4 | ELAV (embryonic lethal, abnormal vision, Drosophila)-like 4 (Hu antigen D) | PC-3p-457530_9 |
| ELAVL4 | ELAV (embryonic lethal, abnormal vision, Drosophila)-like 4 (Hu antigen D) | gga-miR-1782_L+2R-1 |
| SGMS2 | sphingomyelin synthase 2 | aca-miR-363-3p_R+1 |
| SGMS2 | sphingomyelin synthase 2 | oan-miR-363-3p_R+1 |
| FAF1 | Fas (TNFRSF6) associated factor 1 | PC-5p-71982_65 |
| FAF1 | Fas (TNFRSF6) associated factor 1 | gga-miR-7468-3p_L+3 |
| BIRC6 | baculoviral IAP repeat containing 6 | gga-let-7g-3p_1ss22CT |
| BIRC6 | baculoviral IAP repeat containing 6 | gga-miR-1782_L+2R-1 |
| BIRC6 | baculoviral IAP repeat containing 6 | gga-miR-30a-5p_R+2_1ss13CT |
| BIRC6 | baculoviral IAP repeat containing 6 | gga-miR-30e-5p_R+5 |
| PAPSS1 | 3'-phosphoadenosine 5'-phosphosulfate synthase 1 | PC-3p-173245_25 |
| PAPSS1 | 3'-phosphoadenosine 5'-phosphosulfate synthase 1 | gga-miR-1329-3p |
| EPS15 | epidermal growth factor receptor pathway substrate 15 | aca-miR-18a-5p_R-2 |
| EPS15 | epidermal growth factor receptor pathway substrate 15 | chi-miR-16b-5p_R+1 |
| EPS15 | epidermal growth factor receptor pathway substrate 15 | gga-miR-16-5p |
| EPS15 | epidermal growth factor receptor pathway substrate 15 | gga-miR-1a-3p_R+1_1ss2GT |
| EPS15 | epidermal growth factor receptor pathway substrate 15 | gga-miR-7468-3p_L+3 |
| EPS15 | epidermal growth factor receptor pathway substrate 15 | hsa-miR-483-3p_L-1R+2 |
| EPS15 | epidermal growth factor receptor pathway substrate 15 | tgu-miR-2970-5p_1ss21GT |
| YIPF4 | Yip1 domain family, member 4 | PC-5p-71982_65 |
| YIPF4 | Yip1 domain family, member 4 | tgu-miR-2970-5p_1ss21GT |
| EIF2AK2 | eukaryotic translation initiation factor 2-alpha kinase 2 | PC-5p-113875_40 |
| EIF2AK2 | eukaryotic translation initiation factor 2-alpha kinase 2 | PC-5p-97606_47 |
| EIF2AK2 | eukaryotic translation initiation factor 2-alpha kinase 2 | hsa-miR-483-3p_L-1R+2 |
| CCDC75 | coiled-coil domain containing 75 | gga-miR-30a-5p_R+2_1ss13CT |
| CCDC75 | coiled-coil domain containing 75 | gga-miR-30e-5p_R+5 |
| TBCK | TBC1 domain containing kinase | gga-miR-7468-3p_L+3 |
| TXNDC12 | thioredoxin domain containing 12 (endoplasmic reticulum) | PC-5p-92816_49 |
| TXNDC12 | thioredoxin domain containing 12 (endoplasmic reticulum) | gga-miR-1677-3p_L+1 |
| TXNDC12 | thioredoxin domain containing 12 (endoplasmic reticulum) | gga-miR-33-3p_L+1R+1 |
| BTF3L4 | basic transcription factor 3-like 4 | PC-5p-233284_18 |
| BTF3L4 | basic transcription factor 3-like 4 | chi-miR-16b-5p_R+1 |
| BTF3L4 | basic transcription factor 3-like 4 | gga-miR-16-5p |
| HEATR5B | HEAT repeat containing 5B | aca-miR-363-3p_R+1 |
| HEATR5B | HEAT repeat containing 5B | oan-miR-363-3p_R+1 |
| GTF2A1 | general transcription factor IIA, 1, 19/37kDa | PC-5p-233284_18 |
| GTF2A1 | general transcription factor IIA, 1, 19/37kDa | gga-let-7g-3p_1ss22CT |
| GTF2A1 | general transcription factor IIA, 1, 19/37kDa | gga-miR-1782_L+2R-1 |
| GTF2A1 | general transcription factor IIA, 1, 19/37kDa | gga-miR-7468-3p_L+3 |
| SLC25A4 | solute carrier family 25 (mitochondrial carrier; adenine nucleotide translocator), member 4 | gga-miR-1805-5p_L-1 |
| SLC25A4 | solute carrier family 25 (mitochondrial carrier; adenine nucleotide translocator), member 4 | tgu-miR-2970-5p_1ss21GT |
| SLC30A6 | solute carrier family 30 (zinc transporter), member 6 | PC-3p-457530_9 |
| CC2D1B | coiled-coil and C2 domain containing 1B | chi-miR-16b-5p_R+1 |
| CC2D1B | coiled-coil and C2 domain containing 1B | gga-miR-16-5p |
| CC2D1B | coiled-coil and C2 domain containing 1B | gga-miR-1782_L+2R-1 |
| DPY30 | dpy-30 homolog (C. elegans) | PC-5p-233284_18 |
| ACSL1 | acyl-CoA synthetase long-chain family member 1 | PC-5p-233284_18 |
| ACSL1 | acyl-CoA synthetase long-chain family member 1 | PC-5p-97606_47 |
| ACSL1 | acyl-CoA synthetase long-chain family member 1 | aca-miR-363-3p_R+1 |
| ACSL1 | acyl-CoA synthetase long-chain family member 1 | gga-let-7g-3p_1ss22CT |
| ACSL1 | acyl-CoA synthetase long-chain family member 1 | gga-miR-1782_L+2R-1 |
| ACSL1 | acyl-CoA synthetase long-chain family member 1 | gga-miR-34b-5p_L-1R+1 |
| ACSL1 | acyl-CoA synthetase long-chain family member 1 | oan-miR-363-3p_R+1 |
| AHCTF1 | AT hook containing transcription factor 1 | gga-miR-1782_L+2R-1 |
| CASP3 | caspase 3, apoptosis-related cysteine peptidase | PC-5p-71982_65 |
| CASP3 | caspase 3, apoptosis-related cysteine peptidase | gga-miR-34b-5p_L-1R+1 |
| SCCPDH | saccharopine dehydrogenase (putative) | gga-miR-30a-5p_R+2_1ss13CT |
| SCCPDH | saccharopine dehydrogenase (putative) | gga-miR-30e-5p_R+5 |
| SCCPDH | saccharopine dehydrogenase (putative) | tgu-miR-2970-5p_1ss21GT |
| ZYG11B | zyg-11 homolog B (C. elegans) | PC-5p-97606_47 |
| SCP2 | sterol carrier protein 2 | PC-3p-173245_25 |
| SCP2 | sterol carrier protein 2 | PC-5p-71982_65 |
| SCP2 | sterol carrier protein 2 | chi-miR-16b-5p_R+1 |
| SCP2 | sterol carrier protein 2 | gga-miR-16-5p |
| TFB2M | transcription factor B2, mitochondrial | chi-miR-16b-5p_R+1 |
| TFB2M | transcription factor B2, mitochondrial | gga-miR-16-5p |
| PODN | podocan | PC-3p-338480_12 |
| PODN | podocan | PC-5p-233284_18 |
| PODN | podocan | aca-miR-363-3p_R+1 |
| PODN | podocan | gga-miR-34b-5p_L-1R+1 |
| PODN | podocan | gga-miR-7468-3p_L+3 |
| PODN | podocan | oan-miR-363-3p_R+1 |
| C4H4ORF41 | chromosome 4 open reading frame, human C4orf41 | gga-miR-33-3p_L+1R+1 |
| WWC2 | WW and C2 domain containing 2 | gga-miR-1a-3p_R+1_1ss2GT |
| DCTD | dCMP deaminase | gga-miR-1a-3p_R+1_1ss2GT |
| DCTD | dCMP deaminase | hsa-miR-483-3p_L-1R+2 |
| C5H14ORF102 | chromosome 5 open reading frame, human C14orf102 | tgu-miR-2970-5p_1ss21GT |
| PPPDE1 | PPPDE peptidase domain containing 1 | PC-3p-338480_12 |
| PPPDE1 | PPPDE peptidase domain containing 1 | PC-5p-113875_40 |
| PPPDE1 | PPPDE peptidase domain containing 1 | PC-5p-233284_18 |
| PPPDE1 | PPPDE peptidase domain containing 1 | gga-let-7g-3p_1ss22CT |
| PPPDE1 | PPPDE peptidase domain containing 1 | gga-miR-1a-3p_R+1_1ss2GT |
| PPPDE1 | PPPDE peptidase domain containing 1 | gga-miR-30a-5p_R+2_1ss13CT |
| PPPDE1 | PPPDE peptidase domain containing 1 | gga-miR-30e-5p_R+5 |
| PPPDE1 | PPPDE peptidase domain containing 1 | gga-miR-33-3p_L+1R+1 |
| TTC7B | tetratricopeptide repeat domain 7B | PC-5p-71982_65 |
| MAGOH2 | mago-nashi homolog 2 (pseudogene) | PC-5p-37716_121 |
| MAGOH2 | mago-nashi homolog 2 (pseudogene) | gga-let-7g-3p_1ss22CT |
| MAGOH2 | mago-nashi homolog 2 (pseudogene) | tgu-miR-2970-5p_1ss21GT |
| MIOS | missing oocyte, meiosis regulator, homolog (Drosophila) | tgu-miR-2970-5p_1ss21GT |
| ICA1 | islet cell autoantigen 1, 69kDa | PC-5p-92816_49 |
| CCDC88C | coiled-coil domain containing 88C | gga-miR-7468-3p_L+3 |
| AGA | aspartylglucosaminidase | PC-5p-92816_49 |
| AGA | aspartylglucosaminidase | gga-miR-1677-3p_L+1 |
| AGA | aspartylglucosaminidase | gga-miR-1782_L+2R-1 |
| SMEK1 | SMEK homolog 1, suppressor of mek1 (Dictyostelium) | PC-5p-582426_7 |
| SMEK1 | SMEK homolog 1, suppressor of mek1 (Dictyostelium) | PC-5p-71982_65 |
| SMEK1 | SMEK homolog 1, suppressor of mek1 (Dictyostelium) | gga-miR-33-3p_L+1R+1 |
| TMEM48 | transmembrane protein 48 | PC-5p-113875_40 |
| MAP1LC3C | microtubule-associated protein 1 light chain 3 gamma | PC-5p-582426_7 |
| GALNT7 | UDP-N-acetyl-alpha-D-galactosamine:polypeptide N-acetylgalactosaminyltransferase 7 (GalNAc-T7) | gga-let-7g-3p_1ss22CT |
| HMGB2 | high mobility group box 2 | chi-miR-16b-5p_R+1 |
| HMGB2 | high mobility group box 2 | gga-let-7g-3p_1ss22CT |
| HMGB2 | high mobility group box 2 | gga-miR-16-5p |
| TMEM59 | transmembrane protein 59 | gga-miR-1a-3p_R+1_1ss2GT |
| TMEM59 | transmembrane protein 59 | gga-miR-33-3p_L+1R+1 |
| TMEM59 | transmembrane protein 59 | tgu-miR-2970-5p_1ss21GT |
| SCIN | scinderin | PC-3p-173245_25 |
| SCIN | scinderin | PC-5p-71982_65 |
| GLRA3 | glycine receptor, alpha 3 | hsa-miR-483-3p_L-1R+2 |
| CPSF2 | cleavage and polyadenylation specific factor 2, 100kDa | PC-3p-338480_12 |
| CPSF2 | cleavage and polyadenylation specific factor 2, 100kDa | gga-let-7g-3p_1ss22CT |
| CPSF2 | cleavage and polyadenylation specific factor 2, 100kDa | tgu-miR-2970-5p_1ss21GT |
| TTC4 | tetratricopeptide repeat domain 4 | gga-miR-1782_L+2R-1 |
| SLC24A4 | solute carrier family 24 (sodium/potassium/calcium exchanger), member 4 | chi-miR-16b-5p_R+1 |
| SLC24A4 | solute carrier family 24 (sodium/potassium/calcium exchanger), member 4 | gga-miR-16-5p |
| MEOX2 | mesenchyme homeobox 2 | chi-miR-16b-5p_R+1 |
| MEOX2 | mesenchyme homeobox 2 | gga-miR-1456-5p_L-1 |
| MEOX2 | mesenchyme homeobox 2 | gga-miR-16-5p |
| DHCR24 | 24-dehydrocholesterol reductase | PC-5p-233284_18 |
| DHCR24 | 24-dehydrocholesterol reductase | PC-5p-97606_47 |
| BZW2 | basic leucine zipper and W2 domains 2 | PC-5p-582426_7 |
| RYR2 | ryanodine receptor 2 (cardiac) | PC-5p-92816_49 |
| GOLGA5 | golgin A5 | aca-miR-363-3p_R+1 |
| GOLGA5 | golgin A5 | gga-miR-30a-5p_R+2_1ss13CT |
| GOLGA5 | golgin A5 | gga-miR-30e-5p_R+5 |
| GOLGA5 | golgin A5 | oan-miR-363-3p_R+1 |
| GOLGA5 | golgin A5 | tgu-miR-2970-5p_1ss21GT |
| PPAP2B | phosphatidic acid phosphatase type 2B | PC-5p-233284_18 |
| PPAP2B | phosphatidic acid phosphatase type 2B | aca-miR-363-3p_R+1 |
| PPAP2B | phosphatidic acid phosphatase type 2B | chi-miR-16b-5p_R+1 |
| PPAP2B | phosphatidic acid phosphatase type 2B | gga-miR-16-5p |
| PPAP2B | phosphatidic acid phosphatase type 2B | oan-miR-363-3p_R+1 |
| CHGA | chromogranin A (parathyroid secretory protein 1) | PC-5p-71982_65 |
| CHGA | chromogranin A (parathyroid secretory protein 1) | PC-5p-97606_47 |
| CHGA | chromogranin A (parathyroid secretory protein 1) | tgu-miR-2970-5p_1ss21GT |
| AGR2 | anterior gradient homolog 2 (Xenopus laevis) | PC-5p-233284_18 |
| AGR2 | anterior gradient homolog 2 (Xenopus laevis) | gga-let-7g-3p_1ss22CT |
| AGR2 | anterior gradient homolog 2 (Xenopus laevis) | gga-miR-30a-5p_R+2_1ss13CT |
| AGR2 | anterior gradient homolog 2 (Xenopus laevis) | gga-miR-30e-5p_R+5 |
| ITPK1 | inositol-tetrakisphosphate 1-kinase | PC-3p-338480_12 |
| ITPK1 | inositol-tetrakisphosphate 1-kinase | PC-3p-52432_89 |
| ITPK1 | inositol-tetrakisphosphate 1-kinase | PC-5p-113875_40 |
| ITPK1 | inositol-tetrakisphosphate 1-kinase | PC-5p-71982_65 |
| ITPK1 | inositol-tetrakisphosphate 1-kinase | gga-miR-1456-5p_L-1 |
| ITPK1 | inositol-tetrakisphosphate 1-kinase | gga-miR-30a-5p_R+2_1ss13CT |
| ITPK1 | inositol-tetrakisphosphate 1-kinase | gga-miR-30e-5p_R+5 |
| MTR | 5-methyltetrahydrofolate-homocysteine methyltransferase | PC-5p-92816_49 |
| MTR | 5-methyltetrahydrofolate-homocysteine methyltransferase | gga-miR-1677-3p_L+1 |
| MTR | 5-methyltetrahydrofolate-homocysteine methyltransferase | tgu-miR-2970-5p_1ss21GT |
| AHR | aryl hydrocarbon receptor | tgu-miR-2970-5p_1ss21GT |
| ERO1LB | ERO1-like beta (S. cerevisiae) | PC-5p-71982_65 |
| UBR7 | ubiquitin protein ligase E3 component n-recognin 7 (putative) | PC-5p-92816_49 |
| UBR7 | ubiquitin protein ligase E3 component n-recognin 7 (putative) | gga-miR-1782_L+2R-1 |
| UBR7 | ubiquitin protein ligase E3 component n-recognin 7 (putative) | gga-miR-33-3p_L+1R+1 |
| AREGB | amphiregulin B | chi-miR-16b-5p_R+1 |
| AREGB | amphiregulin B | gga-miR-16-5p |
| AREGB | amphiregulin B | gga-miR-7468-3p_L+3 |
| MACC1 | metastasis associated in colon cancer 1 | chi-miR-16b-5p_R+1 |
| MACC1 | metastasis associated in colon cancer 1 | gga-miR-16-5p |
| MACC1 | metastasis associated in colon cancer 1 | gga-miR-30a-5p_R+2_1ss13CT |
| MACC1 | metastasis associated in colon cancer 1 | gga-miR-30e-5p_R+5 |
| UNC79 | unc-79 homolog (C. elegans) | PC-5p-233284_18 |
| FGGY | FGGY carbohydrate kinase domain containing | PC-5p-92816_49 |
| G3BP2 | GTPase activating protein (SH3 domain) binding protein 2 | PC-5p-97606_47 |
| G3BP2 | GTPase activating protein (SH3 domain) binding protein 2 | gga-miR-1677-3p_L+1 |
| HOOK1 | hook homolog 1 (Drosophila) | gga-miR-7468-3p_L+3 |
| SPC25 | SPC25, NDC80 kinetochore complex component, homolog (S. cerevisiae) | gga-miR-33-3p_L+1R+1 |
| RAPGEF5 | Rap guanine nucleotide exchange factor (GEF) 5 | PC-5p-113875_40 |
| RAPGEF5 | Rap guanine nucleotide exchange factor (GEF) 5 | gga-miR-1782_L+2R-1 |
| LOC424676 | cytochrome P450 2J2-like | PC-5p-233284_18 |
| CYP2J2 | cytochrome P450, family 2, subfamily J, polypeptide 2 | chi-miR-16b-5p_R+1 |
| CYP2J2 | cytochrome P450, family 2, subfamily J, polypeptide 2 | gga-miR-16-5p |
| STK39 | serine threonine kinase 39 | chi-miR-16b-5p_R+1 |
| STK39 | serine threonine kinase 39 | gga-miR-16-5p |
| STK39 | serine threonine kinase 39 | gga-miR-1a-3p_R+1_1ss2GT |
| STK39 | serine threonine kinase 39 | tgu-miR-2970-5p_1ss21GT |
| NUDT9 | nudix (nucleoside diphosphate linked moiety X)-type motif 9 | aca-miR-363-3p_R+1 |
| NUDT9 | nudix (nucleoside diphosphate linked moiety X)-type motif 9 | oan-miR-363-3p_R+1 |
| NUDT9 | nudix (nucleoside diphosphate linked moiety X)-type motif 9 | tgu-miR-2970-5p_1ss21GT |
| INADL | InaD-like (Drosophila) | PC-5p-113875_40 |
| KANK4 | KN motif and ankyrin repeat domains 4 | PC-5p-97606_47 |
| GALNT3 | UDP-N-acetyl-alpha-D-galactosamine:polypeptide N-acetylgalactosaminyltransferase 3 (GalNAc-T3) | gga-miR-1782_L+2R-1 |
| GALNT3 | UDP-N-acetyl-alpha-D-galactosamine:polypeptide N-acetylgalactosaminyltransferase 3 (GalNAc-T3) | gga-miR-30a-5p_R+2_1ss13CT |
| GALNT3 | UDP-N-acetyl-alpha-D-galactosamine:polypeptide N-acetylgalactosaminyltransferase 3 (GalNAc-T3) | gga-miR-30e-5p_R+5 |
| IGF2BP3 | insulin-like growth factor 2 mRNA binding protein 3 | PC-3p-457530_9 |
| IGF2BP3 | insulin-like growth factor 2 mRNA binding protein 3 | PC-5p-92816_49 |
| IGF2BP3 | insulin-like growth factor 2 mRNA binding protein 3 | gga-miR-1782_L+2R-1 |
| IGF2BP3 | insulin-like growth factor 2 mRNA binding protein 3 | gga-miR-34b-5p_L-1R+1 |
| DOCK7 | dedicator of cytokinesis 7 | gga-miR-1782_L+2R-1 |
| TRA2A | transformer 2 alpha homolog (Drosophila) | PC-3p-173245_25 |
| TRA2A | transformer 2 alpha homolog (Drosophila) | PC-5p-37716_121 |
| TRA2A | transformer 2 alpha homolog (Drosophila) | PC-5p-582426_7 |
| TRA2A | transformer 2 alpha homolog (Drosophila) | gga-miR-1677-3p_L+1 |
| HSD17B11 | hydroxysteroid (17-beta) dehydrogenase 11 | PC-5p-71982_65 |
| HSD17B11 | hydroxysteroid (17-beta) dehydrogenase 11 | gga-miR-1677-3p_L+1 |
| STK31 | serine/threonine kinase 31 | PC-3p-173245_25 |
| NID1 | nidogen 1 | PC-5p-97606_47 |
| NID1 | nidogen 1 | aca-miR-18a-5p_R-2 |
| NID1 | nidogen 1 | tgu-miR-2970-5p_1ss21GT |
| ATG4C | autophagy related 4C, cysteine peptidase | PC-5p-233284_18 |
| DFNA5 | deafness, autosomal dominant 5 | PC-5p-71982_65 |
| DFNA5 | deafness, autosomal dominant 5 | PC-5p-97606_47 |
| KCNK1 | potassium channel, subfamily K, member 1 | gga-miR-33-3p_L+1R+1 |
| PGM1 | phosphoglucomutase 1 | chi-miR-16b-5p_R+1 |
| PGM1 | phosphoglucomutase 1 | gga-miR-16-5p |
| KLHL8 | kelch-like 8 (Drosophila) | PC-5p-97606_47 |
| ROR1 | receptor tyrosine kinase-like orphan receptor 1 | PC-3p-338480_12 |
| ROR1 | receptor tyrosine kinase-like orphan receptor 1 | PC-5p-71982_65 |
| ROR1 | receptor tyrosine kinase-like orphan receptor 1 | aca-miR-363-3p_R+1 |
| ROR1 | receptor tyrosine kinase-like orphan receptor 1 | oan-miR-363-3p_R+1 |
| TSNAX | translin-associated factor X | gga-miR-34b-5p_L-1R+1 |
| TSNAX | translin-associated factor X | tgu-miR-2970-5p_1ss21GT |
| HNRNPA2B1 | heterogeneous nuclear ribonucleoprotein A2/B1 | PC-5p-71982_65 |
| CBX3 | chromobox homolog 3 | PC-3p-173245_25 |
| CBX3 | chromobox homolog 3 | PC-5p-97606_47 |
| CBX3 | chromobox homolog 3 | gga-miR-1782_L+2R-1 |
| CBX3 | chromobox homolog 3 | gga-miR-30a-5p_R+2_1ss13CT |
| CBX3 | chromobox homolog 3 | gga-miR-30e-5p_R+5 |
| DNAJC6 | DnaJ (Hsp40) homolog, subfamily C, member 6 | PC-5p-233284_18 |
| DNAJC6 | DnaJ (Hsp40) homolog, subfamily C, member 6 | chi-miR-16b-5p_R+1 |
| DNAJC6 | DnaJ (Hsp40) homolog, subfamily C, member 6 | gga-miR-16-5p |
| SNX10 | sorting nexin 10 | PC-3p-173245_25 |
| SNX10 | sorting nexin 10 | PC-5p-92816_49 |
| SNX10 | sorting nexin 10 | chi-miR-16b-5p_R+1 |
| SNX10 | sorting nexin 10 | gga-miR-16-5p |
| SNX10 | sorting nexin 10 | gga-miR-1677-3p_L+1 |
| LEPROT | leptin receptor overlapping transcript | PC-3p-173245_25 |
| LEPROT | leptin receptor overlapping transcript | PC-3p-457530_9 |
| LEPROT | leptin receptor overlapping transcript | aca-miR-363-3p_R+1 |
| LEPROT | leptin receptor overlapping transcript | gga-miR-1782_L+2R-1 |
| LEPROT | leptin receptor overlapping transcript | oan-miR-363-3p_R+1 |
| COBLL1 | COBL-like 1 | chi-miR-16b-5p_R+1 |
| COBLL1 | COBL-like 1 | gga-miR-16-5p |
| ATG2B | autophagy related 2B | PC-5p-582426_7 |
| C5H14ORF129 | chromosome 5 open reading frame, human C14orf129 | gga-miR-1677-3p_L+1 |
| C5H14ORF129 | chromosome 5 open reading frame, human C14orf129 | gga-miR-1782_L+2R-1 |
| C5H14ORF129 | chromosome 5 open reading frame, human C14orf129 | gga-miR-30a-5p_R+2_1ss13CT |
| C5H14ORF129 | chromosome 5 open reading frame, human C14orf129 | gga-miR-30e-5p_R+5 |
| C5H14ORF129 | chromosome 5 open reading frame, human C14orf129 | hsa-miR-483-3p_L-1R+2 |
| URB2 | URB2 ribosome biogenesis 2 homolog (S. cerevisiae) | gga-miR-1329-3p |
| DPP4 | dipeptidyl-peptidase 4 | PC-5p-113875_40 |
| VRK1 | vaccinia related kinase 1 | PC-5p-113875_40 |
| AGT | angiotensinogen (serpin peptidase inhibitor, clade A, member 8) | PC-5p-97606_47 |
| LRRIQ1 | leucine-rich repeats and IQ motif containing 1 | PC-5p-113875_40 |
| PSMD14 | proteasome (prosome, macropain) 26S subunit, non-ATPase, 14 | PC-5p-233284_18 |
| LIN54 | lin-54 homolog (C. elegans) | PC-5p-233284_18 |
| CAPN9 | calpain 9 | chi-miR-16b-5p_R+1 |
| CAPN9 | calpain 9 | gga-miR-16-5p |
| CCNK | cyclin K | PC-5p-92816_49 |
| CCNK | cyclin K | gga-let-7g-3p_1ss22CT |
| CCNK | cyclin K | gga-miR-30a-5p_R+2_1ss13CT |
| CCNK | cyclin K | gga-miR-30e-5p_R+5 |
| ITGB6 | integrin, beta 6 | PC-3p-173245_25 |
| SEC31A | SEC31 homolog A (S. cerevisiae) | PC-3p-52432_89 |
| SEC31A | SEC31 homolog A (S. cerevisiae) | PC-5p-37716_121 |
| PKDCC | protein kinase domain containing, cytoplasmic homolog (mouse) | PC-5p-113875_40 |
| PKDCC | protein kinase domain containing, cytoplasmic homolog (mouse) | PC-5p-97606_47 |
| PKDCC | protein kinase domain containing, cytoplasmic homolog (mouse) | chi-miR-16b-5p_R+1 |
| PKDCC | protein kinase domain containing, cytoplasmic homolog (mouse) | gga-miR-16-5p |
| C3H6ORF120 | chromosome 3 open reading frame, human C6orf120 | chi-miR-16b-5p_R+1 |
| C3H6ORF120 | chromosome 3 open reading frame, human C6orf120 | gga-miR-16-5p |
| C3H6ORF120 | chromosome 3 open reading frame, human C6orf120 | gga-miR-30a-5p_R+2_1ss13CT |
| C3H6ORF120 | chromosome 3 open reading frame, human C6orf120 | gga-miR-30e-5p_R+5 |
| C3H6ORF120 | chromosome 3 open reading frame, human C6orf120 | gga-miR-33-3p_L+1R+1 |
| KITLG | KIT ligand | PC-5p-97606_47 |
| KITLG | KIT ligand | gga-miR-1a-3p_R+1_1ss2GT |
| EVL | Enah/Vasp-like | PC-5p-233284_18 |
| EVL | Enah/Vasp-like | PC-5p-92816_49 |
| EVL | Enah/Vasp-like | chi-miR-16b-5p_R+1 |
| EVL | Enah/Vasp-like | gga-miR-16-5p |
| HACL1 | 2-hydroxyacyl-CoA lyase 1 | aca-miR-363-3p_R+1 |
| HACL1 | 2-hydroxyacyl-CoA lyase 1 | oan-miR-363-3p_R+1 |
| SERPINH1 | serpin peptidase inhibitor, clade H (heat shock protein 47), member 1, (collagen binding protein 1) | PC-3p-173245_25 |
| SERPINH1 | serpin peptidase inhibitor, clade H (heat shock protein 47), member 1, (collagen binding protein 1) | PC-5p-113875_40 |
| SERPINH1 | serpin peptidase inhibitor, clade H (heat shock protein 47), member 1, (collagen binding protein 1) | PC-5p-71982_65 |
| SERPINH1 | serpin peptidase inhibitor, clade H (heat shock protein 47), member 1, (collagen binding protein 1) | tgu-miR-2970-5p_1ss21GT |
| AGPAT9 | 1-acylglycerol-3-phosphate O-acyltransferase 9 | PC-3p-173245_25 |
| AGPAT9 | 1-acylglycerol-3-phosphate O-acyltransferase 9 | PC-5p-582426_7 |
| AGPAT9 | 1-acylglycerol-3-phosphate O-acyltransferase 9 | aca-miR-363-3p_R+1 |
| AGPAT9 | 1-acylglycerol-3-phosphate O-acyltransferase 9 | chi-miR-16b-5p_R+1 |
| AGPAT9 | 1-acylglycerol-3-phosphate O-acyltransferase 9 | gga-miR-16-5p |
| AGPAT9 | 1-acylglycerol-3-phosphate O-acyltransferase 9 | oan-miR-363-3p_R+1 |
| SLC4A3 | solute carrier family 4, anion exchanger, member 3 | gga-miR-34b-5p_L-1R+1 |
| ANKRD28 | ankyrin repeat domain 28 | PC-5p-582426_7 |
| CDS1 | CDP-diacylglycerol synthase (phosphatidate cytidylyltransferase) 1 | gga-miR-1782_L+2R-1 |
| WARS | tryptophanyl-tRNA synthetase | gga-let-7g-3p_1ss22CT |
| WLS | wntless homolog (Drosophila) | PC-5p-71982_65 |
| WLS | wntless homolog (Drosophila) | PC-5p-97606_47 |
| WLS | wntless homolog (Drosophila) | gga-miR-33-3p_L+1R+1 |
| OXNAD1 | oxidoreductase NAD-binding domain containing 1 | chi-miR-16b-5p_R+1 |
| OXNAD1 | oxidoreductase NAD-binding domain containing 1 | gga-miR-16-5p |
| RFTN1 | raftlin, lipid raft linker 1 | gga-miR-33-3p_L+1R+1 |
| LOC429115 | methylcrotonoyl-Coenzyme A carboxylase 2-like | aca-miR-18a-5p_R-2 |
| WDFY3 | WD repeat and FYVE domain containing 3 | PC-3p-338480_12 |
| WDFY3 | WD repeat and FYVE domain containing 3 | gga-miR-30a-5p_R+2_1ss13CT |
| WDFY3 | WD repeat and FYVE domain containing 3 | gga-miR-30e-5p_R+5 |
| PPP2R5C | protein phosphatase 2, regulatory subunit B', gamma | PC-3p-457530_9 |
| PPP2R5C | protein phosphatase 2, regulatory subunit B', gamma | PC-5p-71982_65 |
| PPP2R5C | protein phosphatase 2, regulatory subunit B', gamma | chi-miR-16b-5p_R+1 |
| PPP2R5C | protein phosphatase 2, regulatory subunit B', gamma | gga-miR-16-5p |
| PPP2R5C | protein phosphatase 2, regulatory subunit B', gamma | gga-miR-33-3p_L+1R+1 |
| LUM | lumican | PC-5p-92816_49 |
| RAB5A | RAB5A, member RAS oncogene family | PC-5p-582426_7 |
| RAB5A | RAB5A, member RAS oncogene family | gga-miR-1a-3p_R+1_1ss2GT |
| RAB5A | RAB5A, member RAS oncogene family | hsa-miR-483-3p_L-1R+2 |
| DEPDC1 | DEP domain containing 1 | gga-miR-30a-5p_R+2_1ss13CT |
| DEPDC1 | DEP domain containing 1 | gga-miR-30e-5p_R+5 |
| BTG1 | B-cell translocation gene 1, anti-proliferative | PC-3p-457530_9 |
| BTG1 | B-cell translocation gene 1, anti-proliferative | gga-let-7g-3p_1ss22CT |
| KAT2B | K(lysine) acetyltransferase 2B | gga-miR-1782_L+2R-1 |
| CRADD | CASP2 and RIPK1 domain containing adaptor with death domain | PC-3p-457530_9 |
| CRADD | CASP2 and RIPK1 domain containing adaptor with death domain | PC-5p-92816_49 |
| CRADD | CASP2 and RIPK1 domain containing adaptor with death domain | aca-miR-18a-5p_R-2 |
| CRADD | CASP2 and RIPK1 domain containing adaptor with death domain | chi-miR-16b-5p_R+1 |
| CRADD | CASP2 and RIPK1 domain containing adaptor with death domain | gga-miR-16-5p |
| TOP2B | topoisomerase (DNA) II beta 180kDa | PC-5p-97606_47 |
| LRRC40 | leucine rich repeat containing 40 | gga-let-7g-3p_1ss22CT |
| LRRC40 | leucine rich repeat containing 40 | gga-miR-30a-5p_R+2_1ss13CT |
| LRRC40 | leucine rich repeat containing 40 | gga-miR-30e-5p_R+5 |
| NGLY1 | N-glycanase 1 | aca-miR-18a-5p_R-2 |
| ANKRD13C | ankyrin repeat domain 13C | gga-miR-1456-5p_L-1 |
| STK16 | serine/threonine kinase 16 | PC-3p-338480_12 |
| NR2C1 | nuclear receptor subfamily 2, group C, member 1 | PC-5p-92816_49 |
| NR2C1 | nuclear receptor subfamily 2, group C, member 1 | aca-miR-18a-5p_R-2 |
| MLLT4 | myeloid/lymphoid or mixed-lineage leukemia (trithorax homolog, Drosophila); translocated to, 4 | PC-5p-71982_65 |
| CTH | cystathionase (cystathionine gamma-lyase) | gga-miR-1782_L+2R-1 |
| TECRL | trans-2,3-enoyl-CoA reductase-like | PC-5p-113875_40 |
| TECRL | trans-2,3-enoyl-CoA reductase-like | chi-miR-16b-5p_R+1 |
| TECRL | trans-2,3-enoyl-CoA reductase-like | gga-miR-16-5p |
| ZRANB2 | zinc finger, RAN-binding domain containing 2 | PC-5p-37716_121 |
| ZRANB2 | zinc finger, RAN-binding domain containing 2 | PC-5p-582426_7 |
| ZRANB2 | zinc finger, RAN-binding domain containing 2 | PC-5p-71982_65 |
| ZRANB2 | zinc finger, RAN-binding domain containing 2 | hsa-miR-483-3p_L-1R+2 |
| CRYZ | crystallin, zeta (quinone reductase) | PC-3p-173245_25 |
| CRYZ | crystallin, zeta (quinone reductase) | PC-5p-582426_7 |
| CRYZ | crystallin, zeta (quinone reductase) | PC-5p-71982_65 |
| CRYZ | crystallin, zeta (quinone reductase) | aca-miR-18a-5p_R-2 |
| CRYZ | crystallin, zeta (quinone reductase) | chi-miR-16b-5p_R+1 |
| CRYZ | crystallin, zeta (quinone reductase) | gga-miR-16-5p |
| LHX8 | LIM homeobox 8 | gga-miR-30a-5p_R+2_1ss13CT |
| LHX8 | LIM homeobox 8 | gga-miR-30e-5p_R+5 |
| LHX8 | LIM homeobox 8 | gga-miR-33-3p_L+1R+1 |
| METAP2 | methionyl aminopeptidase 2 | aca-miR-18a-5p_R-2 |
| METAP2 | methionyl aminopeptidase 2 | gga-miR-33-3p_L+1R+1 |
| BCS1L | BCS1-like (S. cerevisiae) | PC-3p-52432_89 |
| BCS1L | BCS1-like (S. cerevisiae) | PC-5p-113875_40 |
| BCS1L | BCS1-like (S. cerevisiae) | PC-5p-37716_121 |
| TRAF3 | TNF receptor-associated factor 3 | gga-miR-33-3p_L+1R+1 |
| TMEM66 | transmembrane protein 66 | PC-5p-113875_40 |
| LTA4H | leukotriene A4 hydrolase | chi-miR-16b-5p_R+1 |
| LTA4H | leukotriene A4 hydrolase | gga-miR-16-5p |
| ELK3 | ELK3, ETS-domain protein (SRF accessory protein 2) | PC-5p-97606_47 |
| ELK3 | ELK3, ETS-domain protein (SRF accessory protein 2) | gga-miR-34b-5p_L-1R+1 |
| EXOC3L4 | exocyst complex component 3-like 4 | PC-5p-92816_49 |
| EXOC3L4 | exocyst complex component 3-like 4 | gga-miR-33-3p_L+1R+1 |
| TNFAIP2 | tumor necrosis factor, alpha-induced protein 2 | gga-miR-34b-5p_L-1R+1 |
| ERI1 | exoribonuclease 1 | aca-miR-18a-5p_R-2 |
| STT3B | STT3, subunit of the oligosaccharyltransferase complex, homolog B (S. cerevisiae) | PC-5p-92816_49 |
| SEPT11 | septin 11 | hsa-miR-483-3p_L-1R+2 |
| NEDD1 | neural precursor cell expressed, developmentally down-regulated 1 | PC-5p-582426_7 |
| BRP44L | brain protein 44-like | PC-5p-92816_49 |
| BRP44L | brain protein 44-like | gga-miR-1782_L+2R-1 |
| T | T, brachyury homolog (mouse) | PC-5p-37716_121 |
| T | T, brachyury homolog (mouse) | PC-5p-71982_65 |
| T | T, brachyury homolog (mouse) | PC-5p-92816_49 |
| T | T, brachyury homolog (mouse) | chi-miR-16b-5p_R+1 |
| T | T, brachyury homolog (mouse) | gga-miR-16-5p |
| T | T, brachyury homolog (mouse) | gga-miR-7468-3p_L+3 |
| DYNC1LI1 | dynein, cytoplasmic 1, light intermediate chain 1 | PC-5p-233284_18 |
| XRCC5 | X-ray repair complementing defective repair in Chinese hamster cells 5 (double-strand-break rejoining) | PC-5p-71982_65 |
| PECR | peroxisomal trans-2-enoyl-CoA reductase | gga-miR-34b-5p_L-1R+1 |
| SCARB2 | scavenger receptor class B, member 2 | gga-let-7g-3p_1ss22CT |
| MARK3 | MAP/microtubule affinity-regulating kinase 3 | PC-5p-97606_47 |
| NUP54 | nucleoporin 54kDa | gga-miR-1782_L+2R-1 |
| ERCC3 | excision repair cross-complementing rodent repair deficiency, complementation group 3 | PC-3p-52432_89 |
| BAG5 | BCL2-associated athanogene 5 | PC-5p-97606_47 |
| BAG5 | BCL2-associated athanogene 5 | gga-miR-1782_L+2R-1 |
| BAG5 | BCL2-associated athanogene 5 | gga-miR-34b-5p_L-1R+1 |
| GGCT | gamma-glutamylcyclotransferase | PC-3p-457530_9 |
| GGCT | gamma-glutamylcyclotransferase | gga-let-7g-3p_1ss22CT |
| GGCT | gamma-glutamylcyclotransferase | gga-miR-30a-5p_R+2_1ss13CT |
| GGCT | gamma-glutamylcyclotransferase | gga-miR-30e-5p_R+5 |
| GGCT | gamma-glutamylcyclotransferase | gga-miR-7468-3p_L+3 |
| BIN1 | bridging integrator 1 | PC-5p-71982_65 |
| BIN1 | bridging integrator 1 | PC-5p-97606_47 |
| BIN1 | bridging integrator 1 | gga-miR-1456-3p_R+1 |
| AMPH | amphiphysin | chi-miR-16b-5p_R+1 |
| AMPH | amphiphysin | gga-miR-16-5p |
| AMPH | amphiphysin | tgu-miR-2970-5p_1ss21GT |
| BCL2L14 | BCL2-like 14 (apoptosis facilitator) | gga-miR-34b-5p_L-1R+1 |
| PPP1R13B | protein phosphatase 1, regulatory (inhibitor) subunit 13B | PC-3p-173245_25 |
| PPP1R13B | protein phosphatase 1, regulatory (inhibitor) subunit 13B | aca-miR-363-3p_R+1 |
| PPP1R13B | protein phosphatase 1, regulatory (inhibitor) subunit 13B | gga-miR-1782_L+2R-1 |
| PPP1R13B | protein phosphatase 1, regulatory (inhibitor) subunit 13B | oan-miR-363-3p_R+1 |
| SH3BP5 | SH3-domain binding protein 5 (BTK-associated) | gga-miR-33-3p_L+1R+1 |
| CAPN7 | calpain 7 | PC-5p-233284_18 |
| CAPN7 | calpain 7 | chi-miR-16b-5p_R+1 |
| CAPN7 | calpain 7 | gga-miR-16-5p |
| GRSF1 | G-rich RNA sequence binding factor 1 | chi-miR-16b-5p_R+1 |
| GRSF1 | G-rich RNA sequence binding factor 1 | gga-miR-16-5p |
| SCYL2 | SCY1-like 2 (S. cerevisiae) | gga-miR-1782_L+2R-1 |
| ILF2 | interleukin enhancer binding factor 2, 45kDa | PC-5p-71982_65 |
| MAP3K4 | mitogen-activated protein kinase kinase kinase 4 | chi-miR-16b-5p_R+1 |
| MAP3K4 | mitogen-activated protein kinase kinase kinase 4 | gga-miR-16-5p |
| MAP3K4 | mitogen-activated protein kinase kinase kinase 4 | tgu-miR-2970-5p_1ss21GT |
| ATP2C1 | ATPase, Ca++ transporting, type 2C, member 1 | gga-miR-33-3p_L+1R+1 |
| KIF26A | kinesin family member 26A | gga-let-7g-3p_1ss22CT |
| KIF26A | kinesin family member 26A | gga-miR-1782_L+2R-1 |
| SEC24B | SEC24 family, member B (S. cerevisiae) | hsa-miR-483-3p_L-1R+2 |
| NR1H4 | nuclear receptor subfamily 1, group H, member 4 | PC-3p-173245_25 |
| MRPL3 | mitochondrial ribosomal protein L3 | PC-5p-92816_49 |
| IGF2R | insulin-like growth factor 2 receptor | PC-5p-582426_7 |
| IGF2R | insulin-like growth factor 2 receptor | gga-let-7g-3p_1ss22CT |
| IGF2R | insulin-like growth factor 2 receptor | gga-miR-30a-5p_R+2_1ss13CT |
| IGF2R | insulin-like growth factor 2 receptor | gga-miR-30e-5p_R+5 |
| UTP20 | UTP20, small subunit (SSU) processome component, homolog (yeast) | PC-5p-92816_49 |
| MKI67IP | MKI67 (FHA domain) interacting nucleolar phosphoprotein | gga-miR-34b-5p_L-1R+1 |
| ACAT2 | acetyl-CoA acetyltransferase 2 | gga-miR-34b-5p_L-1R+1 |
| IL8L1 | interleukin 8-like 1 | PC-3p-457530_9 |
| IL8L1 | interleukin 8-like 1 | gga-miR-1677-3p_L+1 |
| HSPBAP1 | HSPB (heat shock 27kDa) associated protein 1 | PC-5p-113875_40 |
| HSPBAP1 | HSPB (heat shock 27kDa) associated protein 1 | hsa-miR-483-3p_L-1R+2 |
| DEPDC7 | DEP domain containing 7 | PC-5p-233284_18 |
| CENPC1 | centromere protein C 1 | PC-3p-173245_25 |
| CENPC1 | centromere protein C 1 | PC-5p-233284_18 |
| SEMA5B | sema domain, seven thrombospondin repeats (type 1 and type 1-like), transmembrane domain (TM) and short cytoplasmic domain, (semaphorin) 5B | PC-3p-52432_89 |
| SEMA5B | sema domain, seven thrombospondin repeats (type 1 and type 1-like), transmembrane domain (TM) and short cytoplasmic domain, (semaphorin) 5B | gga-miR-1782_L+2R-1 |
| C5H14ORF79 | chromosome 5 open reading frame, human C14orf79 | chi-miR-16b-5p_R+1 |
| C5H14ORF79 | chromosome 5 open reading frame, human C14orf79 | gga-let-7g-3p_1ss22CT |
| C5H14ORF79 | chromosome 5 open reading frame, human C14orf79 | gga-miR-16-5p |
| C5H14ORF79 | chromosome 5 open reading frame, human C14orf79 | gga-miR-1677-3p_L+1 |
| HIPK3 | homeodomain interacting protein kinase 3 | aca-miR-363-3p_R+1 |
| HIPK3 | homeodomain interacting protein kinase 3 | oan-miR-363-3p_R+1 |
| PTPLB | protein tyrosine phosphatase-like (proline instead of catalytic arginine), member b | chi-miR-16b-5p_R+1 |
| PTPLB | protein tyrosine phosphatase-like (proline instead of catalytic arginine), member b | gga-miR-16-5p |
| PTPLB | protein tyrosine phosphatase-like (proline instead of catalytic arginine), member b | gga-miR-1a-3p_R+1_1ss2GT |
| MYLK | myosin light chain kinase | PC-5p-97606_47 |
| MYLK | myosin light chain kinase | aca-miR-18a-5p_R-2 |
| MYLK | myosin light chain kinase | chi-miR-16b-5p_R+1 |
| MYLK | myosin light chain kinase | gga-miR-16-5p |
| BFSP2 | beaded filament structural protein 2, phakinin | PC-5p-582426_7 |
| WDR91 | WD repeat domain 91 | PC-3p-52432_89 |
| MGP | matrix Gla protein | chi-miR-16b-5p_R+1 |
| MGP | matrix Gla protein | gga-miR-16-5p |
| PDE4DIP | phosphodiesterase 4D interacting protein | gga-let-7g-3p_1ss22CT |
| ITGB5 | integrin, beta 5 | chi-miR-16b-5p_R+1 |
| ITGB5 | integrin, beta 5 | gga-miR-16-5p |
| ITGB5 | integrin, beta 5 | tgu-miR-2970-5p_1ss21GT |
| DDX47 | DEAD (Asp-Glu-Ala-Asp) box polypeptide 47 | PC-5p-113875_40 |
| DDX47 | DEAD (Asp-Glu-Ala-Asp) box polypeptide 47 | gga-miR-34b-5p_L-1R+1 |
| MTHFD1 | methylenetetrahydrofolate dehydrogenase (NADP+ dependent) 1, methenyltetrahydrofolate cyclohydrolase, formyltetrahydrofolate synthetase | PC-5p-233284_18 |
| MTHFD1 | methylenetetrahydrofolate dehydrogenase (NADP+ dependent) 1, methenyltetrahydrofolate cyclohydrolase, formyltetrahydrofolate synthetase | PC-5p-71982_65 |
| MTHFD1 | methylenetetrahydrofolate dehydrogenase (NADP+ dependent) 1, methenyltetrahydrofolate cyclohydrolase, formyltetrahydrofolate synthetase | gga-let-7g-3p_1ss22CT |
| MTHFD1 | methylenetetrahydrofolate dehydrogenase (NADP+ dependent) 1, methenyltetrahydrofolate cyclohydrolase, formyltetrahydrofolate synthetase | gga-miR-1677-3p_L+1 |
| MTHFD1 | methylenetetrahydrofolate dehydrogenase (NADP+ dependent) 1, methenyltetrahydrofolate cyclohydrolase, formyltetrahydrofolate synthetase | gga-miR-7468-3p_L+3 |
| HEBP1 | heme binding protein 1 | PC-5p-233284_18 |
| HEBP1 | heme binding protein 1 | PC-5p-71982_65 |
| HEBP1 | heme binding protein 1 | PC-5p-92816_49 |
| HEBP1 | heme binding protein 1 | gga-miR-1677-3p_L+1 |
| HEBP1 | heme binding protein 1 | gga-miR-34b-5p_L-1R+1 |
| ESR2 | estrogen receptor 2 (ER beta) | PC-3p-52432_89 |
| ESR2 | estrogen receptor 2 (ER beta) | PC-5p-97606_47 |
| ESR2 | estrogen receptor 2 (ER beta) | aca-miR-18a-5p_R-2 |
| ESR2 | estrogen receptor 2 (ER beta) | chi-miR-16b-5p_R+1 |
| ESR2 | estrogen receptor 2 (ER beta) | gga-miR-16-5p |
| SYNE2 | spectrin repeat containing, nuclear envelope 2 | PC-5p-233284_18 |
| SYNE2 | spectrin repeat containing, nuclear envelope 2 | chi-miR-16b-5p_R+1 |
| SYNE2 | spectrin repeat containing, nuclear envelope 2 | gga-miR-16-5p |
| ATF7IP | activating transcription factor 7 interacting protein | gga-miR-33-3p_L+1R+1 |
| SPATA5 | spermatogenesis associated 5 | PC-5p-71982_65 |
| TTC26 | tetratricopeptide repeat domain 26 | tgu-miR-2970-5p_1ss21GT |
| IL2 | interleukin 2 | PC-3p-52432_89 |
| IL2 | interleukin 2 | gga-miR-30a-5p_R+2_1ss13CT |
| IL2 | interleukin 2 | gga-miR-30e-5p_R+5 |
| UBN2 | ubinuclein 2 | gga-miR-30a-5p_R+2_1ss13CT |
| UBN2 | ubinuclein 2 | gga-miR-30e-5p_R+5 |
| LUC7L2 | LUC7-like 2 (S. cerevisiae) | PC-5p-582426_7 |
| KIAA1109 | KIAA1109 | aca-miR-363-3p_R+1 |
| KIAA1109 | KIAA1109 | oan-miR-363-3p_R+1 |
| POLDIP3 | polymerase (DNA-directed), delta interacting protein 3 | PC-5p-582426_7 |
| POLDIP3 | polymerase (DNA-directed), delta interacting protein 3 | PC-5p-92816_49 |
| POLDIP3 | polymerase (DNA-directed), delta interacting protein 3 | aca-miR-18a-5p_R-2 |
| POLDIP3 | polymerase (DNA-directed), delta interacting protein 3 | gga-let-7g-3p_1ss22CT |
| POLDIP3 | polymerase (DNA-directed), delta interacting protein 3 | gga-miR-34b-5p_L-1R+1 |
| POLDIP3 | polymerase (DNA-directed), delta interacting protein 3 | hsa-miR-483-3p_L-1R+2 |
| SERHL2 | serine hydrolase-like 2 | PC-5p-92816_49 |
| SERHL2 | serine hydrolase-like 2 | chi-miR-16b-5p_R+1 |
| SERHL2 | serine hydrolase-like 2 | gga-miR-16-5p |
| SNAPC1 | small nuclear RNA activating complex, polypeptide 1, 43kDa | PC-3p-52432_89 |
| SNAPC1 | small nuclear RNA activating complex, polypeptide 1, 43kDa | gga-let-7g-3p_1ss22CT |
| HIF1A | hypoxia inducible factor 1, alpha subunit (basic helix-loop-helix transcription factor) | PC-5p-113875_40 |
| HIF1A | hypoxia inducible factor 1, alpha subunit (basic helix-loop-helix transcription factor) | PC-5p-233284_18 |
| HIF1A | hypoxia inducible factor 1, alpha subunit (basic helix-loop-helix transcription factor) | aca-miR-18a-5p_R-2 |
| HIF1A | hypoxia inducible factor 1, alpha subunit (basic helix-loop-helix transcription factor) | gga-let-7g-3p_1ss22CT |
| HIF1A | hypoxia inducible factor 1, alpha subunit (basic helix-loop-helix transcription factor) | gga-miR-1a-3p_R+1_1ss2GT |
| LARS2 | leucyl-tRNA synthetase 2, mitochondrial | chi-miR-16b-5p_R+1 |
| LARS2 | leucyl-tRNA synthetase 2, mitochondrial | gga-miR-16-5p |
| CCNA2 | cyclin A2 | PC-5p-113875_40 |
| CCNA2 | cyclin A2 | gga-miR-1782_L+2R-1 |
| CLEC3B | C-type lectin domain family 3, member B | PC-3p-52432_89 |
| CLEC3B | C-type lectin domain family 3, member B | PC-5p-71982_65 |
| CLEC3B | C-type lectin domain family 3, member B | chi-miR-16b-5p_R+1 |
| CLEC3B | C-type lectin domain family 3, member B | gga-miR-16-5p |
| ANXA5 | annexin A5 | PC-5p-97606_47 |
| ZDHHC3 | zinc finger, DHHC-type containing 3 | gga-miR-1782_L+2R-1 |
| TGM4 | transglutaminase 4 (prostate) | PC-3p-338480_12 |
| TGM4 | transglutaminase 4 (prostate) | gga-miR-34b-5p_L-1R+1 |
| SLC38A6 | solute carrier family 38, member 6 | gga-miR-30a-5p_R+2_1ss13CT |
| SLC38A6 | solute carrier family 38, member 6 | gga-miR-30e-5p_R+5 |
| CYP2D6 | cytochrome P450, family 2, subfamily D, polypeptide 6 | gga-miR-30a-5p_R+2_1ss13CT |
| CYP2D6 | cytochrome P450, family 2, subfamily D, polypeptide 6 | gga-miR-30e-5p_R+5 |
| SIDT2 | SID1 transmembrane family, member 2 | PC-3p-52432_89 |
| C22ORF32 | chromosome 1 open reading frame, human C22orf32 | hsa-miR-483-3p_L-1R+2 |
| NAGA | N-acetylgalactosaminidase, alpha- | hsa-miR-483-3p_L-1R+2 |
| KIAA1143 | KIAA1143 | gga-miR-1a-3p_R+1_1ss2GT |
| KIAA1143 | KIAA1143 | gga-miR-7468-3p_L+3 |
| KIAA1143 | KIAA1143 | hsa-miR-483-3p_L-1R+2 |
| CTNNB1 | catenin (cadherin-associated protein), beta 1, 88kDa | PC-5p-233284_18 |
| CTNNB1 | catenin (cadherin-associated protein), beta 1, 88kDa | hsa-miR-483-3p_L-1R+2 |
| C5H14ORF135 | chromosome 5 open reading frame, human C14orf135 | chi-miR-16b-5p_R+1 |
| C5H14ORF135 | chromosome 5 open reading frame, human C14orf135 | gga-miR-16-5p |
| CCK | cholecystokinin | gga-let-7g-3p_1ss22CT |
| POLR3H | polymerase (RNA) III (DNA directed) polypeptide H (22.9kD) | PC-5p-97606_47 |
| POLR3H | polymerase (RNA) III (DNA directed) polypeptide H (22.9kD) | gga-miR-30a-5p_R+2_1ss13CT |
| POLR3H | polymerase (RNA) III (DNA directed) polypeptide H (22.9kD) | gga-miR-30e-5p_R+5 |
| TMC2 | transmembrane channel-like 2 | tgu-miR-2970-5p_1ss21GT |
| ACO2 | aconitase 2, mitochondrial | PC-5p-113875_40 |
| ACO2 | aconitase 2, mitochondrial | aca-miR-363-3p_R+1 |
| ACO2 | aconitase 2, mitochondrial | oan-miR-363-3p_R+1 |
| CCR8 | chemokine (C-C motif) receptor 8 | PC-3p-52432_89 |
| CCR8 | chemokine (C-C motif) receptor 8 | PC-5p-113875_40 |
| CCR8 | chemokine (C-C motif) receptor 8 | PC-5p-71982_65 |
| CCR8 | chemokine (C-C motif) receptor 8 | aca-miR-18a-5p_R-2 |
| CCR8 | chemokine (C-C motif) receptor 8 | aca-miR-363-3p_R+1 |
| CCR8 | chemokine (C-C motif) receptor 8 | oan-miR-363-3p_R+1 |
| TOB2 | transducer of ERBB2, 2 | aca-miR-363-3p_R+1 |
| TOB2 | transducer of ERBB2, 2 | gga-let-7g-3p_1ss22CT |
| TOB2 | transducer of ERBB2, 2 | gga-miR-1782_L+2R-1 |
| TOB2 | transducer of ERBB2, 2 | oan-miR-363-3p_R+1 |
| RANGAP1 | Ran GTPase activating protein 1 | PC-5p-97606_47 |
| RANGAP1 | Ran GTPase activating protein 1 | gga-miR-34b-5p_L-1R+1 |
| RPL7L1 | ribosomal protein L7-like 1 | PC-5p-113875_40 |
| RPL7L1 | ribosomal protein L7-like 1 | tgu-miR-2970-5p_1ss21GT |
| FBXL2 | F-box and leucine-rich repeat protein 2 | PC-5p-97606_47 |
| L3MBTL2 | l(3)mbt-like 2 (Drosophila) | PC-5p-71982_65 |
| UBP1 | upstream binding protein 1 (LBP-1a) | gga-miR-34b-5p_L-1R+1 |
| MYOZ2 | myozenin 2 | gga-let-7g-3p_1ss22CT |
| CLASP2 | cytoplasmic linker associated protein 2 | gga-miR-7468-3p_L+3 |
| METTL14 | methyltransferase like 14 | PC-3p-457530_9 |
| METTL14 | methyltransferase like 14 | PC-5p-582426_7 |
| METTL14 | methyltransferase like 14 | gga-let-7g-3p_1ss22CT |
| XPNPEP3 | X-prolyl aminopeptidase (aminopeptidase P) 3, putative | PC-5p-97606_47 |
| XPNPEP3 | X-prolyl aminopeptidase (aminopeptidase P) 3, putative | chi-miR-16b-5p_R+1 |
| XPNPEP3 | X-prolyl aminopeptidase (aminopeptidase P) 3, putative | gga-miR-16-5p |
| XPNPEP3 | X-prolyl aminopeptidase (aminopeptidase P) 3, putative | gga-miR-30a-5p_R+2_1ss13CT |
| XPNPEP3 | X-prolyl aminopeptidase (aminopeptidase P) 3, putative | gga-miR-30e-5p_R+5 |
| PRSS12 | protease, serine, 12 (neurotrypsin, motopsin) | PC-5p-92816_49 |
| RTN1 | reticulon 1 | chi-miR-16b-5p_R+1 |
| RTN1 | reticulon 1 | gga-miR-16-5p |
| ST13 | suppression of tumorigenicity 13 (colon carcinoma) (Hsp70 interacting protein) | gga-miR-1a-3p_R+1_1ss2GT |
| CAMK2D | calcium/calmodulin-dependent protein kinase II delta | PC-5p-233284_18 |
| CAMK2D | calcium/calmodulin-dependent protein kinase II delta | gga-let-7g-3p_1ss22CT |
| CAMK2D | calcium/calmodulin-dependent protein kinase II delta | gga-miR-1782_L+2R-1 |
| CAMK2D | calcium/calmodulin-dependent protein kinase II delta | hsa-miR-483-3p_L-1R+2 |
| ARPP21 | cAMP-regulated phosphoprotein, 21kDa | aca-miR-425-3p_L-2R+1_1ss3AT |
| ARPP21 | cAMP-regulated phosphoprotein, 21kDa | chi-miR-16b-5p_R+1 |
| ARPP21 | cAMP-regulated phosphoprotein, 21kDa | gga-miR-16-5p |
| LARP7 | La ribonucleoprotein domain family, member 7 | gga-miR-1782_L+2R-1 |
| LARP7 | La ribonucleoprotein domain family, member 7 | gga-miR-33-3p_L+1R+1 |
| SNX4 | sorting nexin 4 | gga-miR-1805-5p_L-1 |
| DCLK3 | doublecortin-like kinase 3 | tgu-miR-2970-5p_1ss21GT |
| STARD3NL | STARD3 N-terminal like | gga-miR-30a-5p_R+2_1ss13CT |
| STARD3NL | STARD3 N-terminal like | gga-miR-30e-5p_R+5 |
| ELMO1 | engulfment and cell motility 1 | aca-miR-18a-5p_R-2 |
| SLC35F4 | solute carrier family 35, member F4 | gga-miR-30a-5p_R+2_1ss13CT |
| SLC35F4 | solute carrier family 35, member F4 | gga-miR-30e-5p_R+5 |
| NAA30 | N(alpha)-acetyltransferase 30, NatC catalytic subunit | gga-miR-33-3p_L+1R+1 |
| NAA30 | N(alpha)-acetyltransferase 30, NatC catalytic subunit | tgu-miR-2970-5p_1ss21GT |
| PCDP1 | primary ciliary dyskinesia protein 1 | aca-miR-363-3p_R+1 |
| PCDP1 | primary ciliary dyskinesia protein 1 | oan-miR-363-3p_R+1 |
| ENPEP | glutamyl aminopeptidase (aminopeptidase A) | PC-3p-338480_12 |
| ENPEP | glutamyl aminopeptidase (aminopeptidase A) | PC-5p-582426_7 |
| ENPEP | glutamyl aminopeptidase (aminopeptidase A) | aca-miR-18a-5p_R-2 |
| ENPEP | glutamyl aminopeptidase (aminopeptidase A) | gga-miR-34b-5p_L-1R+1 |
| EIF3M | eukaryotic translation initiation factor 3, subunit M | gga-miR-1782_L+2R-1 |
| EEPD1 | endonuclease/exonuclease/phosphatase family domain containing 1 | PC-5p-71982_65 |
| EEPD1 | endonuclease/exonuclease/phosphatase family domain containing 1 | gga-let-7g-3p_1ss22CT |
| C5H14ORF101 | chromosome 5 open reading frame, human C14orf101 | PC-5p-71982_65 |
| C5H14ORF101 | chromosome 5 open reading frame, human C14orf101 | gga-miR-1782_L+2R-1 |
| CFI | complement factor I | PC-5p-113875_40 |
| CFI | complement factor I | gga-let-7g-3p_1ss22CT |
| DNAJC24 | DnaJ (Hsp40) homolog, subfamily C, member 24 | gga-let-7g-3p_1ss22CT |
| FSHB | follicle stimulating hormone, beta polypeptide | gga-miR-1677-3p_L+1 |
| FSHB | follicle stimulating hormone, beta polypeptide | gga-miR-1a-3p_R+1_1ss2GT |
| HERPUD2 | HERPUD family member 2 | PC-5p-71982_65 |
| HERPUD2 | HERPUD family member 2 | aca-miR-363-3p_R+1 |
| HERPUD2 | HERPUD family member 2 | gga-miR-34b-5p_L-1R+1 |
| HERPUD2 | HERPUD family member 2 | oan-miR-363-3p_R+1 |
| TAB1 | TGF-beta activated kinase 1/MAP3K7 binding protein 1 | PC-5p-113875_40 |
| TAB1 | TGF-beta activated kinase 1/MAP3K7 binding protein 1 | gga-let-7g-3p_1ss22CT |
| FBXO34 | F-box protein 34 | PC-5p-71982_65 |
| FBXO34 | F-box protein 34 | gga-miR-30a-5p_R+2_1ss13CT |
| FBXO34 | F-box protein 34 | gga-miR-30e-5p_R+5 |
| ACTR3 | ARP3 actin-related protein 3 homolog (yeast) | PC-5p-113875_40 |
| ACTR3 | ARP3 actin-related protein 3 homolog (yeast) | hsa-miR-483-3p_L-1R+2 |
| KIF18A | kinesin family member 18A | PC-3p-173245_25 |
| PLA2G12A | phospholipase A2, group XIIA | PC-5p-113875_40 |
| PLA2G12A | phospholipase A2, group XIIA | gga-miR-1677-3p_L+1 |
| CASP6 | caspase 6, apoptosis-related cysteine peptidase | gga-miR-33-3p_L+1R+1 |
| BBS9 | Bardet-Biedl syndrome 9 | PC-3p-457530_9 |
| FKBP9 | FK506 binding protein 9, 63 kDa | PC-5p-37716_121 |
| FKBP9 | FK506 binding protein 9, 63 kDa | gga-miR-1677-3p_L+1 |
| FKBP9 | FK506 binding protein 9, 63 kDa | gga-miR-1782_L+2R-1 |
| FKBP9 | FK506 binding protein 9, 63 kDa | gga-miR-30a-5p_R+2_1ss13CT |
| FKBP9 | FK506 binding protein 9, 63 kDa | gga-miR-30e-5p_R+5 |
| DNAL4 | dynein, axonemal, light chain 4 | gga-miR-1456-5p_L-1 |
| SAMD4A | sterile alpha motif domain containing 4A | gga-miR-34b-5p_L-1R+1 |
| AVL9 | AVL9 homolog (S. cerevisiase) | PC-5p-582426_7 |
| AVL9 | AVL9 homolog (S. cerevisiase) | chi-miR-16b-5p_R+1 |
| AVL9 | AVL9 homolog (S. cerevisiase) | gga-miR-16-5p |
| CCNT2 | cyclin T2 | PC-3p-457530_9 |
| RAB3GAP1 | RAB3 GTPase activating protein subunit 1 (catalytic) | chi-miR-16b-5p_R+1 |
| RAB3GAP1 | RAB3 GTPase activating protein subunit 1 (catalytic) | gga-miR-16-5p |
| RAB3GAP1 | RAB3 GTPase activating protein subunit 1 (catalytic) | gga-miR-1782_L+2R-1 |
| CDKN3 | cyclin-dependent kinase inhibitor 3 | gga-miR-34b-5p_L-1R+1 |
| RAP1GDS1 | RAP1, GTP-GDP dissociation stimulator 1 | chi-miR-16b-5p_R+1 |
| RAP1GDS1 | RAP1, GTP-GDP dissociation stimulator 1 | gga-miR-16-5p |
| RAP1GDS1 | RAP1, GTP-GDP dissociation stimulator 1 | tgu-miR-2970-5p_1ss21GT |
| ZRANB3 | zinc finger, RAN-binding domain containing 3 | aca-miR-363-3p_R+1 |
| ZRANB3 | zinc finger, RAN-binding domain containing 3 | chi-miR-16b-5p_R+1 |
| ZRANB3 | zinc finger, RAN-binding domain containing 3 | gga-miR-16-5p |
| ZRANB3 | zinc finger, RAN-binding domain containing 3 | oan-miR-363-3p_R+1 |
| CIRBP | cold inducible RNA binding protein | PC-5p-582426_7 |
| CIRBP | cold inducible RNA binding protein | gga-miR-1456-5p_L-1 |
| ITGA9 | integrin, alpha 9 | PC-3p-173245_25 |
| ITGA9 | integrin, alpha 9 | PC-5p-233284_18 |
| ITGA9 | integrin, alpha 9 | aca-miR-18a-5p_R-2 |
| METAP1 | methionyl aminopeptidase 1 | PC-5p-92816_49 |
| METAP1 | methionyl aminopeptidase 1 | aca-miR-18a-5p_R-2 |
| METAP1 | methionyl aminopeptidase 1 | hsa-miR-483-3p_L-1R+2 |
| METAP1 | methionyl aminopeptidase 1 | tgu-miR-2970-5p_1ss21GT |
| KLHDC1 | kelch domain containing 1 | PC-5p-97606_47 |
| KLHDC1 | kelch domain containing 1 | gga-miR-1456-5p_L-1 |
| ADH1C | alcohol dehydrogenase 1C (class I), gamma polypeptide | chi-miR-16b-5p_R+1 |
| ADH1C | alcohol dehydrogenase 1C (class I), gamma polypeptide | gga-miR-16-5p |
| KDELR3 | KDEL (Lys-Asp-Glu-Leu) endoplasmic reticulum protein retention receptor 3 | PC-3p-338480_12 |
| KDELR3 | KDEL (Lys-Asp-Glu-Leu) endoplasmic reticulum protein retention receptor 3 | gga-miR-1456-3p_R+1 |
| UBXN4 | UBX domain protein 4 | gga-miR-1677-3p_L+1 |
| LRRFIP2 | leucine rich repeat (in FLII) interacting protein 2 | aca-miR-363-3p_R+1 |
| LRRFIP2 | leucine rich repeat (in FLII) interacting protein 2 | oan-miR-363-3p_R+1 |
| TARP | TCR gamma alternate reading frame protein | PC-3p-457530_9 |
| TARP | TCR gamma alternate reading frame protein | gga-miR-34b-5p_L-1R+1 |
| PLA2G6 | phospholipase A2, group VI (cytosolic, calcium-independent) | PC-5p-97606_47 |
| L2HGDH | L-2-hydroxyglutarate dehydrogenase | PC-5p-37716_121 |
| ATP5S | ATP synthase, H+ transporting, mitochondrial Fo complex, subunit s (factor B) | chi-miR-16b-5p_R+1 |
| ATP5S | ATP synthase, H+ transporting, mitochondrial Fo complex, subunit s (factor B) | gga-miR-16-5p |
| SLC16A8 | solute carrier family 16, member 8 (monocarboxylic acid transporter 3) | PC-5p-233284_18 |
| SHPRH | SNF2 histone linker PHD RING helicase, E3 ubiquitin protein ligase | tgu-miR-2970-5p_1ss21GT |
| SOX10 | SRY (sex determining region Y)-box 10 | PC-5p-233284_18 |
| SOX10 | SRY (sex determining region Y)-box 10 | gga-miR-1456-5p_L-1 |
| RAB32 | RAB32, member RAS oncogene family | chi-miR-16b-5p_R+1 |
| RAB32 | RAB32, member RAS oncogene family | gga-miR-16-5p |
| UBE2D3 | ubiquitin-conjugating enzyme E2D 3 | PC-3p-173245_25 |
| UBE2D3 | ubiquitin-conjugating enzyme E2D 3 | gga-miR-1677-3p_L+1 |
| UBE2D3 | ubiquitin-conjugating enzyme E2D 3 | gga-miR-30a-5p_R+2_1ss13CT |
| UBE2D3 | ubiquitin-conjugating enzyme E2D 3 | gga-miR-30e-5p_R+5 |
| NHEDC2 | Na+/H+ exchanger domain containing 2 | chi-miR-16b-5p_R+1 |
| NHEDC2 | Na+/H+ exchanger domain containing 2 | gga-miR-16-5p |
| LOC420770 | uncharacterized LOC420770 | gga-miR-1805-5p_L-1 |
| SAV1 | salvador homolog 1 (Drosophila) | aca-miR-18a-5p_R-2 |
| HECW1 | HECT, C2 and WW domain containing E3 ubiquitin protein ligase 1 | gga-miR-30a-5p_R+2_1ss13CT |
| HECW1 | HECT, C2 and WW domain containing E3 ubiquitin protein ligase 1 | gga-miR-30e-5p_R+5 |
| TDRD7 | tudor domain containing 7 | gga-miR-30a-5p_R+2_1ss13CT |
| TDRD7 | tudor domain containing 7 | gga-miR-30e-5p_R+5 |
| STK17A | serine/threonine kinase 17a | PC-5p-92816_49 |
| STK17A | serine/threonine kinase 17a | chi-miR-16b-5p_R+1 |
| STK17A | serine/threonine kinase 17a | gga-miR-16-5p |
| STK17A | serine/threonine kinase 17a | gga-miR-1782_L+2R-1 |
| NIN | ninein (GSK3B interacting protein) | PC-5p-113875_40 |
| NIN | ninein (GSK3B interacting protein) | PC-5p-233284_18 |
| NIN | ninein (GSK3B interacting protein) | aca-miR-425-3p_L-2R+1_1ss3AT |
| THSD7B | thrombospondin, type I, domain containing 7B | PC-5p-582426_7 |
| THSD7B | thrombospondin, type I, domain containing 7B | gga-miR-30a-5p_R+2_1ss13CT |
| THSD7B | thrombospondin, type I, domain containing 7B | gga-miR-30e-5p_R+5 |
| C3H6ORF72 | chromosome 3 open reading frame, human C6orf72 | gga-miR-1677-3p_L+1 |
| C5H14ORF166 | chromosome 5 open reading frame, human C14orf166 | gga-let-7g-3p_1ss22CT |
| C5H14ORF166 | chromosome 5 open reading frame, human C14orf166 | gga-miR-1677-3p_L+1 |
| C5H14ORF166 | chromosome 5 open reading frame, human C14orf166 | tgu-miR-2970-5p_1ss21GT |
| IYD | iodotyrosine deiodinase | aca-miR-18a-5p_R-2 |
| IYD | iodotyrosine deiodinase | chi-miR-16b-5p_R+1 |
| IYD | iodotyrosine deiodinase | gga-miR-16-5p |
| IYD | iodotyrosine deiodinase | gga-miR-30a-5p_R+2_1ss13CT |
| IYD | iodotyrosine deiodinase | gga-miR-30e-5p_R+5 |
| TXNDC16 | thioredoxin domain containing 16 | chi-miR-16b-5p_R+1 |
| TXNDC16 | thioredoxin domain containing 16 | gga-miR-16-5p |
| CUL1 | cullin 1 | PC-5p-37716_121 |
| CUL1 | cullin 1 | gga-let-7g-3p_1ss22CT |
| CUL1 | cullin 1 | tgu-miR-2970-5p_1ss21GT |
| NOL12 | nucleolar protein 12 | PC-3p-52432_89 |
| NOL12 | nucleolar protein 12 | gga-miR-30a-5p_R+2_1ss13CT |
| NOL12 | nucleolar protein 12 | gga-miR-30e-5p_R+5 |
| PDIA4 | protein disulfide isomerase family A, member 4 | PC-5p-97606_47 |
| GTDC1 | glycosyltransferase-like domain containing 1 | gga-miR-7468-3p_L+3 |
| PLEKHC1 | pleckstrin homology domain containing, family C (with FERM domain) member 1 | PC-3p-457530_9 |
| PLEKHC1 | pleckstrin homology domain containing, family C (with FERM domain) member 1 | chi-miR-16b-5p_R+1 |
| PLEKHC1 | pleckstrin homology domain containing, family C (with FERM domain) member 1 | gga-miR-16-5p |
| DDHD1 | DDHD domain containing 1 | hsa-miR-483-3p_L-1R+2 |
| ADCY1 | adenylate cyclase 1 (brain) | PC-5p-582426_7 |
| ADCY1 | adenylate cyclase 1 (brain) | gga-miR-1805-5p_L-1 |
| CYTH4 | cytohesin 4 | PC-3p-52432_89 |
| CYTH4 | cytohesin 4 | PC-5p-71982_65 |
| CYTH4 | cytohesin 4 | PC-5p-97606_47 |
| SOUL | SOUL protein | gga-miR-30a-5p_R+2_1ss13CT |
| SOUL | SOUL protein | gga-miR-30e-5p_R+5 |
| SOUL | SOUL protein | gga-miR-34b-5p_L-1R+1 |
| SOUL | SOUL protein | hsa-miR-483-3p_L-1R+2 |
| PRPF39 | PRP39 pre-mRNA processing factor 39 homolog (S. cerevisiae) | gga-let-7g-3p_1ss22CT |
| MPST | mercaptopyruvate sulfurtransferase | gga-miR-1782_L+2R-1 |
| MPST | mercaptopyruvate sulfurtransferase | gga-miR-1a-3p_R+1_1ss2GT |
| TST | thiosulfate sulfurtransferase (rhodanese) | PC-5p-582426_7 |
| TRIP13 | thyroid hormone receptor interactor 13 | aca-miR-363-3p_R+1 |
| TRIP13 | thyroid hormone receptor interactor 13 | oan-miR-363-3p_R+1 |
| PVALB | parvalbumin | gga-miR-34b-5p_L-1R+1 |
| MB | myoglobin | PC-5p-97606_47 |
| MCM5 | minichromosome maintenance complex component 5 | gga-miR-34b-5p_L-1R+1 |
| TOM1 | target of myb1 | PC-5p-97606_47 |
| CCDC148 | coiled-coil domain containing 148 | PC-5p-71982_65 |
| FBXO7 | F-box protein 7 | PC-5p-233284_18 |
| FBXO7 | F-box protein 7 | chi-miR-16b-5p_R+1 |
| FBXO7 | F-box protein 7 | gga-miR-16-5p |
| WDSUB1 | WD repeat, sterile alpha motif and U-box domain containing 1 | PC-5p-113875_40 |
| WDSUB1 | WD repeat, sterile alpha motif and U-box domain containing 1 | chi-miR-16b-5p_R+1 |
| WDSUB1 | WD repeat, sterile alpha motif and U-box domain containing 1 | gga-miR-16-5p |
| WDSUB1 | WD repeat, sterile alpha motif and U-box domain containing 1 | gga-miR-1805-5p_L-1 |
| WDSUB1 | WD repeat, sterile alpha motif and U-box domain containing 1 | gga-miR-1a-3p_R+1_1ss2GT |
| WDSUB1 | WD repeat, sterile alpha motif and U-box domain containing 1 | gga-miR-34b-5p_L-1R+1 |
| WDSUB1 | WD repeat, sterile alpha motif and U-box domain containing 1 | gga-miR-7468-3p_L+3 |
| TLE1 | transducin-like enhancer of split 1 (E(sp1) homolog, Drosophila) | PC-5p-233284_18 |
| TLE1 | transducin-like enhancer of split 1 (E(sp1) homolog, Drosophila) | gga-miR-1782_L+2R-1 |
| BAZ2B | bromodomain adjacent to zinc finger domain, 2B | PC-5p-582426_7 |
| BAZ2B | bromodomain adjacent to zinc finger domain, 2B | aca-miR-363-3p_R+1 |
| BAZ2B | bromodomain adjacent to zinc finger domain, 2B | oan-miR-363-3p_R+1 |
| BAZ2B | bromodomain adjacent to zinc finger domain, 2B | tgu-miR-2970-5p_1ss21GT |
| MARCH7 | membrane-associated ring finger (C3HC4) 7, E3 ubiquitin protein ligase | gga-miR-1677-3p_L+1 |
| GKAP1 | G kinase anchoring protein 1 | PC-5p-92816_49 |
| NAA20 | N(alpha)-acetyltransferase 20, NatB catalytic subunit | PC-3p-173245_25 |
| NAA20 | N(alpha)-acetyltransferase 20, NatB catalytic subunit | gga-miR-34b-5p_L-1R+1 |
| HNRNPK | heterogeneous nuclear ribonucleoprotein K-like | PC-5p-582426_7 |
| RMI1 | RMI1, RecQ mediated genome instability 1, homolog (S. cerevisiae) | PC-3p-173245_25 |
| RMI1 | RMI1, RecQ mediated genome instability 1, homolog (S. cerevisiae) | PC-5p-71982_65 |
| AGTPBP1 | ATP/GTP binding protein 1 | PC-3p-173245_25 |
| AGTPBP1 | ATP/GTP binding protein 1 | gga-let-7g-3p_1ss22CT |
| ISCA1 | iron-sulfur cluster assembly 1 homolog (S. cerevisiae) | aca-miR-363-3p_R+1 |
| ISCA1 | iron-sulfur cluster assembly 1 homolog (S. cerevisiae) | oan-miR-363-3p_R+1 |
| CTSL2 | cathepsin L2 | gga-miR-1782_L+2R-1 |
| C22ORF28 | chromosome 1 open reading frame, human C22orf28 | PC-3p-338480_12 |
| C22ORF28 | chromosome 1 open reading frame, human C22orf28 | PC-5p-97606_47 |
| FANCC | Fanconi anemia, complementation group C | PC-3p-457530_9 |
| FANCC | Fanconi anemia, complementation group C | PC-5p-233284_18 |
| PRDM4 | PR domain containing 4 | hsa-miR-483-3p_L-1R+2 |
| CFC1B | cripto, FRL-1, cryptic family 1B | PC-3p-457530_9 |
| RECK | reversion-inducing-cysteine-rich protein with kazal motifs | gga-miR-33-3p_L+1R+1 |
| RECK | reversion-inducing-cysteine-rich protein with kazal motifs | hsa-miR-483-3p_L-1R+2 |
| PWP1 | PWP1 homolog (S. cerevisiae) | PC-3p-52432_89 |
| PWP1 | PWP1 homolog (S. cerevisiae) | PC-5p-37716_121 |
| PWP1 | PWP1 homolog (S. cerevisiae) | chi-miR-16b-5p_R+1 |
| PWP1 | PWP1 homolog (S. cerevisiae) | gga-miR-16-5p |
| RBFA | ribosome binding factor A (putative) | PC-3p-338480_12 |
| TMEM263 | chromosome 1 open reading frame, human C12orf23 | PC-3p-457530_9 |
| RIC8B | resistance to inhibitors of cholinesterase 8 homolog B (C. elegans) | gga-miR-1805-5p_L-1 |
| RFX4 | regulatory factor X, 4 (influences HLA class II expression) | gga-let-7g-3p_1ss22CT |
| CTDP1 | CTD (carboxy-terminal domain, RNA polymerase II, polypeptide A) phosphatase, subunit 1 | gga-miR-1a-3p_R+1_1ss2GT |
| CTDP1 | CTD (carboxy-terminal domain, RNA polymerase II, polypeptide A) phosphatase, subunit 1 | gga-miR-7468-3p_L+3 |
| ALDH5A1 | aldehyde dehydrogenase 5 family, member A1 | gga-miR-33-3p_L+1R+1 |
| LOC100857286 | DCC-interacting protein 13-beta-like | PC-5p-113875_40 |
| DEK | DEK oncogene | gga-miR-1805-5p_L-1 |
| DEK | DEK oncogene | gga-miR-33-3p_L+1R+1 |
| KIF13A | kinesin family member 13A | gga-miR-30a-5p_R+2_1ss13CT |
| KIF13A | kinesin family member 13A | gga-miR-30e-5p_R+5 |
| C12ORF45 | chromosome 1 open reading frame, human C12orf45 | PC-5p-71982_65 |
| RANBP9 | RAN binding protein 9 | PC-5p-71982_65 |
| JARID2 | jumonji, AT rich interactive domain 2 | PC-5p-71982_65 |
| JARID2 | jumonji, AT rich interactive domain 2 | hsa-miR-483-3p_L-1R+2 |
| MYLIP | myosin regulatory light chain interacting protein | gga-miR-7468-3p_L+3 |
| RBM24 | RNA binding motif protein 24 | PC-5p-233284_18 |
| RBM24 | RNA binding motif protein 24 | chi-miR-16b-5p_R+1 |
| RBM24 | RNA binding motif protein 24 | gga-miR-16-5p |
| RBM24 | RNA binding motif protein 24 | gga-miR-1805-5p_L-1 |
| TXNRD1 | thioredoxin reductase 1 | gga-miR-1677-3p_L+1 |
| TXNRD1 | thioredoxin reductase 1 | gga-miR-1782_L+2R-1 |
| TXNRD1 | thioredoxin reductase 1 | tgu-miR-2970-5p_1ss21GT |
| NUP153 | nucleoporin 153kDa | gga-miR-1677-3p_L+1 |
| TDG | thymine-DNA glycosylase | PC-5p-71982_65 |
| TDG | thymine-DNA glycosylase | gga-miR-30a-5p_R+2_1ss13CT |
| TDG | thymine-DNA glycosylase | gga-miR-30e-5p_R+5 |
| TDG | thymine-DNA glycosylase | gga-miR-34b-5p_L-1R+1 |
| C1H12ORF48 | chromosome 1 open reading frame, human C12orf48 | PC-5p-92816_49 |
| C1H12ORF48 | chromosome 1 open reading frame, human C12orf48 | gga-miR-1a-3p_R+1_1ss2GT |
| C1H12ORF48 | chromosome 1 open reading frame, human C12orf48 | tgu-miR-2970-5p_1ss21GT |
| PMCH | pro-melanin-concentrating hormone | aca-miR-18a-5p_R-2 |
| LOC420864 | uncharacterized LOC420864 | gga-miR-1a-3p_R+1_1ss2GT |
| EEF1E1 | eukaryotic translation elongation factor 1 epsilon 1 | gga-miR-1782_L+2R-1 |
| EEF1E1 | eukaryotic translation elongation factor 1 epsilon 1 | gga-miR-34b-5p_L-1R+1 |
| BLOC1S5 | muted homolog (mouse) | PC-5p-113875_40 |
| TXNDC5 | thioredoxin domain containing 5 (endoplasmic reticulum) | PC-3p-52432_89 |
| TXNDC5 | thioredoxin domain containing 5 (endoplasmic reticulum) | chi-miR-16b-5p_R+1 |
| TXNDC5 | thioredoxin domain containing 5 (endoplasmic reticulum) | gga-let-7g-3p_1ss22CT |
| TXNDC5 | thioredoxin domain containing 5 (endoplasmic reticulum) | gga-miR-16-5p |
| TXNDC5 | thioredoxin domain containing 5 (endoplasmic reticulum) | gga-miR-1782_L+2R-1 |
| TXNDC5 | thioredoxin domain containing 5 (endoplasmic reticulum) | gga-miR-30a-5p_R+2_1ss13CT |
| TXNDC5 | thioredoxin domain containing 5 (endoplasmic reticulum) | gga-miR-30e-5p_R+5 |
| TXNDC5 | thioredoxin domain containing 5 (endoplasmic reticulum) | gga-miR-33-3p_L+1R+1 |
| TXNDC5 | thioredoxin domain containing 5 (endoplasmic reticulum) | hsa-miR-483-3p_L-1R+2 |
| PARP12 | poly (ADP-ribose) polymerase family, member 12 | gga-miR-1677-3p_L+1 |
| KIAA1549 | KIAA1549 | gga-miR-1677-3p_L+1 |
| SSR1 | signal sequence receptor, alpha | PC-5p-582426_7 |
| SSR1 | signal sequence receptor, alpha | chi-miR-16b-5p_R+1 |
| SSR1 | signal sequence receptor, alpha | gga-let-7g-3p_1ss22CT |
| SSR1 | signal sequence receptor, alpha | gga-miR-16-5p |
| FARS2 | phenylalanyl-tRNA synthetase 2, mitochondrial | PC-5p-97606_47 |
| RPP40 | ribonuclease P/MRP 40kDa subunit | gga-miR-34b-5p_L-1R+1 |
| ECI2 | enoyl-CoA delta isomerase 2 | PC-5p-92816_49 |
| ECI2 | enoyl-CoA delta isomerase 2 | PC-5p-97606_47 |
| PRPF4B | PRP4 pre-mRNA processing factor 4 homolog B (yeast) | gga-miR-7468-3p_L+3 |
| RIPK1 | receptor (TNFRSF)-interacting serine-threonine kinase 1 | PC-5p-582426_7 |
| RIPK1 | receptor (TNFRSF)-interacting serine-threonine kinase 1 | PC-5p-71982_65 |
| RIPK1 | receptor (TNFRSF)-interacting serine-threonine kinase 1 | PC-5p-97606_47 |
| IRF4 | interferon regulatory factor 4 | PC-3p-52432_89 |
| IRF4 | interferon regulatory factor 4 | PC-5p-113875_40 |
| IRF4 | interferon regulatory factor 4 | PC-5p-71982_65 |
| SLC37A3 | solute carrier family 37 (glycerol-3-phosphate transporter), member 3 | PC-3p-52432_89 |
| SLC37A3 | solute carrier family 37 (glycerol-3-phosphate transporter), member 3 | aca-miR-363-3p_R+1 |
| SLC37A3 | solute carrier family 37 (glycerol-3-phosphate transporter), member 3 | gga-miR-1456-5p_L-1 |
| SLC37A3 | solute carrier family 37 (glycerol-3-phosphate transporter), member 3 | gga-miR-16-5p |
| SLC37A3 | solute carrier family 37 (glycerol-3-phosphate transporter), member 3 | oan-miR-363-3p_R+1 |
| MKRN1 | makorin ring finger protein 1 | PC-5p-113875_40 |
| MKRN1 | makorin ring finger protein 1 | PC-5p-71982_65 |
| MKRN1 | makorin ring finger protein 1 | chi-miR-16b-5p_R+1 |
| MKRN1 | makorin ring finger protein 1 | gga-miR-16-5p |
| MKRN1 | makorin ring finger protein 1 | gga-miR-30a-5p_R+2_1ss13CT |
| MKRN1 | makorin ring finger protein 1 | gga-miR-30e-5p_R+5 |
| WRNIP1 | Werner helicase interacting protein 1 | chi-miR-16b-5p_R+1 |
[truncated: 679,032 more chars]
